# Hybrid approach to sieve out natural compounds against dual targets in Alzheimer’s Disease

**DOI:** 10.1038/s41598-019-40271-9

**Published:** 2019-03-06

**Authors:** Sucharita Das, Sandipan Chakraborty, Soumalee Basu

**Affiliations:** 1 Department of Microbiology, University of Calcutta, 35 Ballygunge Circular Road, Kolkata, 700 019 India; 2 School of Chemical Sciences, Indian Association for the Cultivation of Science, Jadavpur, Kolkata, 700032 India

## Abstract

Excess Aβ production by the key protease BACE1, results in Aβ aggregation, forming amyloid plaques, all of which contribute to the pathogenesis of Alzheimer**’**s disease. Besides the multi-factorial nature of the disease, the diversity in the size and shape of known ligands that bind to the active site of BACE1, that is the flexibility of the enzyme, pose a serious challenge for the identification of drug candidates. To address the issue of receptor flexibility we have carried out ensemble docking with multiple receptor conformations. Therein, two representative structures each from closed and semi-open BACE1 conformations were selected for virtual screening to identify compounds that bind to the active site of both the conformations. These outperformed compounds were ranked using pharmacophore models generated by a ligand-based approach, for the identification of BACE1 inhibitors. The inhibitors were further predicted for anti-amyloidogenic activity using a QSAR model already established by our group thus enlisting compounds with dual potency. BACE1 inhibitory and anti-amyloidogenic activity for the commercially available compounds were validated using *in vitro* studies. Thus, incorporation of receptor flexibility in BACE1 through ensemble docking in conjunction with structure and ligand-based approach for screening might act as an effective protocol for obtaining promising scaffolds against AD.

## Introduction

Alzheimer’s disease (AD) is a debilitating disorder that has become one of the most common forms of dementia. It is characterised by loss of functional neurons and synapses leading to disrupted communication amongst nerve cells effecting in irreversible decline in intellectual abilities. Neuropathological symptoms of the disease include the presence of extracellular amyloid plaques and intracellular neurofibrillary tangles. Other pathophysiological aspects of AD include oxidative damage, mitochondrial dysfunction, failure of molecular transport mechanisms, inflammation and cell-cycle dysregulation. However, out of these, the amyloid hypothesis, according to which accumulation of Aβ in the brain drives AD pathogenesis, wins the spotlight^1^. The rest of the disease process, including formation of neurofibrillary tangles, is believed to be a result of imbalance between Aβ production and Aβ clearance. Aβ is known to be generated upon sequential cleavage of the amyloid precursor protein (APP) by β-secretase (BACE1) followed by γ-secretase. Thus inhibition of BACE1 has emerged as a prime treatment strategy for the disease not only because cleavage by BACE1 is the rate limiting step of Aβ production more because BACE1 knock-out mice have been found to be healthy^2^ unlike γ-secretase knock-out mice.

Several innovative methods to develop inhibitors against BACE1 have been adopted accordingly. The first generation inhibitors consisted of peptide based transition state analogs^3–5^. They were highly potent since they could occupy several sub-pockets of BACE1. But their undesirable pharmacological properties made them unfit as drugs. Thereafter the second generation small molecule inhibitors covering varied classes of molecules like imidazolidinone analogs, hydroxymethylcarbonyl isosteres, pyridinium-based derivatives, flavonoids and acylguanidines^6–10^ were developed which were more drug-like. These compounds emerged from advanced techniques like iterative X-ray crystallography, NMR, Surface Plasmon Resonance (SPR) to name a few. Several of them displayed improved CNS (central nervous system) penetration^11,12^. Despite this, many of these compounds could not make it to the Phase 1 clinical trials due to severe side effects^13,14^. It may be iterated that parallel to these efforts, novel methods of computer-based drug designing was customarily pursued^15–18^.

Ongoing treatment for the disease improves or stabilizes the symptoms of dementia by increasing the communication between the nerve cells. Currently approved medications for the disease include the cholinesterase inhibitors and NMDA receptor antagonists like Donepezil, Rivastigmine, Galantamine and Memantine that provide symptomatic relief without addressing the neuropathology^19–21^. Hence the search for BACE1 inhibitors as drugs against AD still continues to be a hot pursuit.

**Figure 1:**
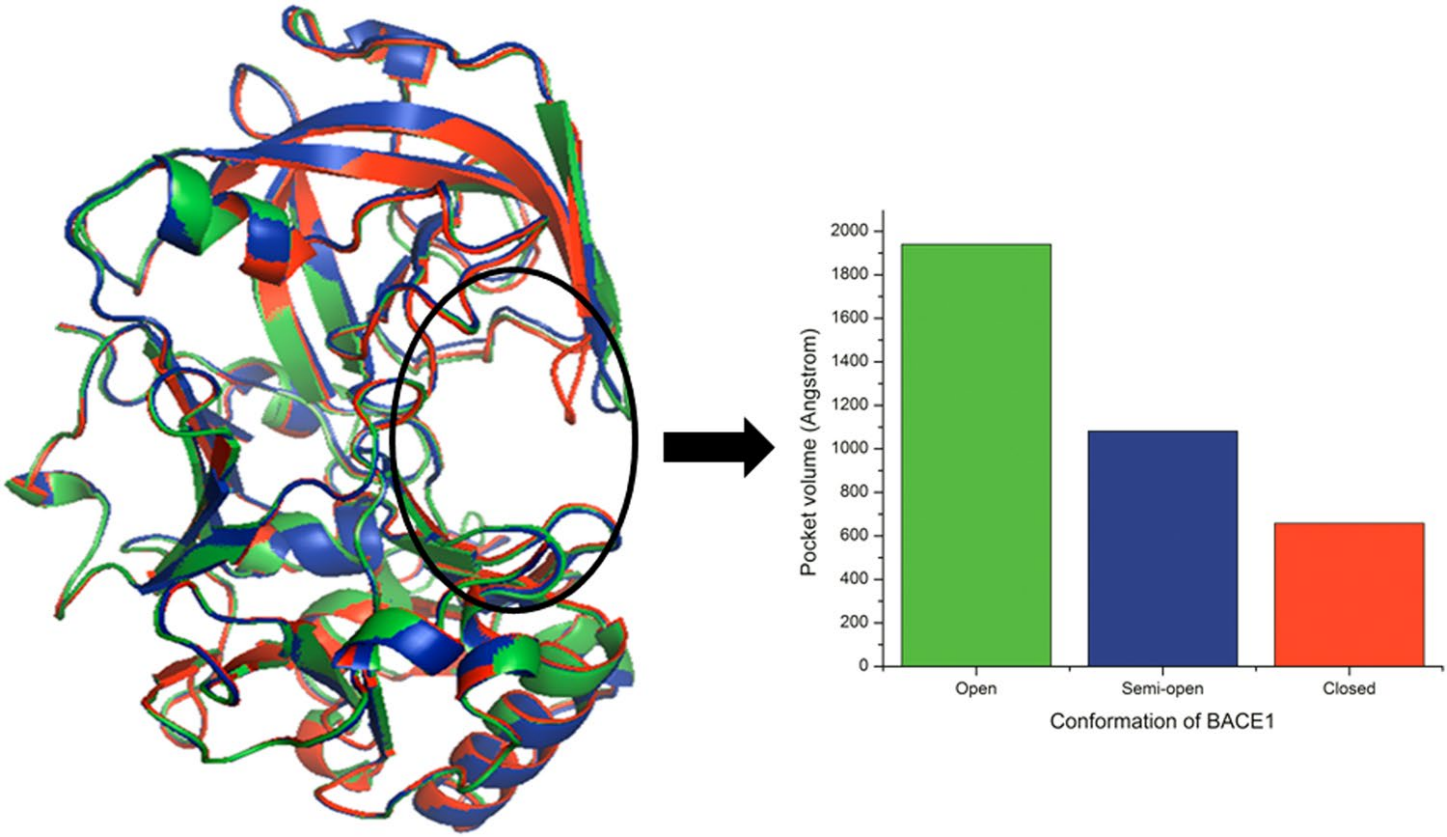
Three superimposed crystal structures of BACE1 representing the three different conformations: Flexible loops of the active site of open (green: 1SGZ), semi-open (blue: 4DJX) and closed (red: 3CIC) BACE1 structures show decreasing pocket volume (inset bar graph).

As a consequence of this ongoing hunt, we find hundreds of BACE1 crystal structures in complex with inhibitors of different size and shape in the public structural repository, PDB. Comparison of the different crystal structures of BACE1-inhibitor complexes and their molecular dynamics studies show BACE1 to be quite flexible. The conformational flexibility of BACE1 enables it to switch from open to closed form upon ligand binding^22,23^ (Fig. 1). It has been observed that in the presence of peptide-like bound inhibitors, the flap moves closer to the catalytic aspartate dyad thus representing a closed conformation. Recent studies have revealed that upon binding with some special classes of inhibitors, the flap, due to steric clashes, adopts a semi-open form moving away from the dyads than in the closed form^24^. Also an extensive study by Xu *et al*.^25^ proved the existence of semi-open conformation of BACE1 exhibiting space group specificity with P6122. The space group specificity was however ruled out by a series of fragment screening approaches that have identified complexes of other space groups with a semi-open form too^26–30^. Thus, to summarize, different classes of inhibitors may interact with the flap residues to a greater or lesser extent generating varied flap conformations of the protein. It is worth mentioning that lack of protein flexibility in structure-based drug designing severely limits the identification of true ligands as flexibility leads to structural rearrangement of the binding site and the adjoining structures, thus influencing ligand binding. Previous efforts of inhibitor identification have used a single BACE1 structure for virtual screening without consideration of the flap flexibility^31,32^. So, BACE1 structure in which ligands of different inhibitor classes can dock needs to be determined^33^.

**Figure 2:**
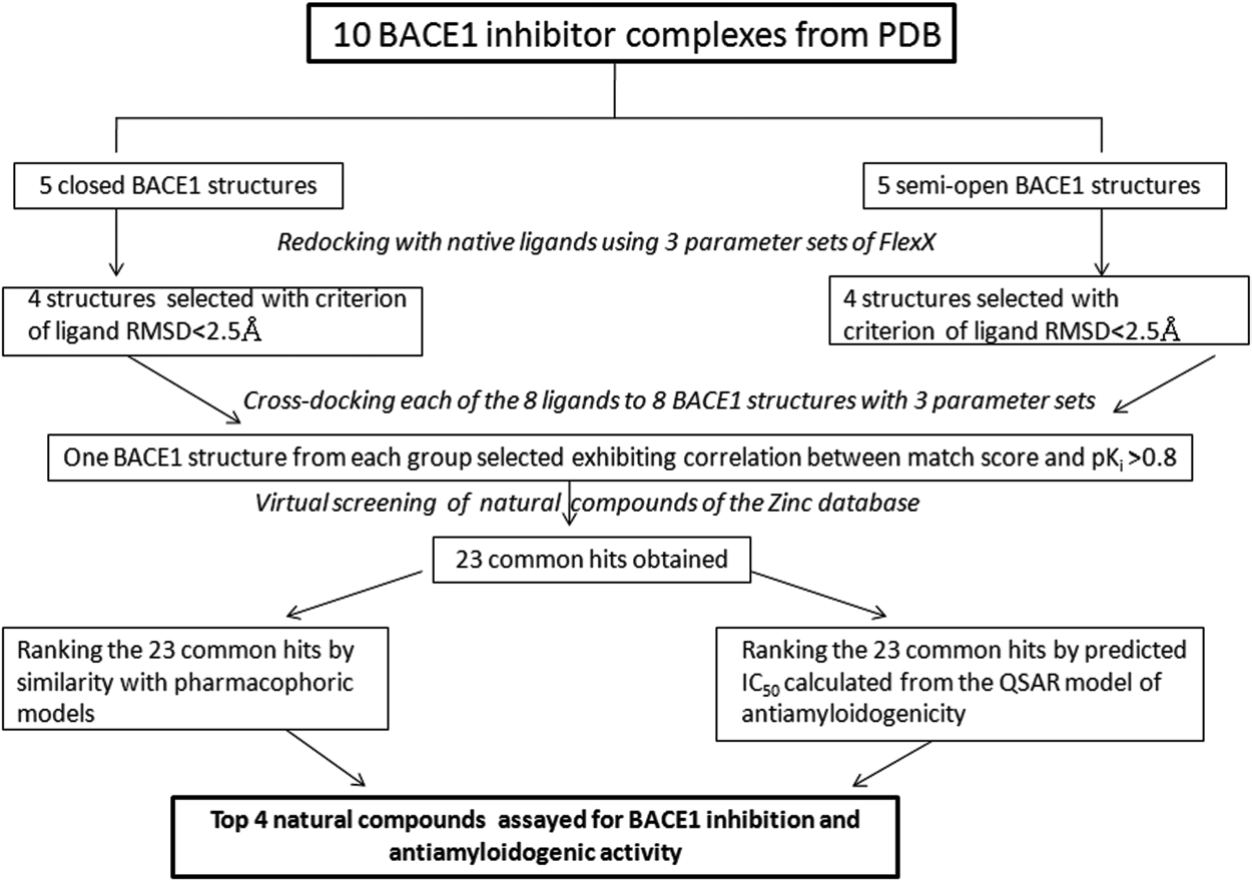
Flowchart showing the step by step progression of the work.

One of the major problems related to design of inhibitors of enzymes is the consideration of the intrinsic flexibility of the binding site of the receptor, during docking. Scientists have been trying endlessly to incorporate flexibility using either combination of crystal and NMR structures or using computational methods^34^ by combination of rotamers for receptor side chain conformations or sampling side chain torsion angle space^35^ or even considering backbone flexibility through ensemble docking^36–38^. Recently, Hou *et al*. have shown the success of appropriately using multiple structures for the open and closed conformation of the WPD loop of the lymphoid-specific tyrosine phosphatase (LYP) to virtually screen LYP inhibitors^39^. Here, we have considered multiple crystal structures of closed and semi-open conformation of BACE1 taken from complexes with ligands of wide range of size and shape, for the fortification of receptor flexibility. It needs special mention at this point that out of 334 BACE1-inhibitor crystal structures in the PDB, none has a ligand attached to the open conformation of BACE1. Therefore, in this study, open conformation of BACE1 was excluded as an option while considering multiple crystal structures. Using multiple representative conformations of the enzyme, a database of natural compounds was screened virtually as inhibitors. These screened compounds that were capable of binding to both semi-open and closed conformation of BACE1 were further assessed for their anti-amyloidogenic activities using an already established QSAR model of our group. The flow of work is schematically depicted in Fig. 2 and discussed in details in the Methods section.

## Methods for Computational Studies

### Grouping of the structures

Structures of BACE1-inhibitor complexes having resolution <2.5 Å, no missing residues, and known K_i_ were downloaded from the PDB database. The maximum number of semi-open conformations satisfying the above criteria was found to be five. We therefore formed the dataset with altogether ten members including five more receptor structures in the closed conformation. The receptor structures were divided into two groups based on three distances - the depth of the active site cleft given by the distances between the flap tip and the two catalytic dyads individually (*d1*, *d2*) and width of the mouth of the active site cleft (d3). Here *d1* is the Cβ D32-Cα T72 distance, *d2* is the Cα D228-Cα T72 and *d3* is the Cα S325-Cα T72 distance. While one group consisted of semi-open conformations (PDB IDs: 4DJX, 4FS4, 4HA5, 4H3G, 4H3F) with *d1*, *d2*, *d3* ranging from 14.3 to 15 Å and the other group consisted of flap closed conformation (PDB IDs: 2P4J, 2G94, 2QMG, 3LPK and 3CIC) with *d1*, *d2*, *d3* ranging from 11.6 to 13.9 Å. Active site RMSD was calculated in Pymol^40^ (version downloaded in 2014) considering the flap region (67–77), 10 s loop (9–14), A-loop (residues 158–167), F-loop (residues 311–318) and D-loop (residues 270–273)^41^.

### Protein preparation

Water molecules from the crystal structures were removed. One of the aspartates (D32) of the aspartate dyad of BACE1 was protonated^23^ for all the docking studies. Binding site was determined individually for each of the ten structures choosing the corresponding ligand as a reference ligand and specifying a radius of 13 Å.

### Docking

All docking studies were carried out using the FlexX module^42^. The hybrid approach in which the ligand binding is driven by enthalpy and entropy was used as the docking strategy. During each run, default values for access scaling and all the stereo modes (E/Z, R/S, pseudo R/S) were considered. Most of the docking studies carried out previously using FlexX, have used the default values of maximum number of solutions per iterations and maximum number of solutions per fragmentations. However, we have varied the default values of these parameters to generate two more sets of parameters. This was done to check if changing the default parameters led to better docking results. So, three different parameter sets (200 and 200; 125 and 300; 400 and 400) with maximum number of solutions per iterations and maximum number of solutions per fragmentations were considered during re-docking and cross docking.

### Re-docking

Each inhibitor was re-docked to its native BACE1 crystal structure using the three parameter sets mentioned above. RMSD between the crystal conformation of the ligand and the docked pose was calculated and a cut off of 2.5 Å was selected as a criterion for the subsequent cross-docking studies. During re-docking of inhibitors the common interactions were analysed and the presence of any two of the favourable interactions were used as a criterion to identify hits in virtual screening. The criterion was first determined manually and reaffirmed by the FlexX-Pharm docking.

### Cross-docking

Cross-docking studies were carried out between inhibitors of varied sizes and receptors with varied volume of the active sites using the FlexX software. In FlexX, “Match score” is calculated from the contribution of the matched interacting groups. Therefore, a correlation between the Match score and pK_i_ of the BACE1-inhibitor complexes selected from re-docking was obtained to determine which receptor could dock to inhibitors of varied sizes with favourable interactions. R^2^ > 0.7 was considered to be a criterion for good correlation. The p-value and F-value for the obtained correlation between Match scores and pK_i_ of the BACE1-inhibitor complexes were calculated for top ranked poses using the online server Wessa^43^. P-value < 0.05 and F-Test value $\gg$1 was considered to be significant. The BACE1 structure that resulted in highest squared correlation coefficient (R^2^) was selected from each group (semi-open and closed flap conformations) for virtual screening. Using the correlation plot a hit selection criterion of Match score <−29 kJ/mol was set to screen hits from the database of compounds. Detailed analysis of the interactions was carried out using the Accelrys Discovery Studio Visualizer^44^.

### MM-GBSA calculations

The binding energies of the re-docked and cross-docked complexes were obtained by using the MM-GBSA option of the prime submenu in the Schrodinger software^45^. It may be noted that the MM-GBSA energies for the common hits obtained from virtual screening of the compounds with 4H3G and 2P4J as receptors were also calculated using the same menu.

### Determining squared correlation coefficient for compounds with similar scaffolds

We have collected the structure activity data reported for the ligands of 2P4J^46^ and 4H3G^47^ from Ghosh *et al*. and Mandal *et al*. The ligands were minimized using molecular mechanics force field in Hyperchem^48^ to a convergence of 0.003 kcal Å^−1^ mol^−1^ and docked to their respective BACE1 structures. A correlation was drawn between pK_i_ and Match score.

### Virtual Screening

Natural compounds in standard delay format (sdf) were downloaded from the natural product database, ZINC, and filtered by the Lipinski filter, using DruLiTo^49^. Filtered compounds were screened by docking to the two selected semi-open and closed form of BACE1 structures using the FlexX docking program. Compounds showing interactions with frequently interacting BACE1 residues obtained from re-docking and exhibiting a Match score below −29 kcal/mol were considered as hits.

### Generation of Pharmacophore Model and Pharmacophore Screening Using PharmaGist

A pharmacophore based ranking of the common hits from both forms of BACE1 was carried out. PharmaGist^50,51^ was used to generate candidate pharmacophore model using known BACE1 inhibitors^52–59^ that were obtained from BindingDB^60^. Out of the input ligands one of the ligand is randomly selected as pivot by the algorithm. The method then identifies rigid groups in the remaining ligands and generates a set of transformations for superimposing the target rigid group onto the pivot. The superimpositions are reassembled into new conformations of the target ligand, aligned on the pivot and the alignment is scored by the weighted sum of the matched pivot features. A feature from pivot can be matched to a conformation of the target ligand, if they are of the same type and the distance between them is below a predefined threshold. The distance threshold for centres of aromatic rings is 1.8 Å and for the centres of the other feature types is 1.4 Å. The default weight values are 1 for each feature type except for hydrophobicity which is 0.3 and aromatic rings for which it is 3. The score of a pairwise alignment with a subset {*f*_*i*_} of matched pivot features is ∑_*i*_*s*(*f*_*i*_), where *s*(*f*_*i*_) is the score for matching the pivot feature *f*_*i*_. The high-scoring pairwise alignments are combined into multiple alignments in order to find subsets of pivot features that are matched by several pairwise alignments with different target ligands. The score of a multiple alignment is given by (*m* + 1)^1/2^·∑_*i*_*s*(*f*_*i*_), where *m* is the number of participating target ligands. Candidate pharmacophores are reported on the basis of this score. In our study, the ranking of the common hits was carried out based on the similarity of these molecules to the pharmacophore thus obtained. It may be mentioned that this similarity was measured using the same method of pairwise alignment used for pharmacophore generation. The default score values for matching the pivot features were used.

We used two different models to rank the common hits. Model 1 has been built using 12 known BACE1 inhibitors (IC_50_ varying from 0.7–49 nM) and Model 2 was generated from 9 known BACE1 inhibitors (K_i_ varying from 0.3–270 nM).

### Molecular overlay

Both the models were also validated by aligning to a reference pharmacophore corresponding to each set of input molecules. The reference pharmacophore was constructed by superposition of the BACE1-inhibitor complexes of each set of molecules, using Discovery Studio visualizer. The reference pharmacophore was then aligned to the PharmaGist generated pharmacophore using the molecular overlay tool of Discovery Studio visualizer.

### Enrichment factor calculation

We investigated the effectiveness of the pharmacophore based ranking methodology using enrichment studies. Enrichment factor of the models was computed on a DUD (Directory of useful decoys) data set of BACE1, consisting of 10 actives and 250 decoys, using the following equation:

$$E{F}_{ \% }=\frac{\frac{{N}_{active( \% )}}{{N}_{( \% )}}}{\frac{{N}_{active}}{{N}_{all}}}$$

where *N*_active*(*%*)*_ is the number of active compounds in a subset of the library, *N*_(*%*)_ is the number of compounds in the subset of the library, *N*_active_ and *N*_all_ are the total number of active compounds and the total number of compounds in the library.

### Assessing the applicability domain of the in-house build QSAR models

In a previous study, we have established a QSAR model for antiamyloidogenicity^61^. We checked the applicability of the model on the 23 common hits. The applicability of the QSAR was checked using the PCA Bounding Box method. This method defines the applicability domain by the projection of molecules in the principal component space considering the maximum and minimum values of the principal components^62^. The molecules that were found to lie within the applicability domain of the QSAR were considered for prediction of their activities by the QSAR.

### Prediction of anti-amyloidogenic activity

The common hits thus retrieved were subjected to further screening and ranking by an in-house built QSAR model of anti-amyloidogenic activity. The training set comprised of 18 polyphenols 5 of which are known to inhibit BACE1^61^. All the molecules were minimized using HyperChem with AM1 semi-empirical method to a gradient of 0.003 kcal/Å mol. Descriptor calculations were then carried out using the Codessa software^63^. Prior to the calculation of quantum-chemical and thermodynamic properties, the optimized structures were subjected to wave-function optimization using AM1 semi-empirical method of the AMPAC software^64^. The model with the highest predicted R^2^, generated using heuristic approach was considered. The high predictive potential of the model was ascertained using the statistical parameters such as R^2^ and F-Test values of 0.9135 and 34.31. Principal component analysis was used to characterize the outliers. Additionally cross validation technique was used to validate the QSAR model with R^2^_CV_ of 0.844. The model has been previously used for screening natural compounds such as flavonoids, flavonoid derivatives, alkaloids, stilbenes and terpenoids. These screened compounds have exhibited anti-amyloidogenicity and BACE1 inhibitory activities^65,66^.

The QSAR equation used for generating a list of top 23 compounds is as follows:

1 $$\begin{array}{rcl}LogI{C}_{50} & = & -11.244-0.012\ast (HBSA)+5.762\ast (AICO)\\  &  & +\,0.893\ast (ESP\,-\,RNCS)+1.767\ast (VE)\end{array}$$

where HBSA is hydrogen bonding surface area, AICO is the average information content that reflects the shape and symmetry, ESP-RNCS is the relative negative charged surface area and VE is the vibrational entropy (300 K)/no of atoms.

### Materials and methods for *in vitro* studies

The commercially available screened compounds of ZINC database were procured from Interbioscreen. BACE1 assay kit and HFIP were procured from Sigma Aldrich while Aβ_25–35_ was bought from Anaspec in powdered form.

### BACE1 inhibition assay

BACE1 activity assay kit was used to determine the BACE1 inhibition ability of the screened compounds. The substrate is fluorescent labelled with donor on one side and quencher group on the other side. In the absence of BACE1, due to FRET (Fluorescence resonance energy transfer) the donor fluorescence of the substrate is quenched. Upon cleavage of the labelled substrate by BACE1 the donor and quenching groups are separated, hence fluorescence increases. Therefore inhibition of BACE1 by an inhibitor would result in lesser substrate cleavage and hence lead to a decrease in fluorescence. In our study, concentrated stock solutions of the screened compounds were prepared by dissolving the compounds in DMSO. Final concentration of the screened compounds was 1 µM. A known inhibitor (myricetin) and a weak inhibitor (gossypetin) were also included in the assay. The assay was carried out according to the manufacturer’s instructions. The reaction mixture was incubated at 37 °C for 2 hours. Fluorescence was then measured by excitation at 320 nm and emission at 405 nm. The inhibition percentage was obtained by the following equation:

$${\rm{Single}}\,{\rm{point}}\,{\rm{inhibition}}\, \% \,({{\rm{SPI}}}_{1{\rm{\mu }}{\rm{M}}})=({\rm{1}}-\frac{S-{S}_{{\rm{0}}}}{C-{C}_{{\rm{0}}}})\times {\rm{100}}$$

where C is the fluorescence of the control (containing enzyme, assay buffer and substrate) after two hours of incubation, C_0_ is the fluorescence of the control at the start, S is the fluorescence of the compound solution (containing enzyme, assay buffer, compound solution and substrate) after two hours of incubation and S_0_ is the fluorescence of the compound solution at start.

### Thioflavin T assay

The dye Thioflavin T (ThT), is used to quantify amyloid aggregates. On binding to the beta sheets of amyloid aggregates, the rotation of the ThT rings is hampered thus preserving the excited states leading to fluorescence enhancement. The Aβ_25–35_ sample was prepared by dissolving it in HFIP to remove any preformed aggregates and keeping aliquots at −80 °C. On the day of assay, aliquot of the HFIP dissolved Aβ_25–35_ (final concentration 100 µM) was lyophilized. Thereafter the lyophilized sample was dissolved in phosphate buffer. A stock solution of the compounds with concentration of 1 mM was prepared in DMSO. The compounds with final concentration of 100 µM were co-incubated with Aβ_25–35_ for 72 hours at 37 °C. The co-incubated samples were diluted five times. A stock solution of Thioflavin T was prepared in DMSO and final concentration of 15 µM was added. The fluorescence was measured by excitation at 440 nm and emission at 460–550 nm.

## Results and Discussion

### Classification of the BACE1 conformations from crystal structures of complexes

We have excluded open BACE1 conformation from our study as none from about 300 ligand bound crystal structures from PDB were in the open conformation. Ten BACE1-ligand complexes with no missing residues were chosen and grouped into semi-open and closed structures on the basis of the depth of the active site judged by the flap tip-catalytic dyad distances (d1 and d2) and width of the mouth of active site given by the distance d3 respectively. Table 1 shows the RMSD of the flexible regions near the active site (as mentioned in the Methods section) between structures within and across the groups. PDB IDs from row or column 2 to 6 represent the semi-open conformations and comprise of Group1 while those from row or column 7 to 11 represent closed conformations and comprise of Group2. Within Group 1, the RMSD varied between 0.2–0.37 Å and surprisingly a wider range of 0.28–1.48 Å was observed within Group 2. Within Group 2, the range widened as a result of the differences in the conformation of the flap residues and also other loops near the active site of these closed structures. Thus it may be noted that the active site conformation of the closed structures are more diverse than those of the semi-open structures. The RMSD across groups (bold faced values in Table 1) varied from 0.6 to 1.63 Å. Out of all the pairs the most deviated active site conformations were found between 4H3G, 4HA5, 4FS4 (Group 1) and 2P4J (Group 2).

**Table 1:** RMSD in Å between the flexible regions near the active site of the BACE1 structures.

PDB ID	4H3G	4DJX	4HA5	4FS4	4H3F	3CIC	3LPK	2P4J	2QMG	2G94
4H3G	0	0.24	0.34	0.36	0.33	**0**.**65**	**0**.**61**	**1**.**62**	**0**.**69**	**1**.**1**
4DJX		0	0.27	0.25	0.27	**0**.**66**	**0**.**6**	**1**.**6**	**0**.**68**	**1**.**09**
4HA5			0	0.2	0.3	**0**.**74**	**0**.**72**	**1**.**63**	**0**.**76**	**1**.**13**
4FS4				0	0.27	**0**.**72**	**0**.**69**	**1**.**62**	**0**.**73**	**1**.**1**
4H3F					0	**0**.**75**	**0**.**72**	**1**.**61**	**0**.**75**	**1**.**12**
3CIC						0	0.28	1.47	0.27	0.81
3LPK							0	1.49	0.35	0.84
2P4J								0	1.47	1.42
2QMG									0	0.75
2G94										0

Unlike the RMSD values which varied within the same group of BACE1 conformation, we observed that the distances d1, d2 and d3 followed almost uniform values within the same group. Hence these distances would be better to classify the open and closed conformations of BACE1 (Supplementary Fig. S1 and Supplementary Table S1).

### Re-docking

Re-docking each ligand (Supplementary Table S2) to their respective BACE1 crystal structure was carried out to test the performance of the docking algorithm on the dataset. Using varied parameter sets, root mean square deviation (RMSD) was calculated between the re-docked ligand and the crystallographic conformation of the ligand. Ligands with an RMSD < 2.5 Å on re-docking were assumed to generate a docked pose relevant to the crystal pose and hence were considered for cross-docking. However in cases of failed re-docking (36.6% of the total re-docking studies), the corresponding BACE1 structures were excluded from further cross-docking studies. Thus the ligand of each of the ten BACE1 structures was re-docked using three different parameter sets (described in the docking sub-section of methods section). Re-docking studies with the parameter sets resulted in different RMSD of the ligands tabulated in Supplementary Table S3. Exception was the ligand of 4FS4 which showed no change in RMSD across all the three parameter sets. Two ligand structures 4H3F_L_ and 2G94_L,_ upon re-docking showed RMSD > 2.5 Å hence further studies using these receptor-ligand complexes were not carried out. Consistent high RMSD in case of 2G94 for all the parameter sets might have resulted from high number of rotatable bonds in the corresponding ligand (Supplementary Table S2). In case of the complexes 4H3G and 2P4J, only parameter set 3 could generate an RMSD < 2.5 Å. Also it is noteworthy that ligand RMSD with parameter set 3 has gone above 2.5 Å for two receptor structures in comparison to four and five for the other two parameter sets thus marking parameter set 3 superior over the two.

Excluding 4H3F and 2G94, the rest of the eight complexes were analyzed for the identification of the enzyme-inhibitor interactions. For complexes in the closed BACE1 conformations, the common hydrogen bond interactions included T232, G230, Q73, T72, G34 and either of the aspartate dyads D228 and D32. In case of complexes with semi-open BACE1 conformations, van der Waal’s interaction was much more prominent and hydrogen bonding interaction was lesser. However in these complexes hydrogen bonds with both D228 and D32 were observed, strengthening the interaction in the absence of the proximity of the flap residues. Stacking interactions with F108 and Y71 were observed both in semi-open and closed conformations. A study by Liu *et al*.^67^ corroborated these findings as they showed that van der Waals interactions with Y71 and H-bonding with T72 stabilize the BACE1-inhibitor complex. They also showed that H-bond interactions with G230 of S3 subpocket and T232 of S2 subpocket are favourable interactions that occur in several BACE1 inhibitors. In the present study, H-bond interactions with BACE1 residues, T72, G230 and T232 and π-stacking interaction with Y71 were considered to be important and therefore presence of any two of these favourable interactions were used as a criterion for the selection of hits during virtual screening. It is worth mentioning that since even a weak inhibitor forms hydrogen bond with D228 and/or D32 therefore favourable interaction with either of them was kept as a default criterion.

### Cross-docking

Cross-docking studies were carried out with receptors and inhibitors taken from eight complexes selected from re-docking. For each receptor, a correlation was obtained using eight Match scores (from a self-docking and seven cross docking studies) and eight corresponding inhibition constants. The purpose of the study was to identify BACE1 structures that could show reasonable agreement between Match scores and activity data (pK_i_) for varied inhibitor sizes and also to set a threshold Match score for virtual screening.

**Figure 3:**
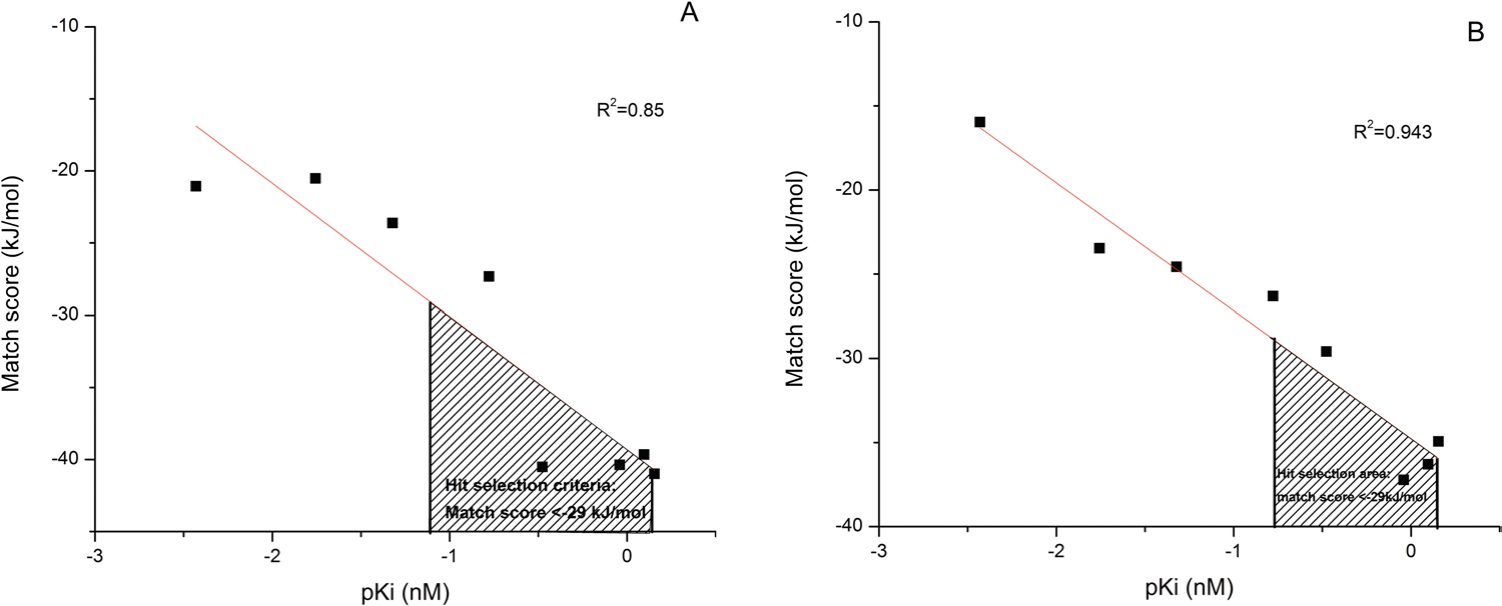
Linear correlation plots for 2P4J and 4H3G. pK_i_ versus Match Score for the receptors (**A**) 2P4J and (**B**) 4H3G. The shaded region of the graphs corresponds to the highly active inhibitors of BACE1 with a Match score <−29 kJ/mol which has been used as a selection criterion during virtual screening.

The cross-docked complexes within a group share interaction profiles similar to the cognate pair indicated by the bold-faced residues in Table 2. However it was found that the original pose could not be recovered in several cases as evident from the corresponding interaction profiles (Supplementary Table S4). It was observed that docking with closed BACE1 conformations were less correlated (R^2^ < 0.8) to the inhibition constants except for the structure of 2P4J (Table 3). Docking of inhibitors with few rotatable bonds to the small cavity of the closed structures (Supplementary Tables S1 and S2) probably resulted in steric clashes leading to poor scores. On the other hand, large inhibitors with high number of rotatable bonds occupied several subpockets and thus gave better scores. Moreover, lesser steric clashes due to larger cavity volume could be the reason for better correlation as observed in case of semi-open conformation over the closed one (Table 3). Hence receptors 4H3G from the semi-open and 2P4J from the closed conformation have been selected that could favourably interact with BACE1 inhibitors of varied sizes. Best R^2^ along with permissible F-test and p-values were considered for the selection. It may be emphasized that the pair showed considerable deviation of the flexible regions of the active site (Table 1) implying distinct representation of the two conformations of the receptor, BACE1. Incidentally, cross-docking with parameter set 3 showed the highest R^2^ value which was in corroboration with the re-docking studies where too parameter set 3 outperformed. Figure 3A,B shows the dependence of Match score, calculated from parameter set 3, on the pK_i_ for the receptors 4H3G and 2P4J respectively. It is clear for both the receptors that a score higher than −29 kJ/mol corresponds to high pK_i_ or low inhibition constant. Thus, a Match score less than −29 kJ/mol was regarded as a threshold during the screening of ligands as inhibitors.

**Table 2:** Comparison of the interacting residues involved in H-bonds, Match scores and MM-GBSA energies for the re-docked and cross-docked complexes (rank 1 poses for parameter set 3) used in the study. For more details refer to the note^§^.

Ligands	23I	SC6	316	Z76	10Q	0KQ	13W	H24
BACE1								
	*T72*, *Q73*, *Y198* *D228*, *G230*, *T231*, *T232*, *N23*, *S325*.	D32, **G34**, T72, R128, **Y198**.	**Q73**, **G230** **T232**, R235, S325.	D32, **G34**, T72, **Q73**, R128, **Y198**.	S35, T72, Q73, S325.	**D32**, T72, R128.	**D32**, S36, N37, T72, Q73.	**D32**, T72, G230, R235, S325.
	*−44*	−41	−40.52	−39.6	−27.33	−23.6	−20.2	−21.07
	*−90*.*31*	−87.22	−70.89	−89.49	−49.7	−40.69	−59.03	−33.95
2QMG	**T72**, **Q73**, **G230**, **N233**, R235, K321, **S325**.	*G34*, *Q73*, *Y198*, *D228* *G230*, *T231*, *T232*.	D32, **G34**, **T72**, **Q73**, **D228**, T231, **T232**.	**G34**, **Q73**, **Y198**, **D228**, **G230**, **T231**, **T232**.	Q73, Y198, T232.	G34, T72, **D228**, T232.	S36, Q73, **D228**.	**D32**, T72, Q73, **T232**.
	−37.61	*−47*.*9*	−44	−52.8	−23.96	−24.37	=−30	−22.8
	−65.82	*−115*.*8*	−111.4	−97.4	−59.63	−56.45	−80.95	−37.12
3CIC	**G34**, P70, **T72**, Q73, **Y198**, **G230**, **T232**.	D32, T72, **D228**, **G230**, **T232**, N233.	*G34*, *T72*, *Q73*, *D228*, *G230*, *T232*.	**G34**, T72, **Q73**, D228, **G230**, **T232**.	P70, R128, Y198, R235, S328.	T72, **D228**.	T72, Q73, R128, **D228**.	**D32**, T72, Q73, D228, **T232**.
	−37.57	−38.25	*−41*.*51*	−41.77	−26.22	−23.81	−32.18	−22.09
	−92.1	−91.2	−99.27	−100.9	−42.48	−59.72	−73.95	−23.58
3LPK	**T72**, R128, R235, Q326, T329.	D32, **Q73**, **D228**, **G230**, **T232**.	**G34**, **Q73**, **D228**, **G230**, T231, **T232**.	*G34*, *Q73*, *Y198*, *D228*, *G230*, *T231*, *T232*.	**D32**, T72, **D228**, R235.	G34, T72, **D228**, T232.	S36, T72, Q73, **D228**.	**D32**, T72, Q73, **T232**.
	−33.64	−42.18	−44.35	*−44*.*98*	−26.69	−26.69	−32.71	−26.78
	−52.68	−110.1	−103.8	*−118*.*9*	−72.79	−66.55	−71.73	−46.95
4H3G	**T72**, Q73, Y198, R235, **G230**, **T232**, **N233**.	D32, **D228**, **G230**, **T232**, N233.	D32, **D228**, **G230**, **T232**, N233.	D32, **G34**, **Y198**, **D228**, **G230**, **T232**.	*D32*, *W76*, *D228*.	**D32**, **D228**.	**D32**, **D228**.	I126, Y198.
	−36.38	−34.94	−29.16	−36.3	*−26*.*31*	−26.66	−26.99	−21.27
	−60.31	−79.57	−66.56	−89.19	*−84*.*06*	−68.44	−62.24	−48.83
4DJX	P70, **T72**, **Y198**, R235, Q326, S328, T329.	D32, **G34**, R128, **D228**.	D32, **G34**, **D228**, **G230**, **T232**.	T72, **Q73**, N233, R235, S328, T329.	G34, Q73, **D228**.	*D32*, *D228*.	**D32**, **D228**, T232.	**D32**, D228, **T232**.
	−36.3	−41.9	−33.02	−36.79	−22.5	*−22*.*73*	−23.28	−20.85
	−65.28	−75.61	−105.4	−63.1	−88.46	*−59*.*29*	−72.3	−25.17
4HA5	S10, G11, **T232**, **N233**, R307, P308.	D32, **G34**, **Y198**, **D228**, T232.	D32, **G34**, S36, **D228**.	Y71, T72, **Q73**, **Y198**, R235, T329.	**D32**, Y71, **D228**.	**D32**, **D228**.	***D32***, ***W115***, ***D228***.	**D32**, D228.
	−36.84	−38.43	−34	−34.27	−27.03	−25.89	*−22*.*65*	−20.25
	−58.4	−90.8	−78.69	−64.77	−88.46	−54.4	*−61*.*16*	−20.72
4FS4	**T72**, Q73, R235, S328, T329.	**Q73**, K107, R235.	D32, **D228**, **G230**, **T232**.	**G34**, **Y198**, **D228**, **G230**, **T232**.	**D32**, Y198, **D228**.	**D32**, **D228**.	***D32***, ***D228***.	**D32**, **G34**, **T232**.
	−40.4	−28.57	−33.42	−39.56	−25.77	−23.76	*−21*.*14*	−22
	−55.09	−58.95	−72.79	−84.79	−86.42	−60.97	*−67*.*32*	−25.95

^§^The rows represent the 8 receptors used in cross-docking and the columns indicate the 8 ligands used therein. For each receptor, the H-bonding residues, Match score and MM-GBSA energy are indicated in the first, second and third row respectively. The diagonal cells correspond to re-docked data, entries of which have been italicized. The bold-faced residues indicate interacting residues similar to the cognate pair.

**Table 3:** Statistical details of the linear correlation models obtained by cross-docking studies.

PDB ID	R^2^, F-test and p-value of the model obtained from Parameter1	R^2^, F-test and p-value of the model obtained from Parameter2	R^2^, F-test and p-value of the model obtained from Parameter3
4H3G	Not done	Not done	R^2^ = 0.943 F-test = 55.11 p-value = 8.89e-10
4DJX	R^2^ = 0.685 F-test = 17.326 p-value = 4.463e-08	R^2^ = 0.765762 F-test = 26.05 p-value = 9.238e-09	R^2^ = 0.783 F-test = 21.573 p-value = 3.275e-08
4HA5	R^2^ = 0.869 F-test = 26.429 p-value = 2.15e-09	Not done	R^2^ = 0.9 F-test = 27.846 p-value = 2.059e-09
4FS4	R^2^ = 0.51 F-test = 8.557 p-value = 2.067e-7	R^2^ = 0.69 F-test = 17.964 p-value = 7.796e-9	R^2^ = 0.55 F-test = 9.79 p-value = 1.625e-7
3CIC	R^2^ = 0.28 F-test = 0.45 p-value = 2.065e-5	R^2^ = 0.58 F-test = 8.449 p-value = 8.146e-8	R^2^ = 0.66 F-test = 10.4 p-value = 8.66e-7
3LPK	R^2^ = 0.503 F-test = 4.679 p-value = 1.669e-6	R^2^ = 0.593 F-test = 4.460 p-value = 4.873e-7	R^2^ = 0.619 F-test = 3.12 p-value = 1.629e-6
2P4J	Not done	Not done	R^2^ = 0.85 F-test = 32.08 p-value = 1.512e-8
2QMG	R^2^ = 0.746 F-test = 11.13 p-value = 1.06e-6	R^2^ = 0.64 F-test = 5.389 p-value = 1.07e-5	R^2^ = 0.654 F-test = 12.69 p-value = 6.52e-7

### Correlation of Match score with experimental activity

**Figure 4:**
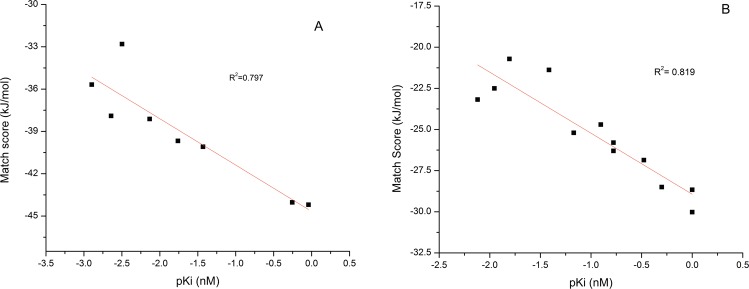
Plot of pK_i_ and Match score for known ligands of (**A**) 2P4J (**B**) 4H3G.

Before using the two structures (2P4J and 4H3G) for virtual screening of compounds using docking study, the correlation between Match score and binding affinity was validated. Therefore optimized structures obtained for 8 ligands of 2P4J (compound no-22, 5a, 5b, 5c, 5d, 5e, 5f and 5g as in Ghosh *et al*.^46^) and 13 ligands of 4H3G (compound no-26, 27, 28, 31, 32, 33, 34, 36, 37, 38, 39, 40, 41 as in Mandal *et al*.^47^) were docked to the respective receptors and the Match score was noted down. pK_i_ was calculated from the K_i_ values reported by Ghosh *et al*. for 2P4J and Mandal *et al*. for 4H3G. The pK_i_ versus Match score plot for both (Fig. 4A,B) indicate good correlation. The K_i_, Match score, MM-GBSA energy and receptor ligand interactions of all are tabulated in Tables S5 and S6.

### Virtual screening

A library of 12000 compounds was simultaneously docked to the selected semi-open (4H3G) and closed (2P4J) conformation of BACE1. The compounds were thus declared hits if (i) they showed two out of the four interactions analyzed from re-docking studies and (ii) exhibited a Match score <−29 kJ/mol, a threshold selected from cross-docking studies. 4H3G yielded 30 compounds while 2P4J yielded 57 compounds, out of which 23 compounds were found common (Table S7).

### MM-GBSA calculations of the screened hits

**Figure 5:**
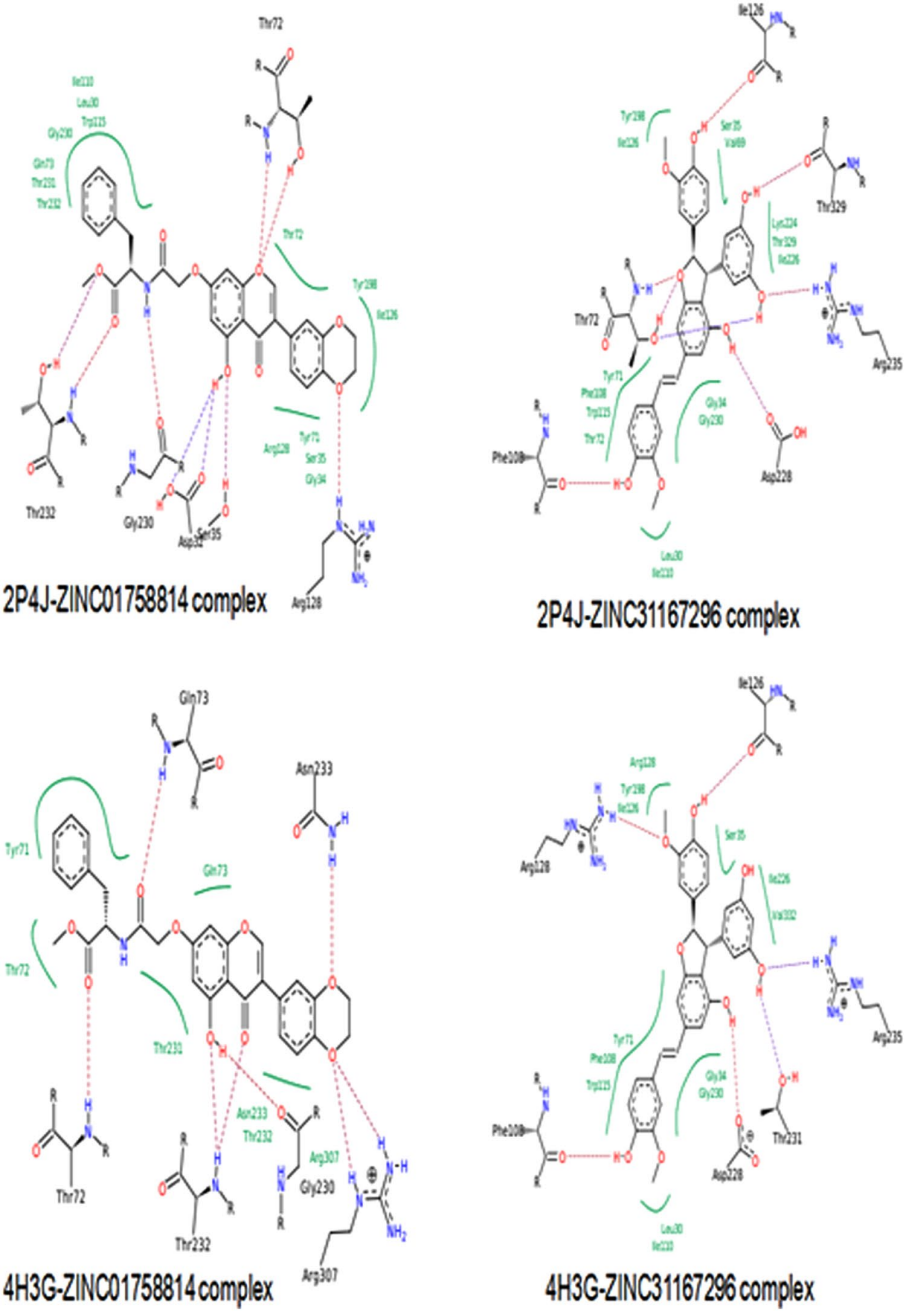
Interactions exhibited by the ligands (ZINC31167296 and ZINC01758814) with lowest binding energies. The interaction map has been generated by FlexX.

Binding energies were calculated for the complexes with the screened compounds (Table S7) and also the known BACE1 inhibitors (Table 2) to assess the stability of the docked complexes. The ∆G values varied from −100.92 kcal/mol to −21.93 kcal/mol for the complexes with closed BACE1 structure. In case of the complexes with semi-open BACE1 structures the ∆G values varied from −89.8 kcal/mol to −39.69 kcal/mol. Interestingly ZINC31167296 and ZINC01758814 were the top two compounds implying high affinity for both the conformations (bold-faced in Table S7). As seen in Fig. 5, ZINC01758814 forms H-bond with T72, T232 and G230 for both the forms and so does ZINC31167296 with I126, F108, D228 and R235.

### Arranging the common compounds with the pharmacophore models

**Figure 6:**
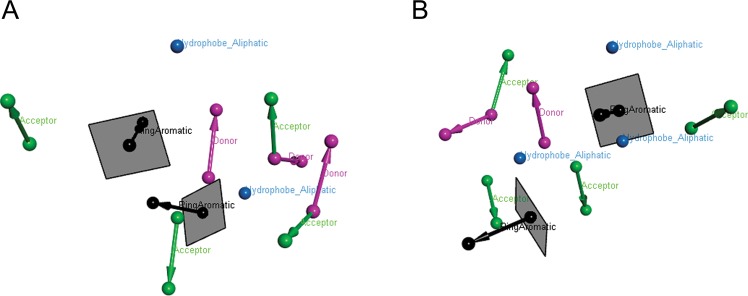
Pharmacophore models obtained from PharmaGist. (**A**) Model 1- denotes the model generated from 12 BACE1 inhibitors (with IC_50_) and (**B**) Model 2- denotes the model generated from 9 BACE1 inhibitors (with K_i_).

Two pharmacophore models (Model 1 and Model 2) were generated using known active inhibitors (Supplementary Tables S2 and S8) of BACE1 (Fig. 6). Both Model 1 and Model 2 gave the highest score of alignment with its pivot (Table 4). As evident from the figure, some of the donor and acceptor features overlapped in both the models. As a result the total spatial features of donor and acceptor for each of the two models remained same i.e. 5. Analysis of the corresponding reference pharmacophore for Model 1 and Model 2 indicate that the donors and acceptors are involved in H-bond interactions with active site residues of BACE1 such as D32, T72 Q73, G230, T232 and N233. Additionally the presence of aromatic feature involved in the stacking interactions with Y71 has been observed in both the models. The two models were validated by overlaying to their respective reference pharmacophore obtained by superposition of the X-ray crystal structures of the input molecules (Table 5). The generated models were found to be similar to their reference pharmacophore.

**Table 4:** The two pharmacophore models obtained from PharmaGist using active inhibitors of BACE1.

Pharmacophore	Score	No of spatial features	No. of aromatic feature	No of Hydrophobic features	No. of donor features	No. of acceptor features	No of positives	No of negatives
Model 1	41	9	2	2	3	4	0	0
Model 2	35.3	10	2	3	2	4	0	0

**Table 5:** Overlay score between the pharmacophore model generated by PharmaGist and reference pharmacophore.

Pharmacophore model	Score
Model 1	0.7154
Model 2	0.6022

The 23 common compounds were arranged in descending order according to the pairwise alignment score obtained by aligning each compound with the two pharmacophore models generated. For the pairwise alignment, the default criterion of a minimum match of 3 features between the model and the common compound was set. The arrangement of the common compounds selected through virtual screening was assessed using the enrichment factor. The maximum value of enrichment factor that could be attained using the top 10 and 15 compounds were 26 and 17.34 respectively. We found that the pharmacophore models [Model 1 (using 12 inhibitors with IC_50_ values) and Model 2 (using 9 inhibitors with K_i_ values)] showed comparable performance (Table 6) and therefore both were used for arranging the compounds.

**Table 6:** Performance of the pharmacophore models.

PharmaGist models	Enrichment factor in top 10	Enrichment factor in top 15
Model 1	10.52	12.28
Model 2	13.157	13.157

### Activity prediction of BACE1 inhibitors as multi-target agents

AD being a multi-factorial neurodegenerative disease, a promising designing approach should be one that would target more than one molecule responsible for the pathology. It is to be noted that the disease is characterized by elevated levels of amyloid β in the brain as an underlying pathogenesis of the disease. Therefore, 23 virtually screened and arranged BACE1 inhibitors were predicted for anti-amyloidogenic activity to finally declare them as predicted BACE1 inhibitors with dual potency.

### Antiamyloidogenic activity prediction of BACE1 inhibitors

All of the 23 screened compounds lay within the applicability domain of the in-house developed QSAR model^61^ that was used to predict the anti-amyloidogenic activity (logIC_50_). Using the predicted IC_50_ values, a list of the 23 compounds was prepared. IC_50_ of the compounds have been calculated considering the ligand-based descriptors like hydrogen bonding surface area, average information content reflecting the shape and symmetry, the relative negative charged surface area and vibrational energy of the atoms as in Eqn. 1 of Methods section. An important descriptor that might have contributed significantly to its antiamyloidogenic activity is hydrogen bonding surface area (HBSA) which was high for ZINC72319974, ZINC03175470, ZINC31167296, ZINC16027834 and ZINC01758814 signifying less ability to form hydrogen bonds with bulk solvent which in turn would increase the anti-amyloidogenic activity. The molecules thus bind to the hydrophobic region of the fibril exhibiting anti-amyloidogenic activity. Additionally ZINC01758814 showed highest value of vibrational entropy which elucidates flexibility of the molecule and the associated conformational and rotational degrees of freedom.

In order to identify compounds with dual potency a shortened list was prepared using a criterion of PharmaGist score greater than 20 and predicted antiamyloidogenicity (IC_50_) less than 550 μM. Therefore, ZINC31167296, ZINC16027834, ZINC01758814, ZINC02119155 and ZINC53276039 may be considered as compounds with dual potency to be validated using *in vitro* studies.

### *In vitro* validation

#### Probing the BACE1 inhibition ability of the screened compounds

Four (bold-faced) out of the five screened compounds enlisted in Table 7 are commercially available. Therefore four compounds were procured for validation. All of them showed moderate BACE1 inhibition ability at a concentration of 1 µM (Table 8) with the SPI_1μΜ_ (single point inhibition percentage) of the compounds varying across the range of 16.98 to 21.6. Interestingly, SPI_1μΜ_ of myricetin (20.6), a known BACE1 inhibitor falls within this range. SPI_1μΜ_ of gossypetin, another flavonoid, was found to be low with a value of 4.6.

**Table 7:** Common compounds predicted to be potent BACE1 inhibitor as well as potent antiamyloidogenic agent.

Compounds	Selection criterion for		
	BACE1 inhibitor based on pharmacophore model 1	BACE1 inhibitor based on pharmacophore model 2	Antiamyloidogenic compound
*ZINC31167296*	Score >20	Score >20	Predicted IC_50_ < 550 μM
***ZINC16027834***			
***ZINC01758814***			
***ZINC02119155***			
***ZINC53276039***

**Table 8:** BACE1 inhibition ability.

Compound	SPI_1 μΜ_
Known inhibitor (Myricetin)	20.6
ZINC01758814	16.98
ZINC16027834	20.7
ZINC02119155	19.62
ZINC53276039	21.6
Weak inhibitor (Gossypetin)	4.6

#### Probing the antiamyloidogenicity of the screened compounds

**Figure 7:**
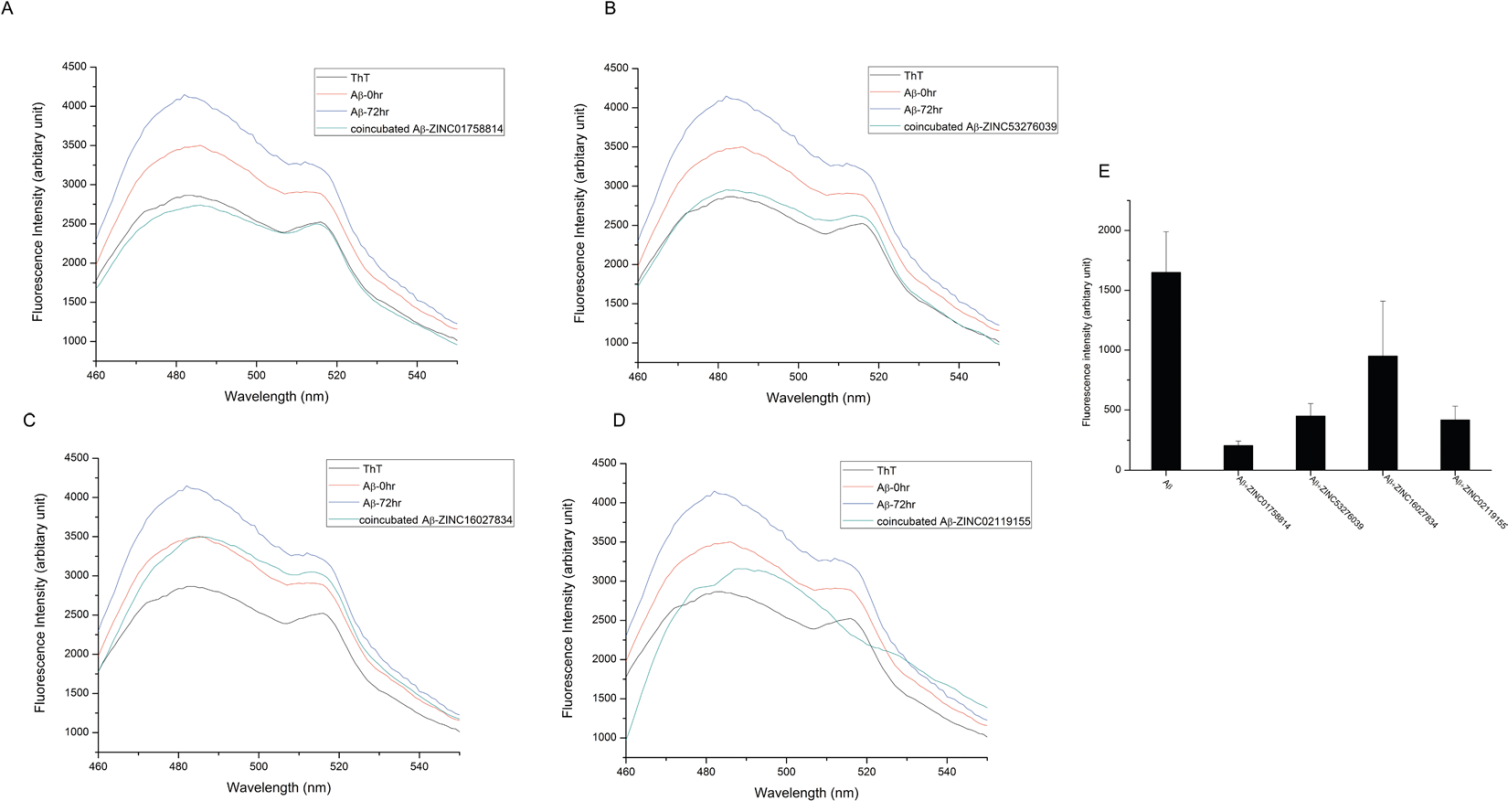
Thioflavin T assay for compounds (**A**) ZINC01758814 (**B**) ZINC53276039 (**C**) ZINC16027834 (**D**) ZINC02119155 (**E**) Comparison of ThT assay for Aβ_25–35_ aggregation for all four compounds.

On adding Thioflavin T (ThT) to the 3-day aged Aβ_25–35_ samples the ThT fluorescence was enhanced due to restricted rotation of the ThT rings in the hydrophobic environment of the amyloid fibrils. Interestingly, all the four compounds procured, showed a reduction in fluorescence when co-incubated with Aβ_25–35_ for 3 days. Our results (Fig. 7) indicate that the compounds show an ability to interfere with the formation of the amyloid fibrils thus reflecting a reduction in the degree of fibril aggregates. One of the compounds, ZINC01758814 showed highest anti-amyloidogenicity with more than 50% reduction in the fibril formation.

## Conclusions

Current treatment of AD is limited to temporary symptomatic support to the cognitive abilities of the patient and is therefore not curative. Since Aβ is believed to play an early role which is crucial in the etiology of AD, therapeutics targeting production of Aβ or its aggregation would lead to more effective disease-modifying drugs. Scientists are working hard to discover/design inhibitor molecules for the protease BACE1 that is known to cleave APP. Although further cleavage of APP by γ-secretase produces Aβ, cleavage by BACE1 forms the rate limiting step. Development of potent BACE1 inhibitors is still being persuaded with much less success than expected. One of the reasons for the lack of success is probably the flexibility of BACE1. The enzyme is known to undergo a conformational transition from open to closed through a half open half closed (HOHC) conformation^68^. Thus, for such an enzyme, use of a static crystal structure during docking would fail to accommodate diverse range of ligand conformations. On the contrary, taking into account receptor flexibility considering multiple crystal structures with slight movement of amino acid residues, loops or turns in different directions gives the ligand a better chance to accommodate itself in the active site of the enzyme. The other reason for the low success rate is the multi-factorial aspect of the disease. For a compound to be used as a therapeutic agent for AD it is thus not enough to target a single molecule. Rather a compound acting on more than one target would fit better.

In the present study, we have considered multiple crystal structures for closed and semi-open conformations of BACE1 with bound ligands of varied size and shape in order to account for the flexibility of the enzyme. By re-docking and cross-docking we could select two most suitable and diverged representative structures from each group, that could adopt ligands of varied size. The closed and semi-open conformations were subsequently used to virtually screen the natural product database of ZINC and from the two lists of screened compounds, 23 common compounds were selected. The common compounds were believed to outperform the other ligands by their versatility to bind to different conformations of BACE1. The common compounds were further arranged in the descending order of their similarity to known BACE1 inhibitors using two pharmacophore models. For the multi-factorial aspect of the disease, these virtually screened BACE1 inhibitors were further predicted for anti-amyloidogenic activity. Finally, we validated our studies through *in vitro* experimentations of four of the screened compounds. ZINC16027834, ZINC02119155, ZINC01758814 and ZINC53276039 showed moderate BACE1 inhibition activity. Additionally, all the compounds showed the ability to interfere with amyloid fibril formation Hence these compounds might act as potential starting compounds to target BACE1 inhibition as well as antiamyloidogenicity and therefore be used in AD therapeutics. The incorporation of flexibility considering multiple structures for different conformations of BACE1 with subsequent use of ligand-based and structure-based approaches for selection of compounds against dual targets makes this effort different from the ones pursued before. Thus we have successfully developed a bilayered (structure and ligand-based approach), dual-target directed screening methodology imbibing receptor flexibility to identify natural compounds that might serve as scaffolds for leads to be used in therapeutics for AD.

## Supplementary information

Hybrid approach to sieve out natural compounds against dual targets in Alzheimer’s Disease
