# Supplementary material for: Hybrid approach to sieve out natural compounds against dual targets in Alzheimer’s Disease
Source: Sci Rep. 2019 Mar 6;9:3714. doi: 10.1038/s41598-019-40271-9 (PMC6403309; doi:10.1038/s41598-019-40271-9)
Supplement: Supplementary file 1 — Hybrid approach to sieve out natural compounds against dual targets in Alzheimer’s Disease [file 41598_2019_40271_MOESM1_ESM.doc]

**SUPPLEMENTARY MATERIAL**

**Hybrid approach to sieve out natural compounds against dual targets in Alzheimer’s Disease**

Sucharita Das,^a^ Sandipan Chakraborty^b^ and Soumalee Basu^a*^

**^a^** Department of Microbiology, University of Calcutta, 35 Ballygunge Circular Road,

Kolkata - 700 019, India

^b^ Department of Chemical Sciences, Indian Association for the Cultivation of Science, Jadavpur, Kolkata-700032, India

* Corresponding author:

Soumalee Basu: Tel: +91-33-4615445/4711/4712 (Ext. 341/342); Fax: +91-33-24614849; E-mail: [soumalee@gmail.com](mailto:soumalee@gmail.com)


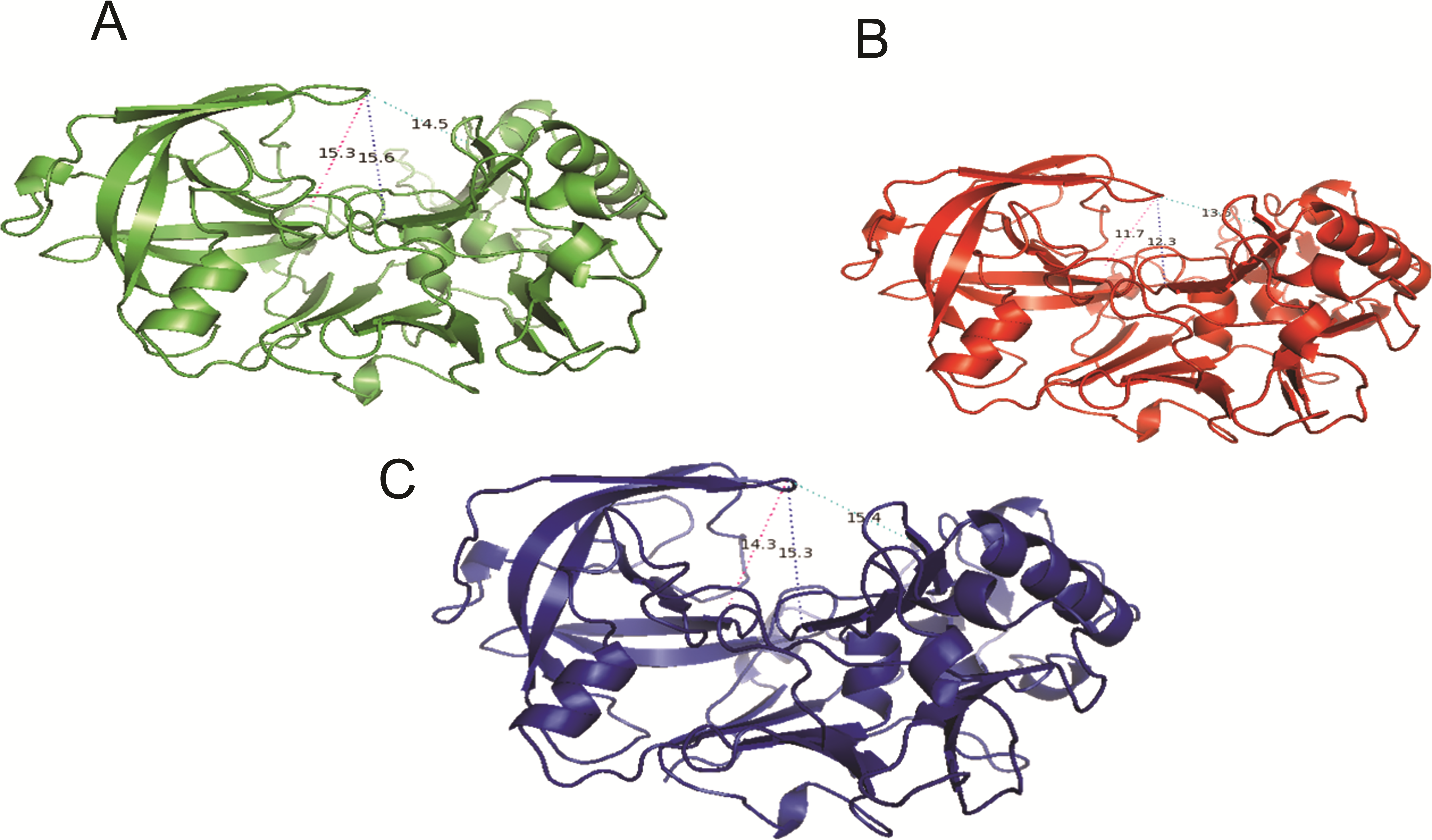


Figure S1 Representative structures from different conformations of BACE1 (A) Open (B) Closed (C) Semi-open conformation showing the difference in d1, d2 and d3. The dotted lines show the distances d1 (magenta) d2 (blue) and d3 (cyan).

Table S1. The distances d1, d2 and d3 for the 10 BACE1-inhibitor complexes.

| PDB | d1 in Å | d2 in Å | d3 in Å | Cavity volume(Å^3^) | Group |
| --- | --- | --- | --- | --- | --- |
| 4DJX | 14.7 | 15.1 | 15.3 | 1081.4 | Group 1 |
| 4HA5 | 14.7 | 15.4 | 15.1 | 1882 |  |
| 4FS4 | 15 | 15.4 | 14.9 | 1672.32 |  |
| 4H3G | 14.3 | 15.3 | 15.4 | 1565.94 |  |
| 4H3F | 14.7 | 15.2 | 14.9 | 1919.02 |  |
| 2G94 | 11.5 | 11.6 | 13.1 | 1218.95 | Group2 |
| 2P4J | 11.8 | 11.6 | 12.8 | 1481.14 |  |
| 2QMG | 12 | 12.2 | 13.7 | 891.35 |  |
| 3CIC | 11.7 | 12.1 | 12.7 | 657.41 |  |
| 3LPK | 12.3 | 12.8 | 13.9 | 702.60 |  |
|  |  |  |  |  |  |

**Table S2.** Details of inhibitors of the 10 complexes used in the study

| LIGANDS | 2D-STRUCTURE | No of rotatable bonds | K_i_  values in nM |
| --- | --- | --- | --- |
| 4H3G_L_ | 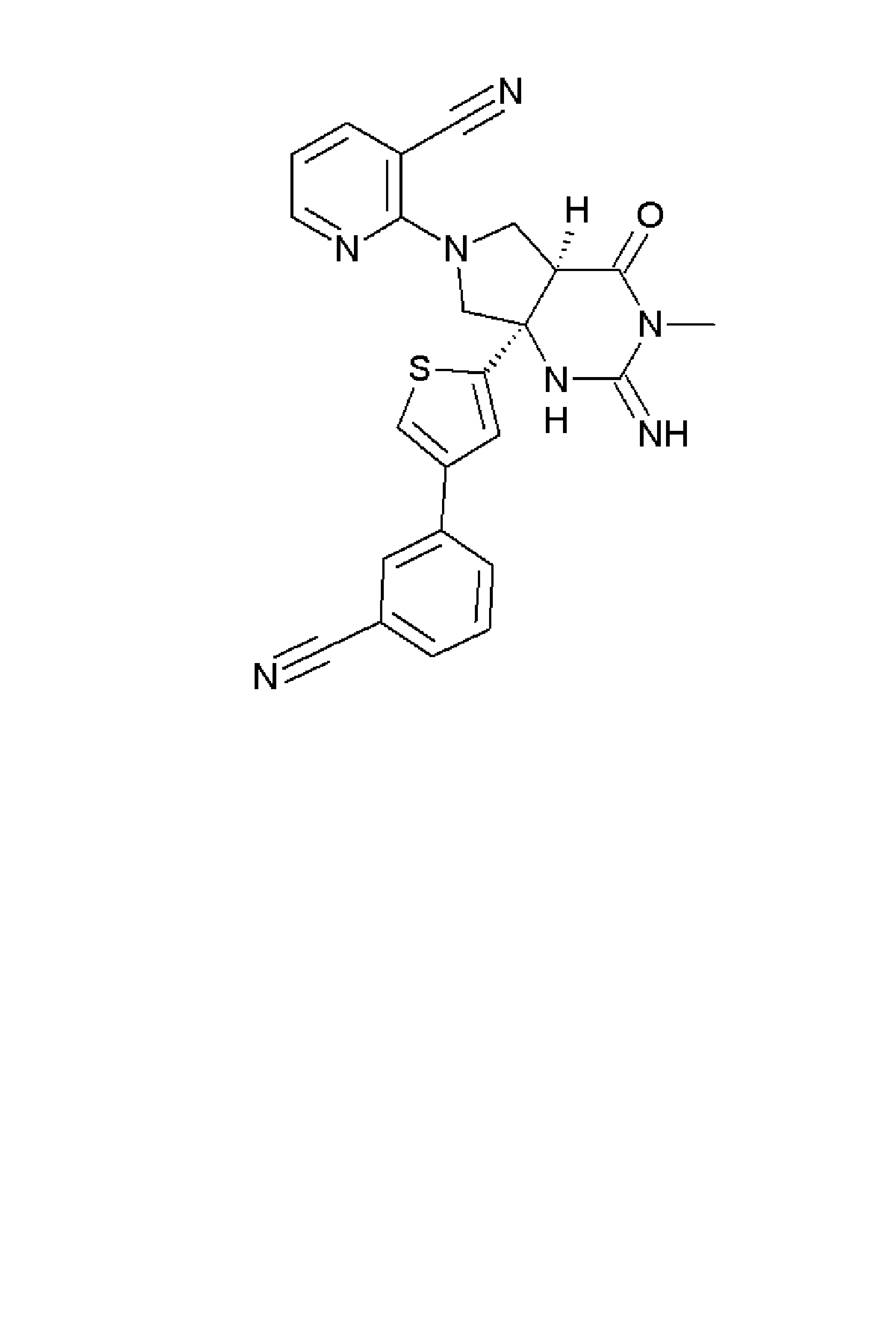 | 3 | 6 |
| 4DJX_L_ | 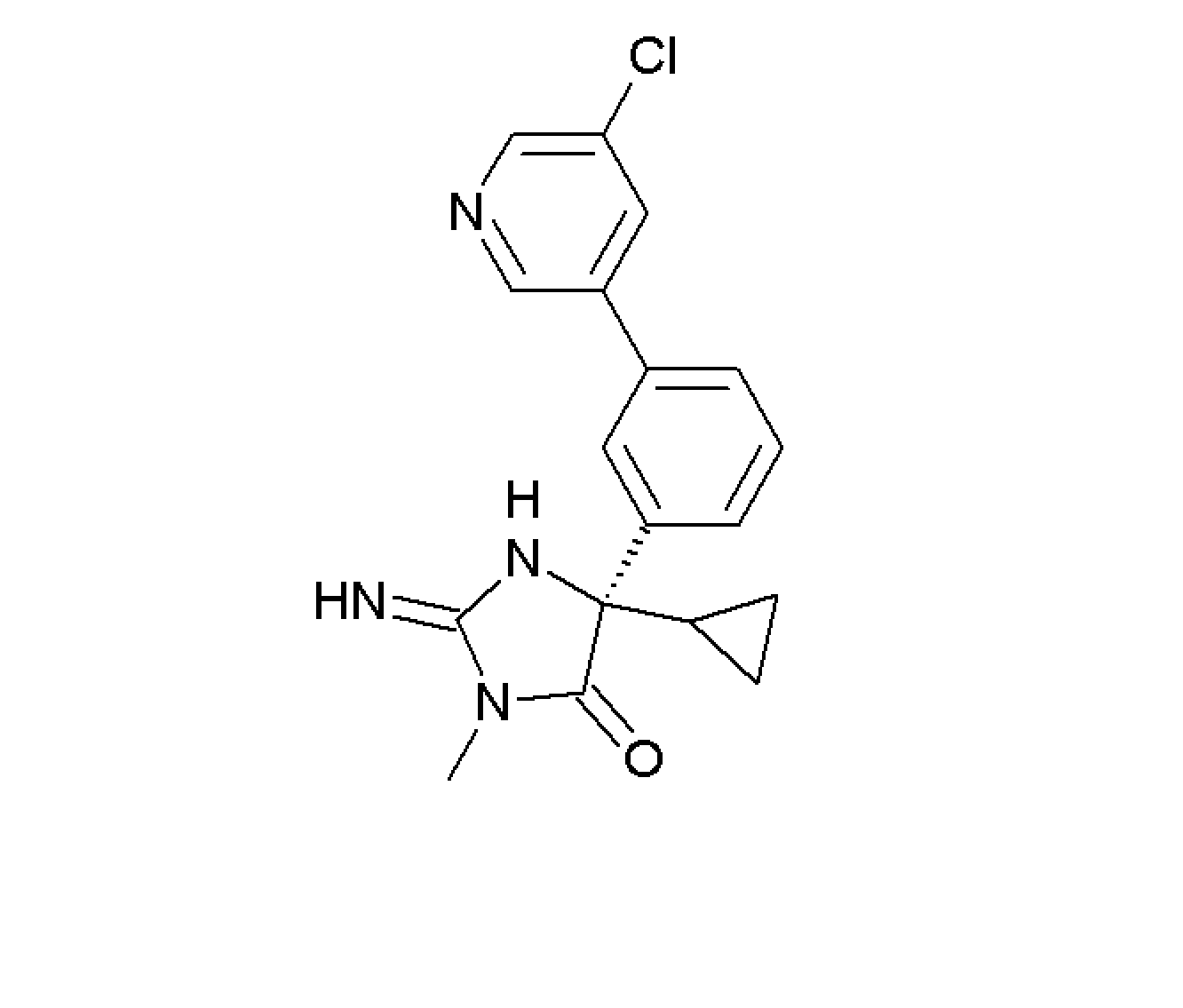 | 3 | 21 |
| 4HA5_L_ | 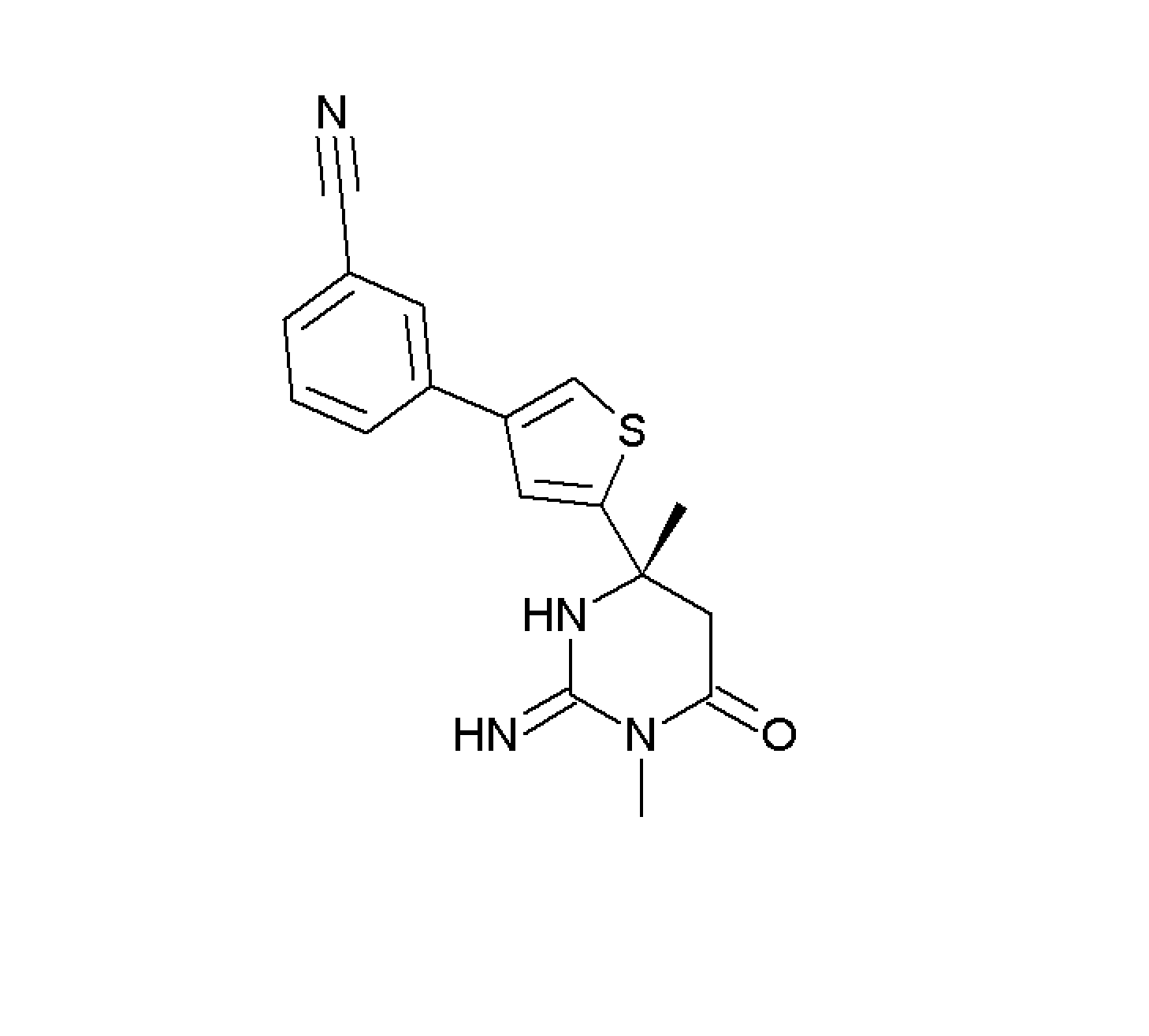 | 2 | 57 |
| 4FS4_L_ | 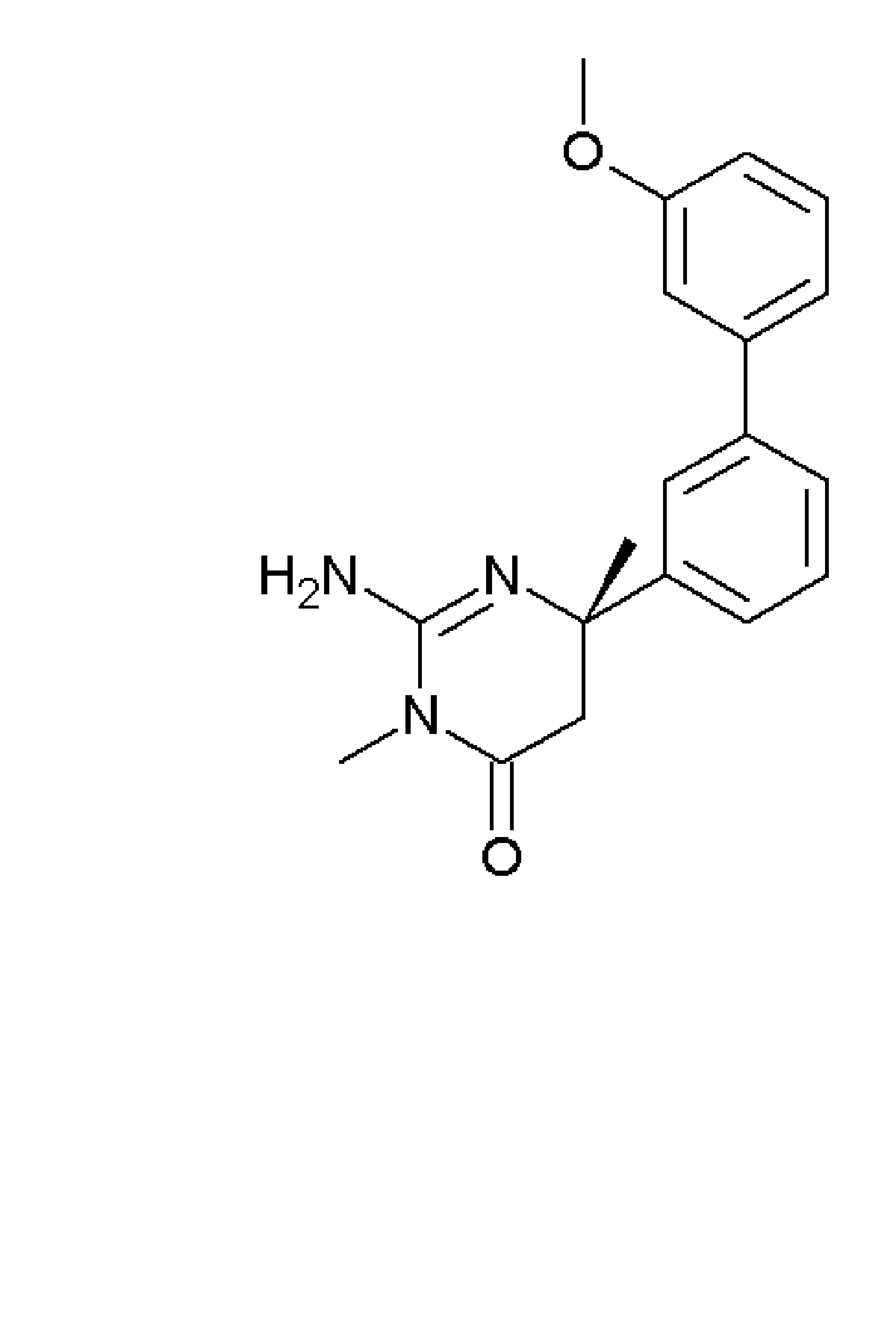 | 4 | 270 |
| 4H3F_L_ | 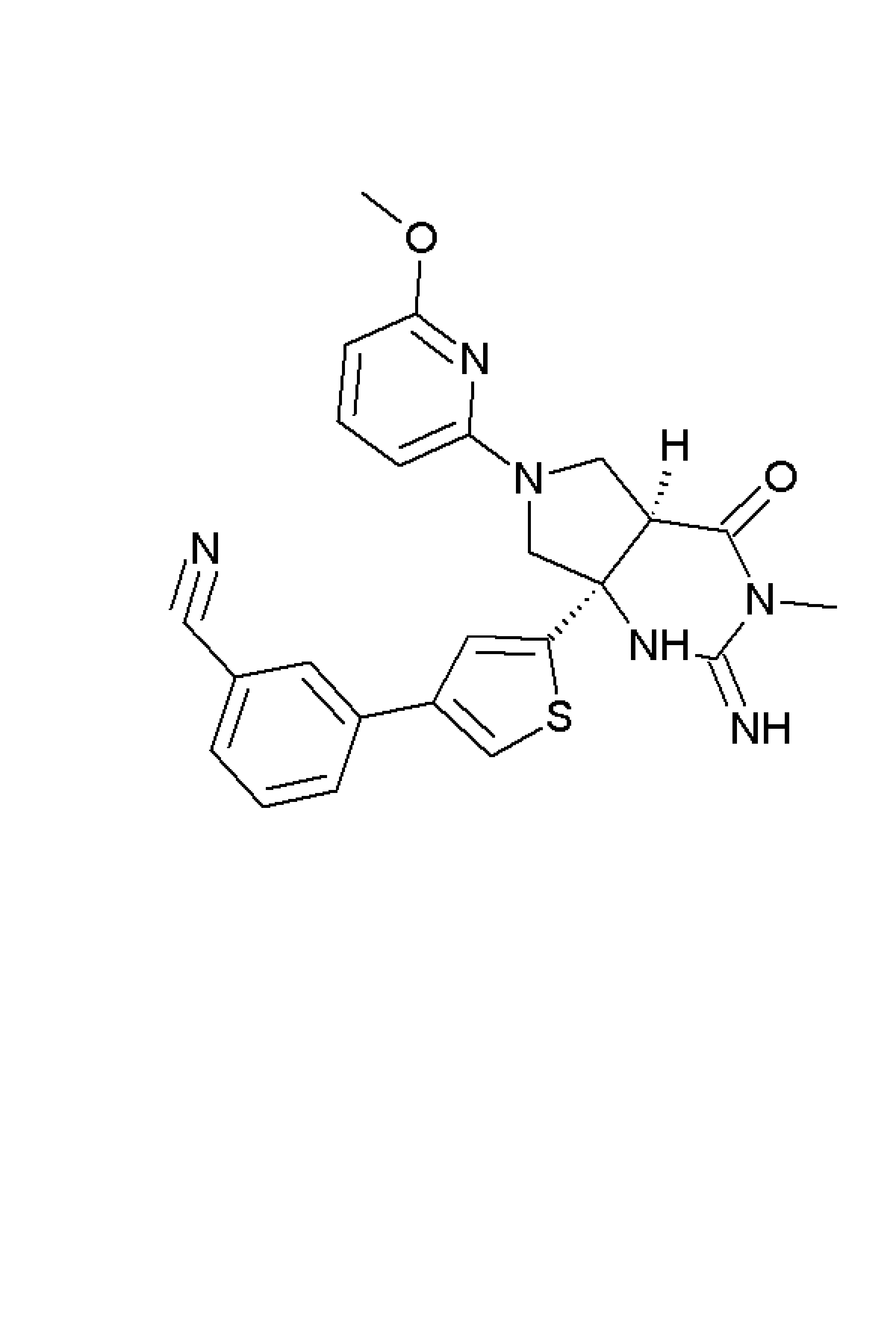 | 4 | 1 |
| 3CIC_L_ | 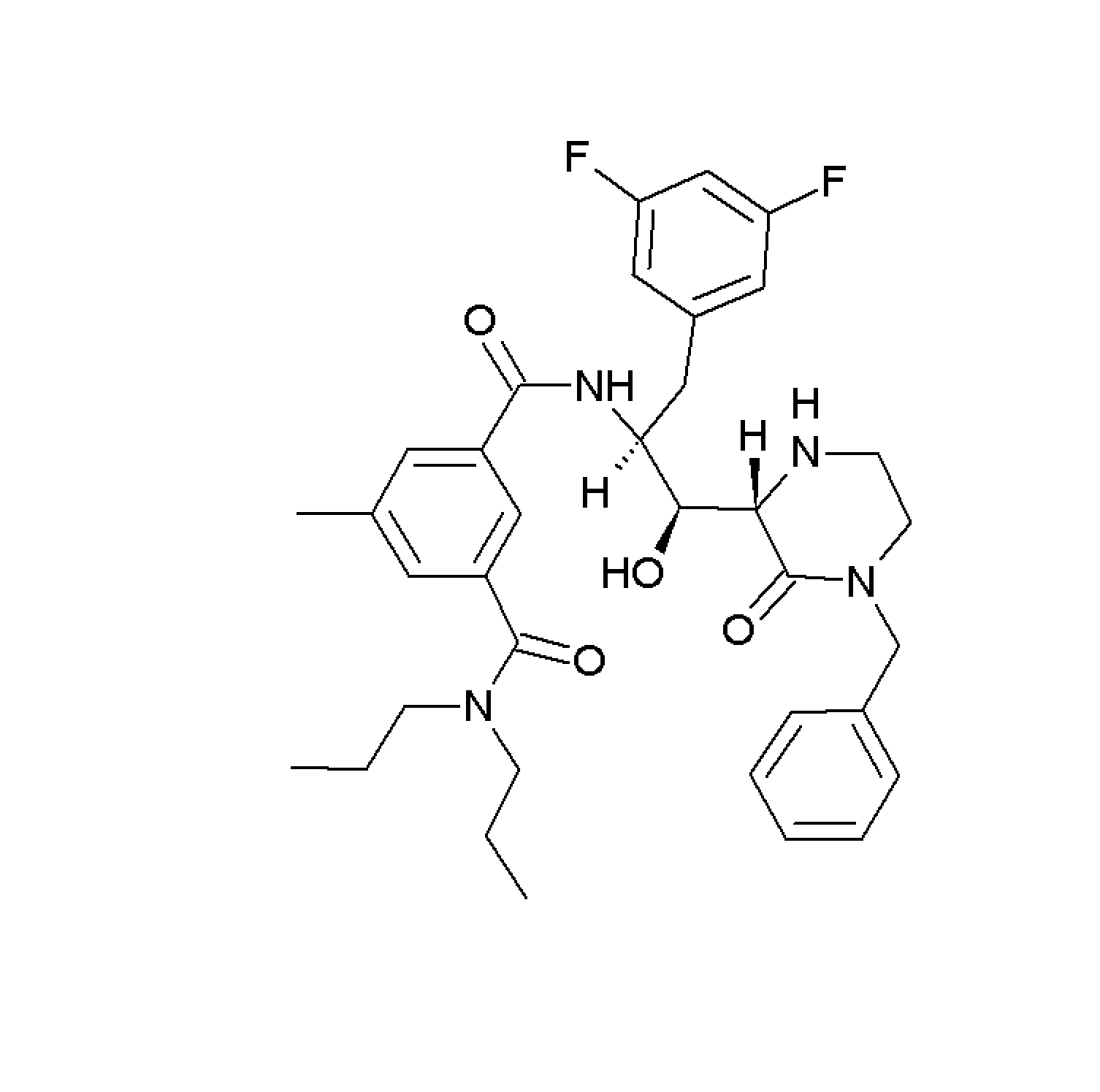 | 16 | 3 |
| 3LPK_L_ | 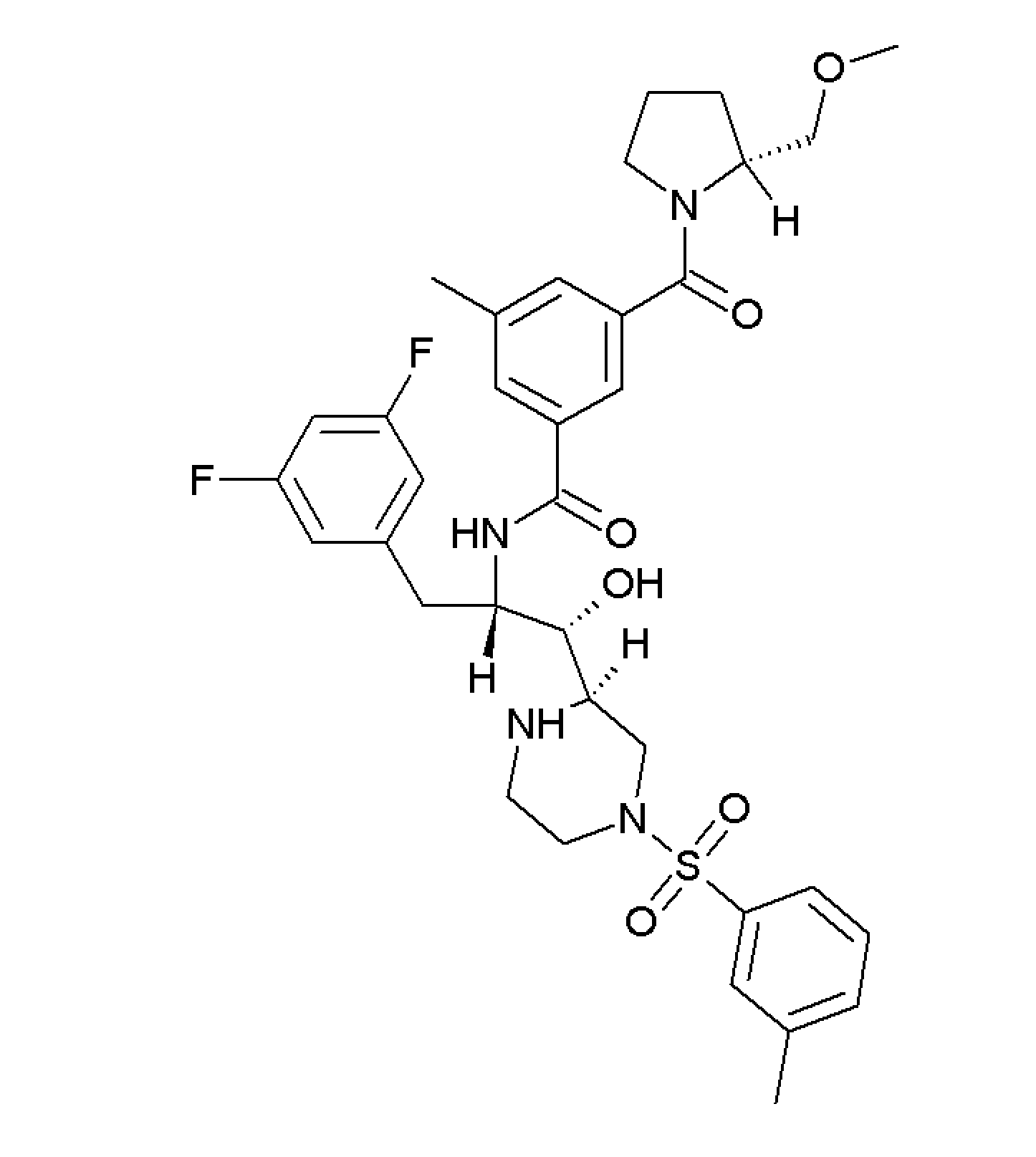 | 14 | 0.8 |
| 2P4J_L_ | 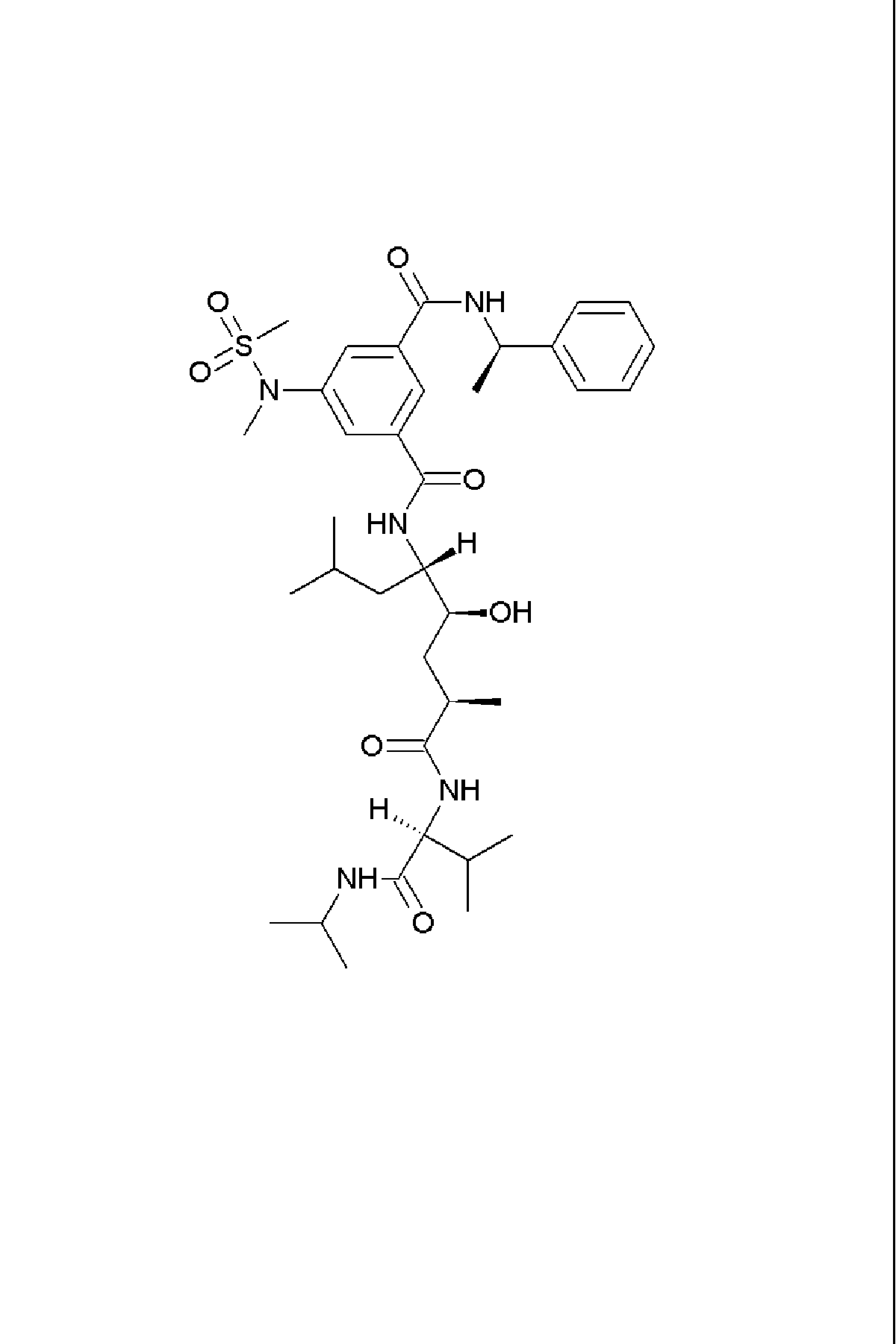 | 22 | 1.1 |
| 2QMG_L_ | 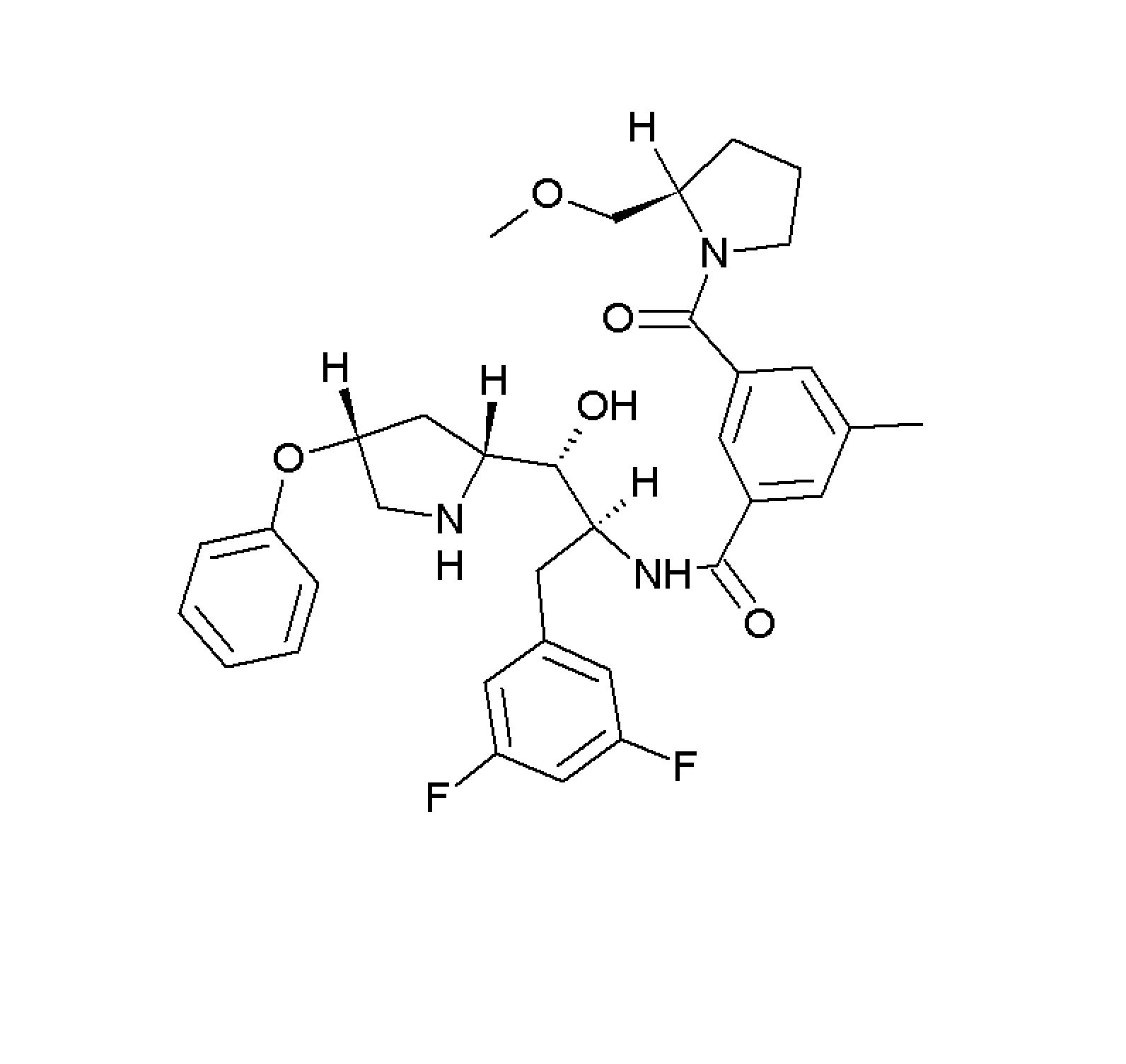 | 14 | 0.7 |
| 2G94_L_ | 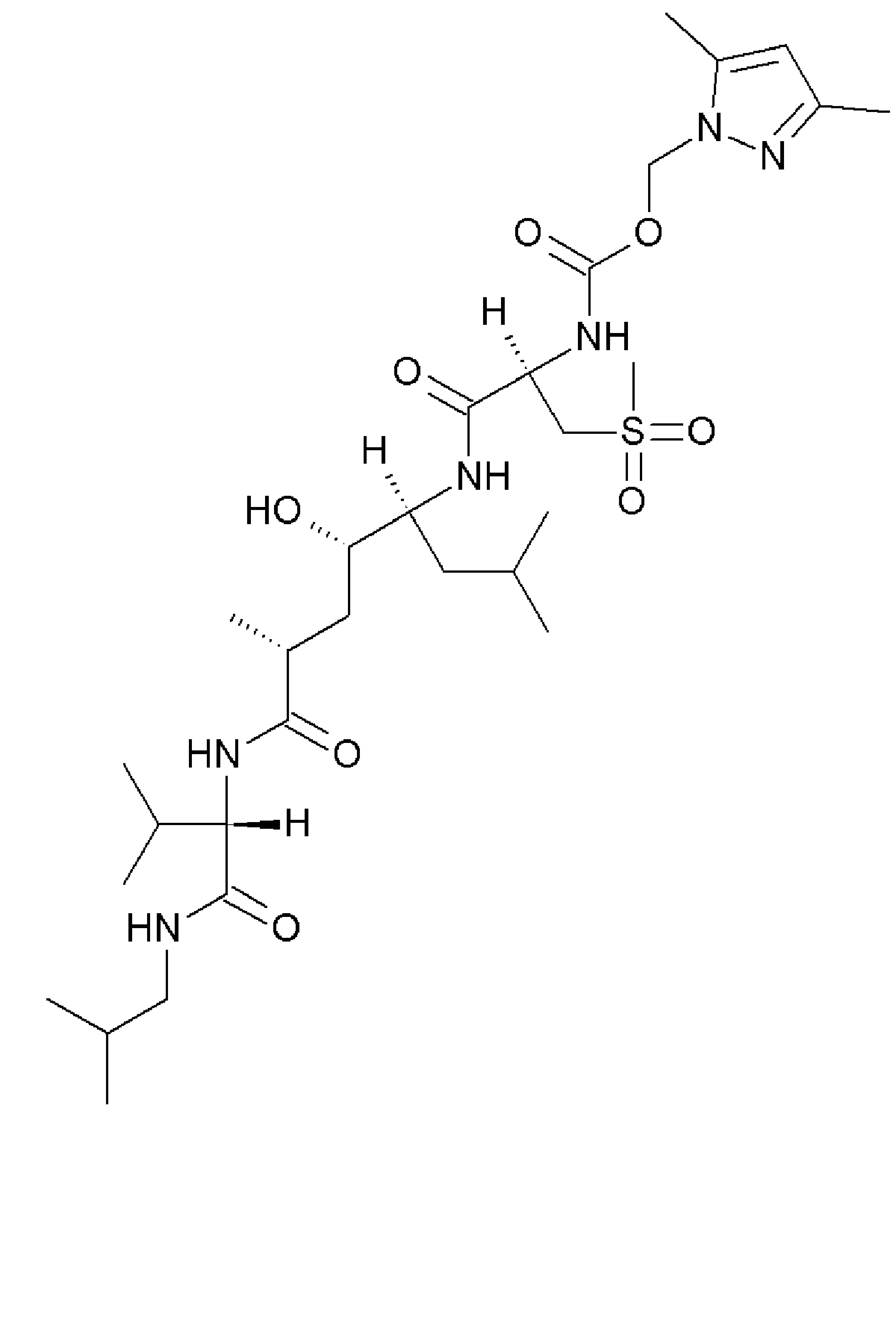 | 24 | 0.3 |

Table S3. RMSD of each ligand after re-docking to the respective BACE1 crystal structure using three parameter sets.

| LIGANDS | RMSD in Å | | |
| --- | --- | --- | --- |
|  | Parameter set 1  max_nsi = 200  max_nsf = 200 | Parameter set 2  max_nsi = 125  max_nsf = 300 | Parameter set 3  max_nsi = 400  max_nsf = 400 |
| 4H3G_L_ | >2.5 | >2.5 | 0.68 |
| 4DJX_L_ | 1.51 | 1.51 | 1.38 |
| 4HA5L | 2.148 | >2.5 | 2.142 |
| 4FS4L | 1.88 | 1.88 | 1.88 |
| 4H3FL | >2.5 | >2.5 | >2.5 |
| 3CIC_L_ | 0.913 | 0.89 | 1.297 |
| 3LPK_L_ | 1.65 | 2.057 | 1.9 |
| 2P4J_L_ | >2.5 | >2.5 | 2.4 |
| 2QMG_L_ | 1.42 | 1.28 | 1.42 |
| 2G94_L_ | >2.5 | >2.5 | >2.5 |

**Table S4**- Cross docking studies of 8 BACE1-inhibitor complexes

| **Receptor -ligand complex** | **Match score** | **MMGBSA**  **ΔG** | **Interactions** |
| --- | --- | --- | --- |
| **Cross-docking studies with 2P4J** | | | |
| 2P4J-23I | -40.37 | -93.091 | 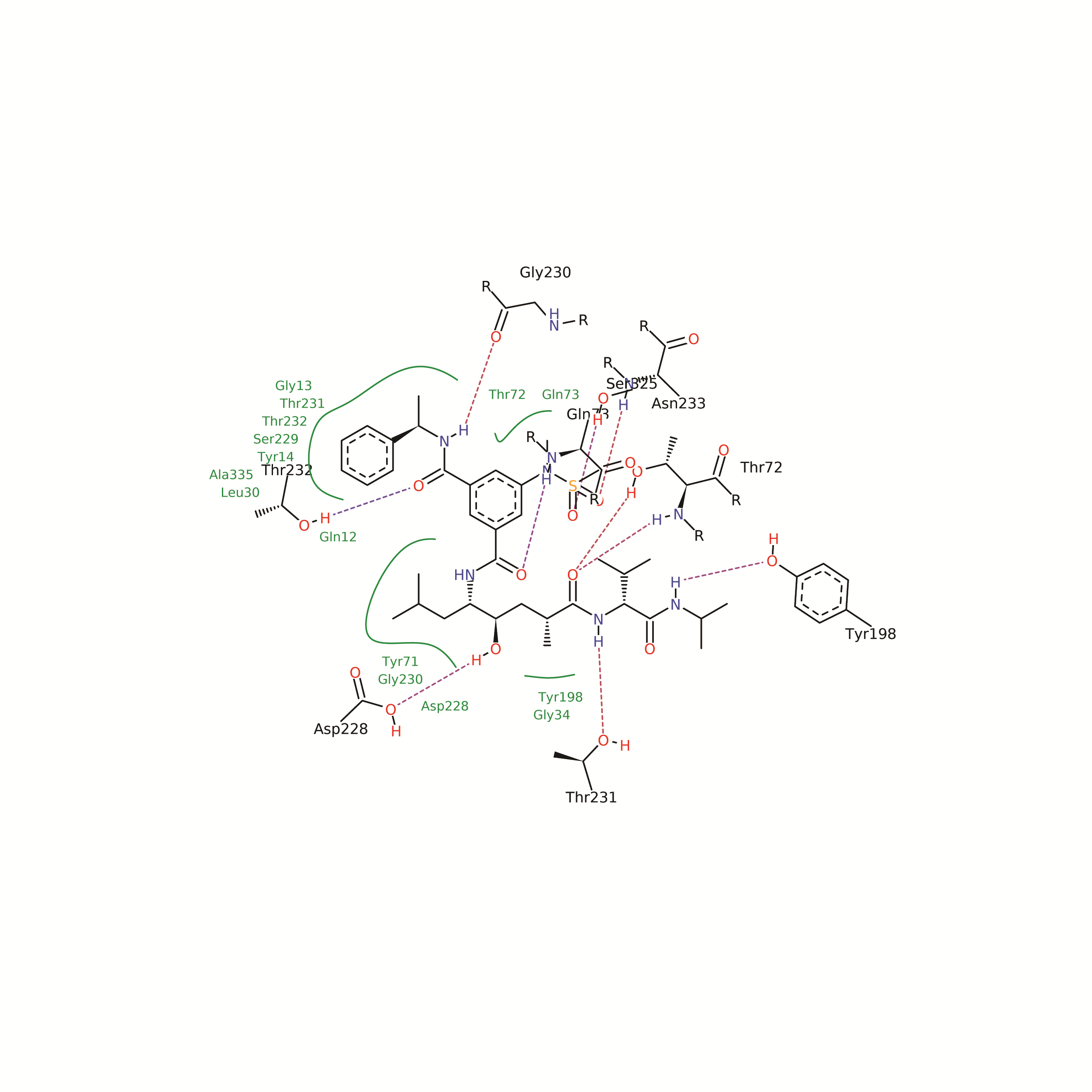 |
| 2P4J-SC6 | -41 | -87.22 | 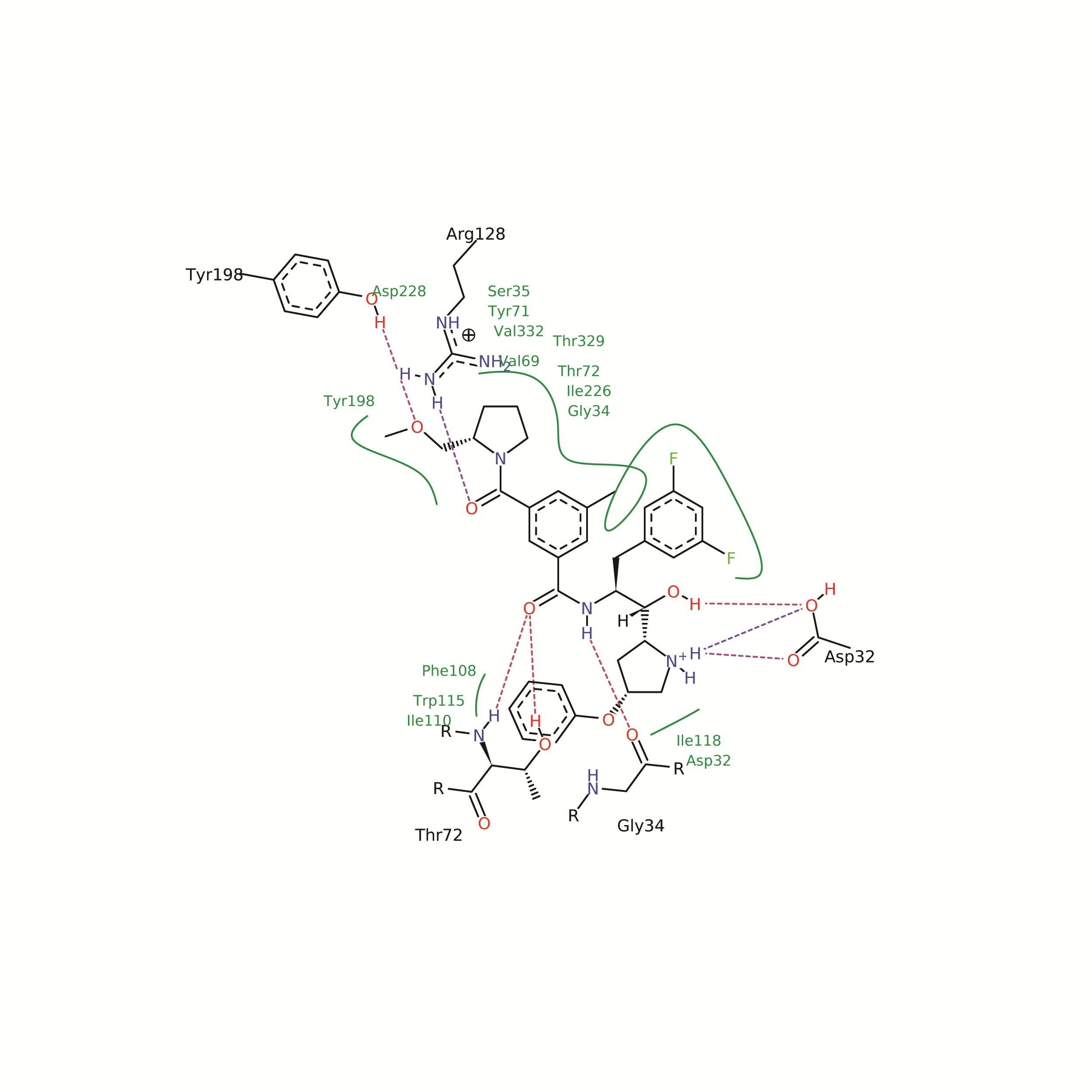 |
| 2P4J-Z76 | -39.66 | -89.495 | 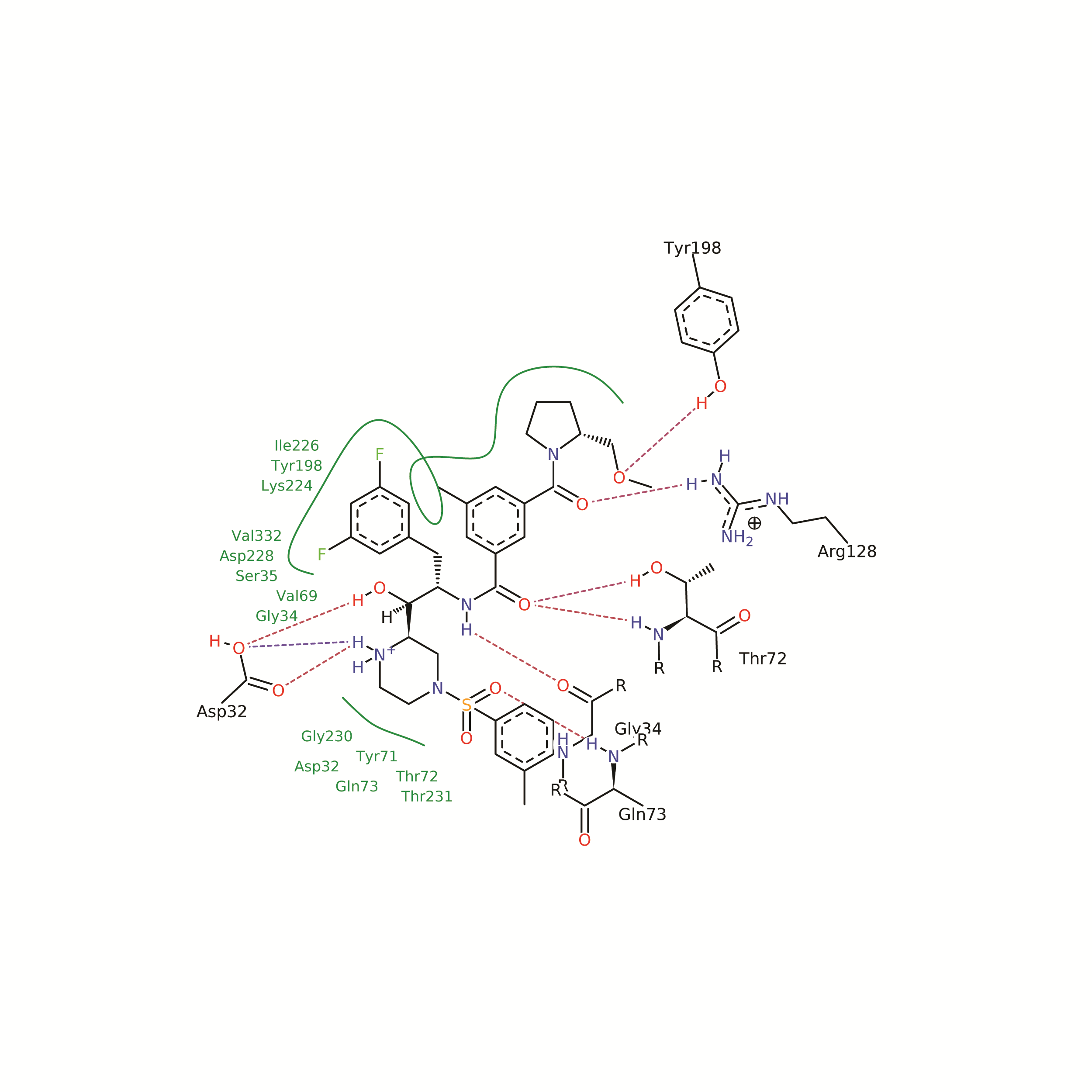 |
| 2P4J-316 | -40.52 | -70.89 | 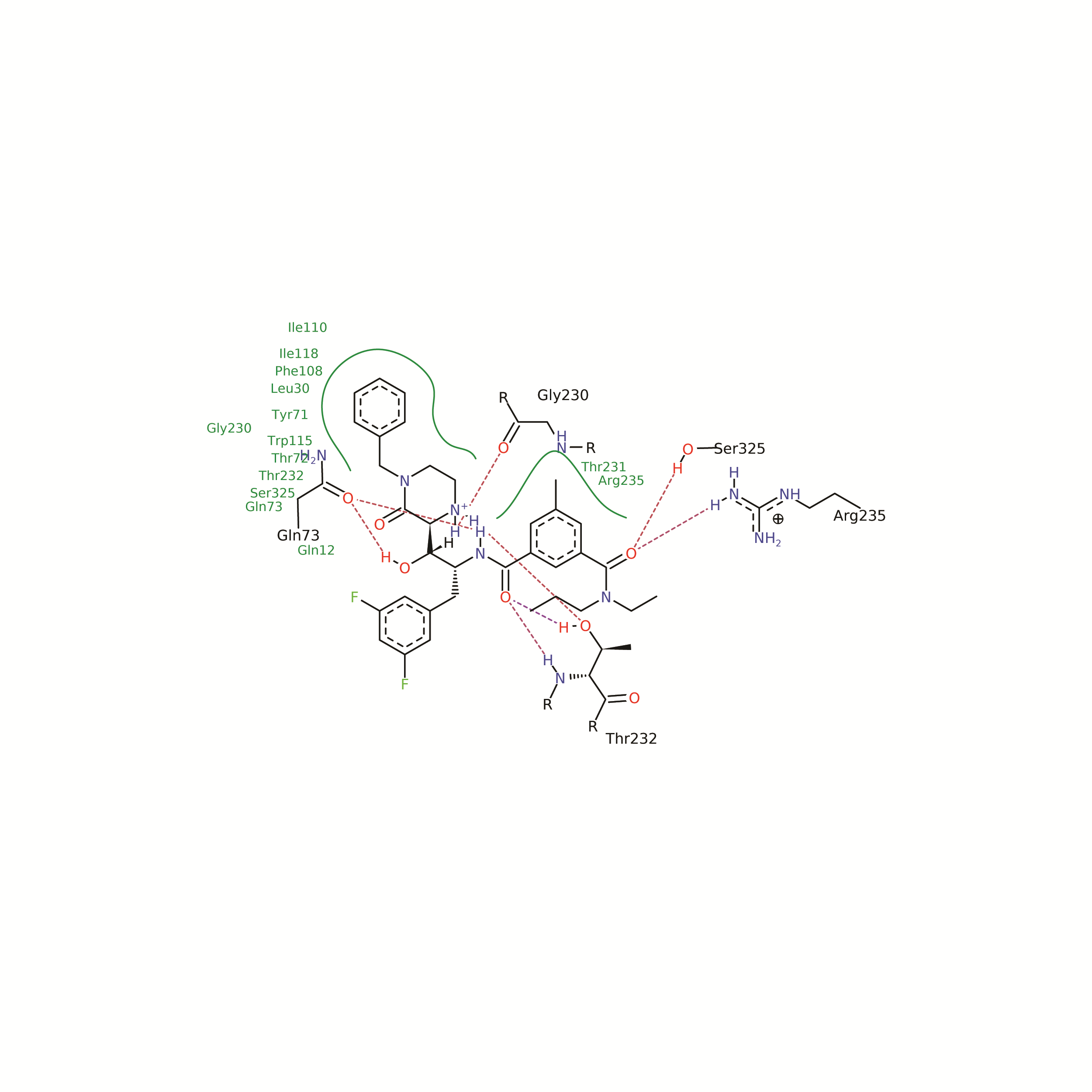 |
| 2P4J-10Q | -27.33 | -49.77 | 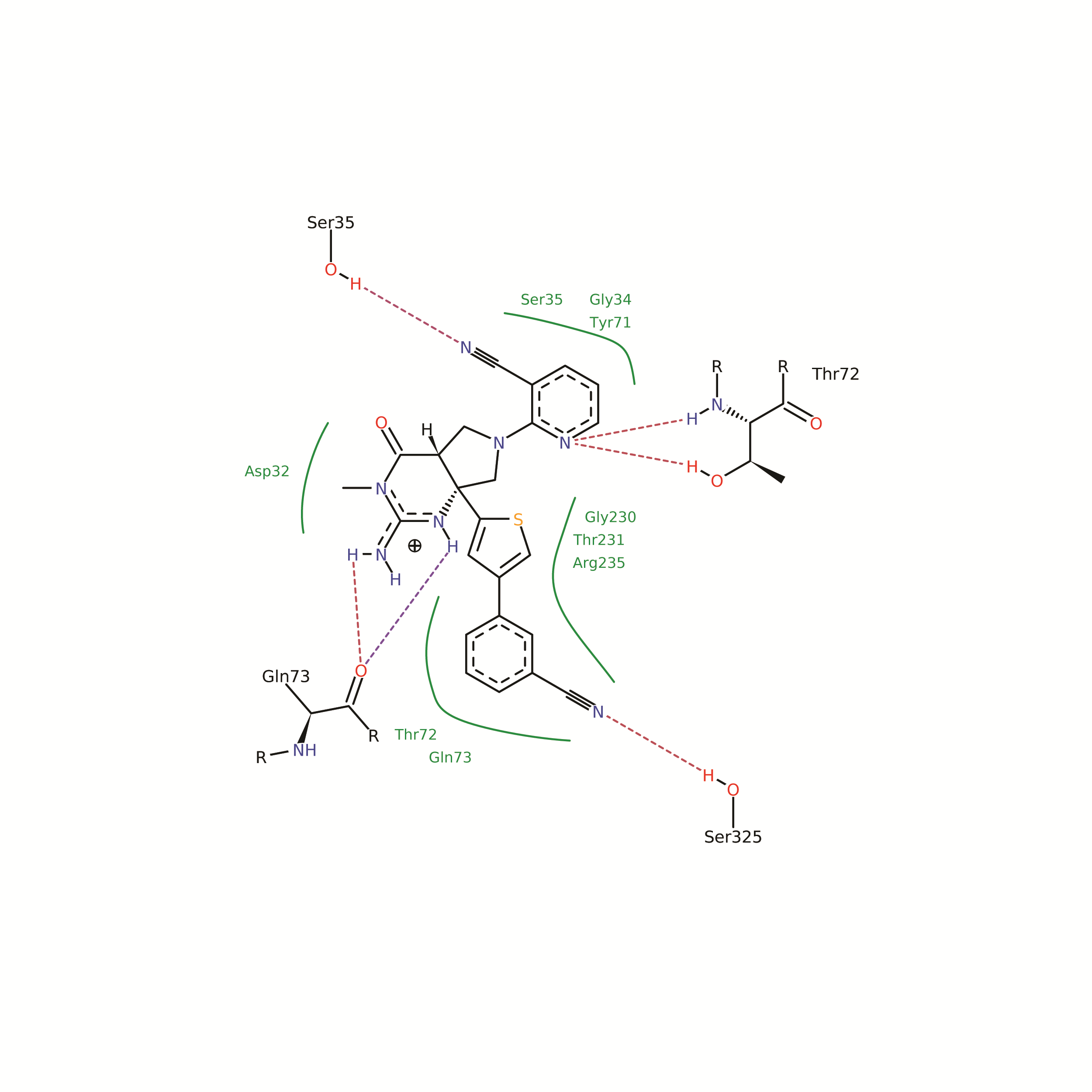 |
| 2P4J-0KQ | -23.62 | -40.699 | 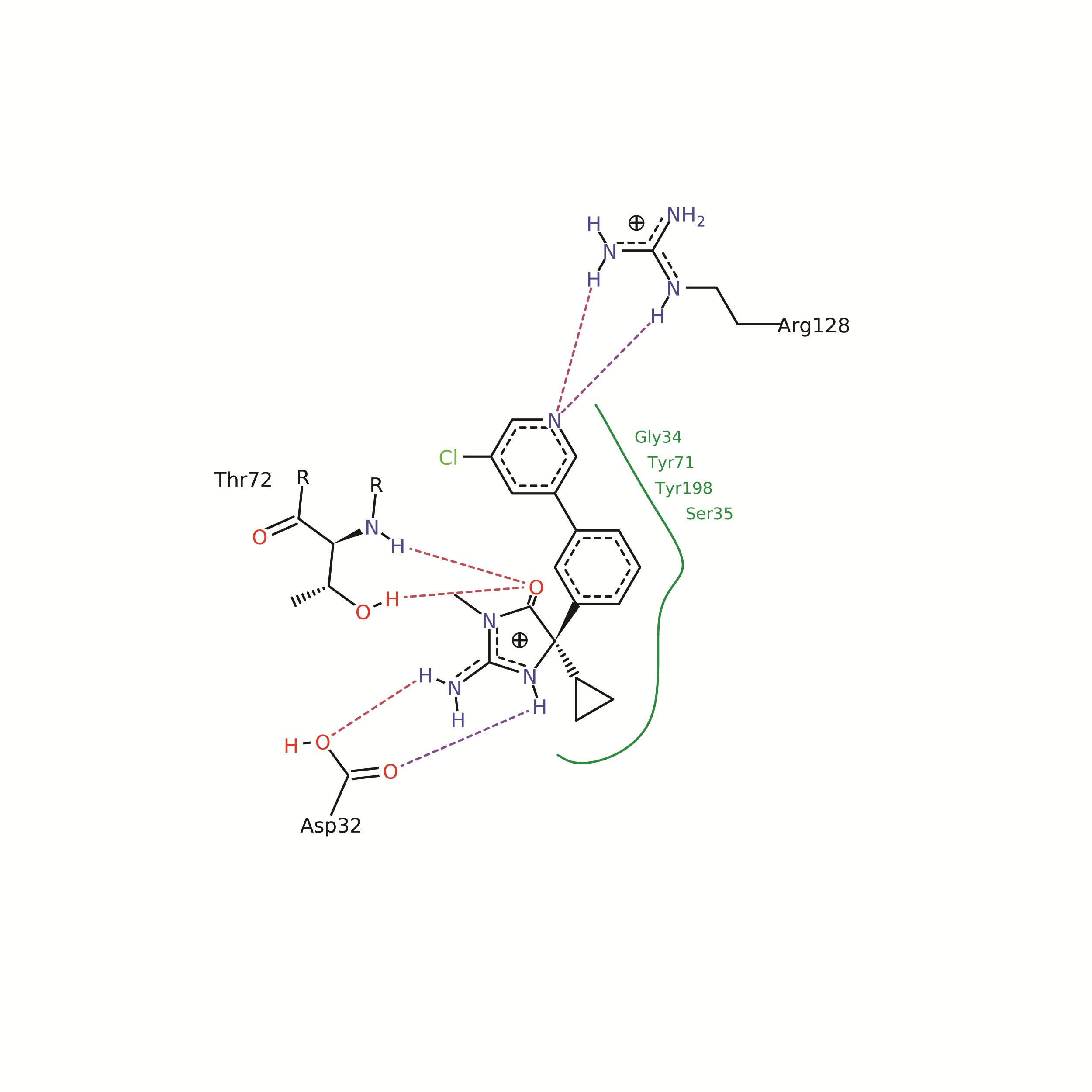 |
| 2P4J-13W | -20.52 | -59.034 | 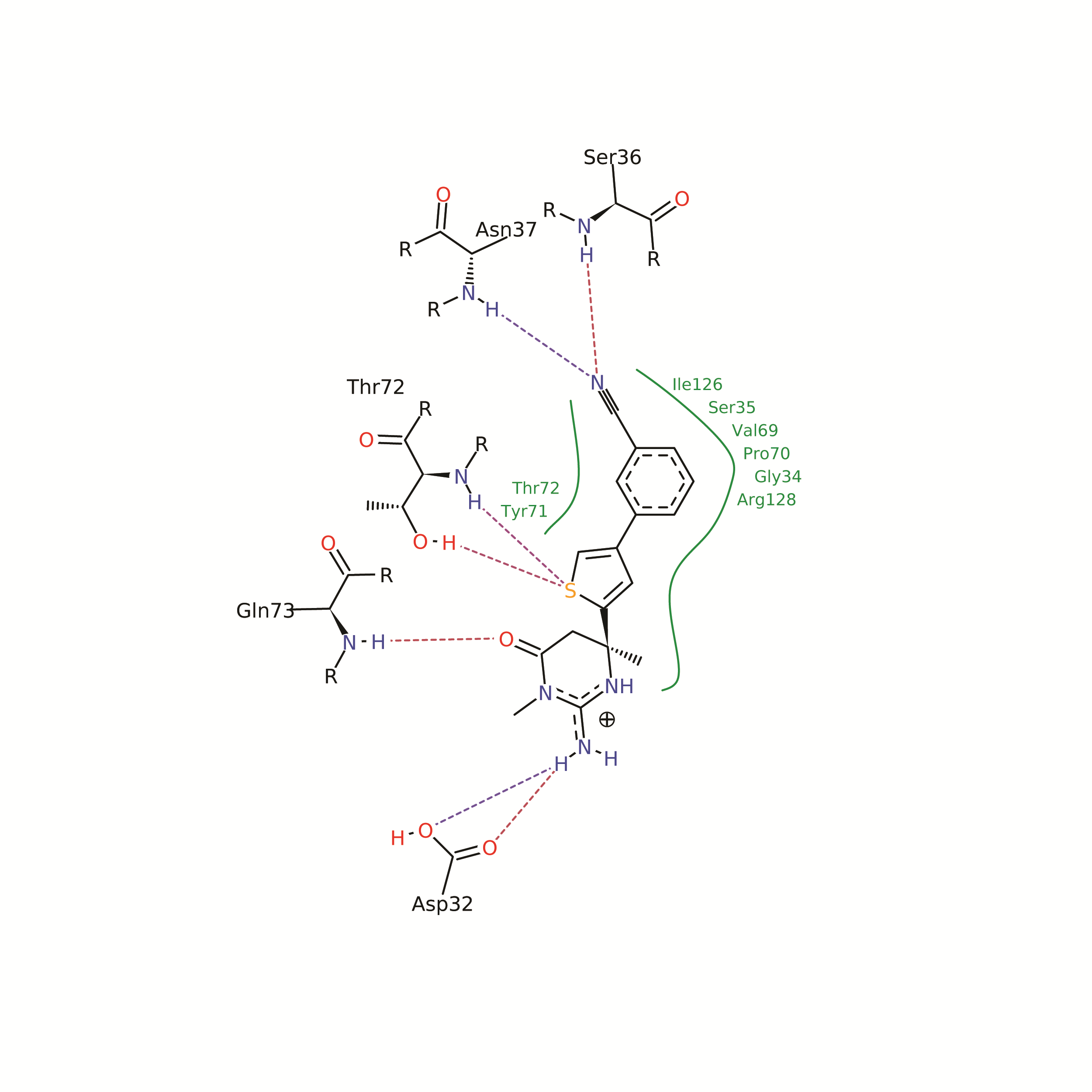 |
| 2P4J-H24 | -21.07 | -33.948 | 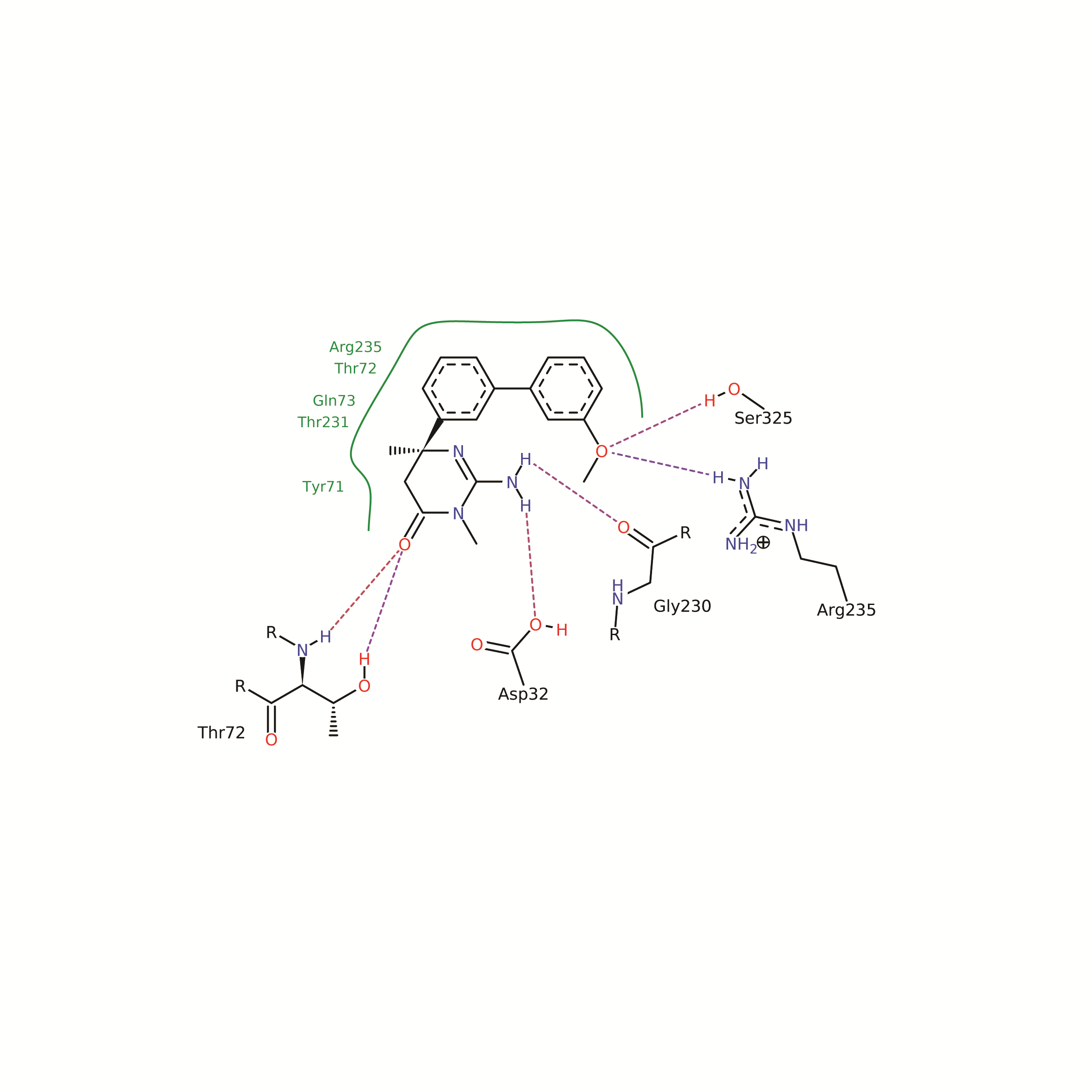 |
| **Cross-docking studies with 4H3G using parameter 3** | | | |
| 4H3G-23I | -36.38 | -60.315 | 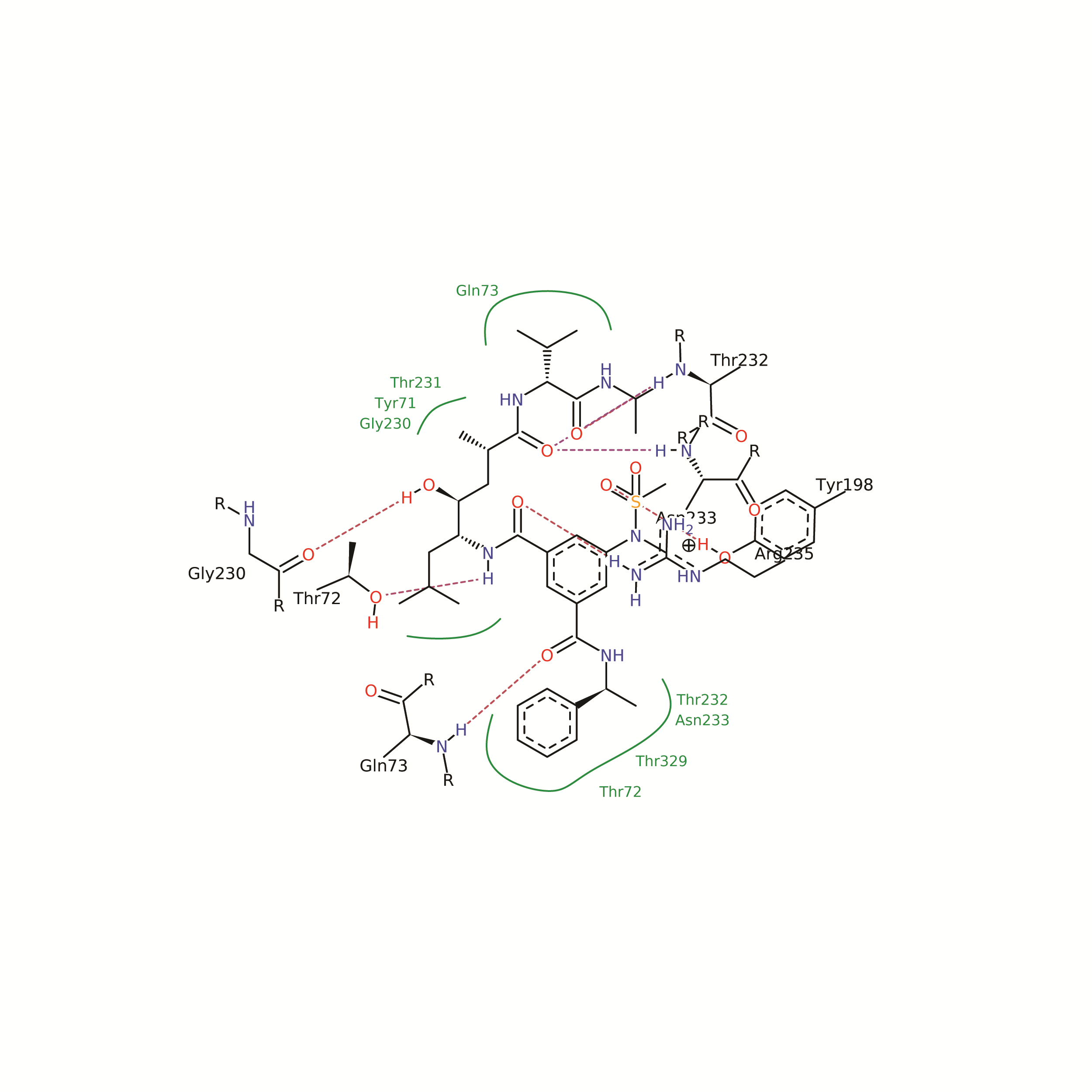 |
| 4H3G-SC6 | -34.94 | -79.57 | 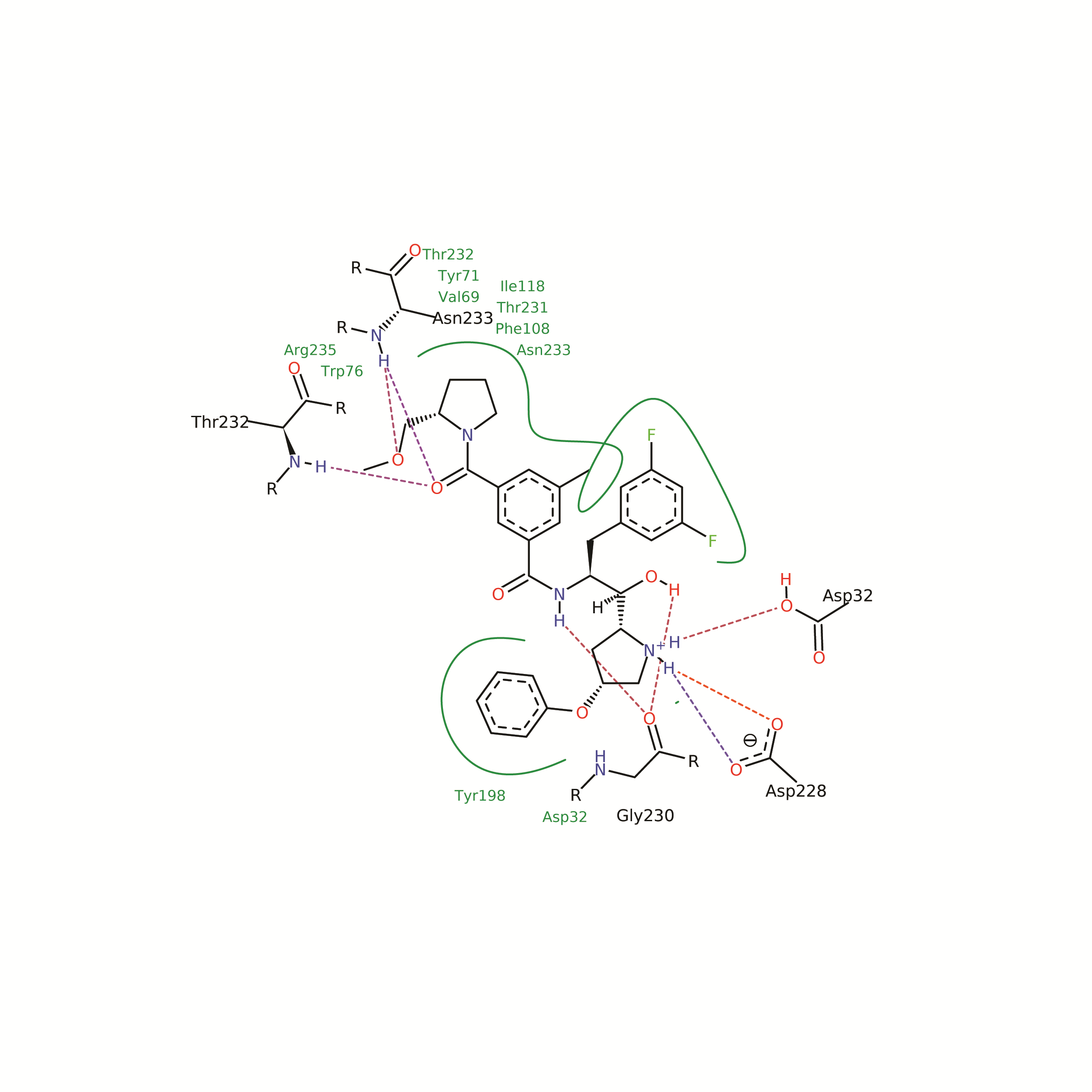 |
| 4H3G-Z76 | -36.3 | -89.197 | 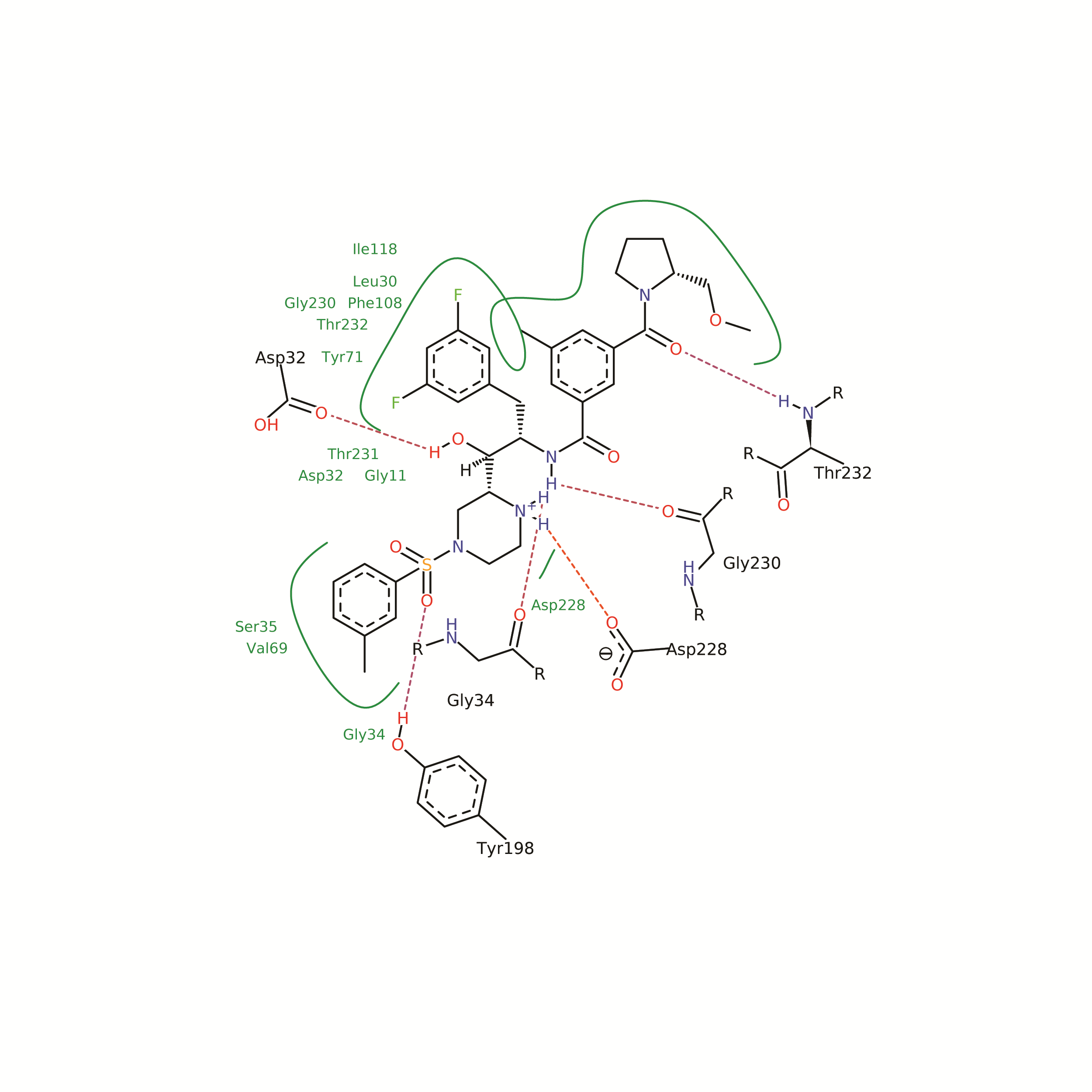 |
| 4H3G-316 | -29.16 | -66.56 | 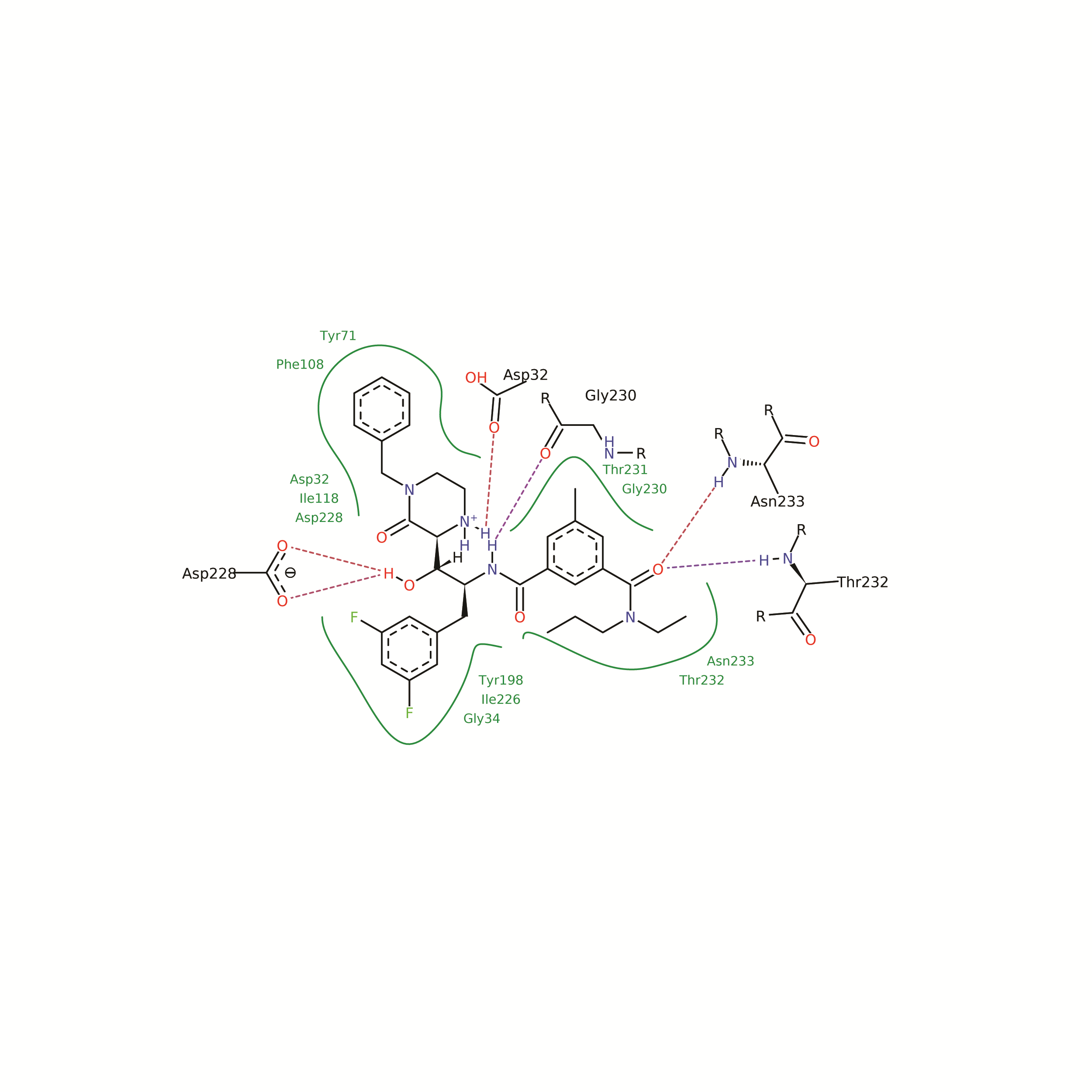 |
| 4H3G-10Q | -26.31 | -84.064 | 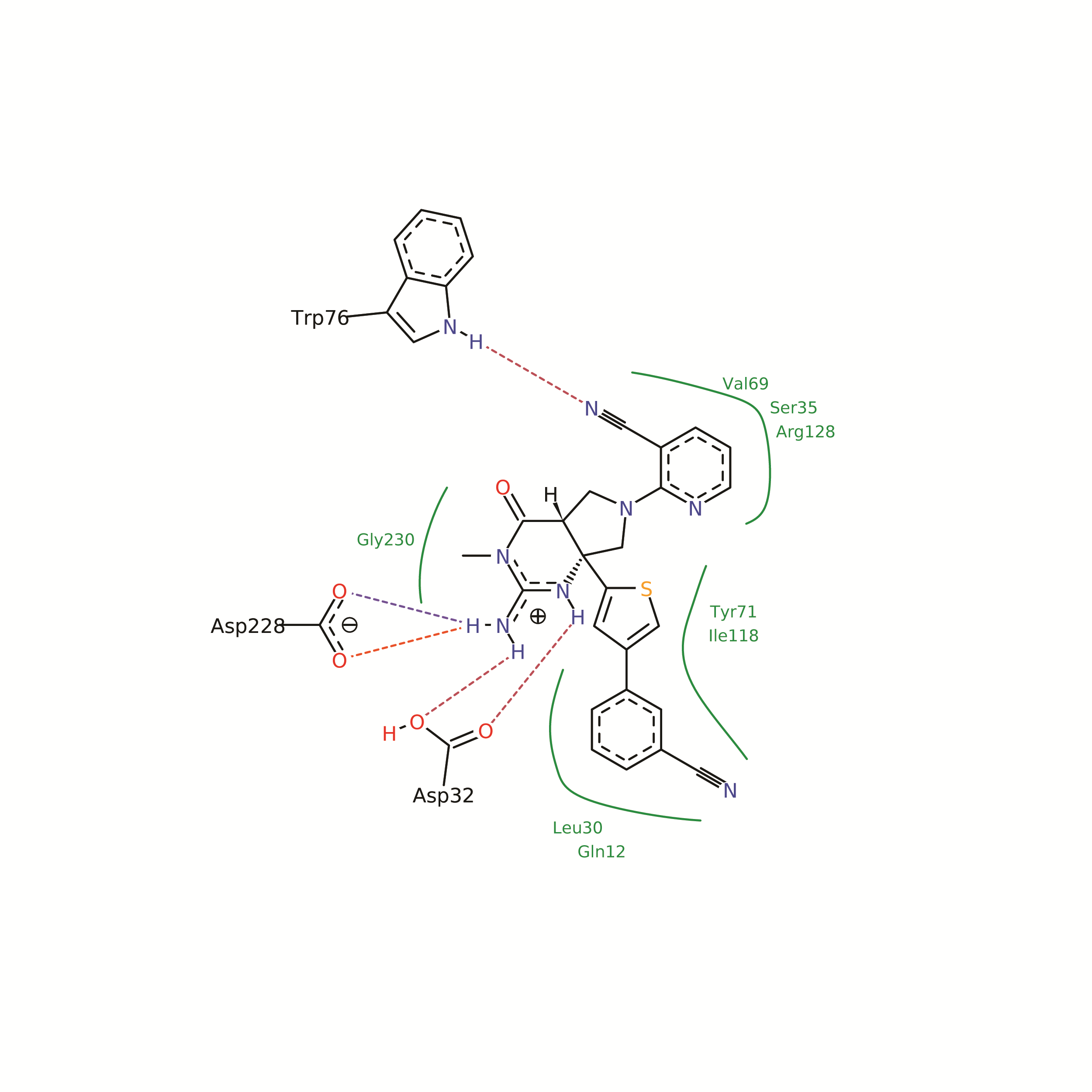 |
| 4H3G-0KQ | -26.66 | -68.44 | 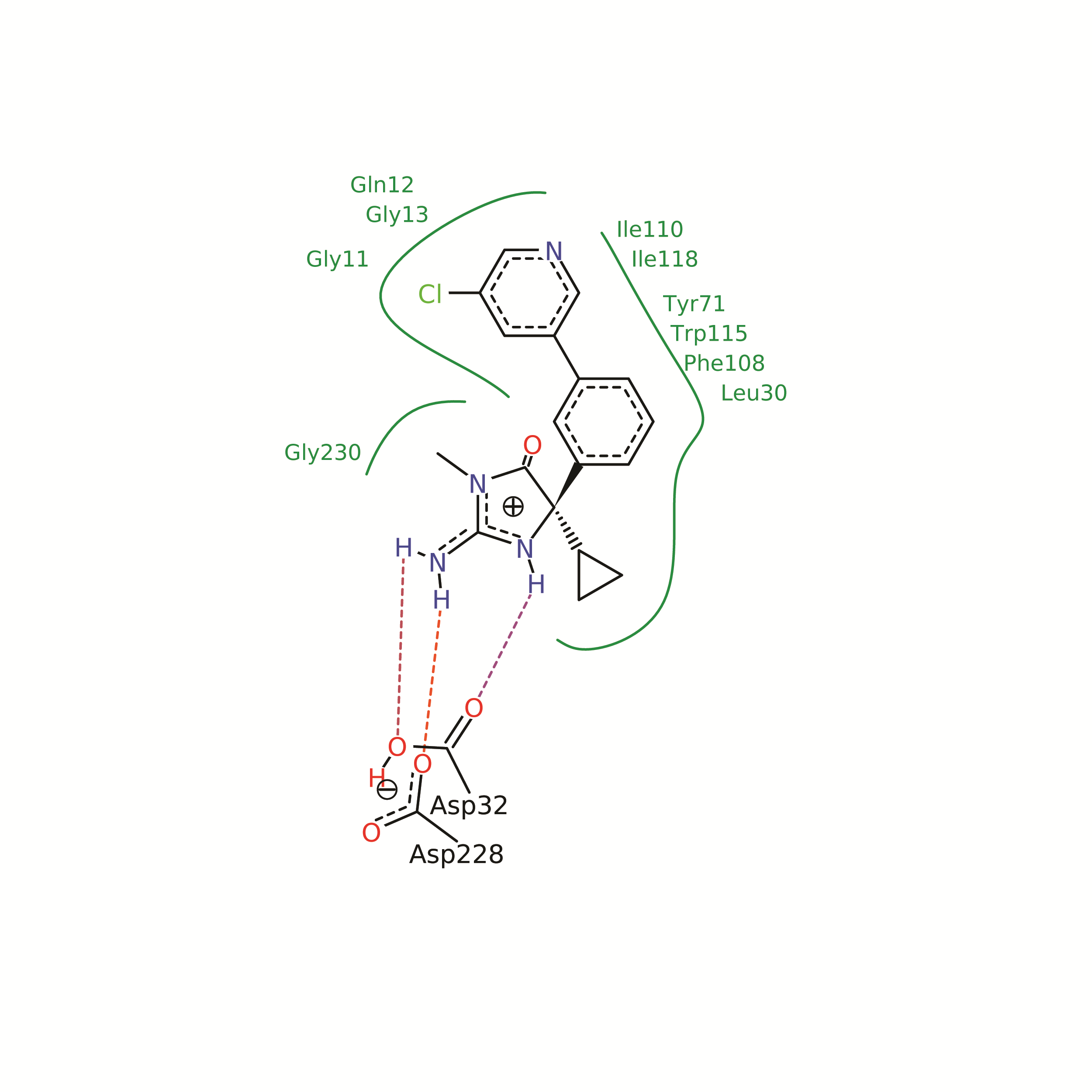 |
| 4H3G-13W | -26.99 | -62.24 | 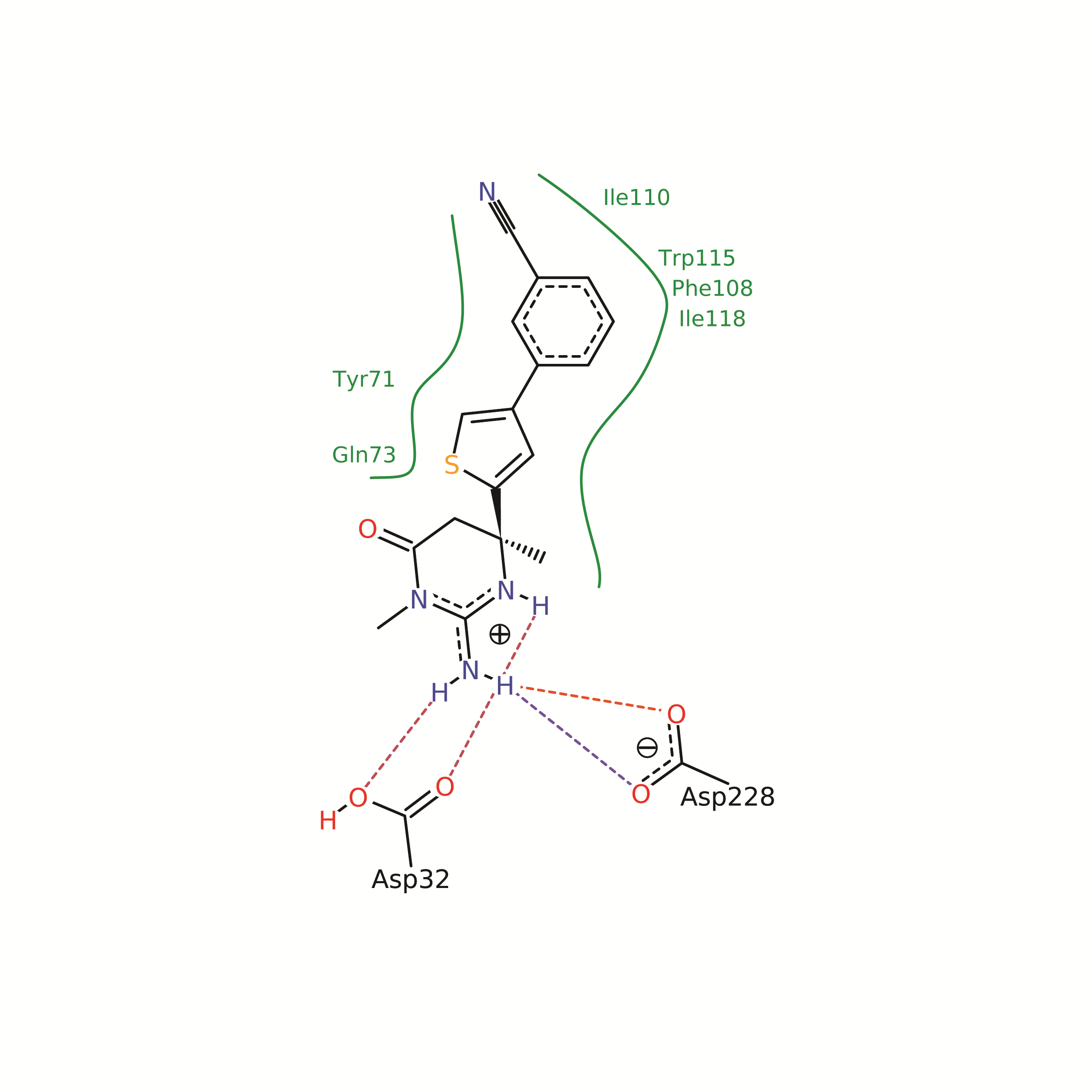 |
| 4H3G-H24 | -21.27 | -48.827 | 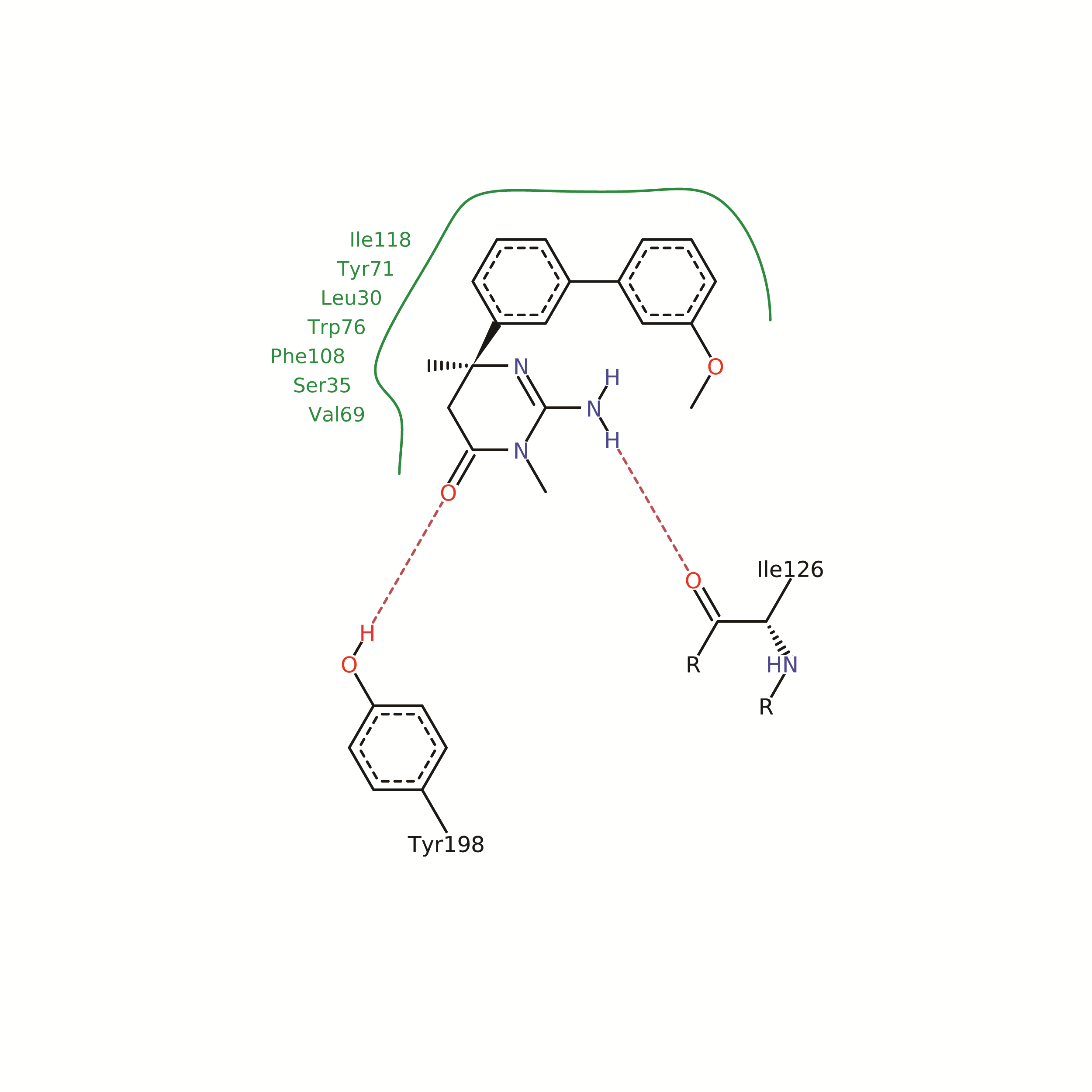 |
| **Cross-docking studies with 4DJX as receptor using parameter 3** | | | |
| 4DJX-23I | -36.3 | -65.28 | 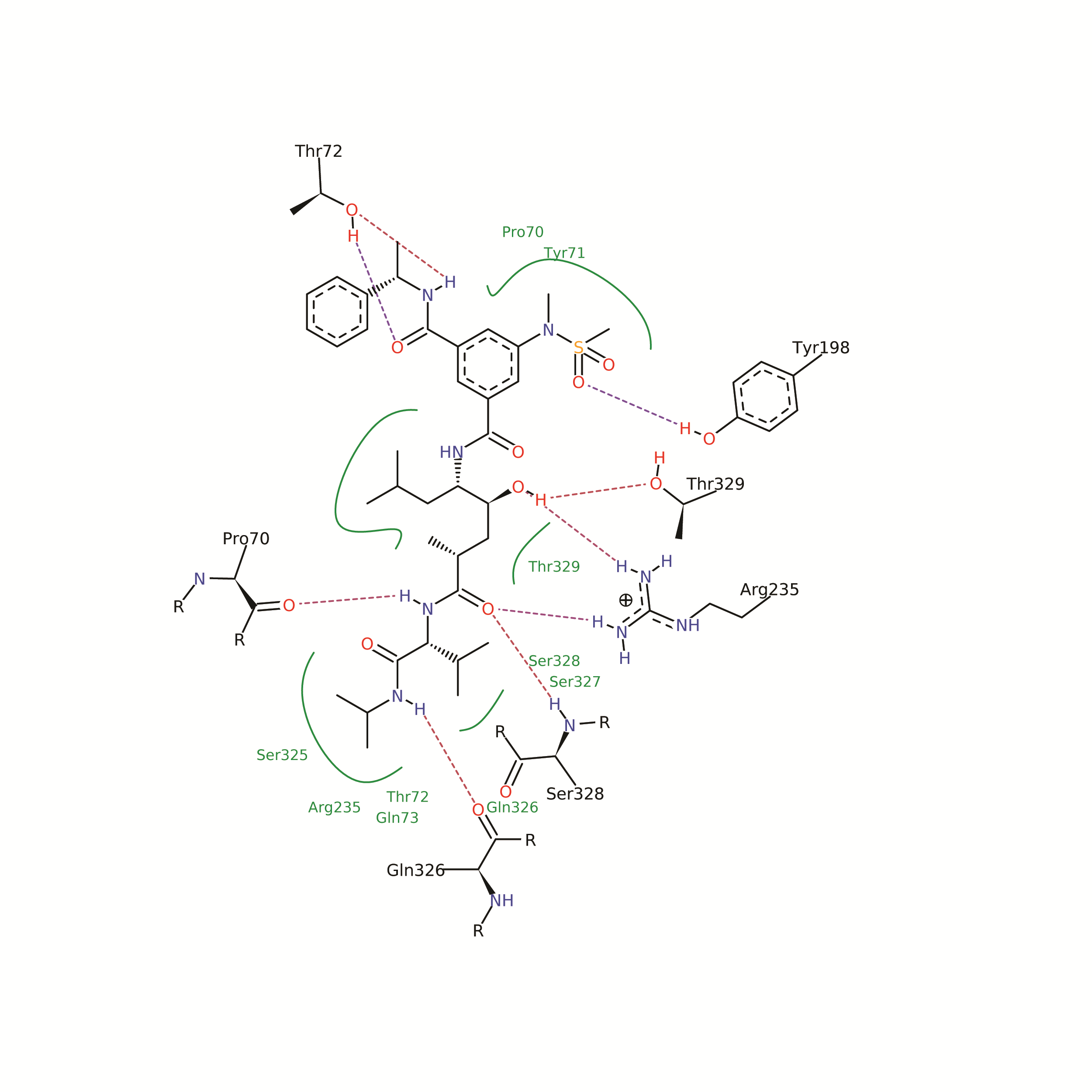 |
| 4DJX-SC6 | -41.98 | -75.61 | 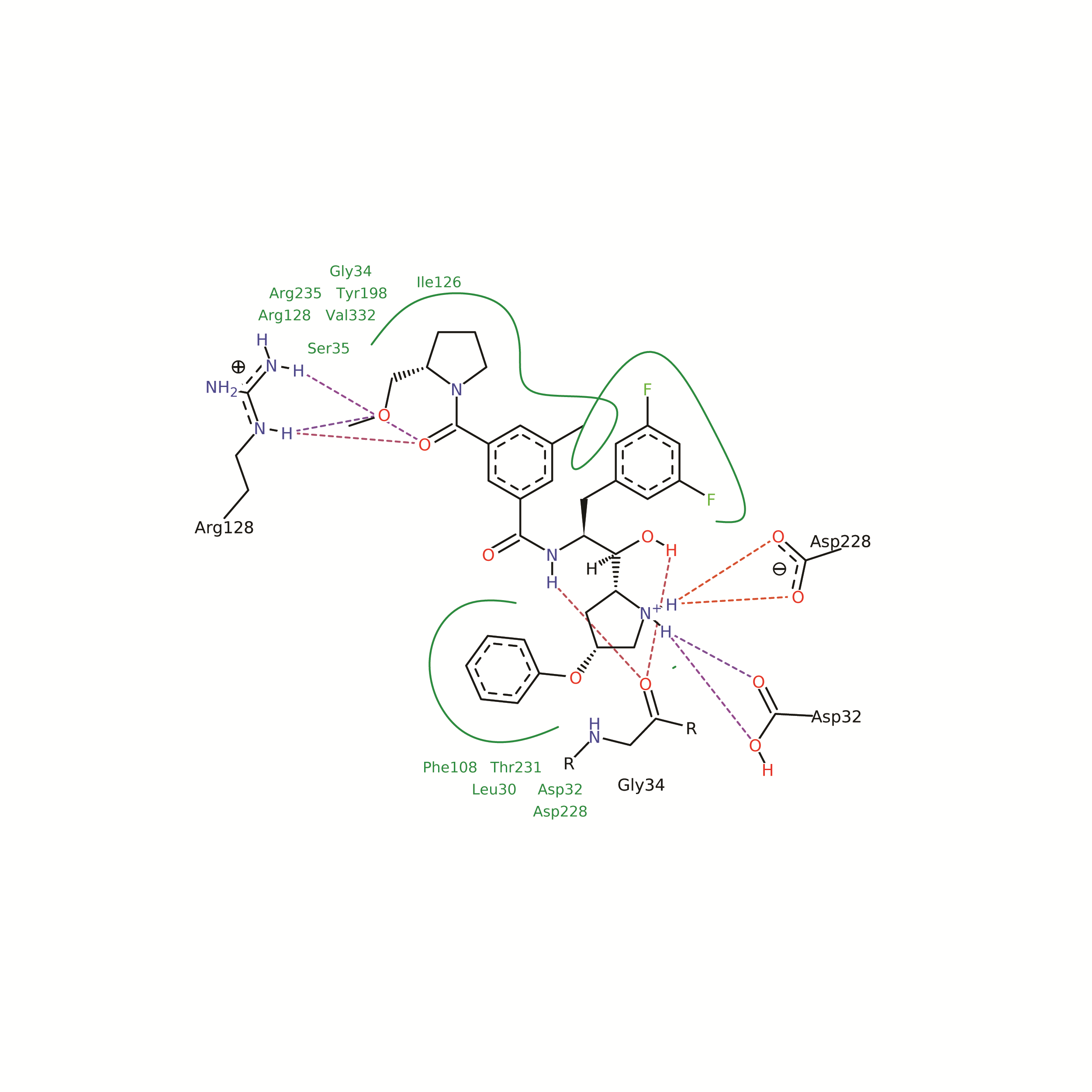 |
| 4DJX-Z76 | -36.79 | -63.098 | 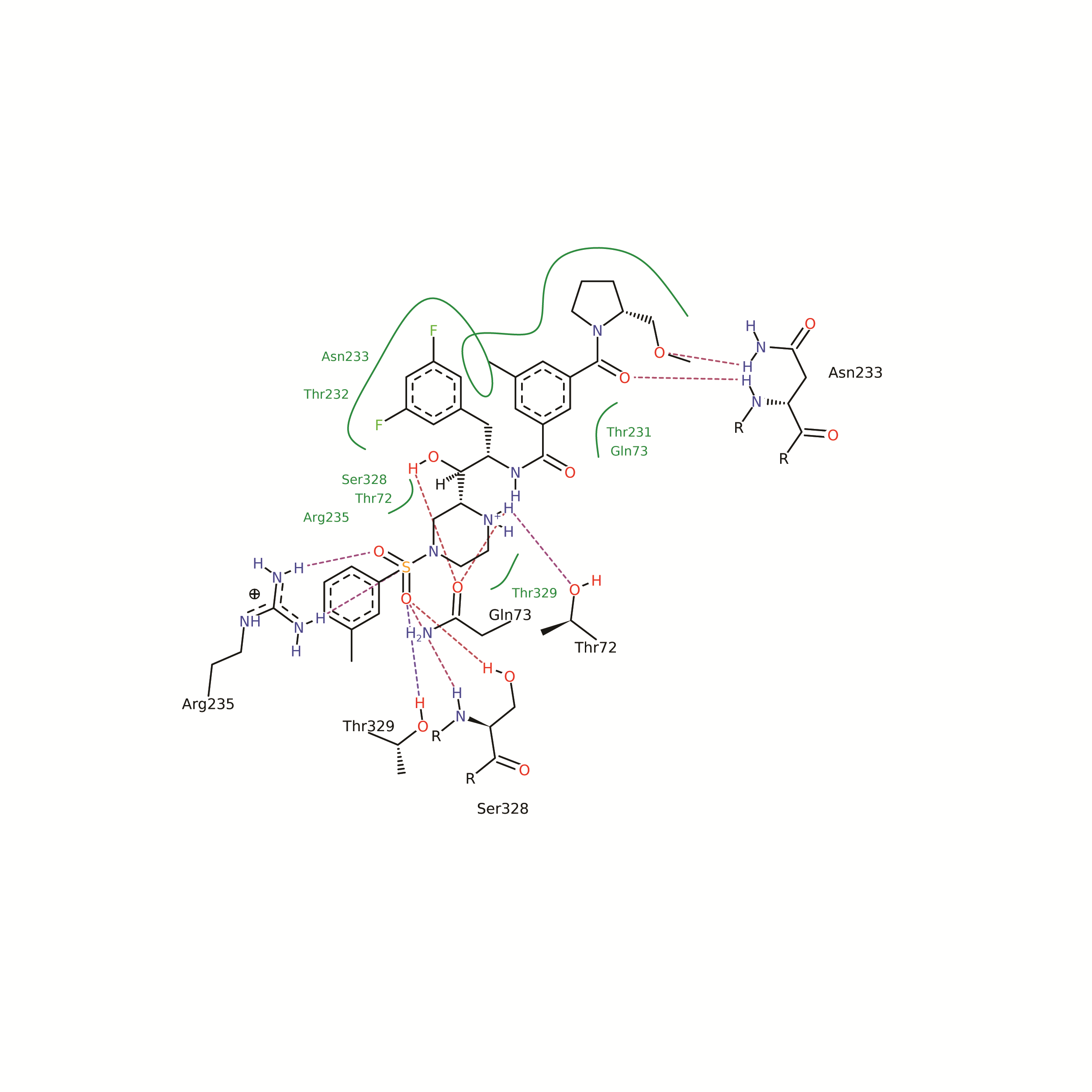 |
| 4DJX-316 | -33.02 | -105.37 | 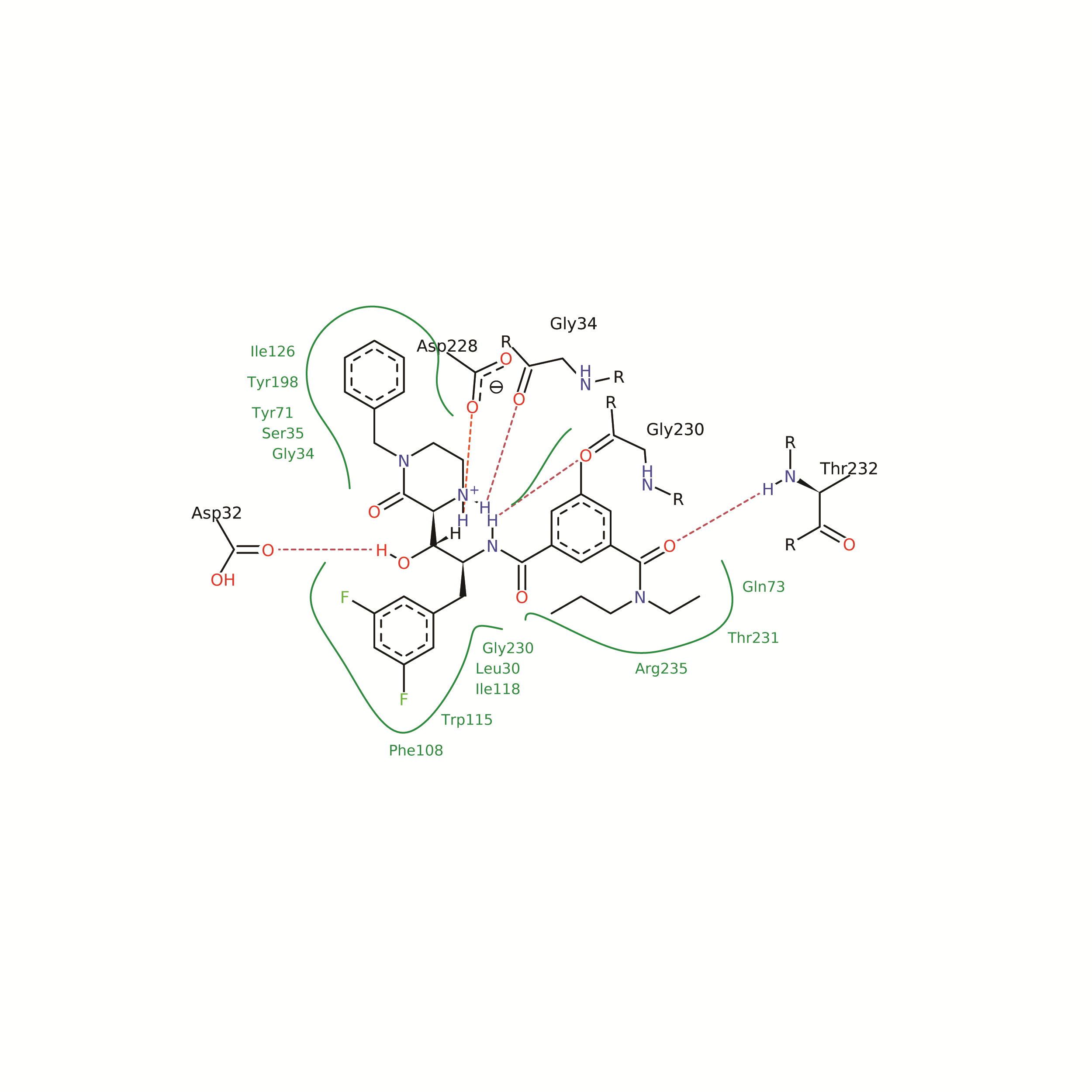 |
| 4DJX-10Q | -22.5 | -88.46 | 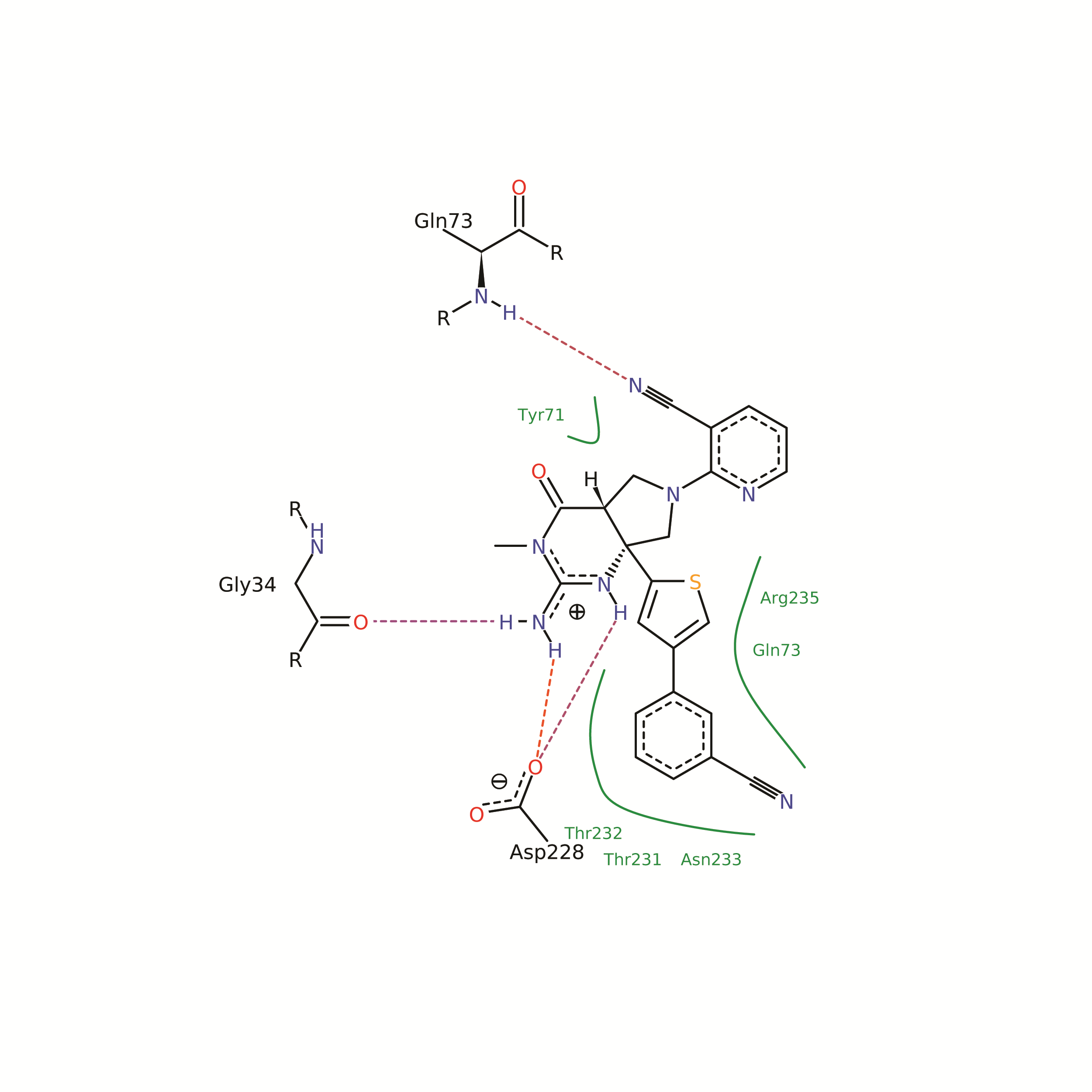 |
| 4DJX-0KQ | -22.73 | -59.29 | 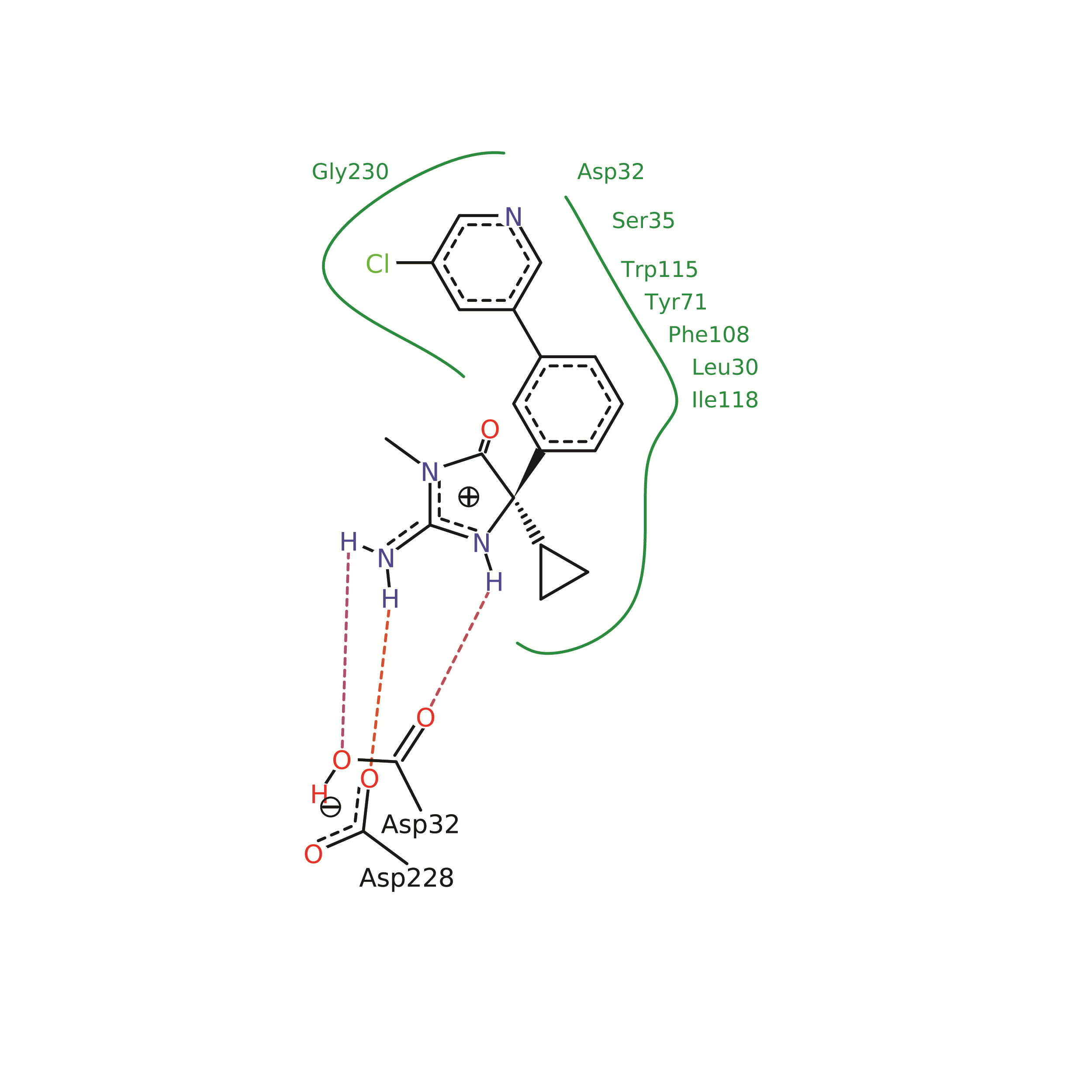 |
| 4DJX-13W | -23.28 | -72.3 | 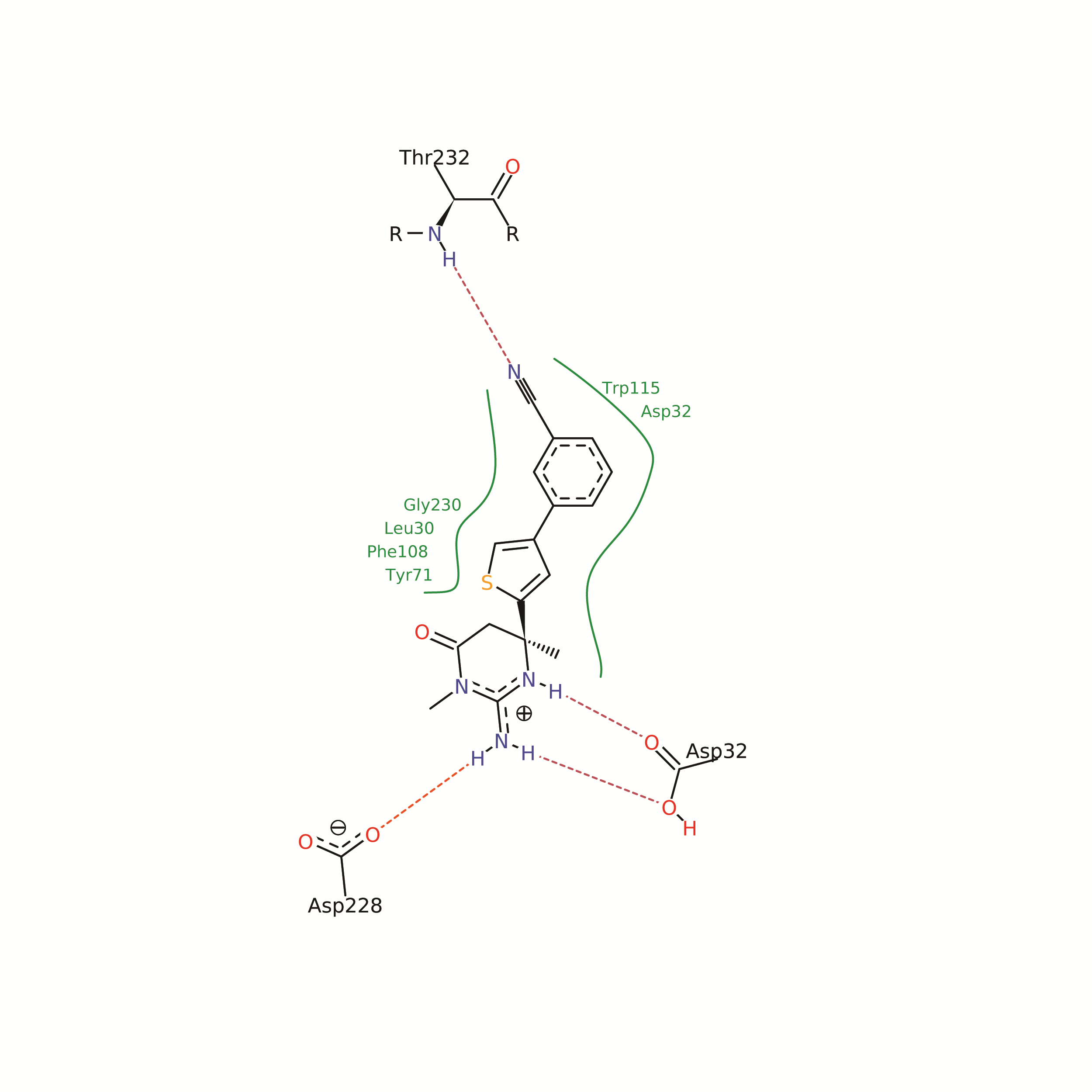 |
| 4DJX-H24 | -20.85 | -25.17 | 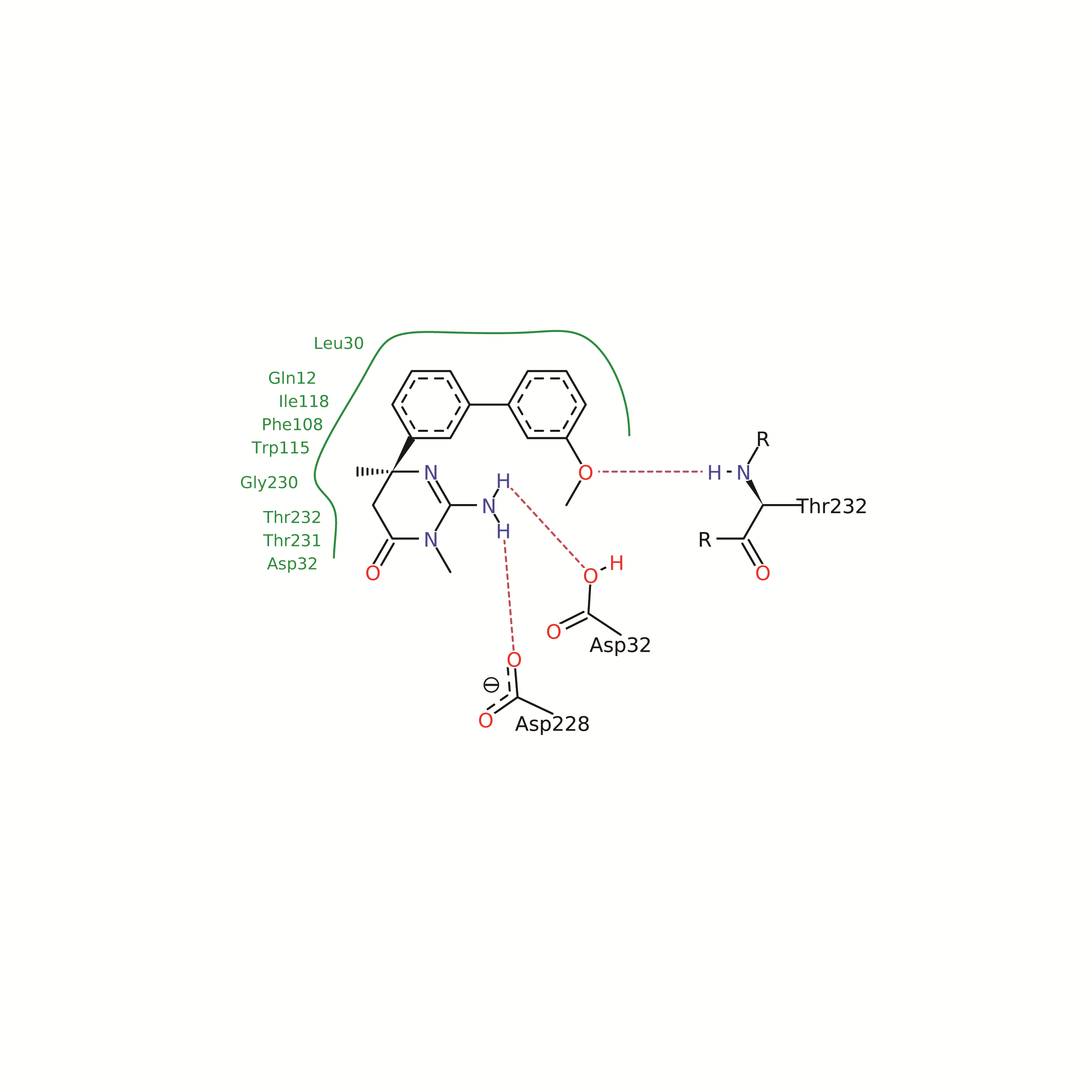 |
| **Cross-docking with 4DJX using parameter 2** | | | |
| 4DJX-23I | -34.55 | -56.13 | 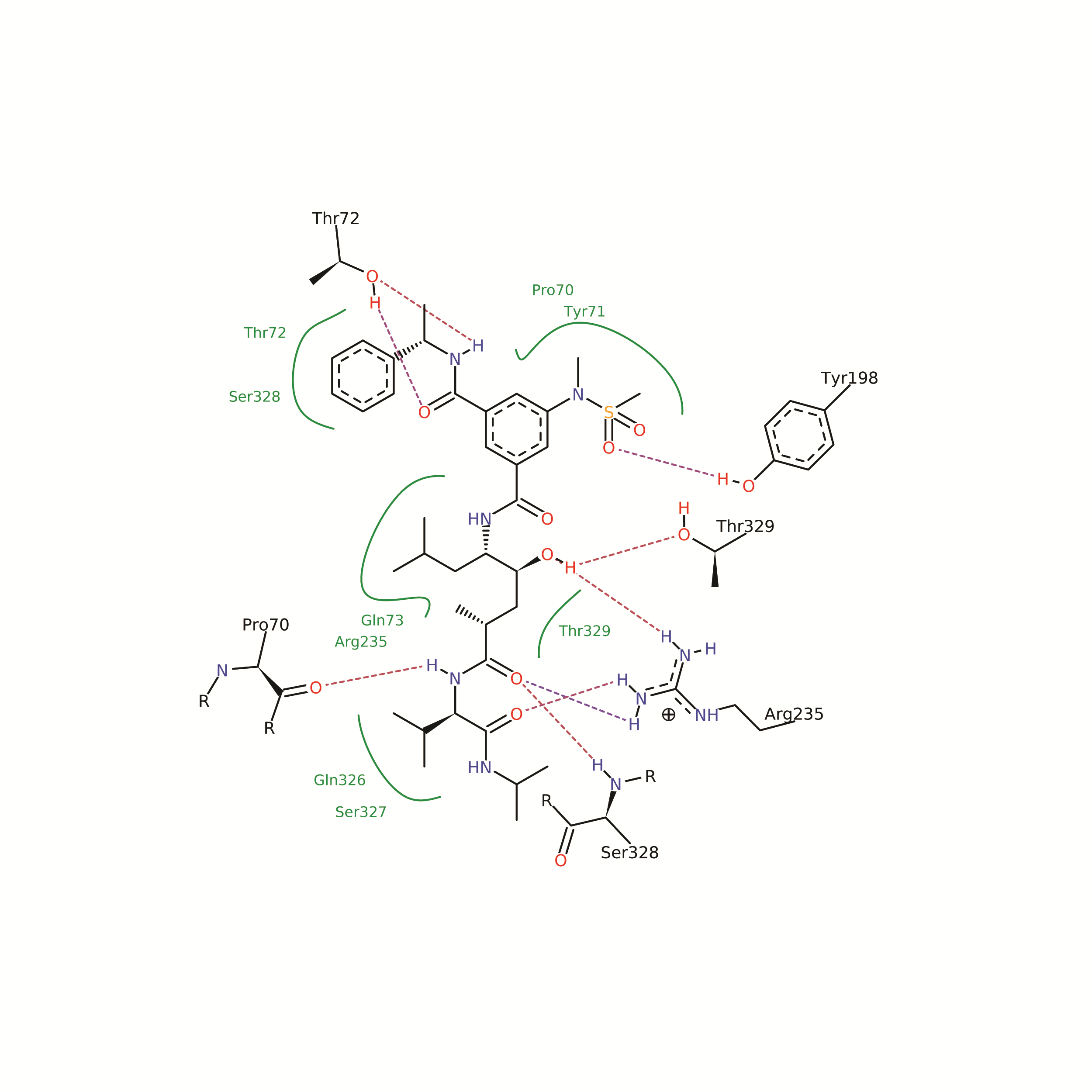 |
| 4DJX-SC6 | -31.58 | -83.27 | 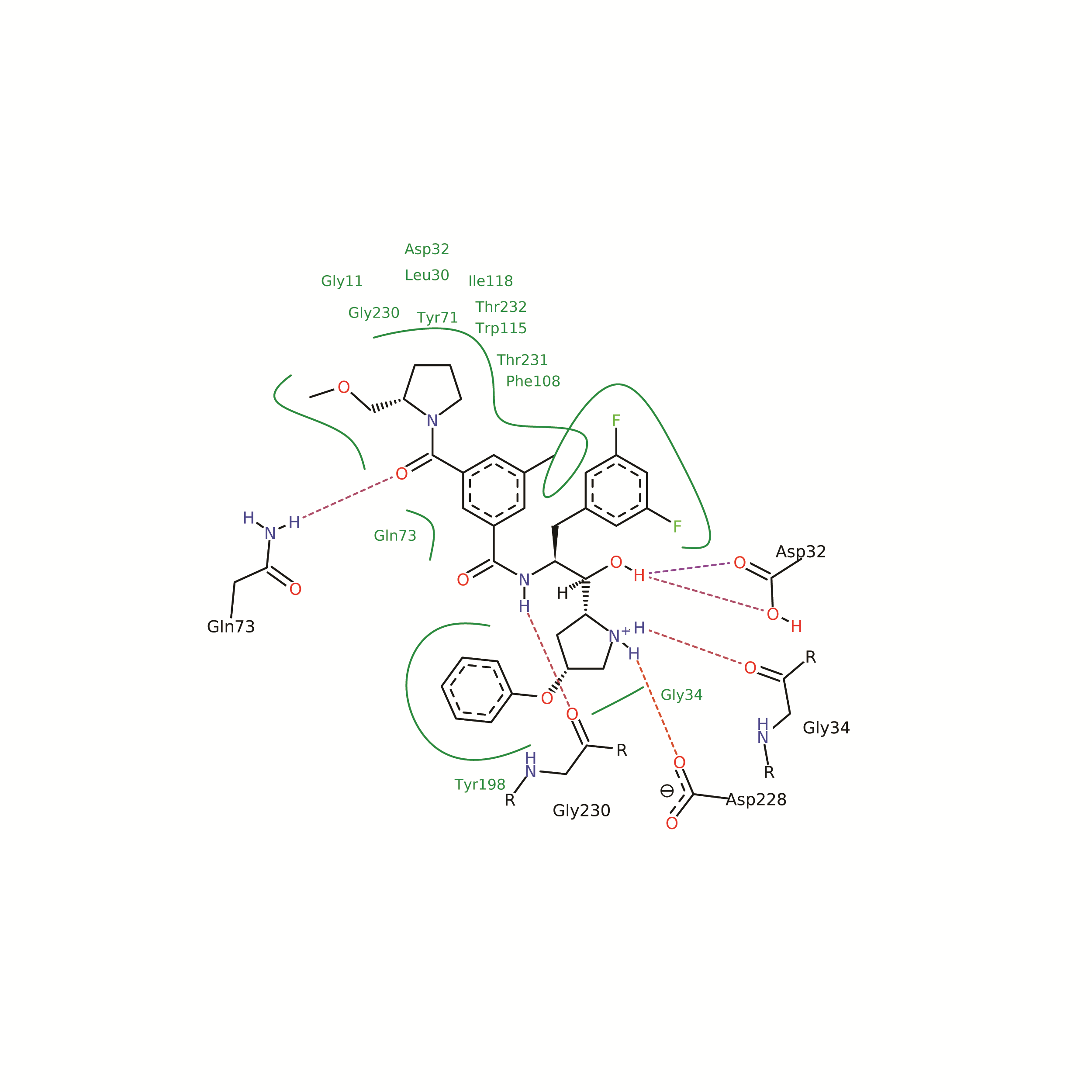 |
| 4DJX-Z76 | -36.62 | -61 | 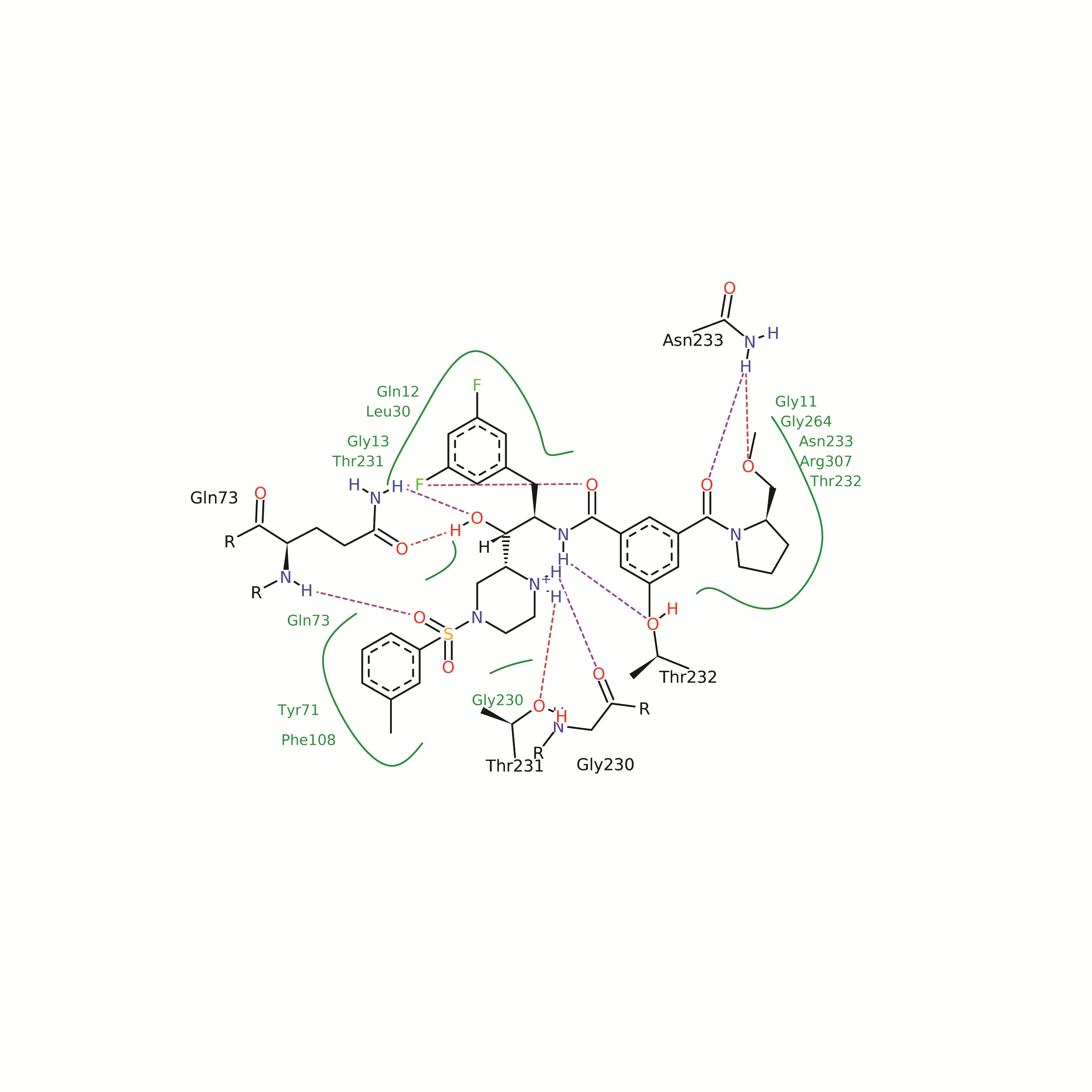 |
| 4DJX-316 | -25.7 | -88.67 | 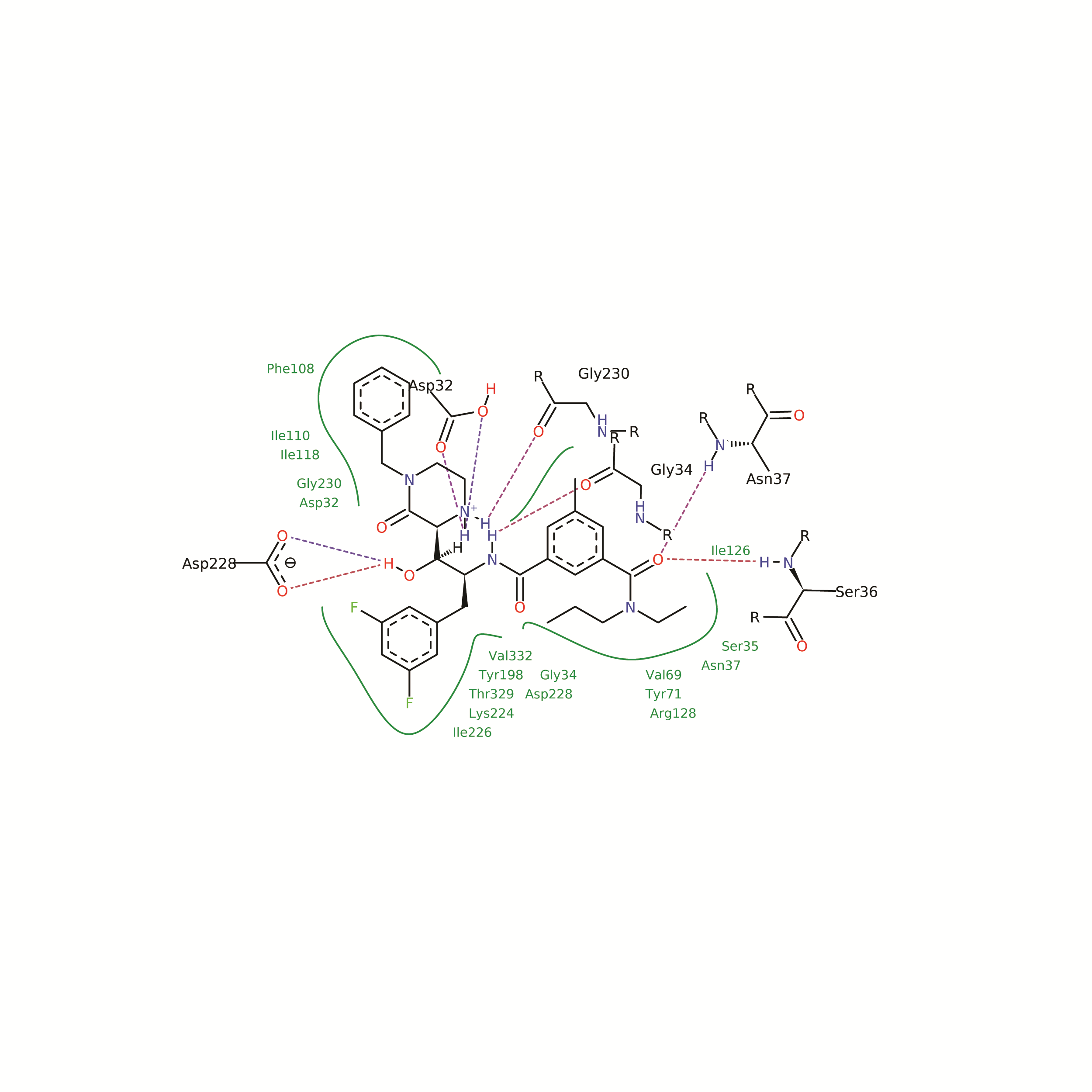 |
| 4DJX-10Q | -22.5 | -88.469 | 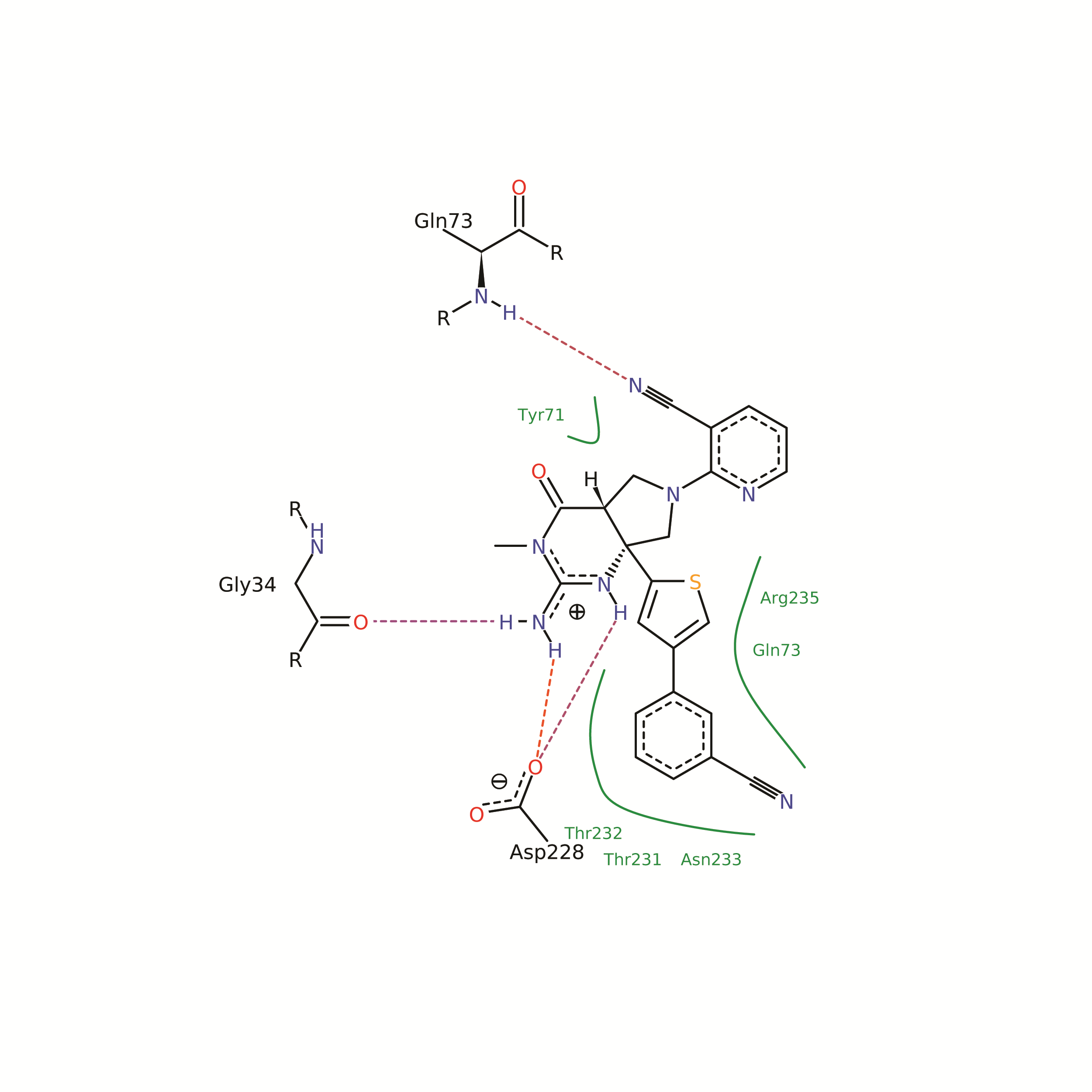 |
| 4DJX-0KQ | -24.28 | -68.11 | 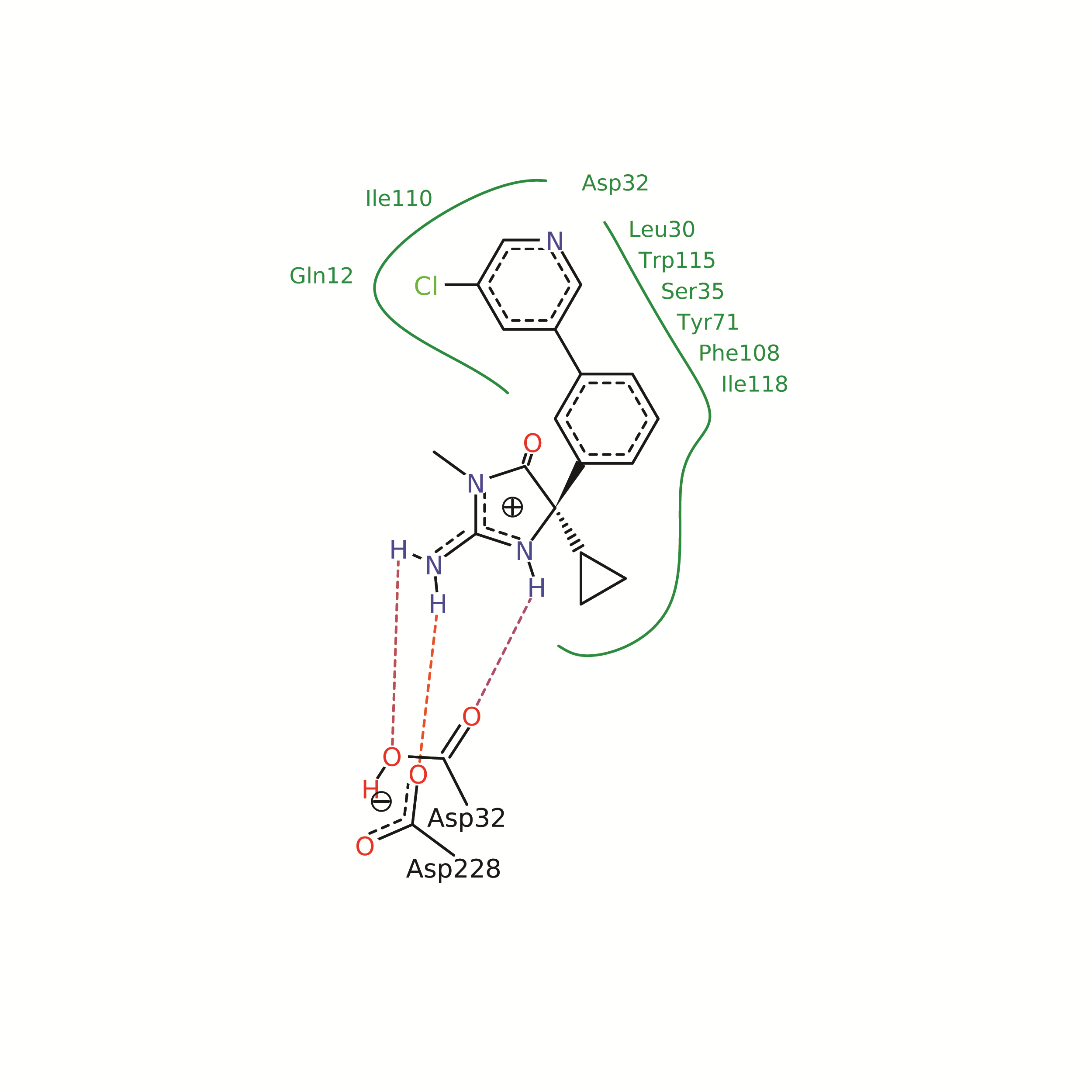 |
| 4DJX-13W | -22.16 | -54.74 | 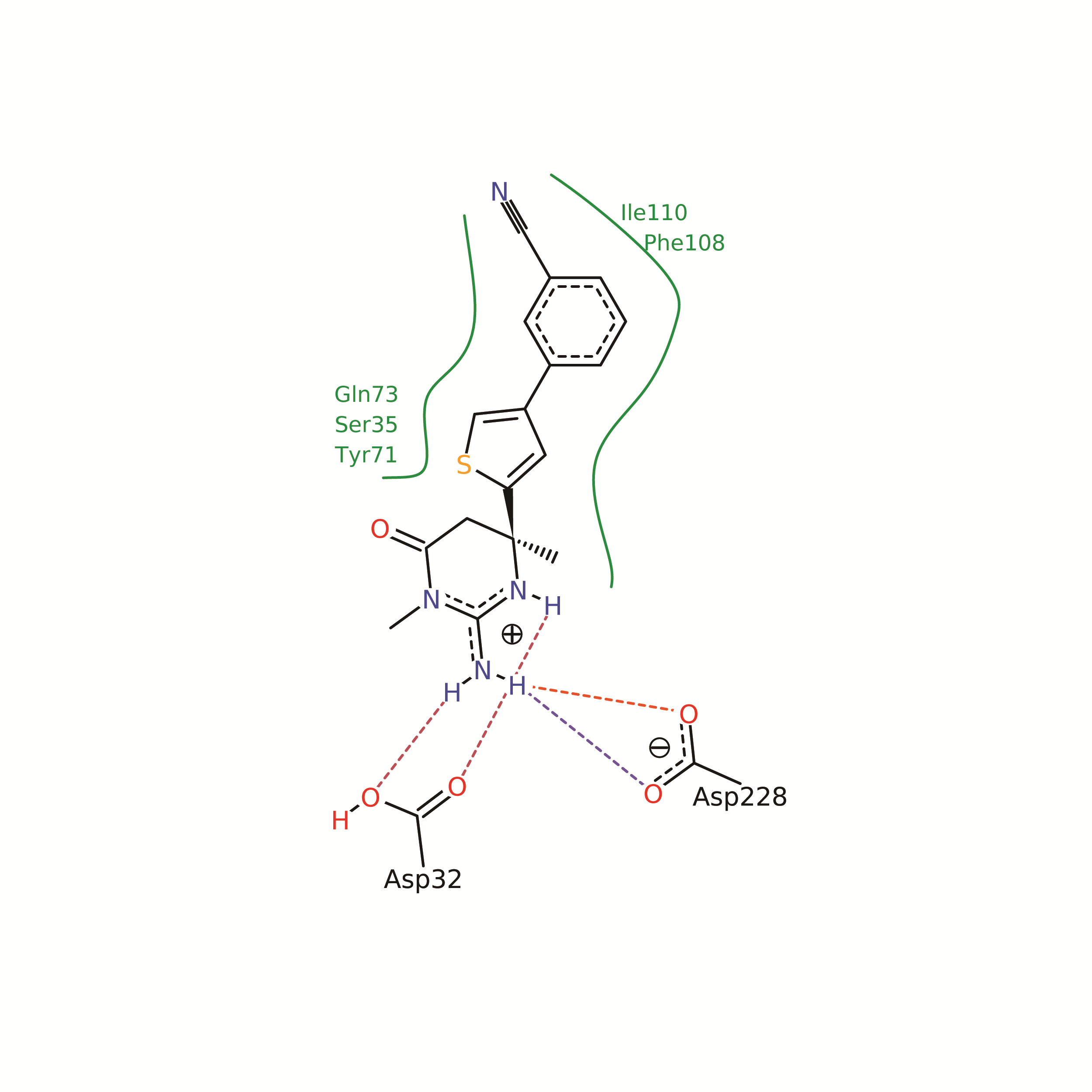 |
| 4DJX-H24 | -19.58 | -25.21 | 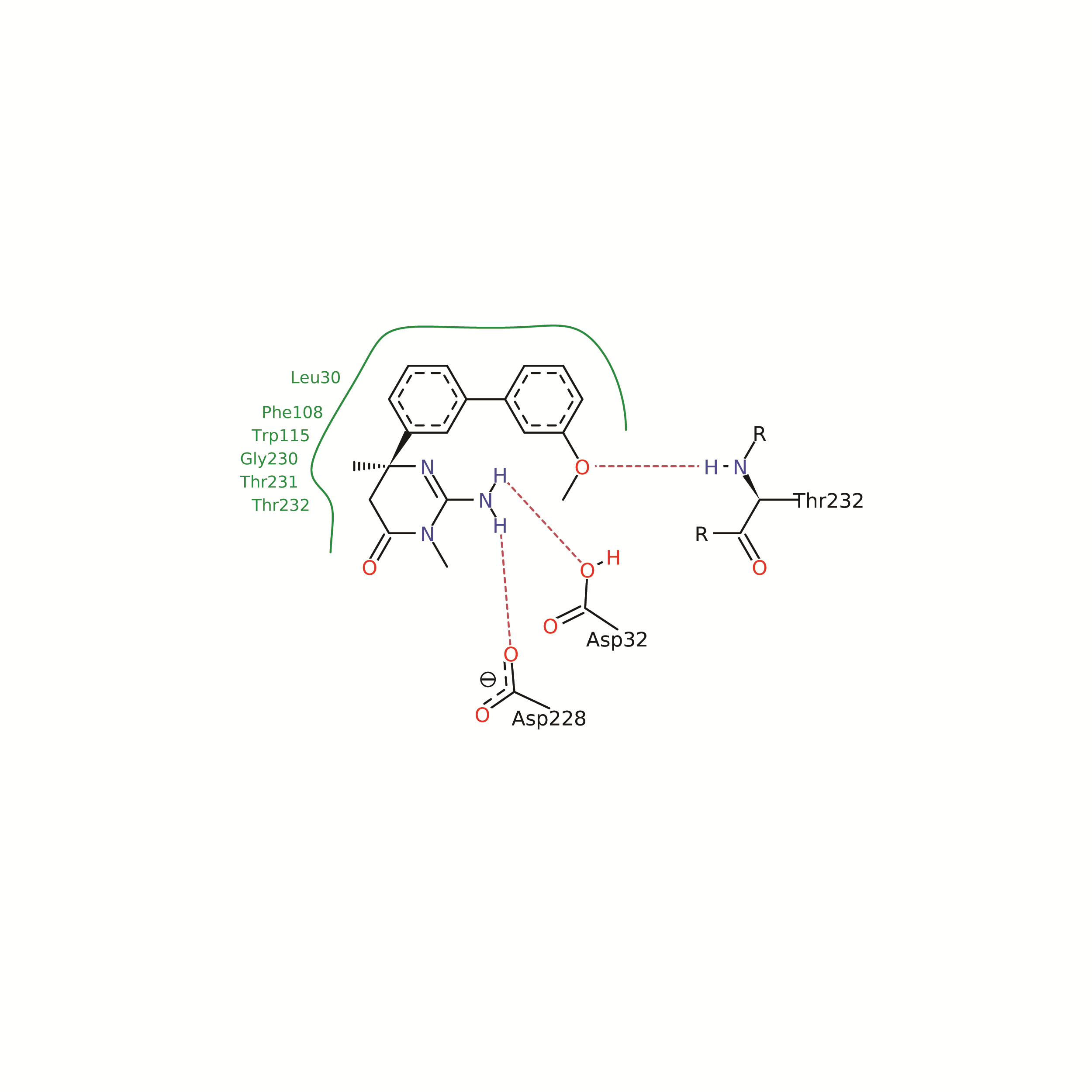 |
| **Cross-docking with 4DJX using parameter 1** | | | |
| 4DJX-23I | -30.38 | -65.76 | 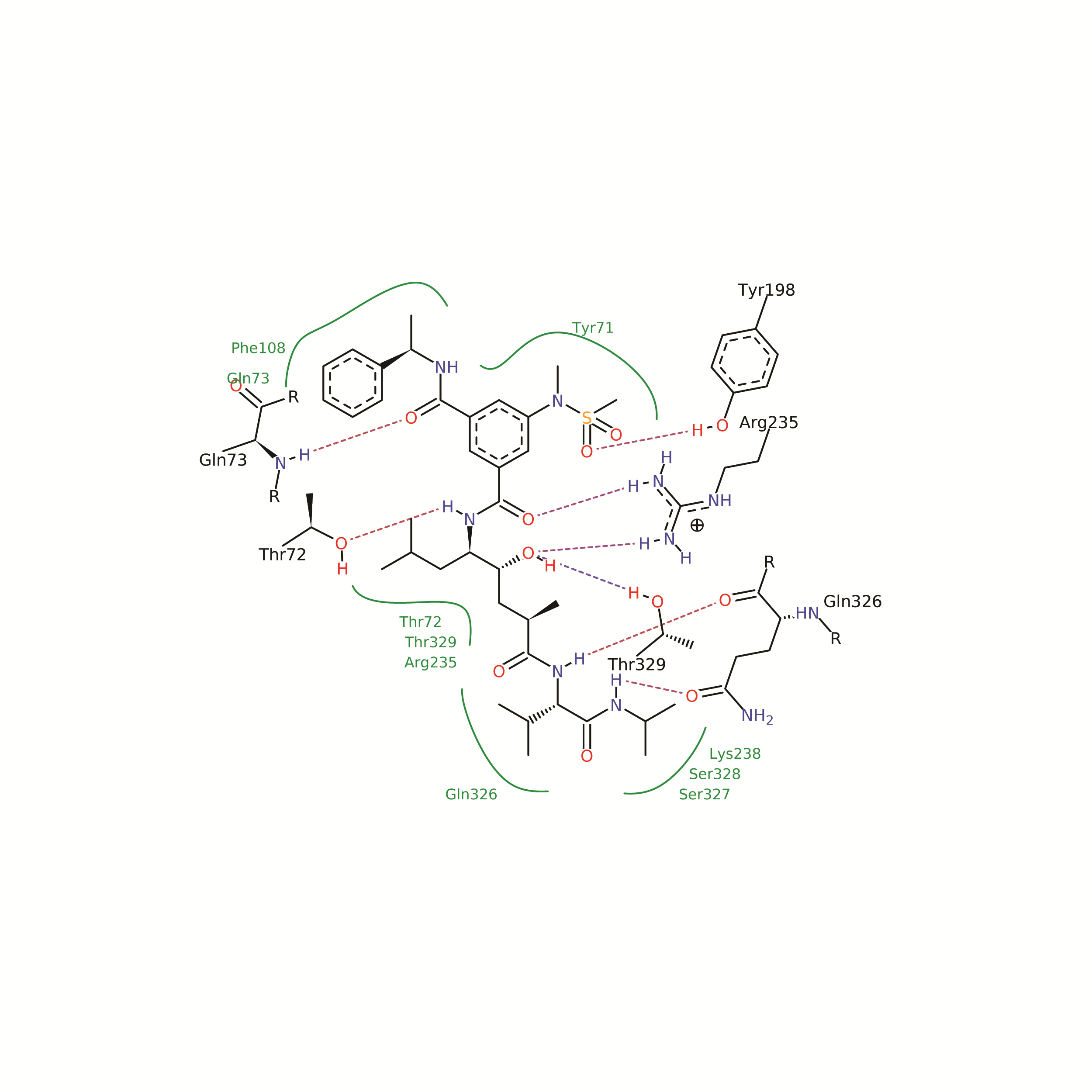 |
| 4DJX-SC6 | -41.44 | -65.39 | 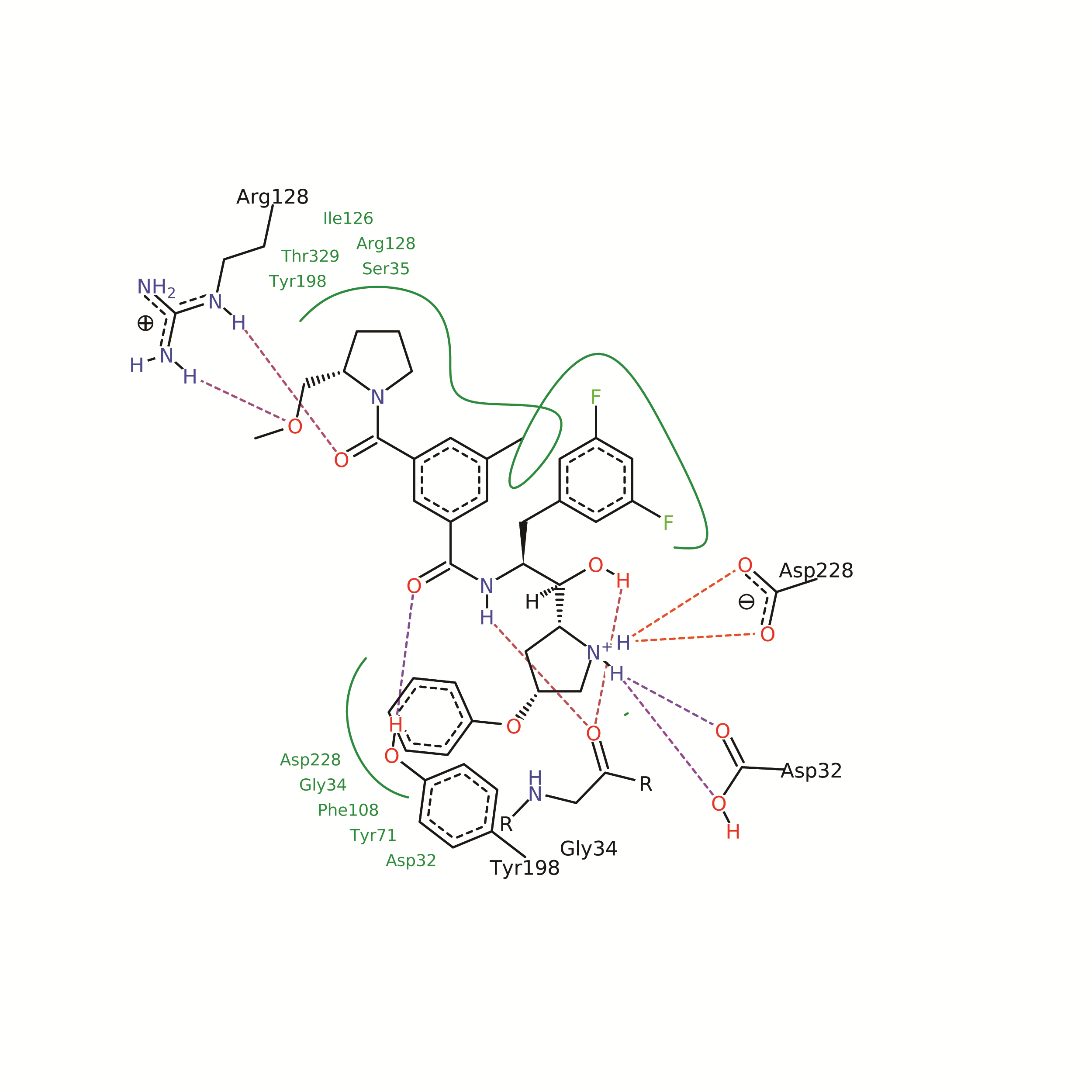 |
| 4DJX-Z76 | -36.79 | -63.09 | 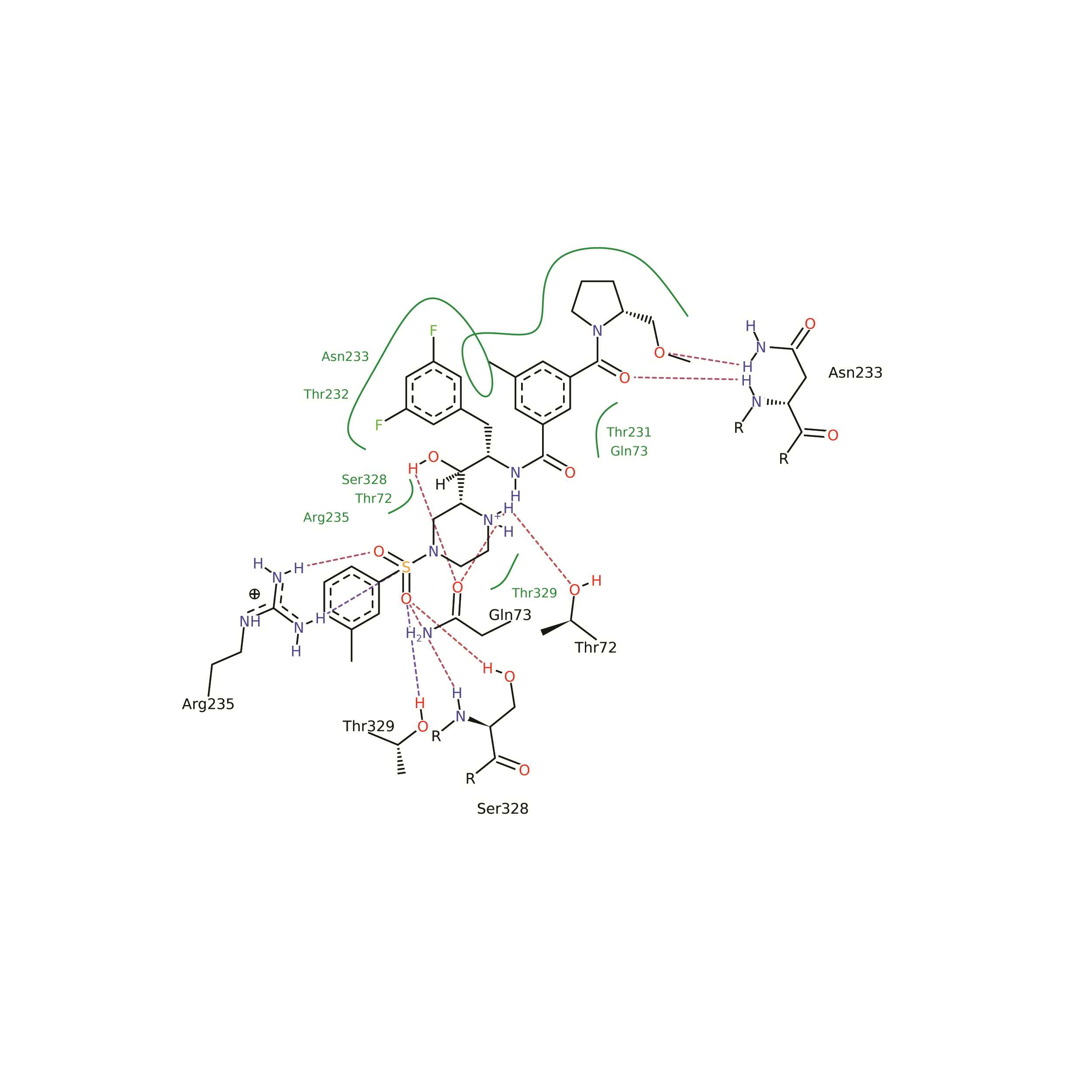 |
| 4DJX-316 | -25.17 | -82.56 | 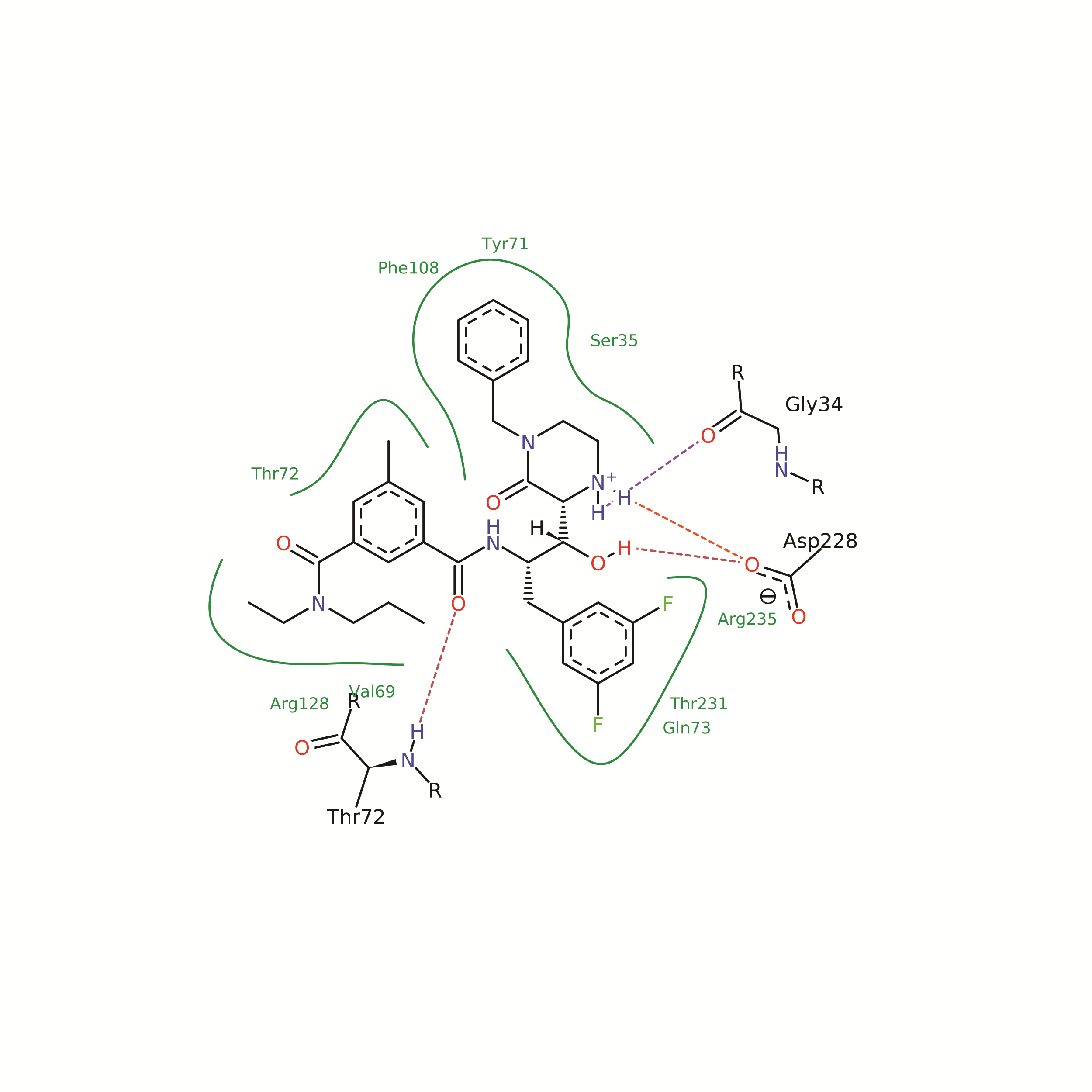 |
| 4DJX-10Q | -21.73 | -62.13 | 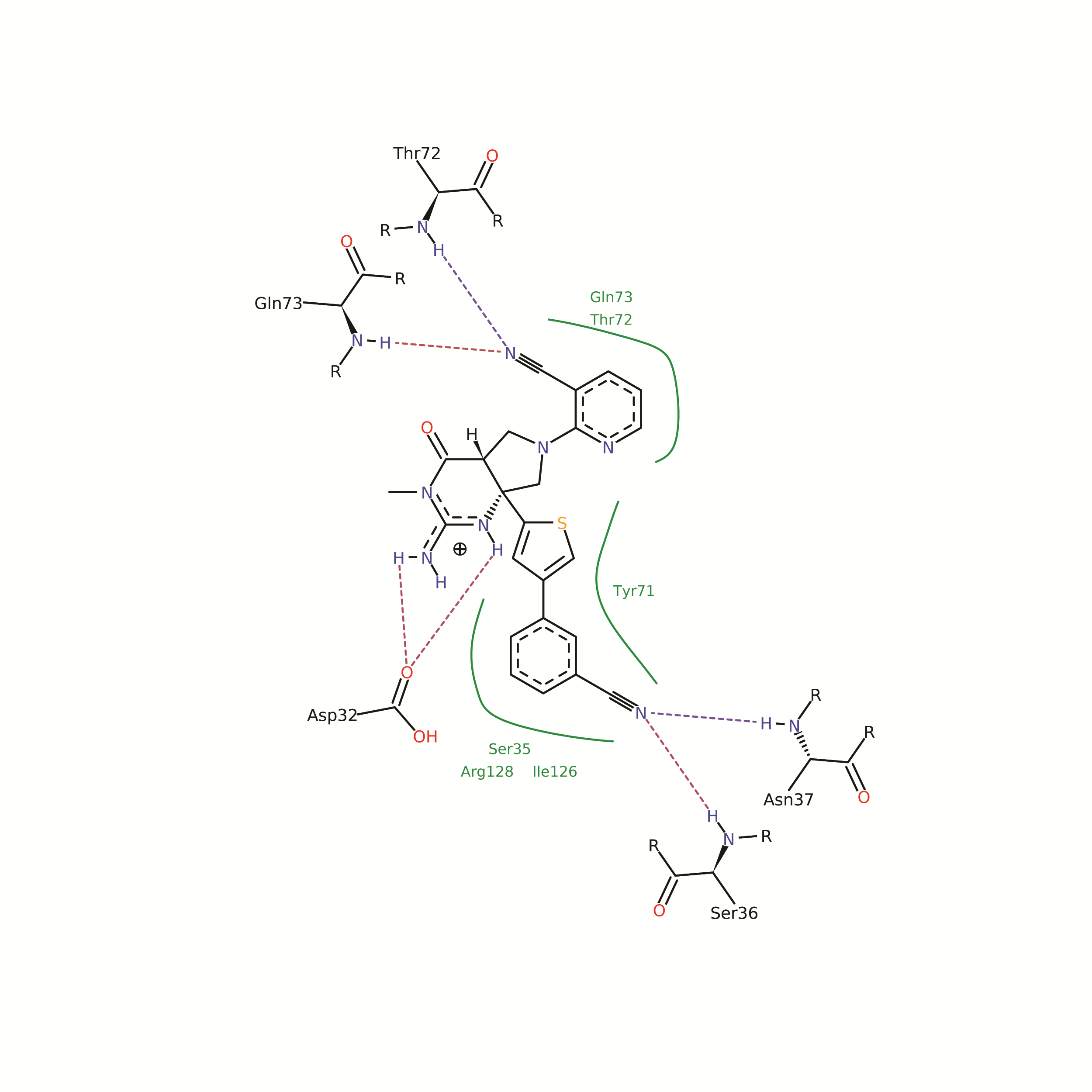 |
| 4DJX-0KQ | -24.28 | -68.11 | 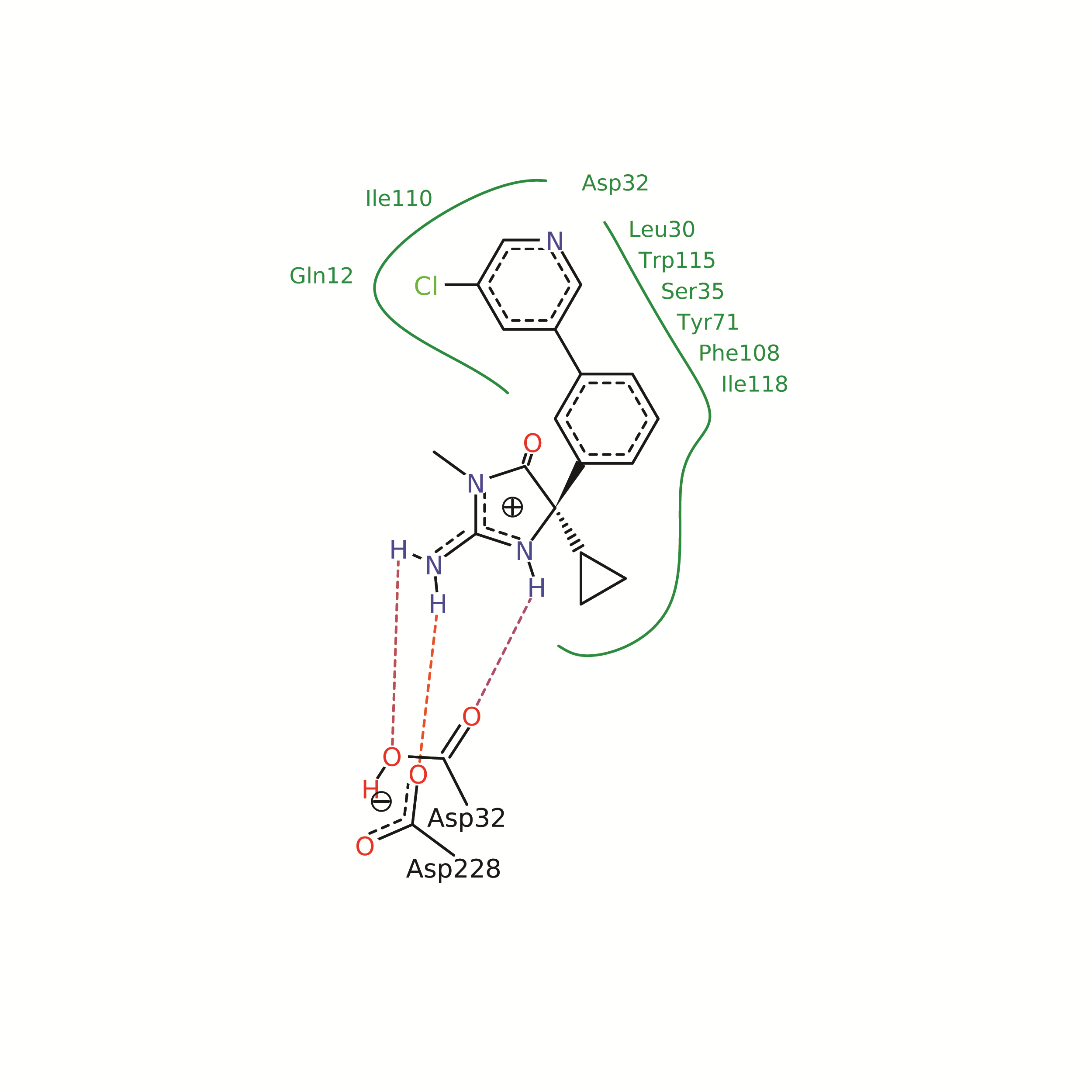 |
| 4DJX-13W | -22.16 | -54.74 | 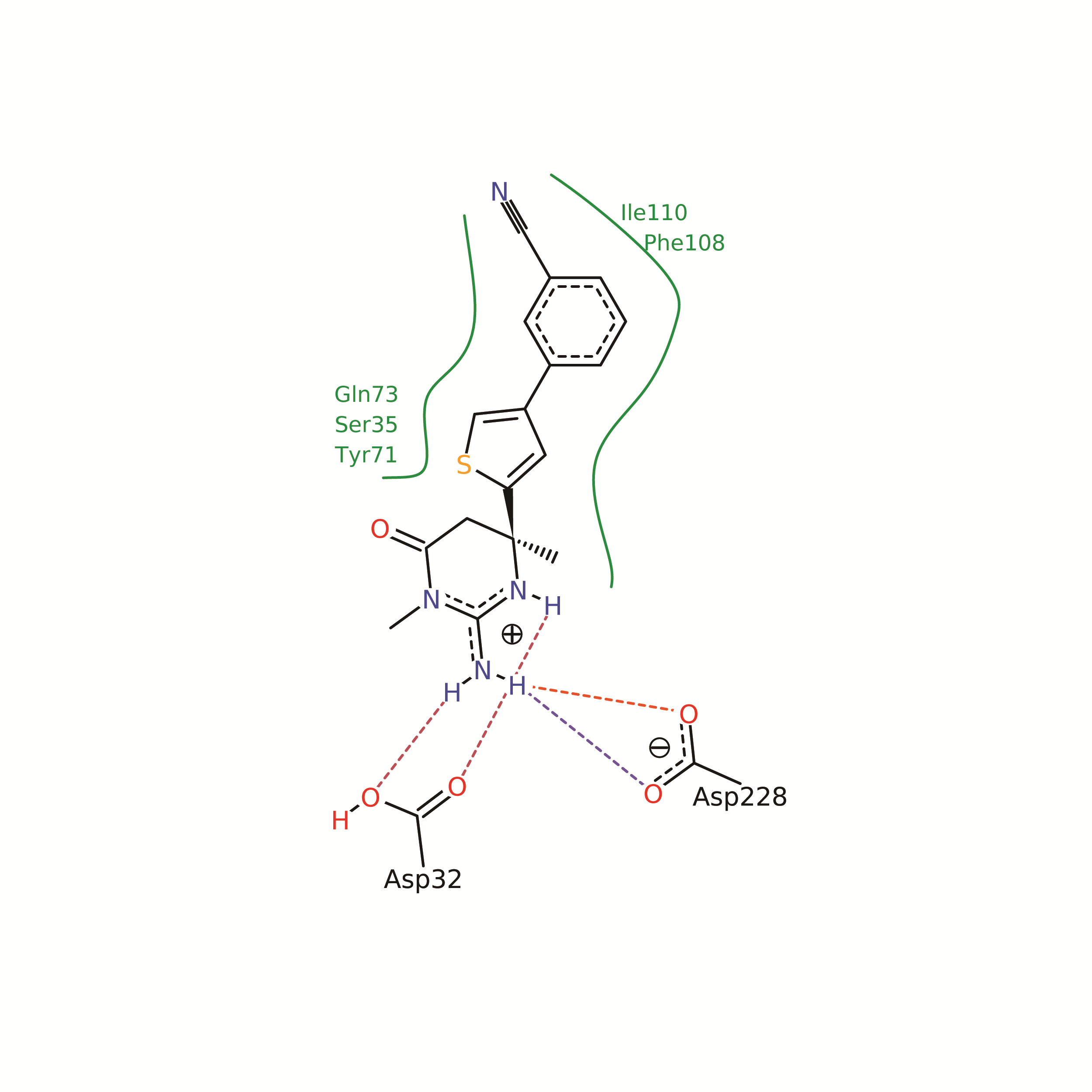 |
| 4DJX-H24 | -19.38 | -25.21 | 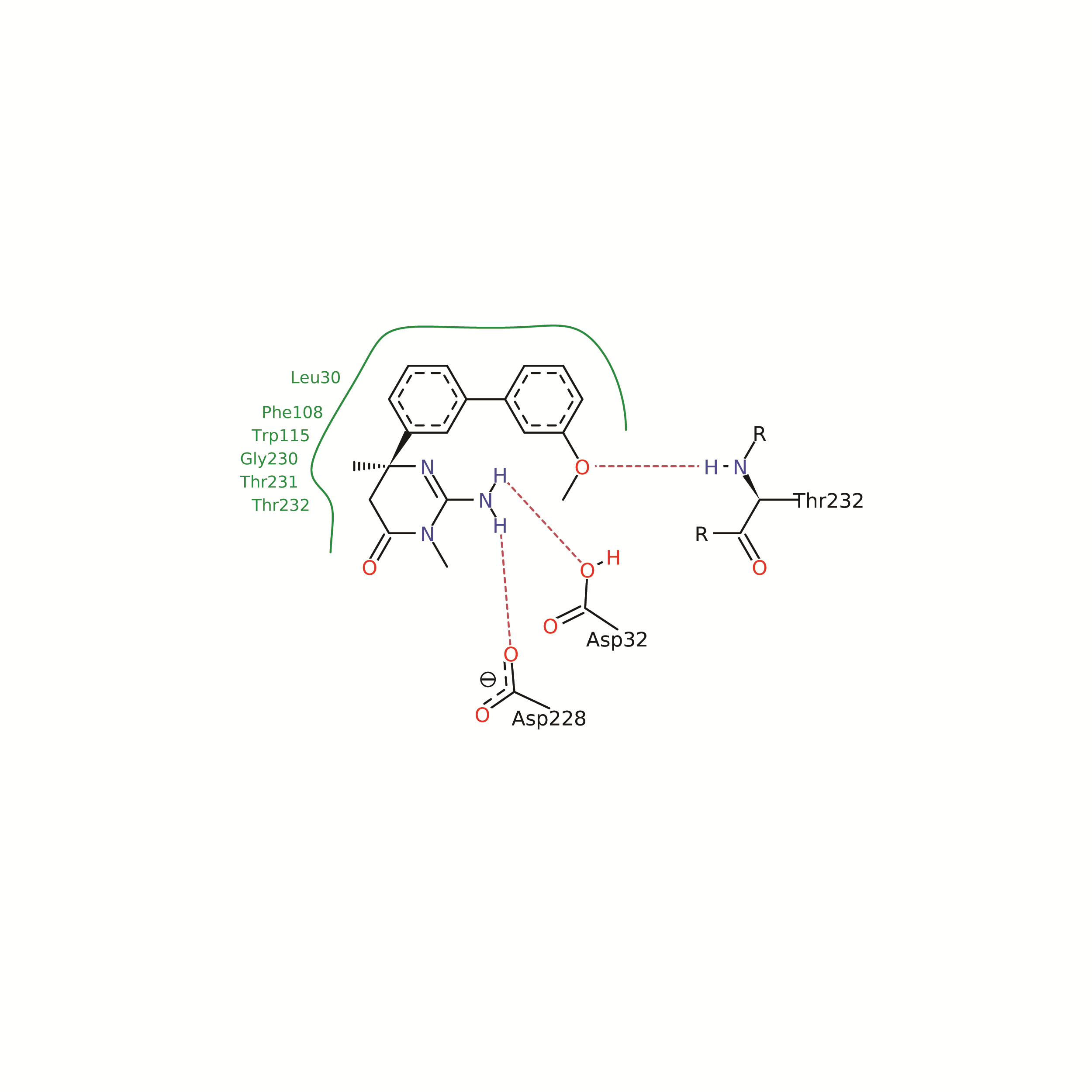 |
| **Cross-docking studies with 4HA5 as receptor using parameter 3** | | | |
| 4HA5-23I | -36.84 | -58.4 | 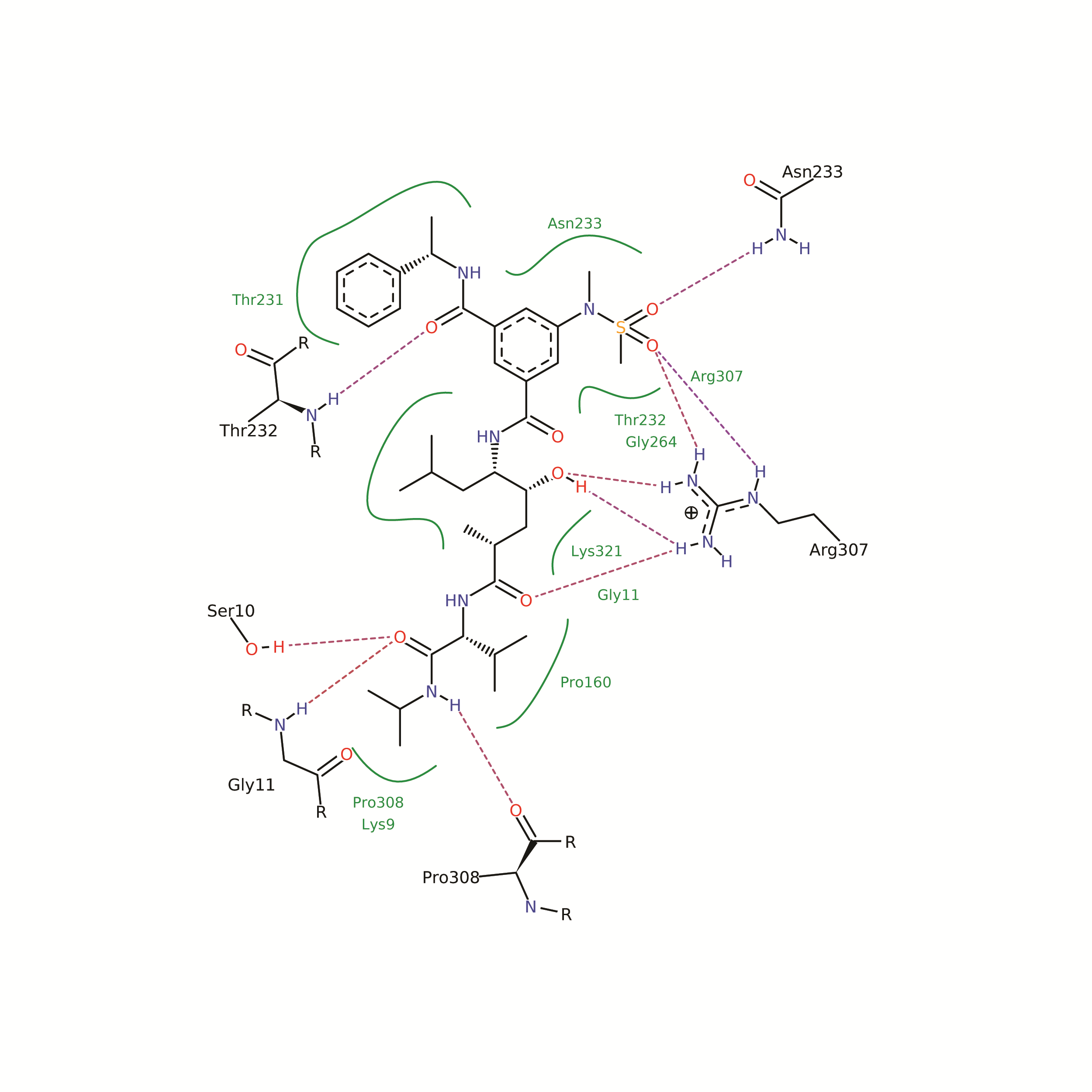 |
| 4HA5-SC6 | -38.43 | -90.8 | 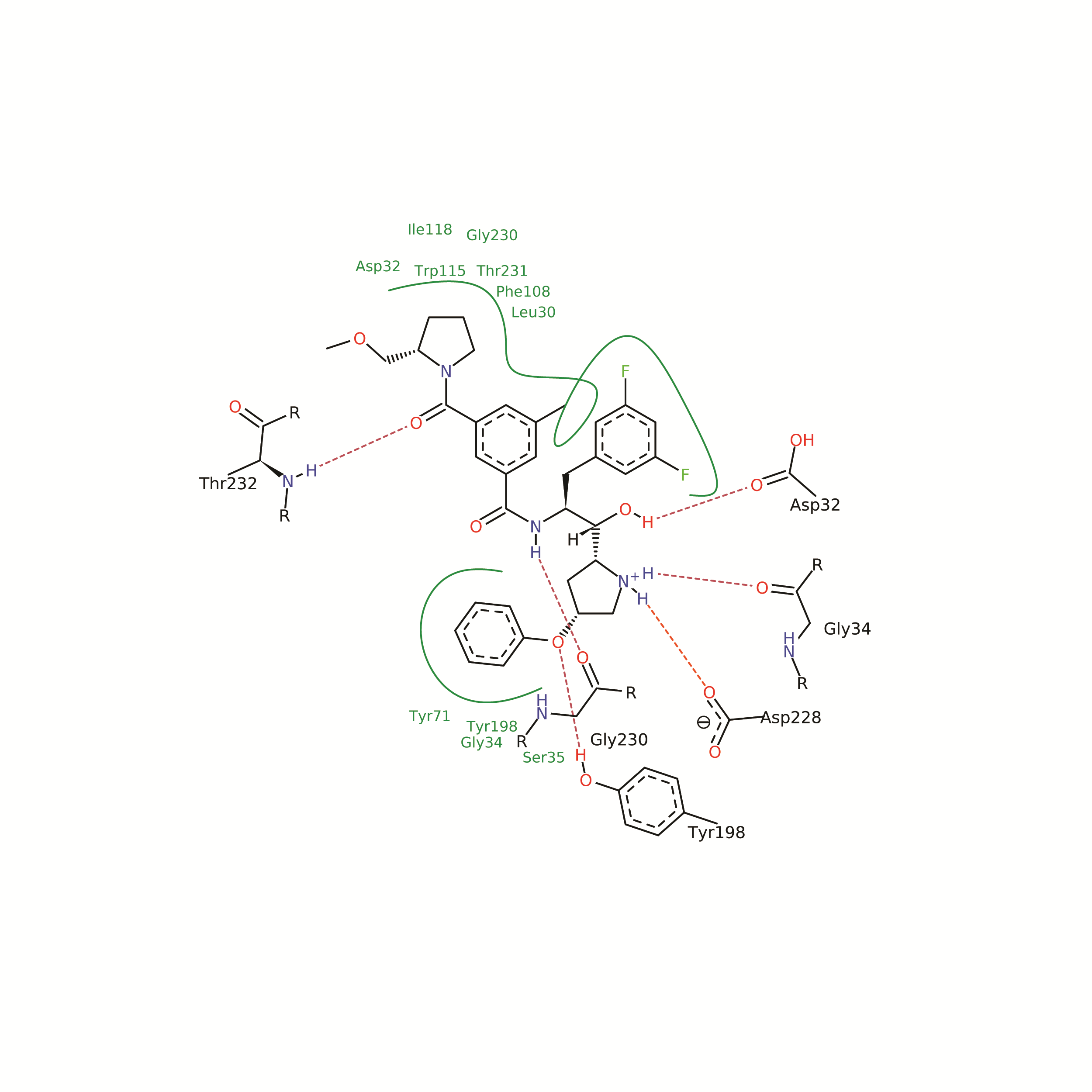 |
| 4HA5-Z76 | -34.27 | -64.77 | 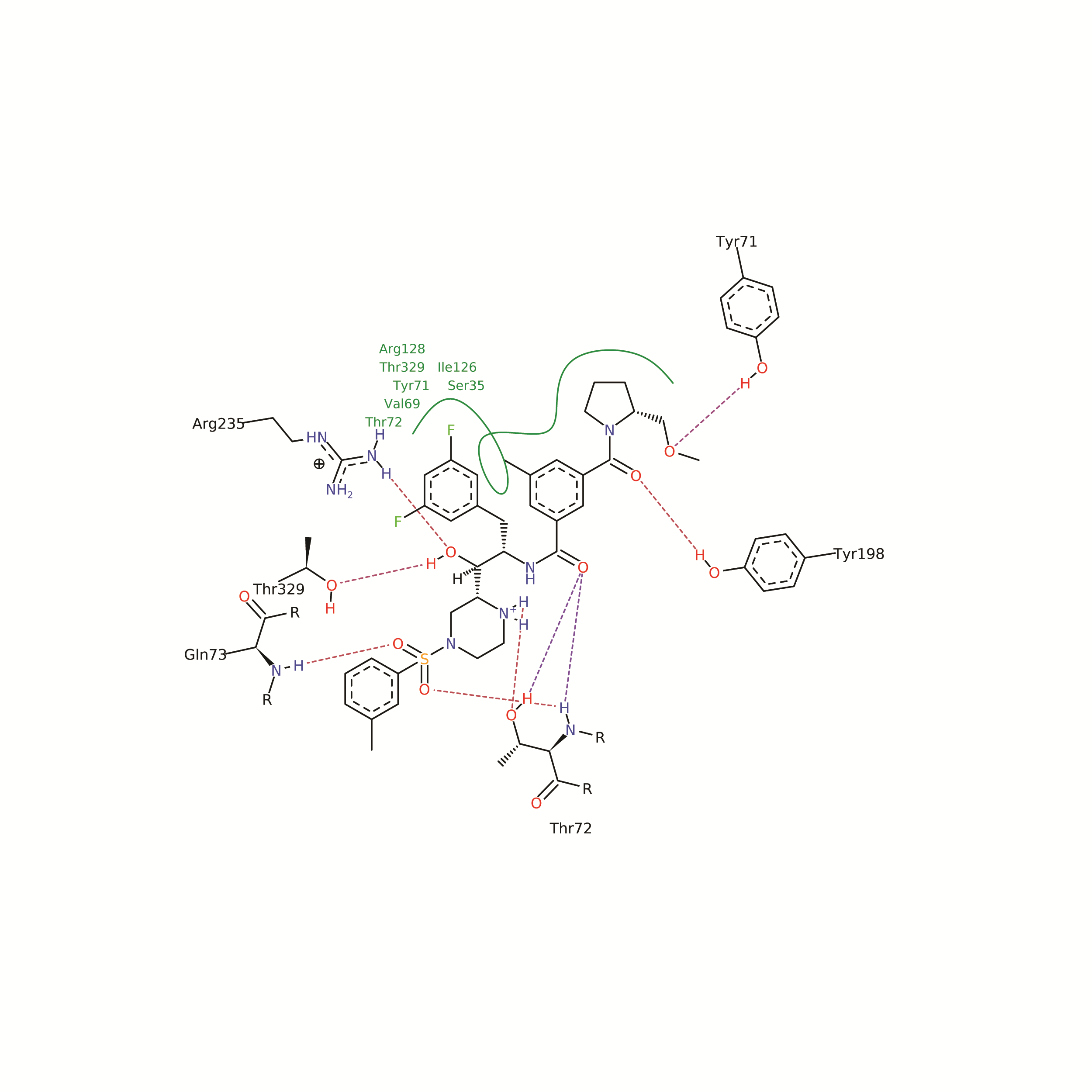 |
| 4HA5-316 | -34 | -78.69 | 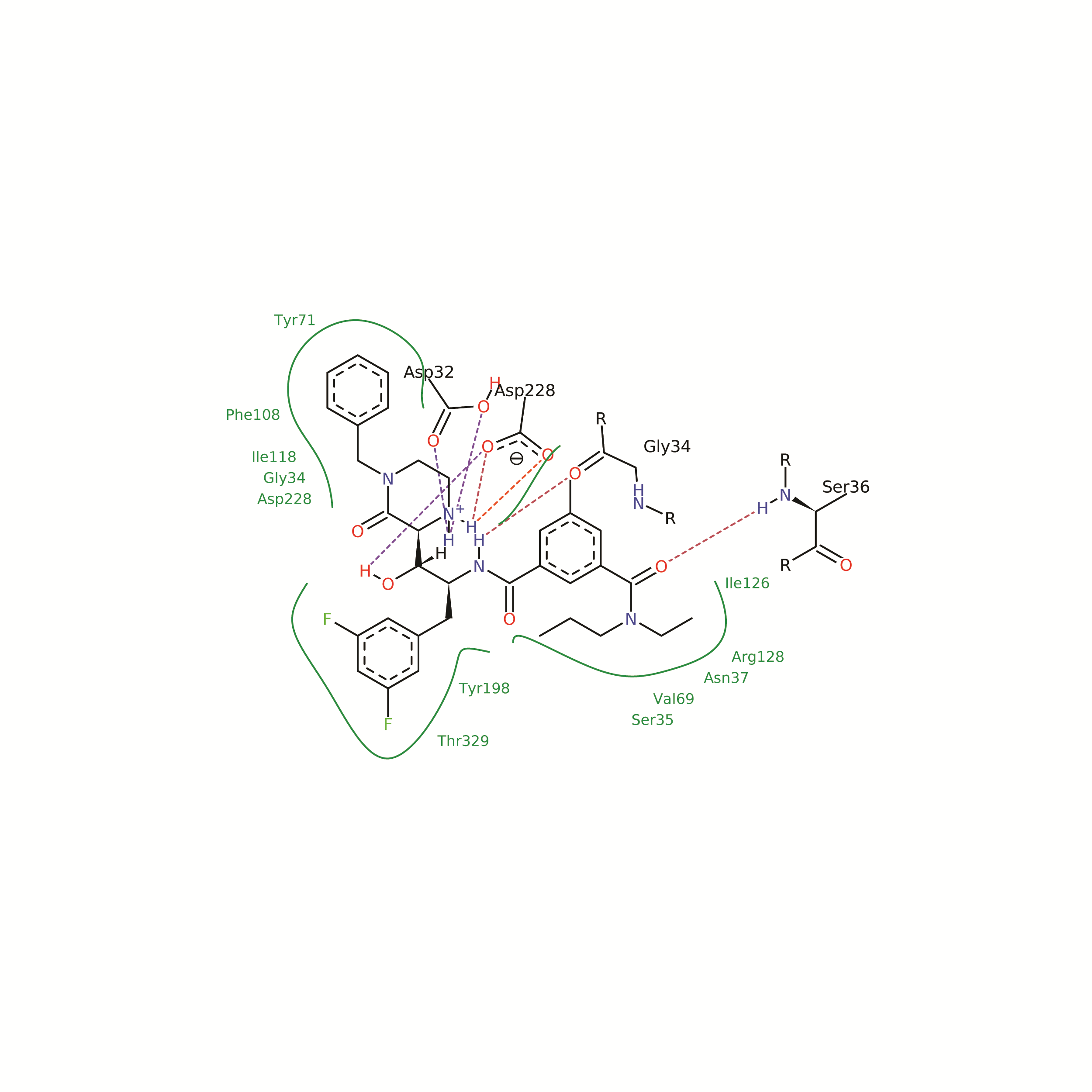 |
| 4HA5-10Q | -27.03 | -88.46 | 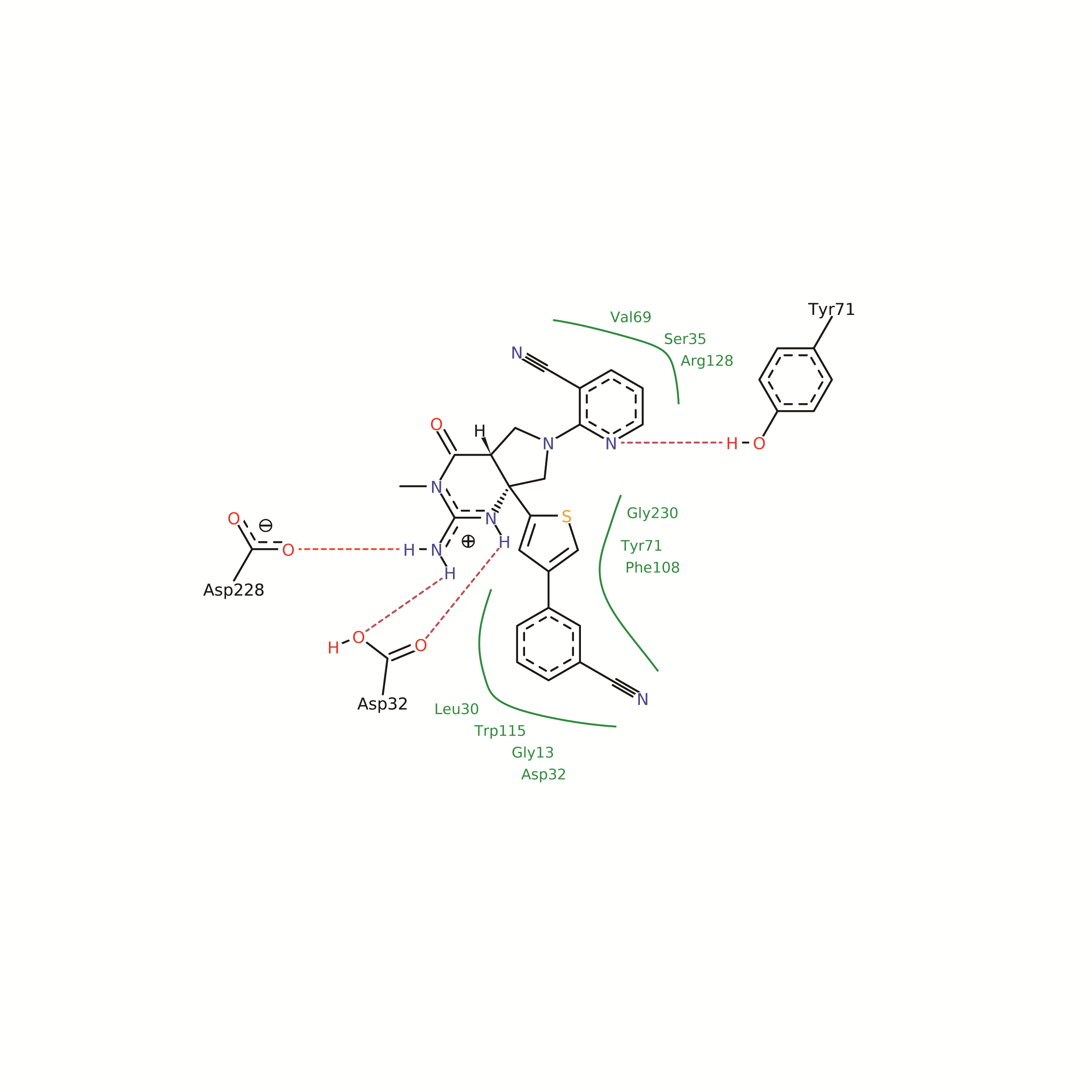 |
| 4HA5-0KQ | -25.89 | -54.4 | 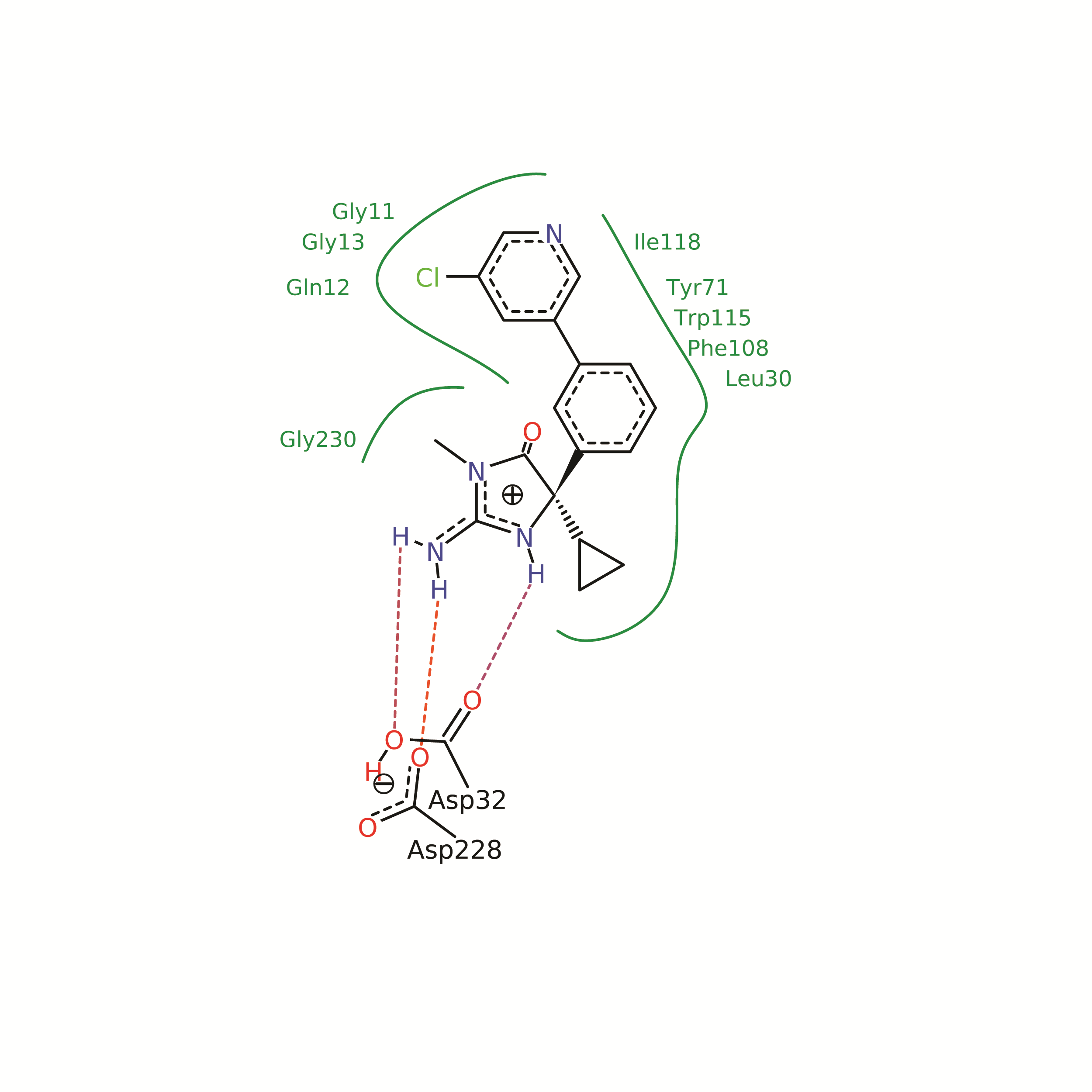 |
| 4HA5-13W | -25.87 | -61.16 | 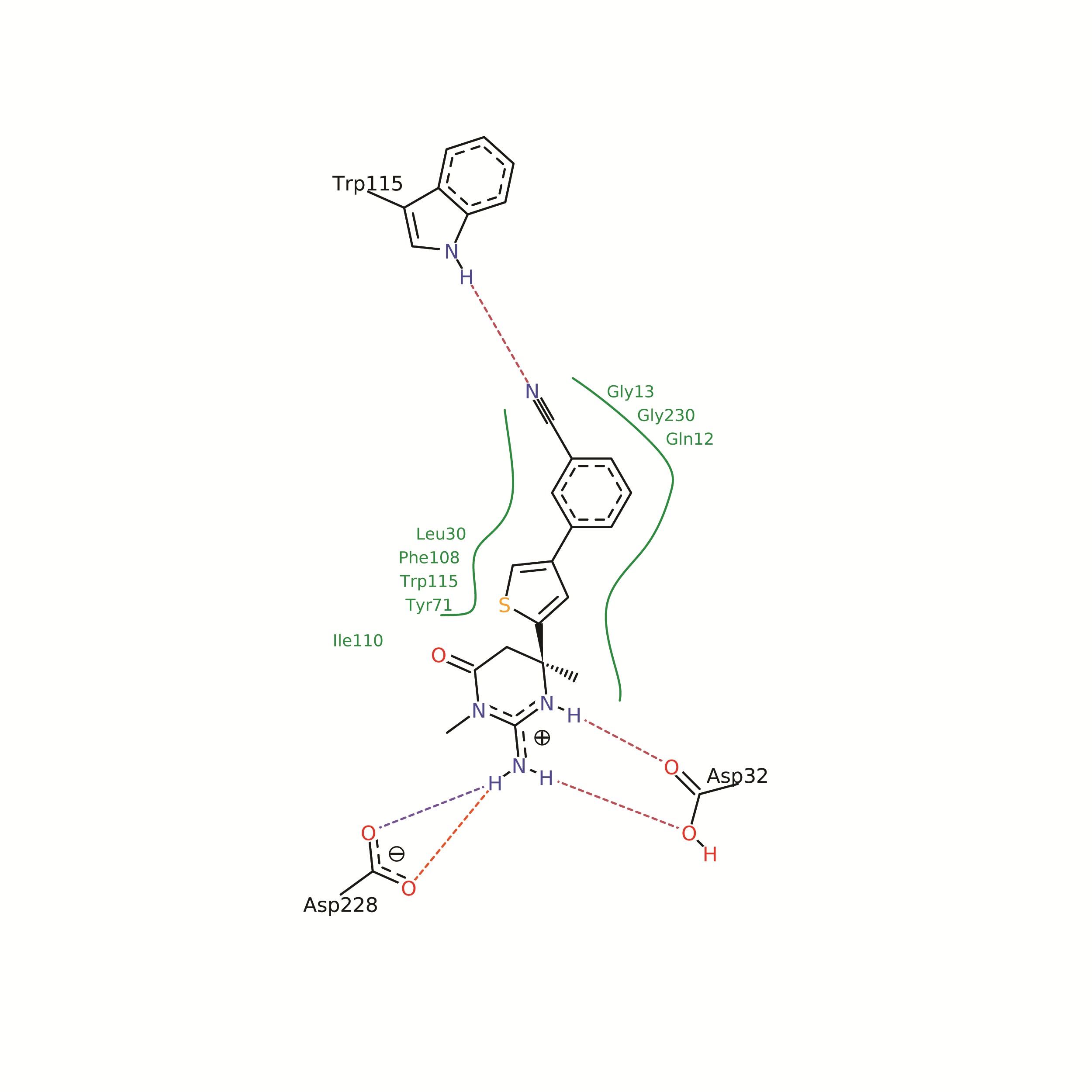 |
| 4HA5-H24 | -20.25 | -20.72 | 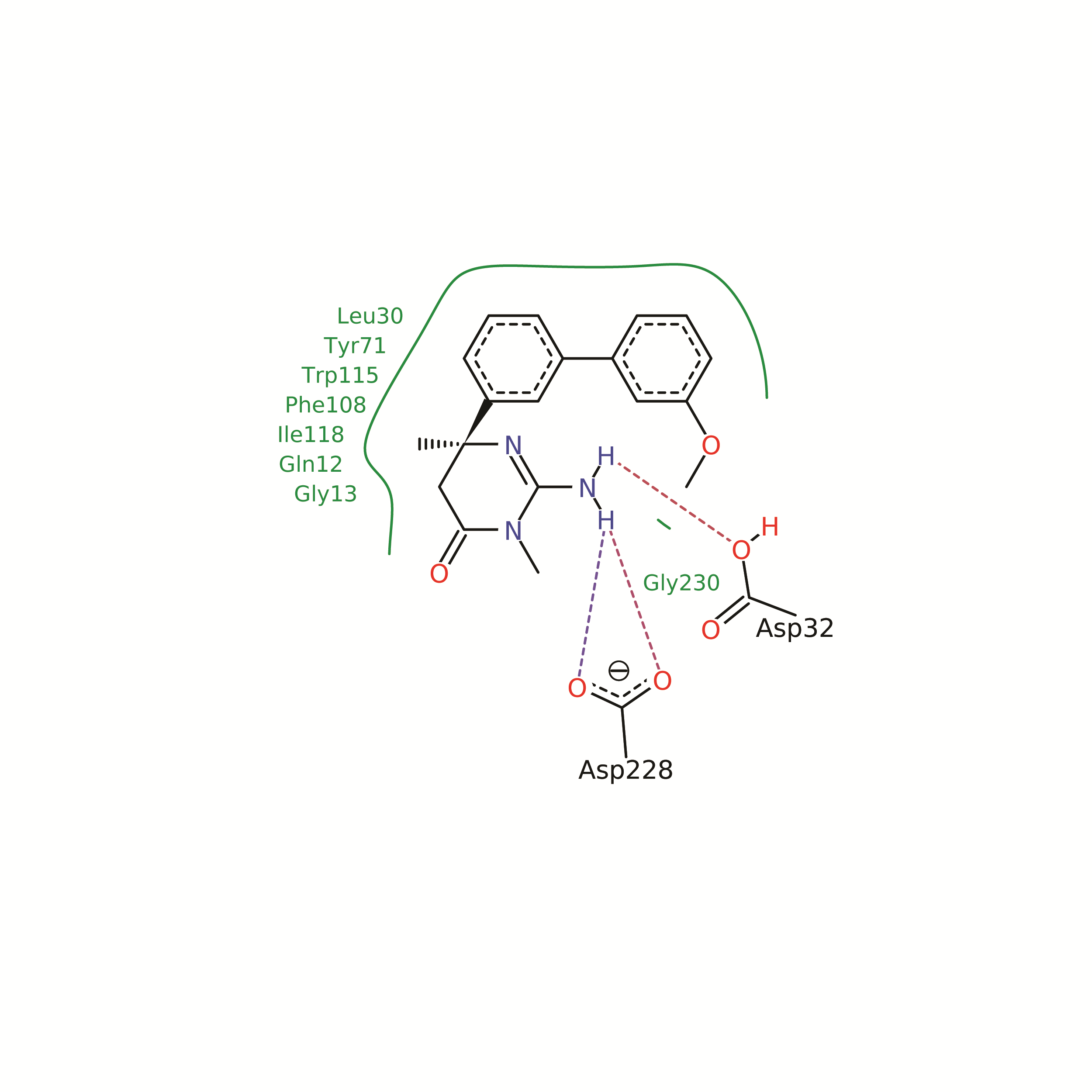 |
| **Cross-docking with 4HA5 as a receptor using parameter 2** | | | |
| 2P4J | -33.4 | -54.15 | 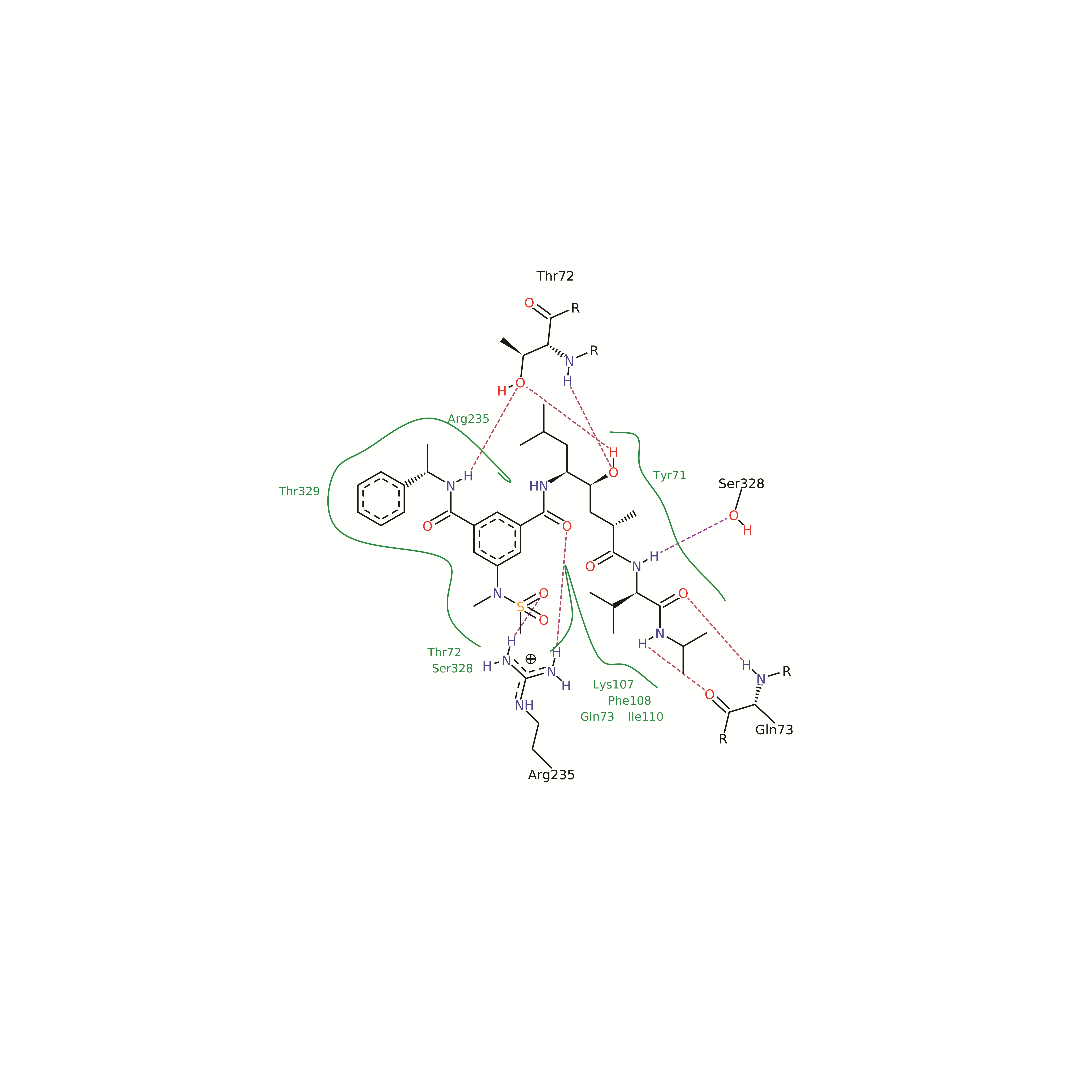 |
| 2QMG | -37.63 | -91.11 | 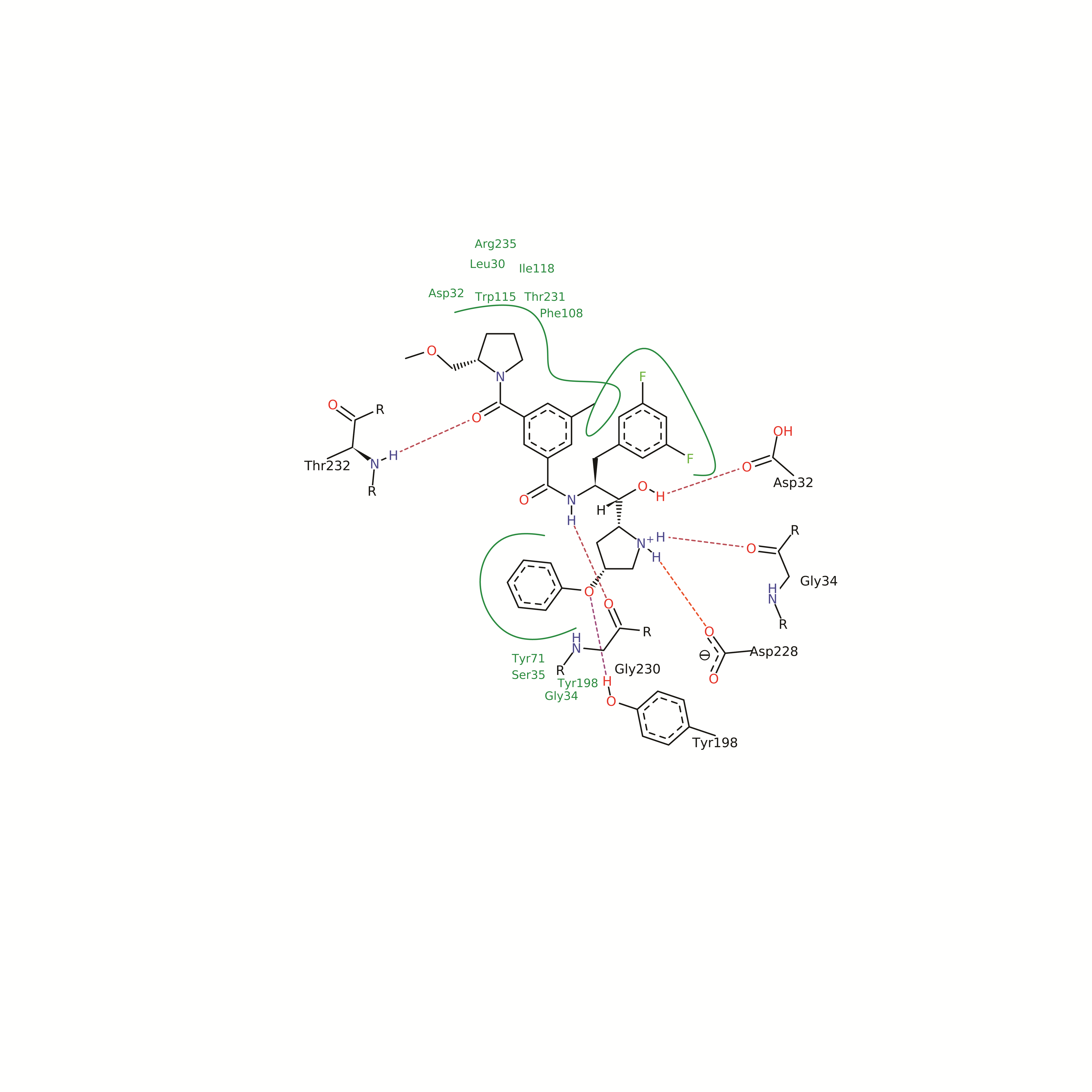 |
| 3LPK | 30.98 | -65.6 | 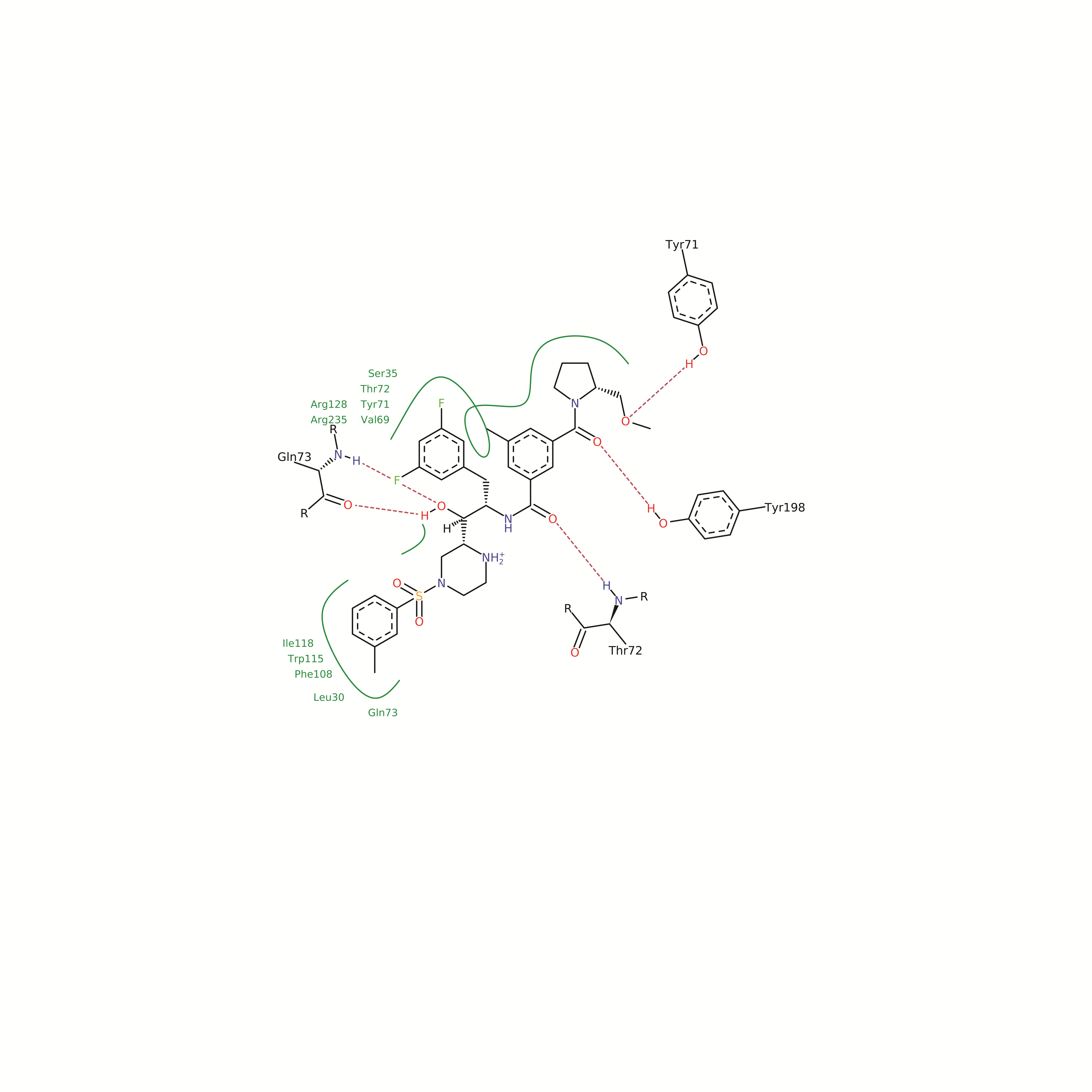 |
| 3CIC | -27.34 | -61.4 | 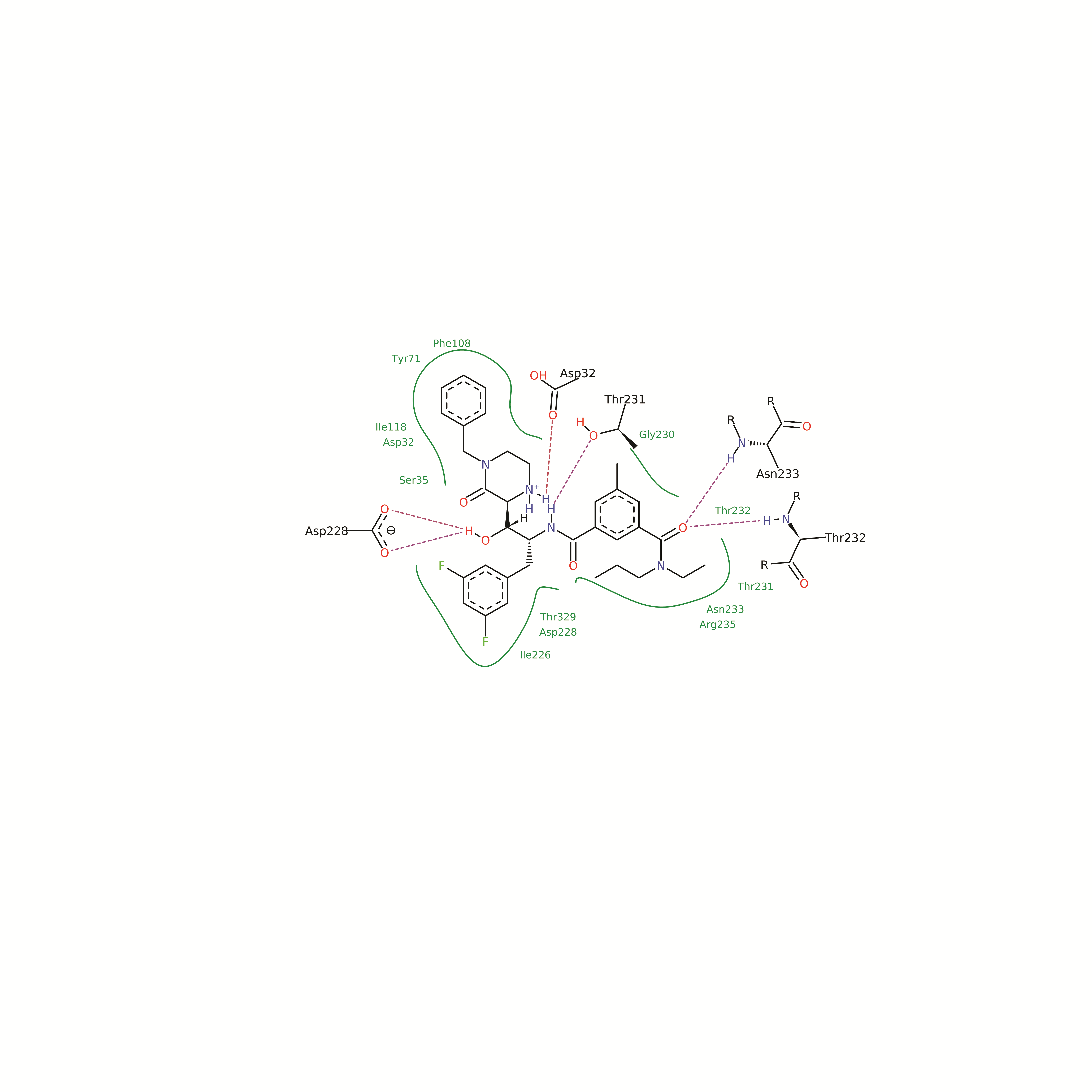 |
| 4H3G | -27.03 | -88.468 | 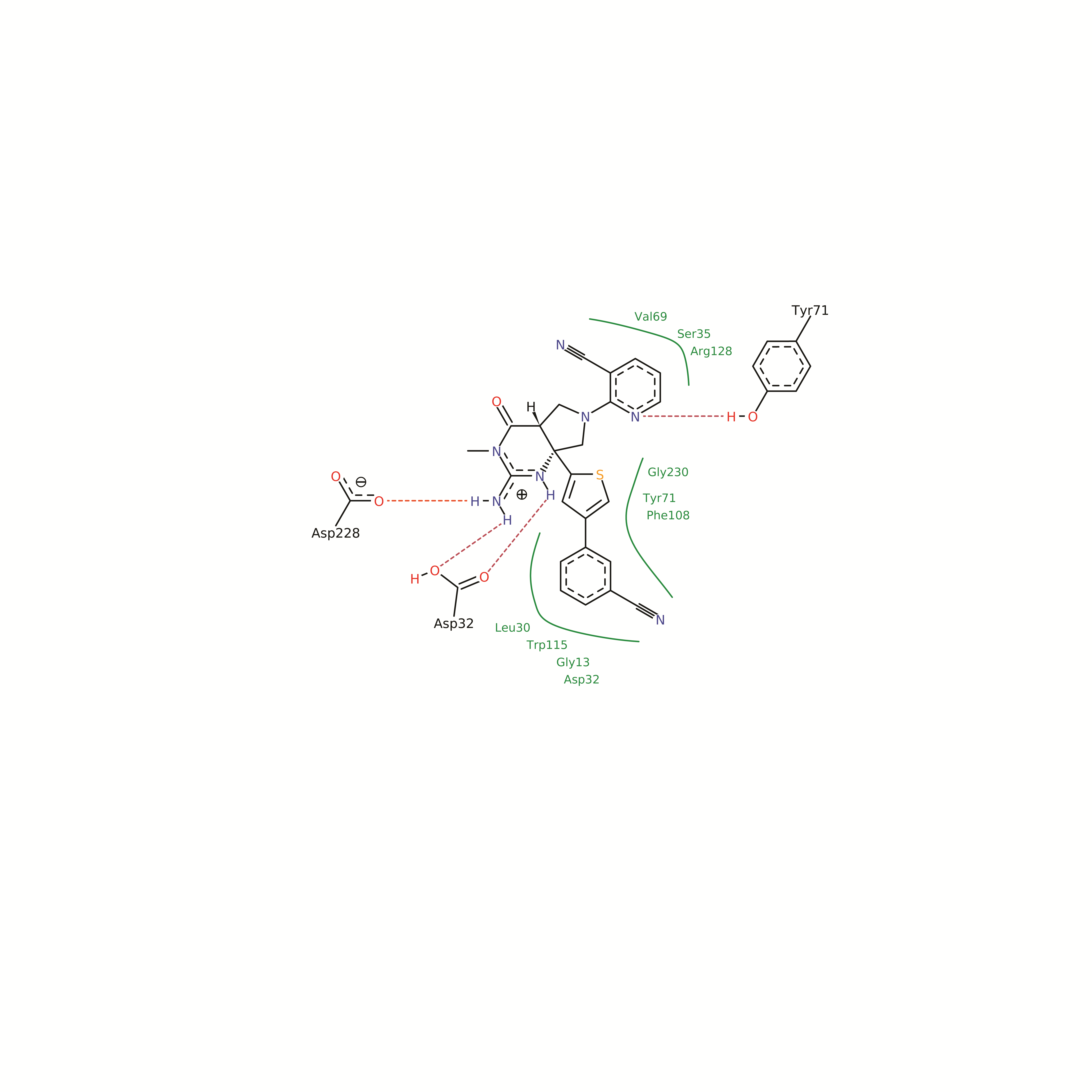 |
| 4DJX | -25.99 | -55.43 | 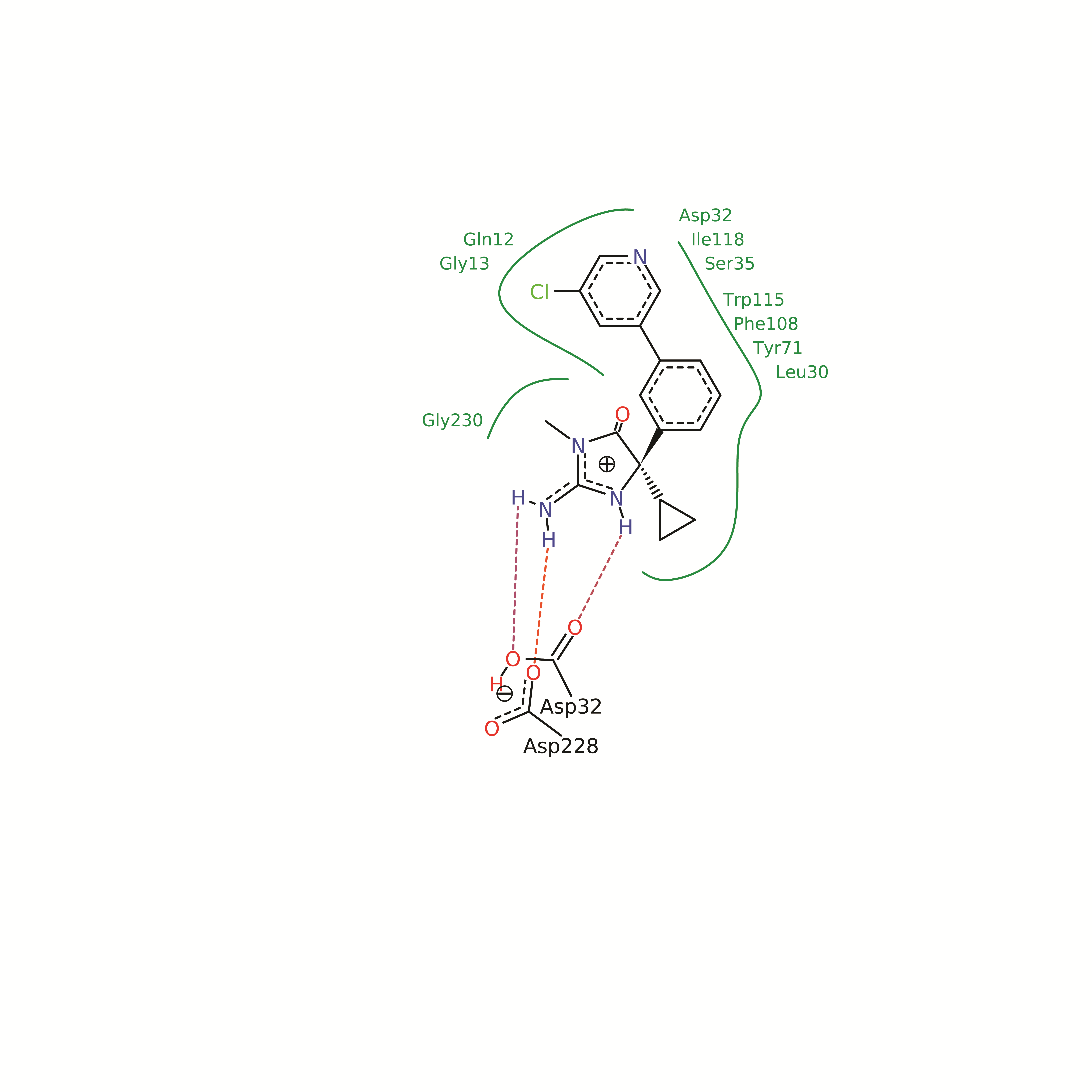 |
| 4HA5 | -22.62 | -53.4 | 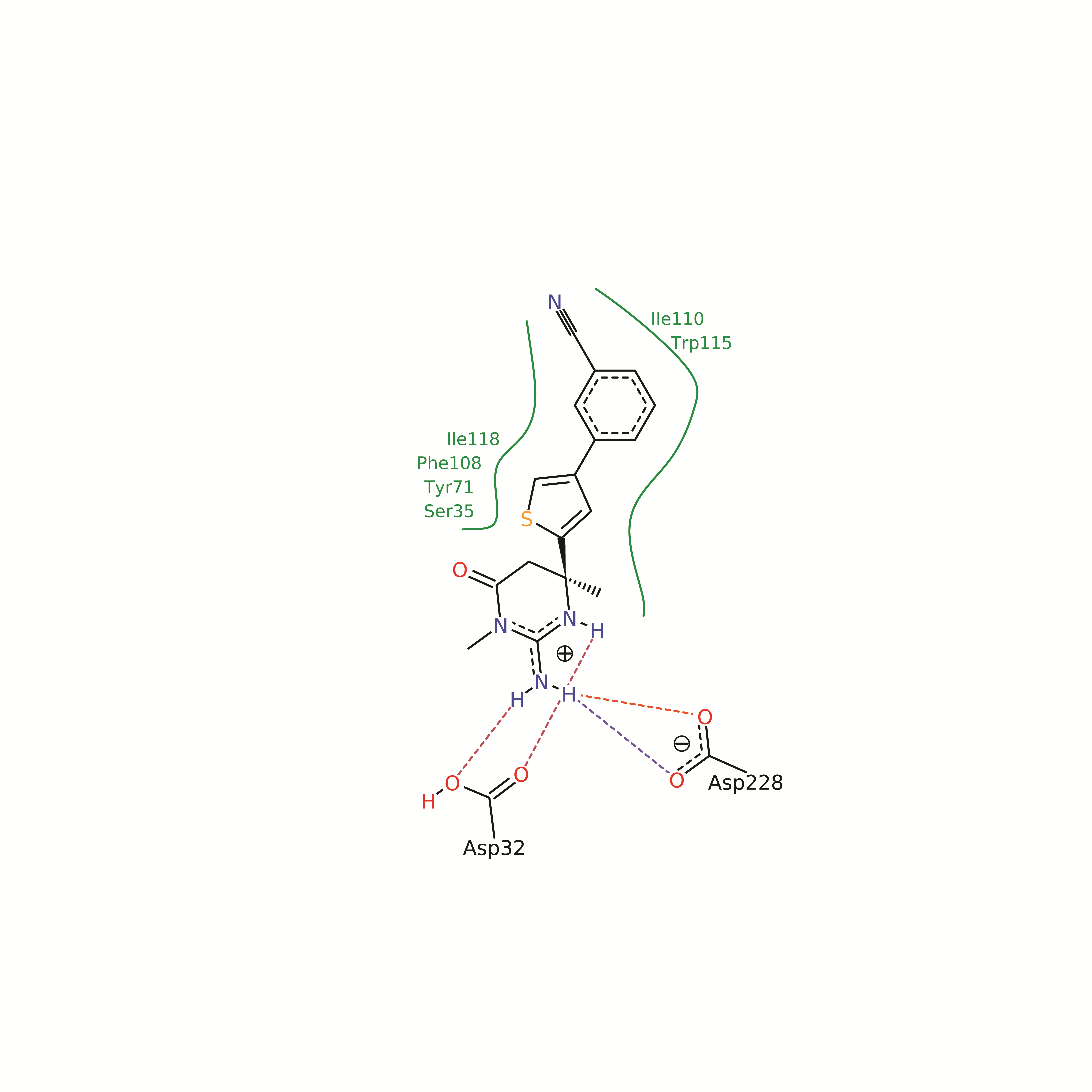 |
| 4FS4 | -22.69 | -24.23 | 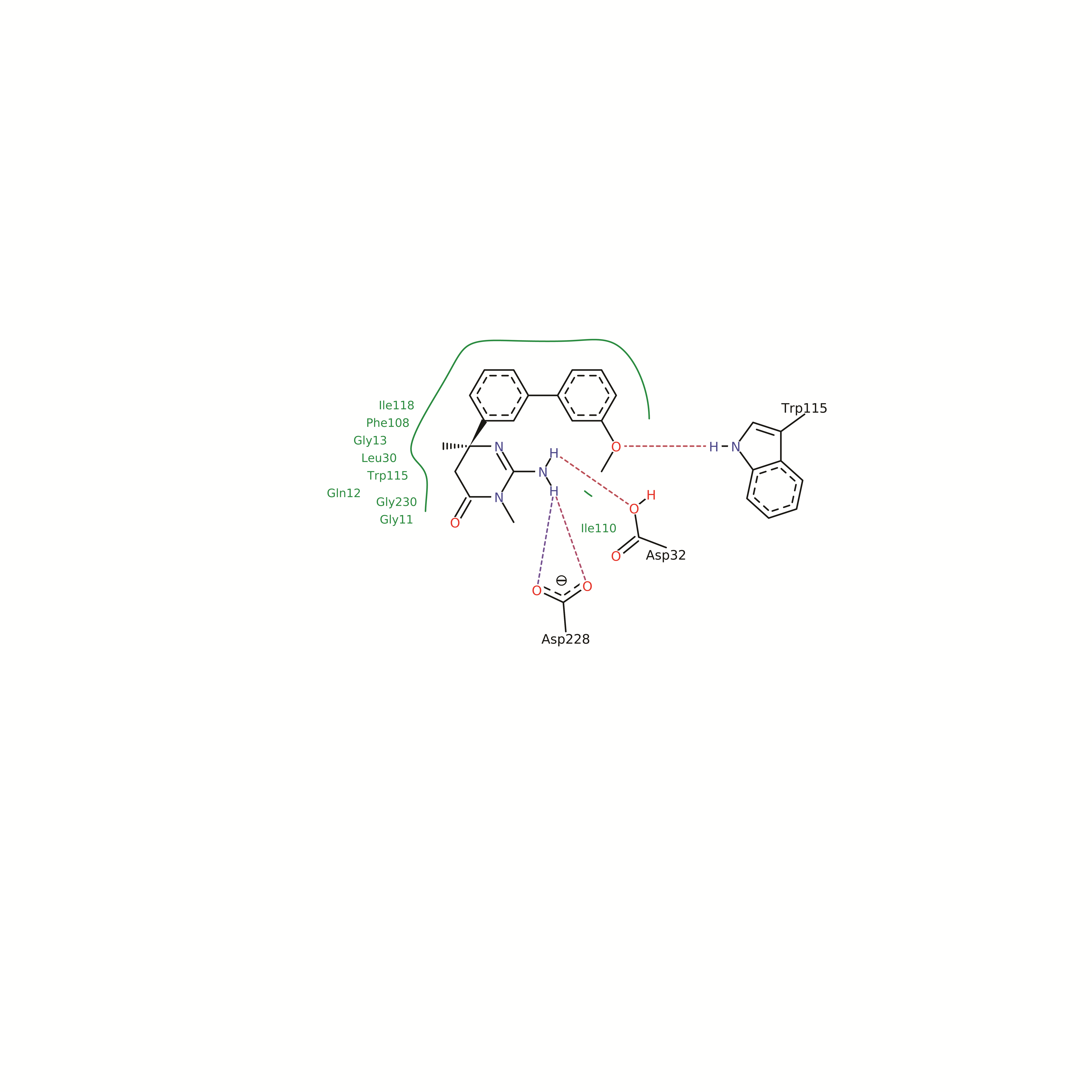 |
| **Cross-docking with 4HA5 as a receptor using parameter 1** | | | |
| 2P4J | -36.63 | -66.1 | 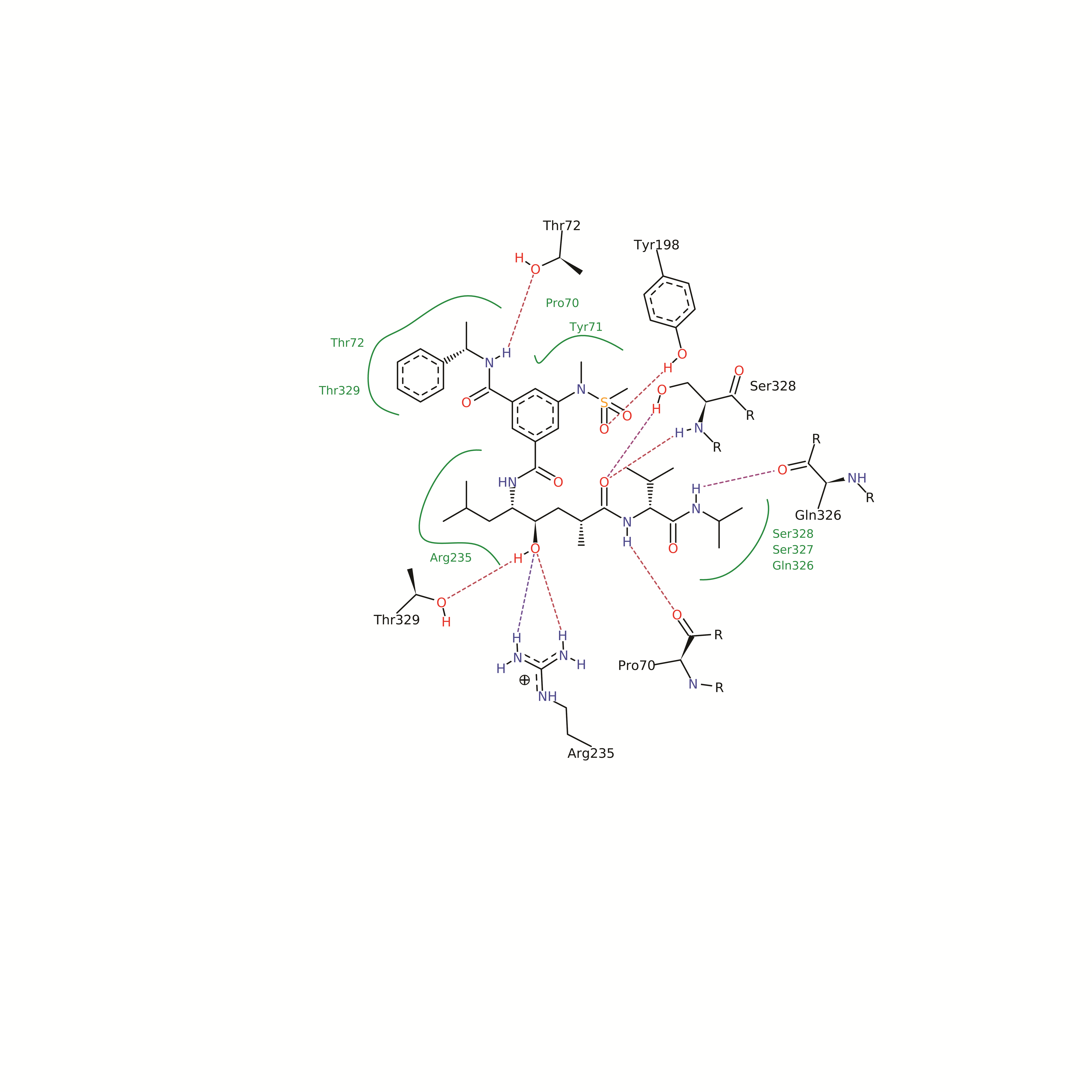 |
| 2QMG | -35.34 | -85.17 | 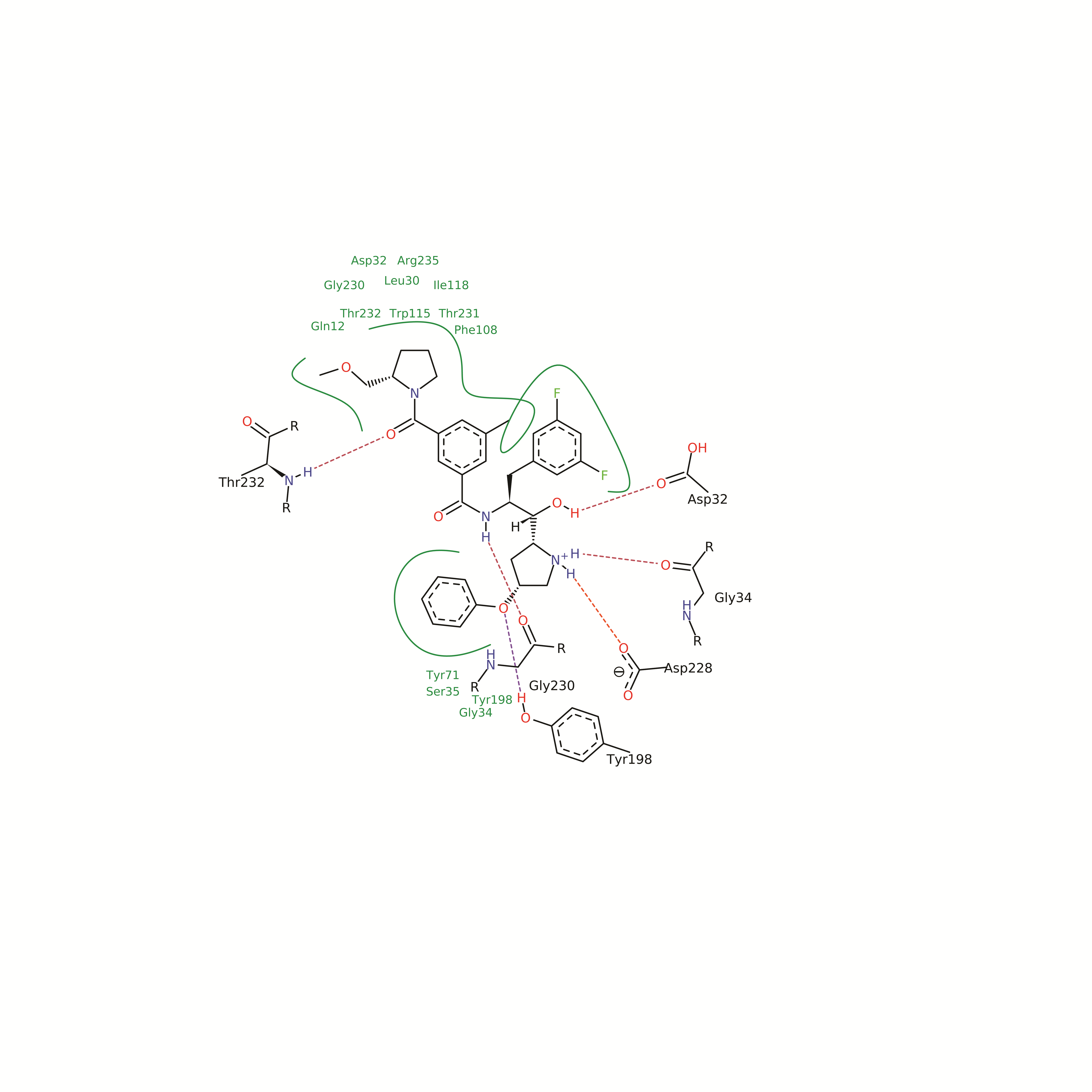 |
| 3LPK | 37.58 | -81.02 | 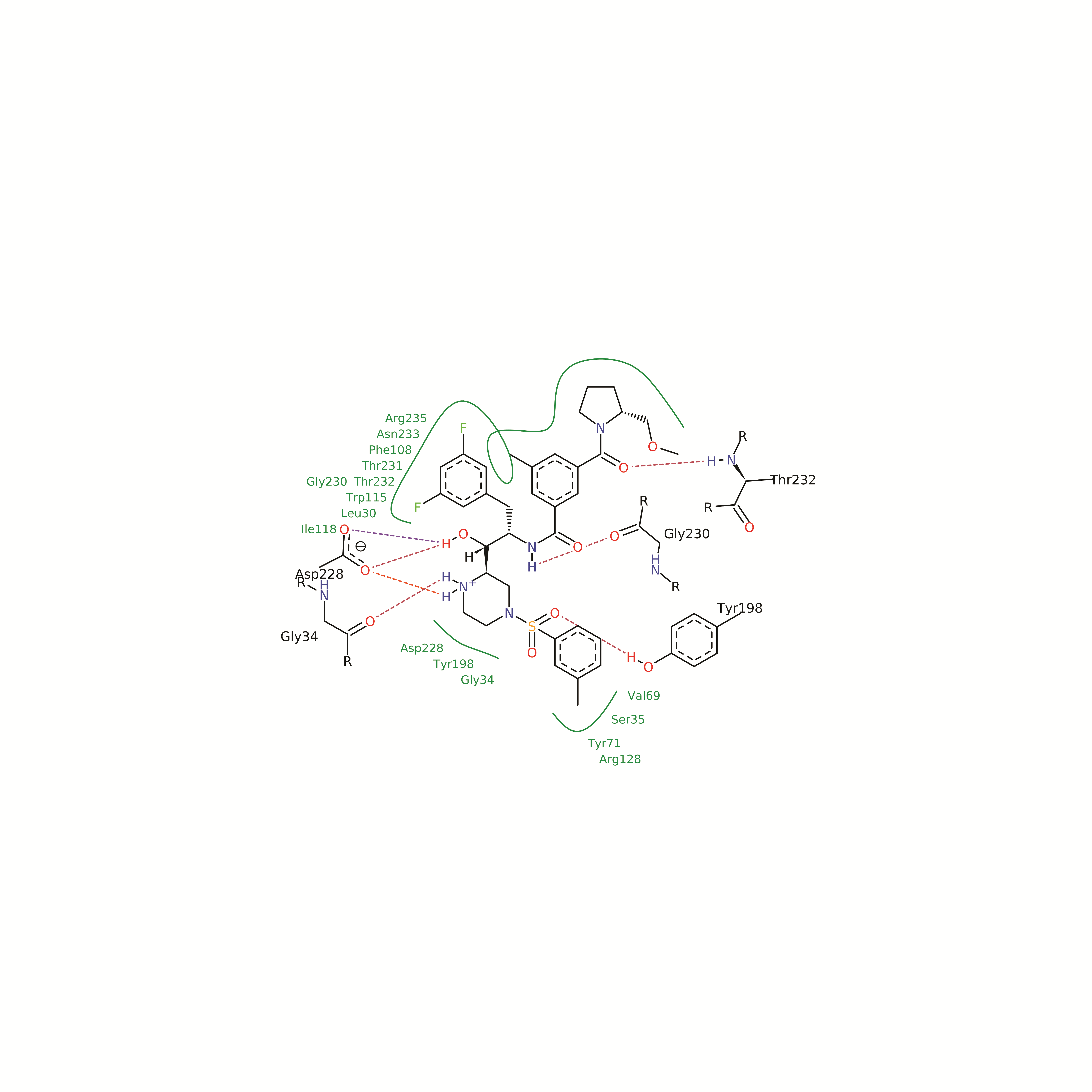 |
| 3CIC | -28.07 | -54.28 | 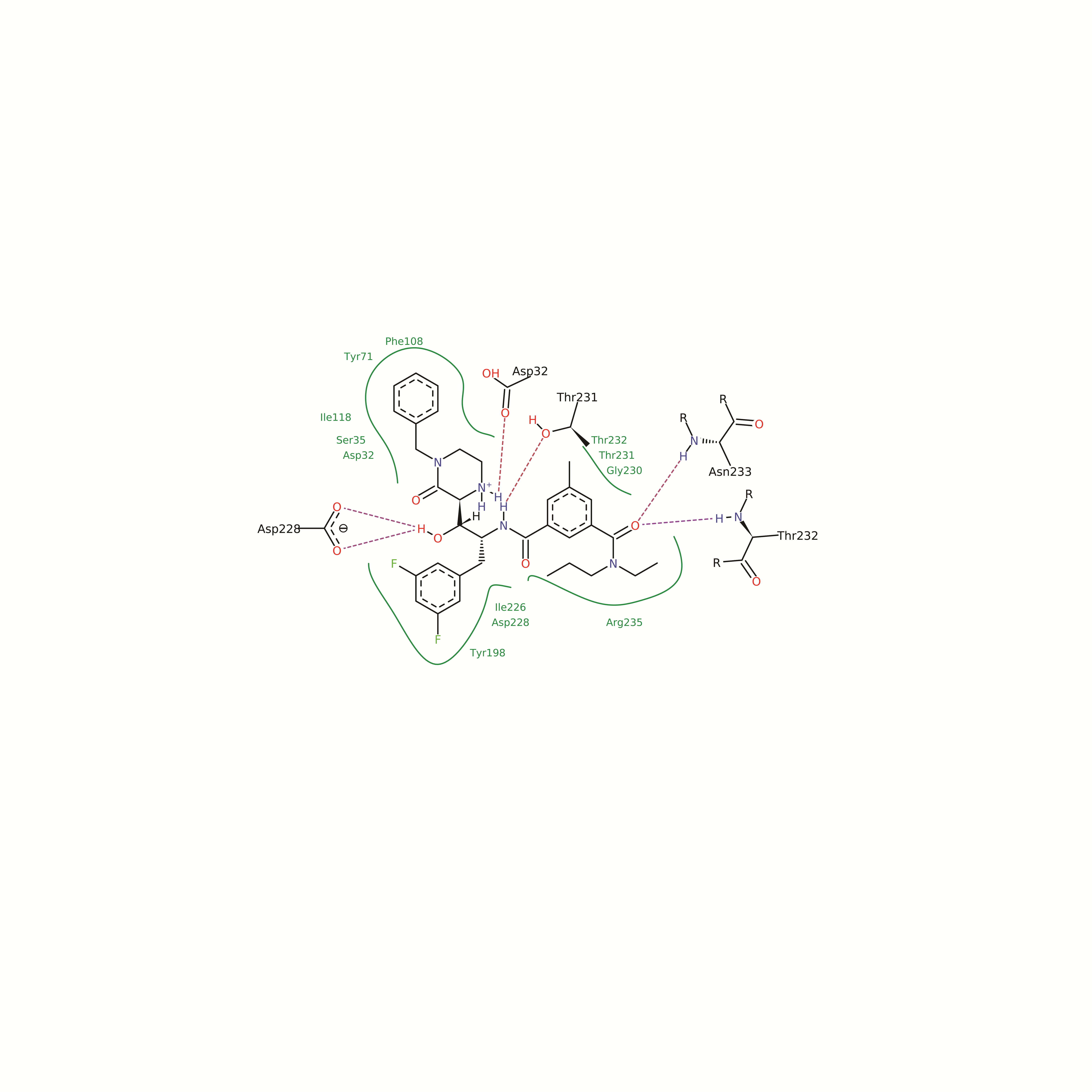 |
| 4H3G | -27.03 | -88.468 | 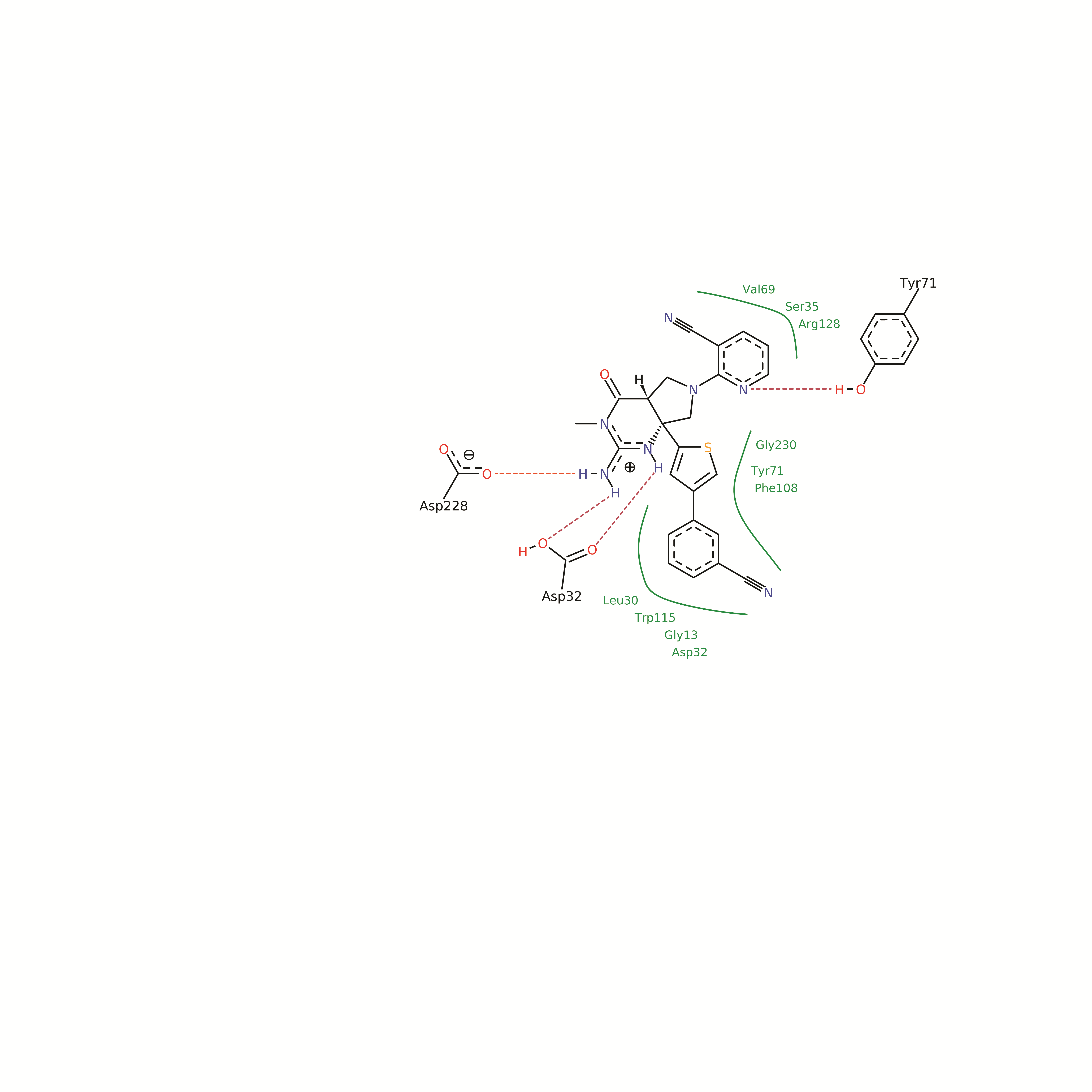 |
| 4DJX | -25.99 | -55.43 | 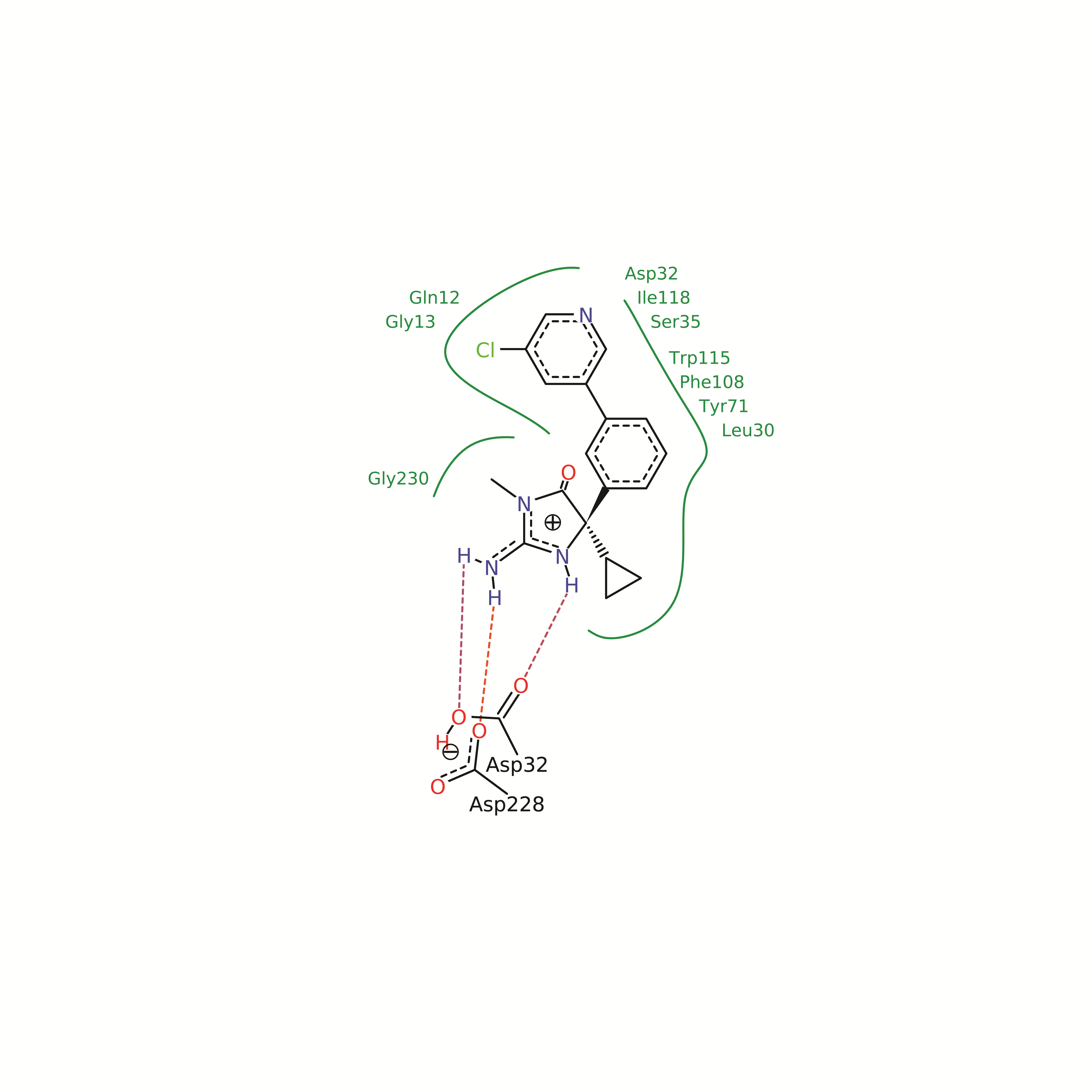 |
| 4HA5 | -25.87 | -61.16 | 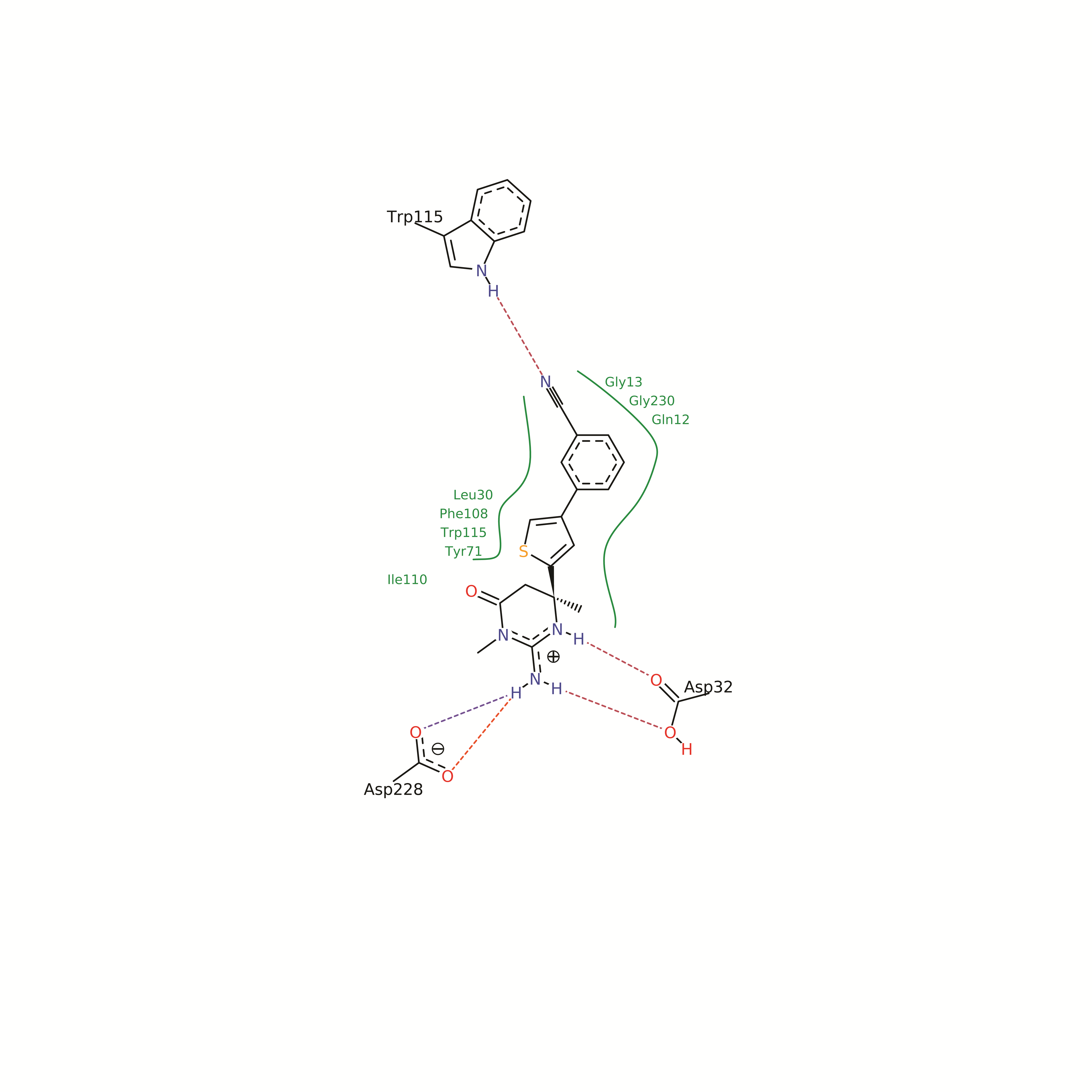 |
| 4FS4 | -19.89 | -55.43 | 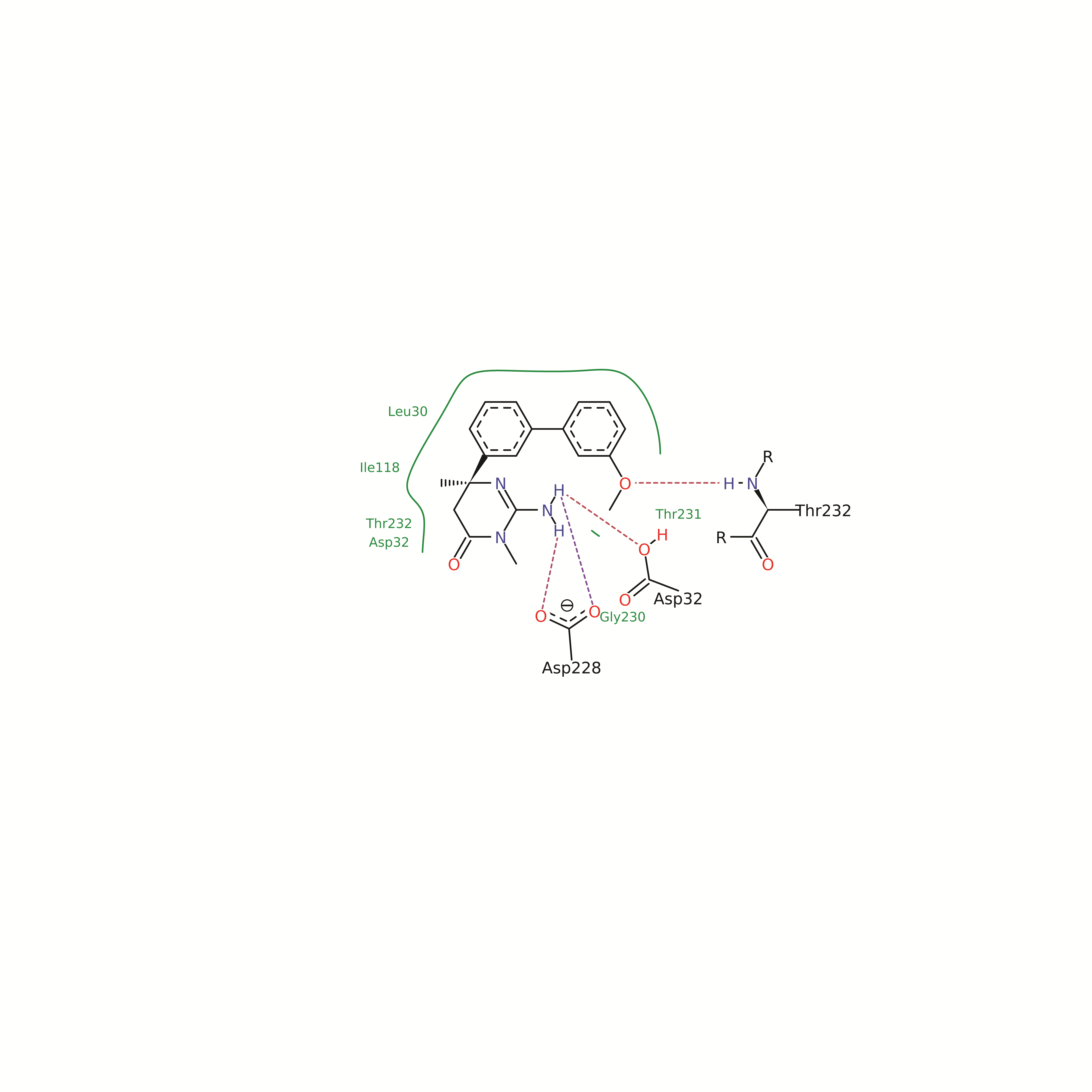 |
| **Cross-docking studies with 4FS4 as receptor using parameter 3** | | | |
| 4FS4-23I | -40.4 | -55.09 | 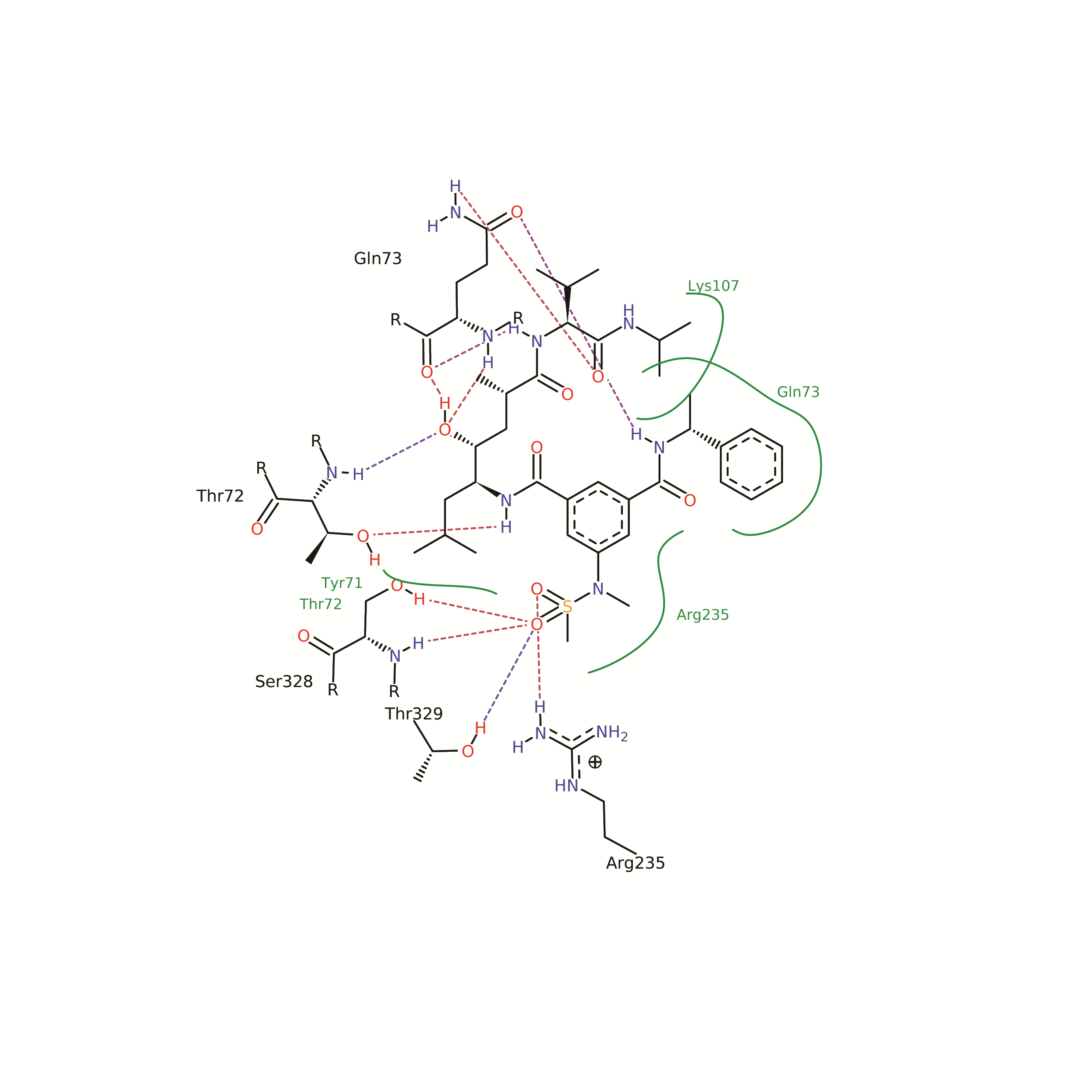 |
| 4FS4-SC6 | -28.57 | -58.95 | 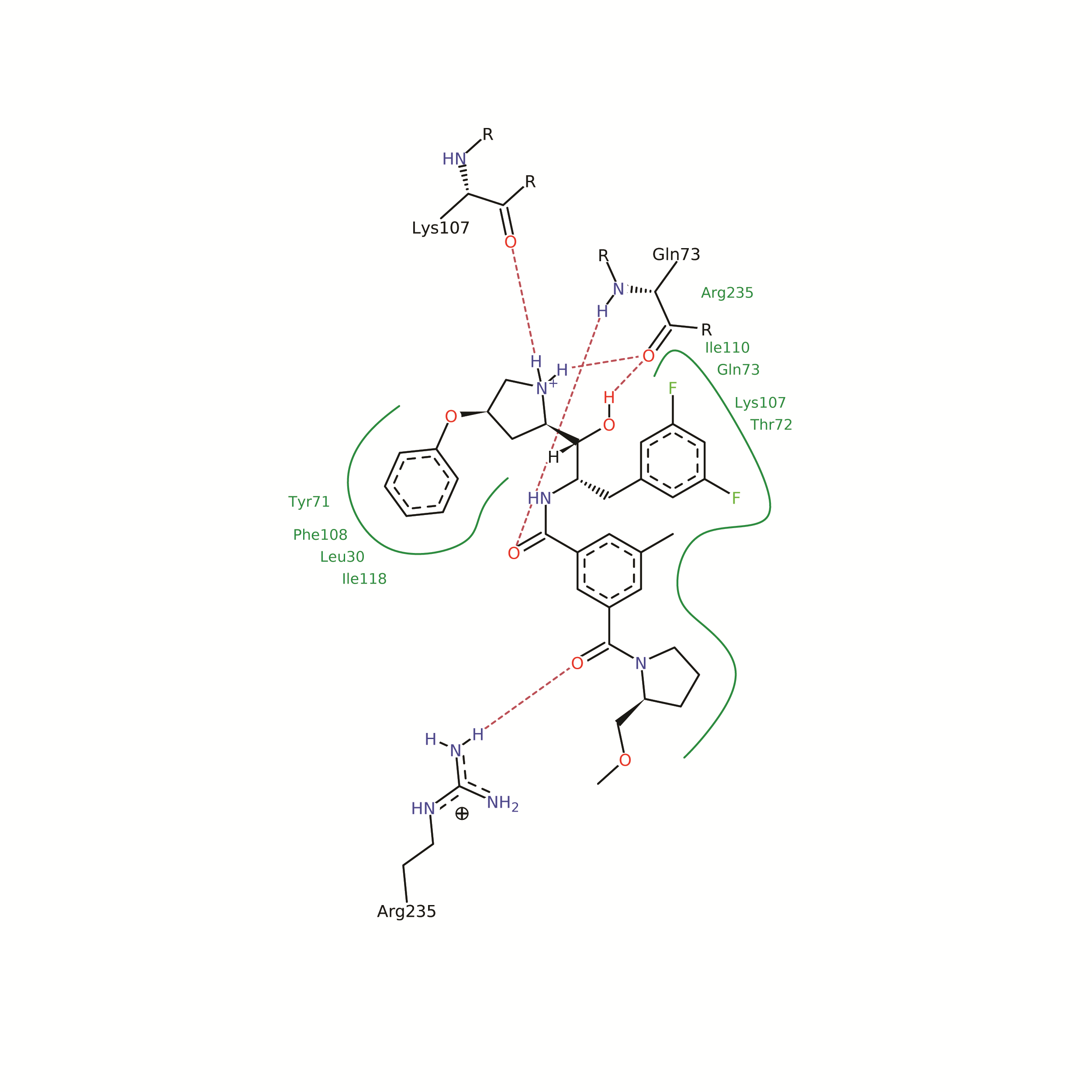 |
| 4FS4-Z76 | -39.56 | -84.79 | 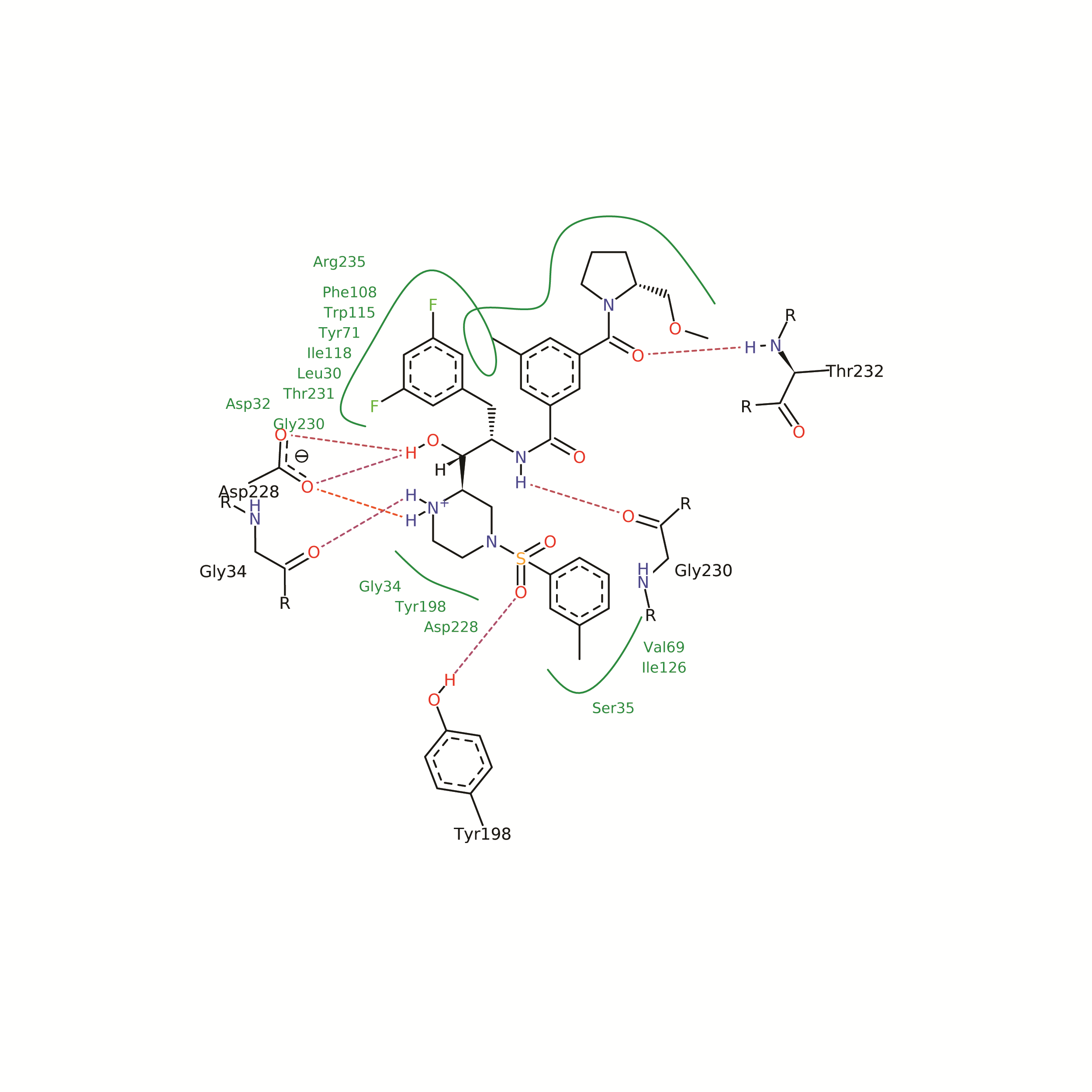 |
| 4FS4-316 | -33.42 | -72.79 | 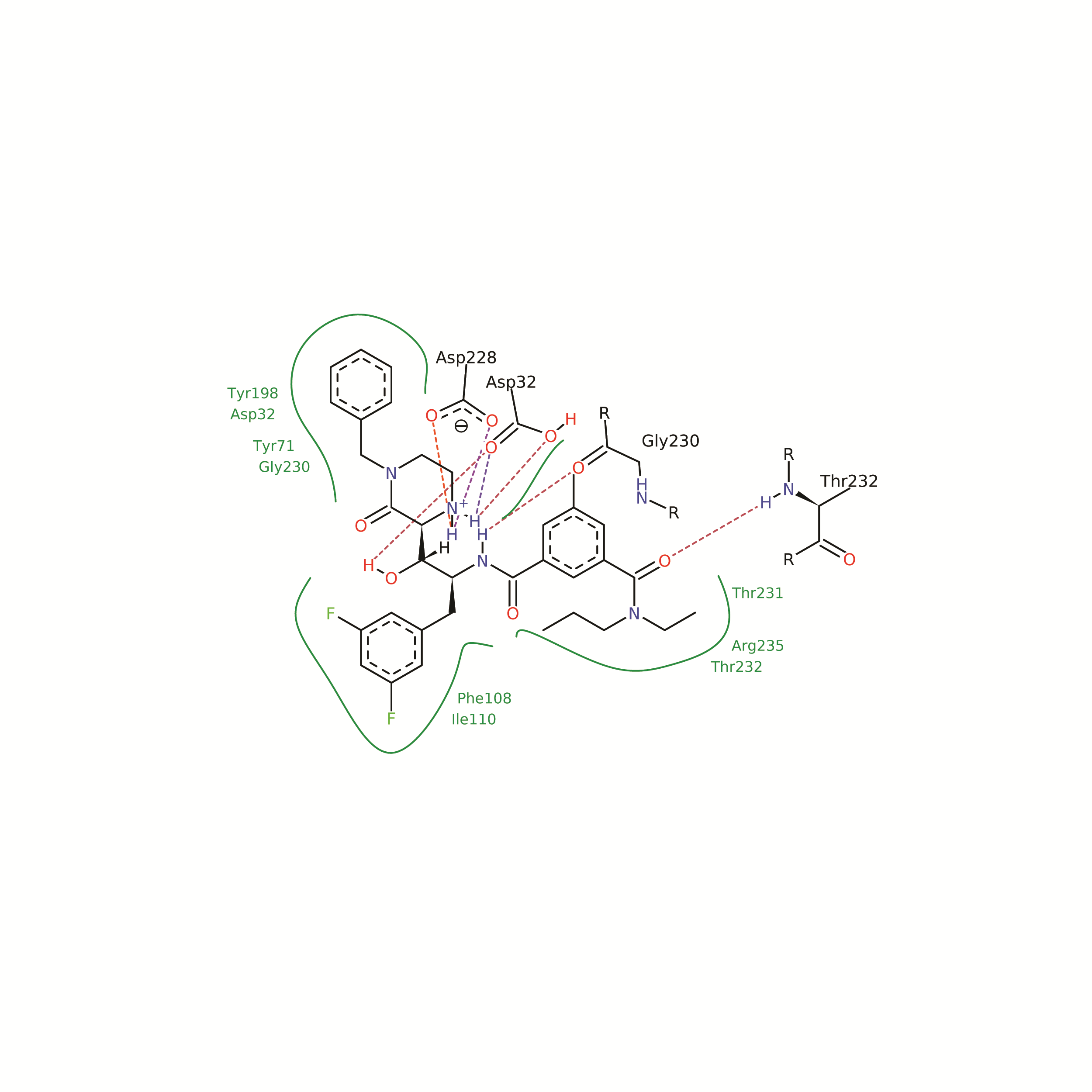 |
| 4FS4-10Q | -25.77 | -86.42 | 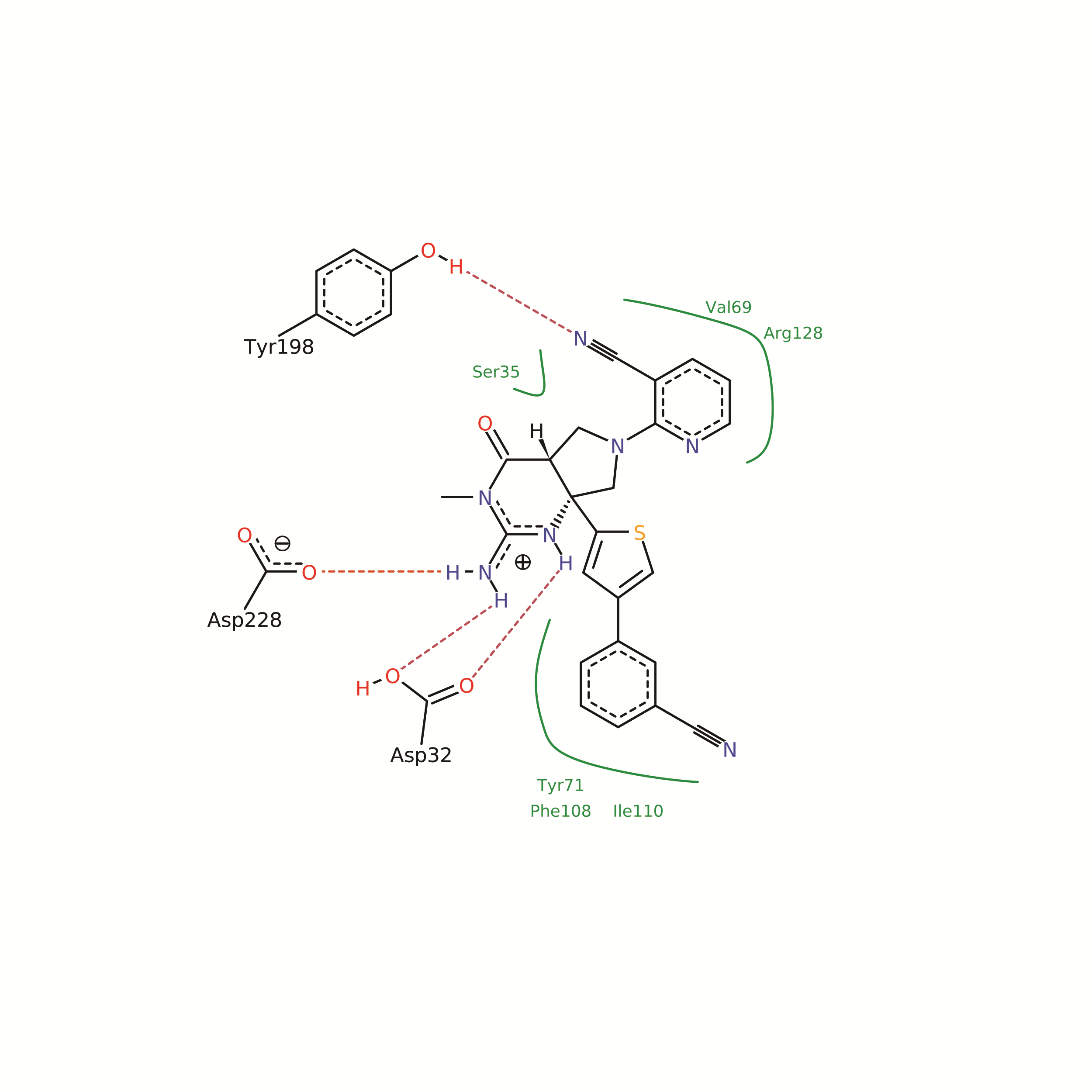 |
| 4FS4-0KQ | -23.76 | -60.97 | 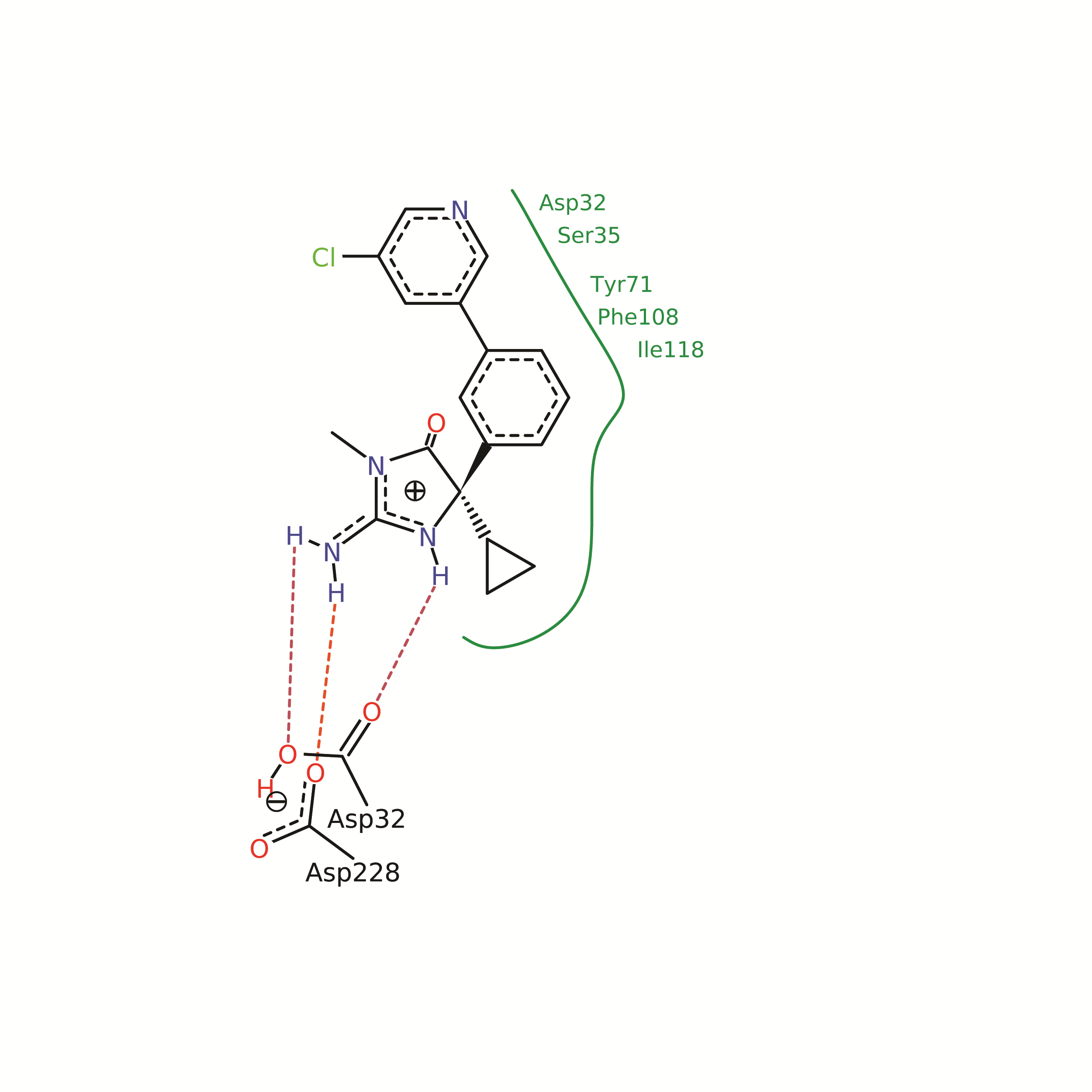 |
| 4FS4-13W | -21.14 | -67.32 | 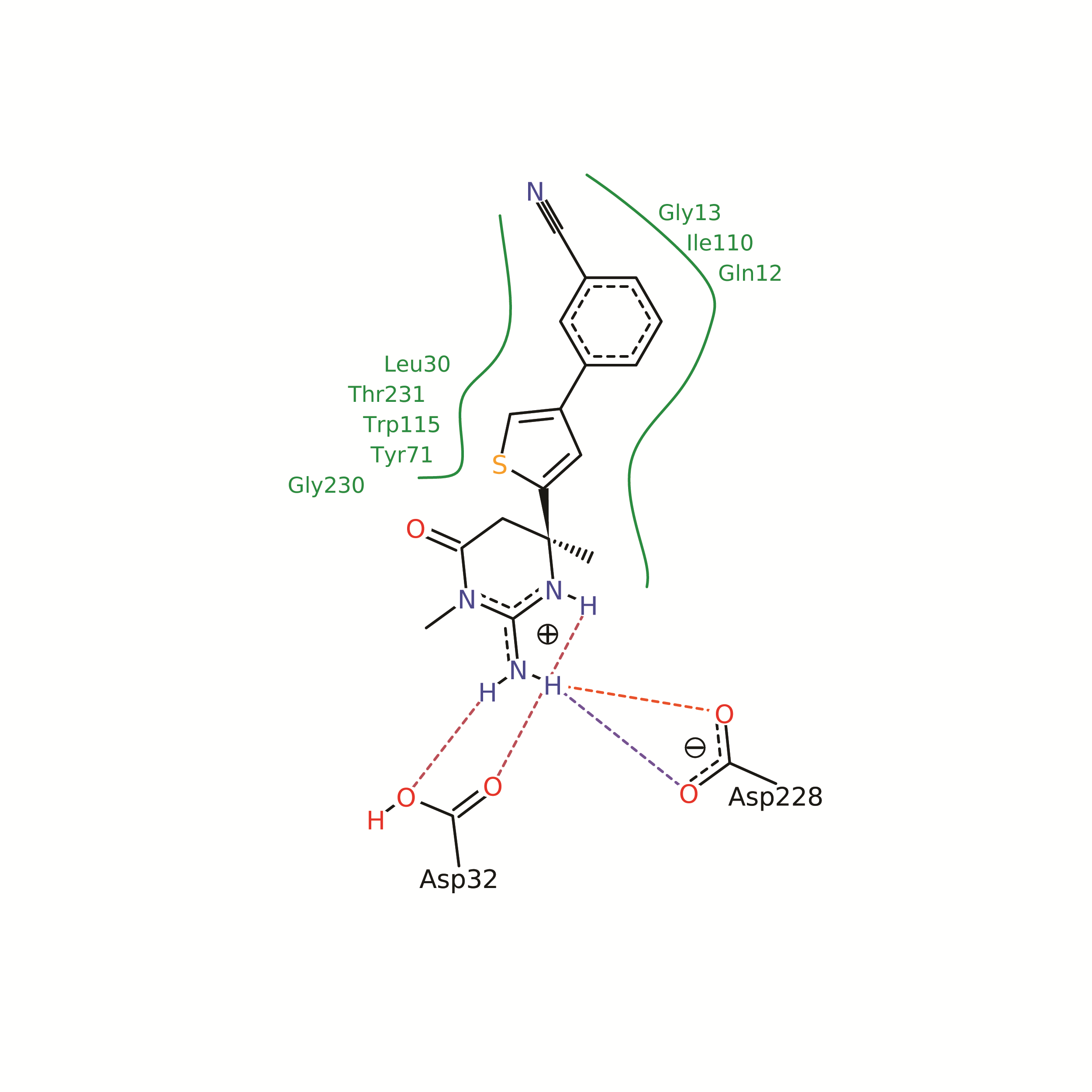 |
| 4FS4-H24 | -22 | -25.95 | 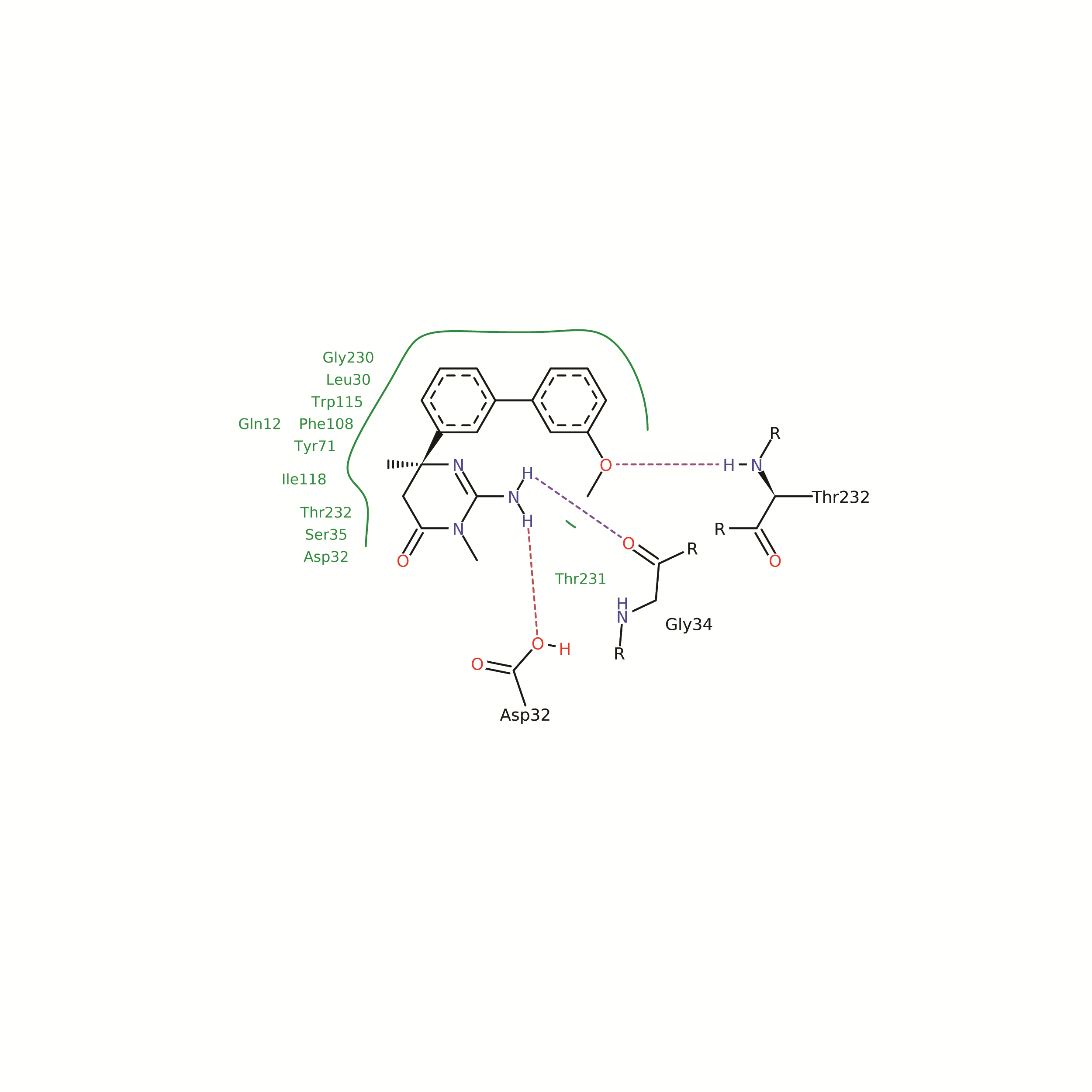 |
| **Cross-docking with 4FS4 using parameter 2** | | | |
| 4FS4-23I | -34.46 | -51.9 | 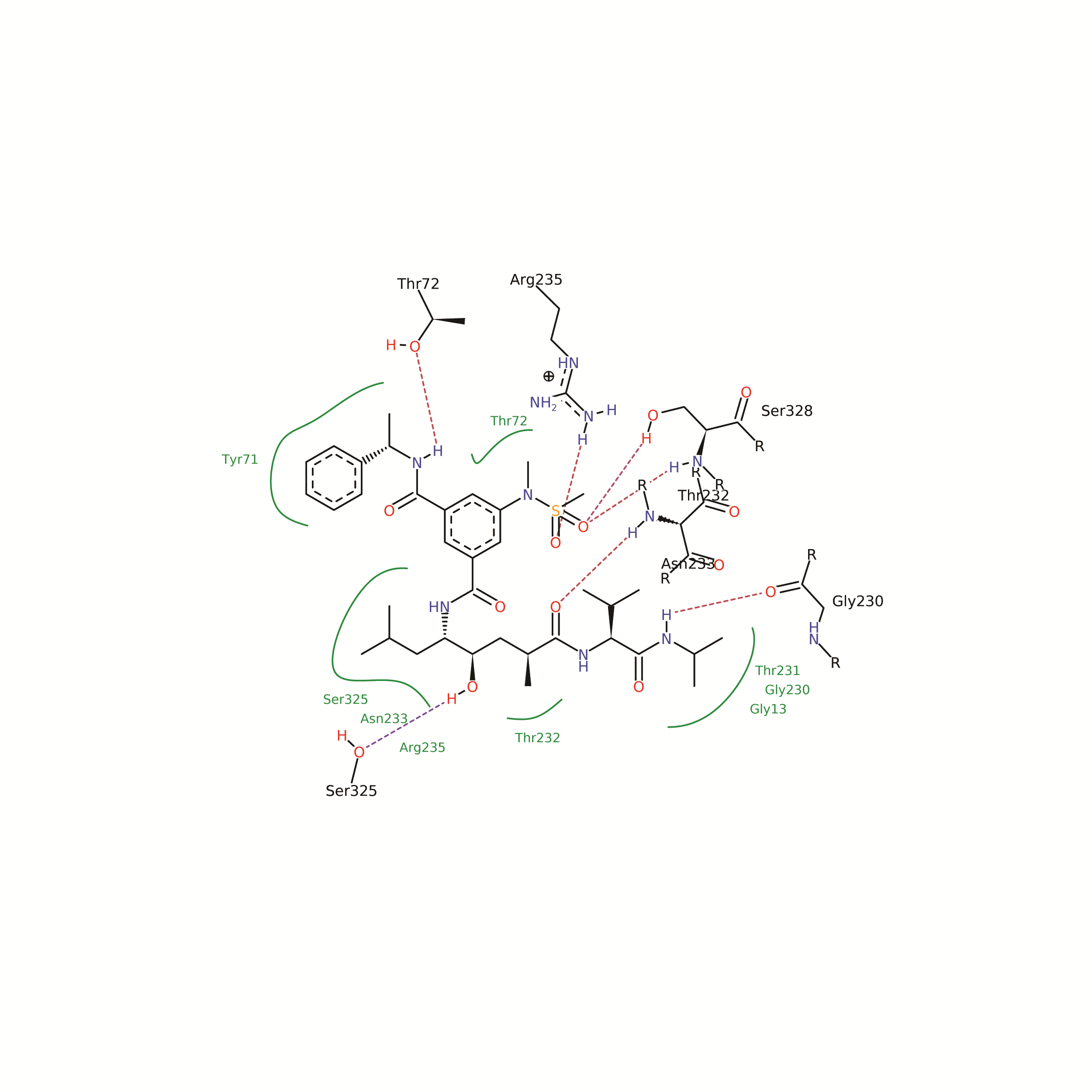 |
| 4FS4-SC6 | -34.51 | -81.55 | 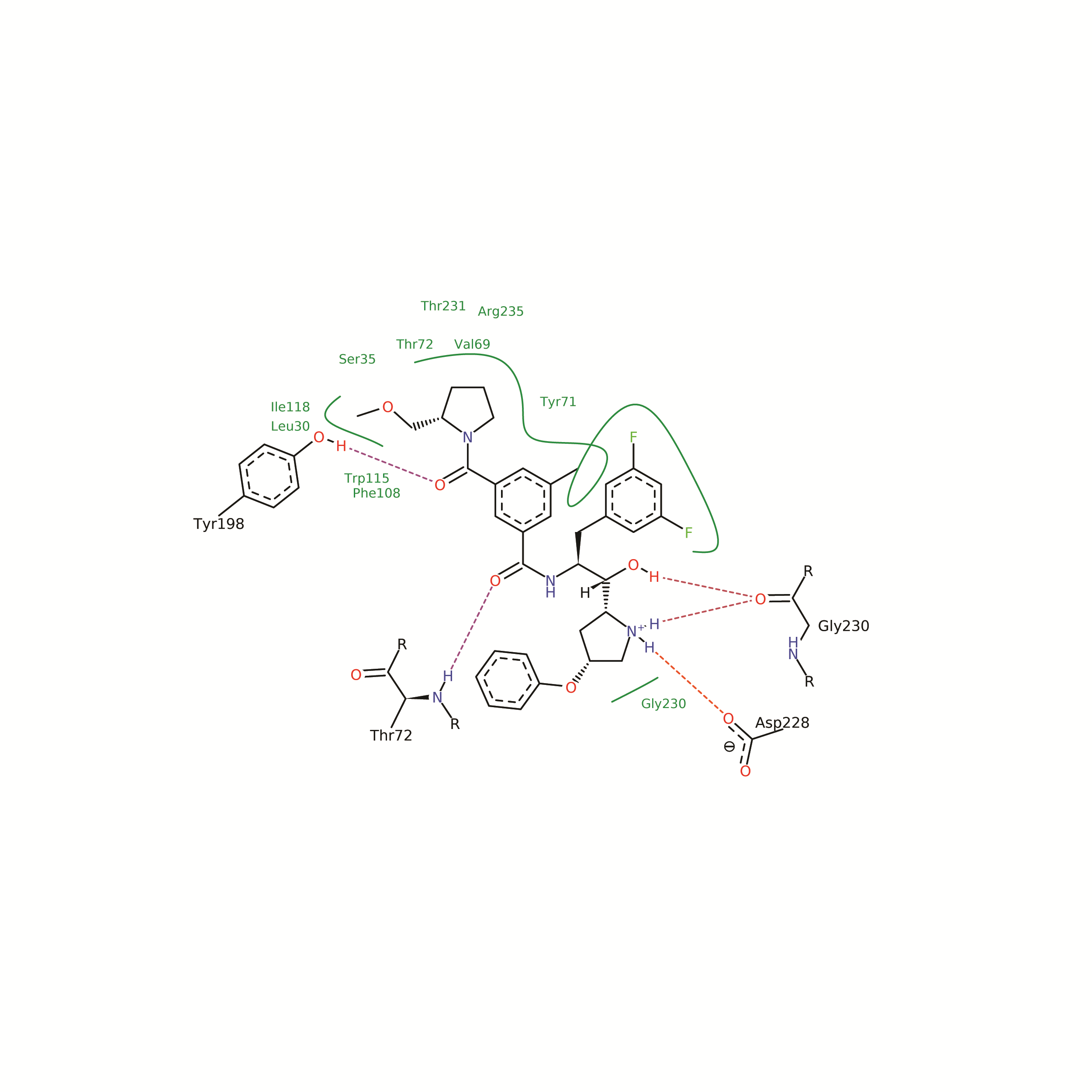 |
| 4FS4-Z76 | -30.86 | -50.28 | 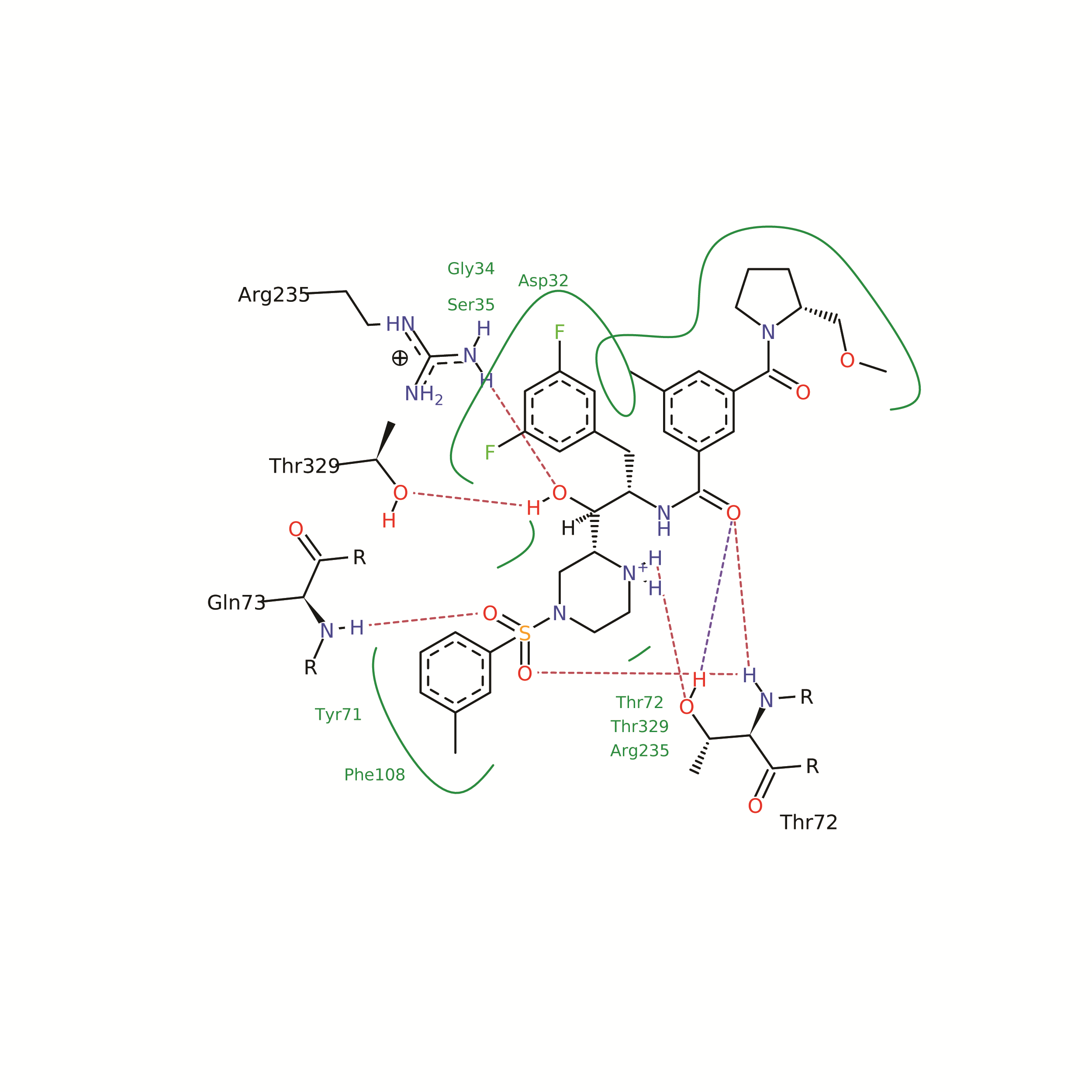 |
| 4FS4-316 | -28.76 | -71.6 | 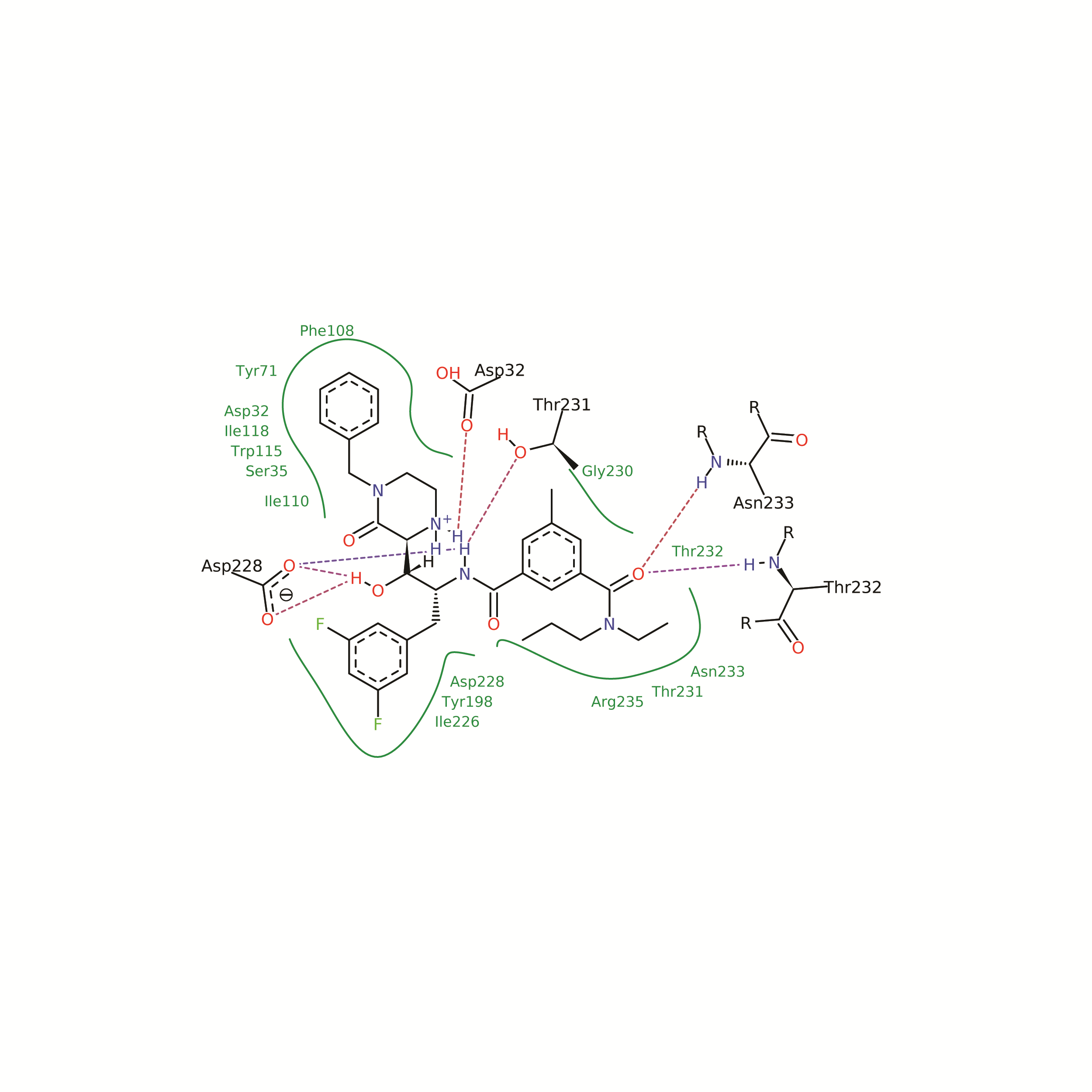 |
| 4FS4-10Q | -29.12 | -53.1 | 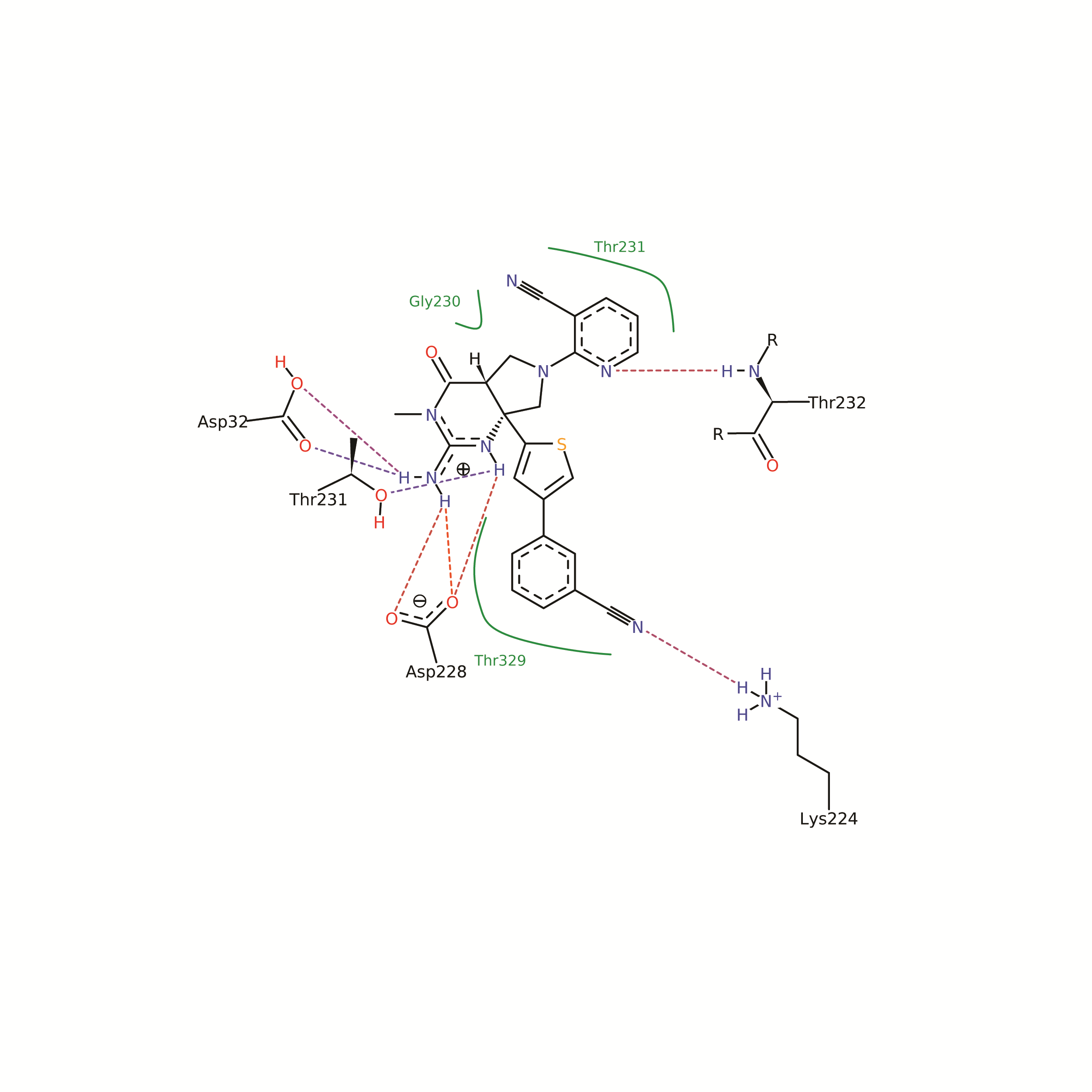 |
| 4FS4-0KQ | -23.76 | -60.97 | 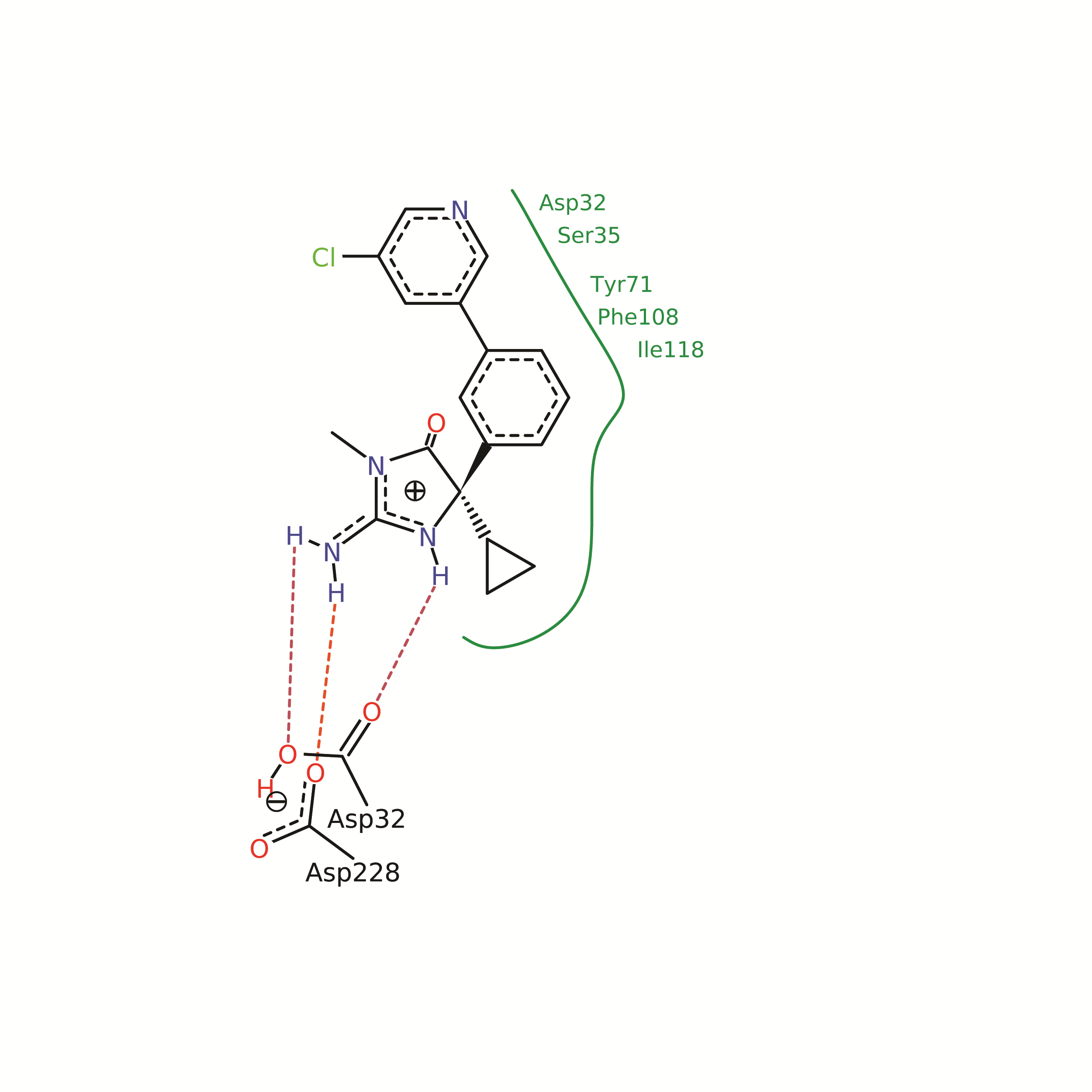 |
| 4FS4-13W | -20.67 | -66.8 | 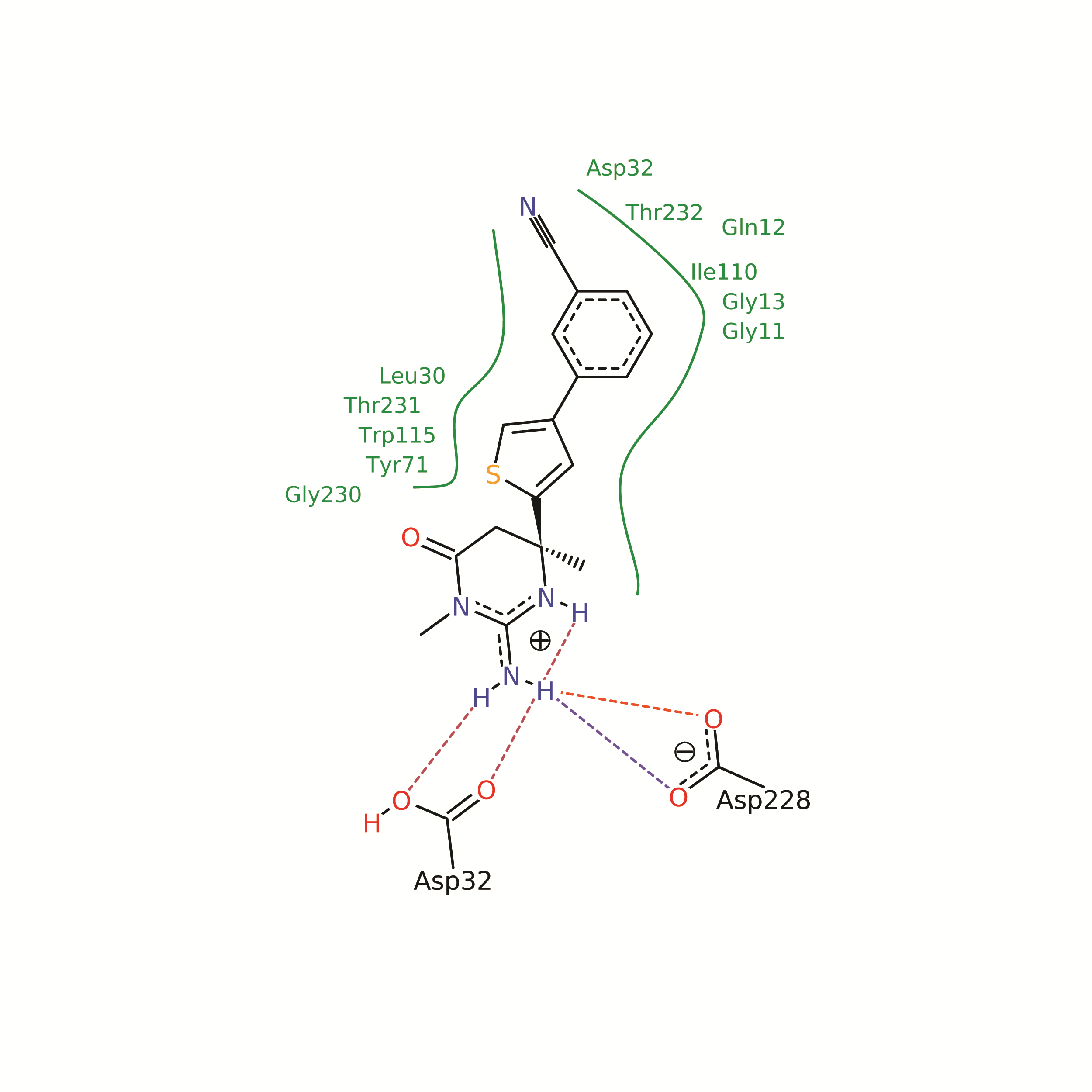 |
| 4FS4-H24 | -22 | -25.76 | 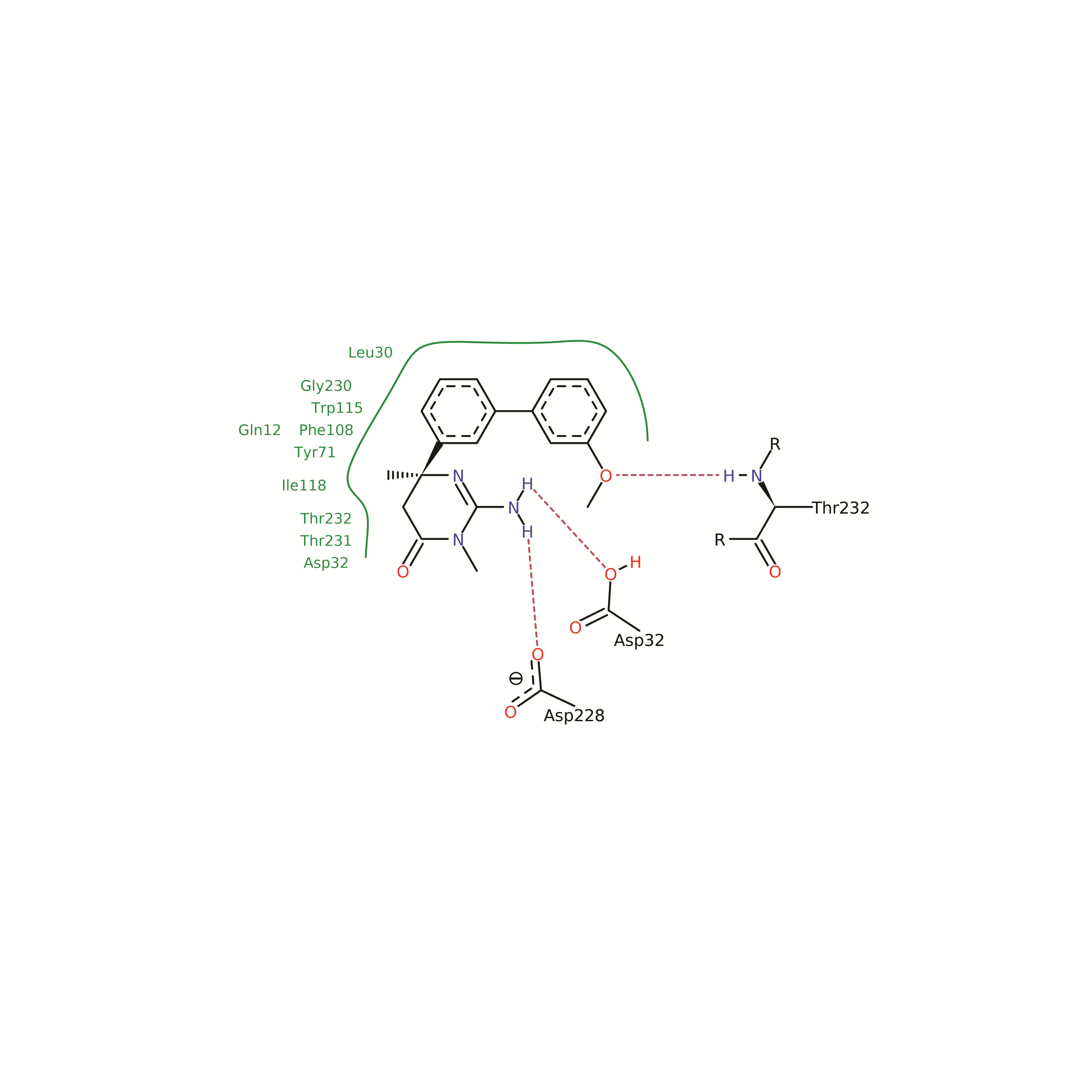 |
| **Cross-docking with 4FS4 using parameter 1** | | | |
| 4FS4-23I | -24.81 | -50.35 | 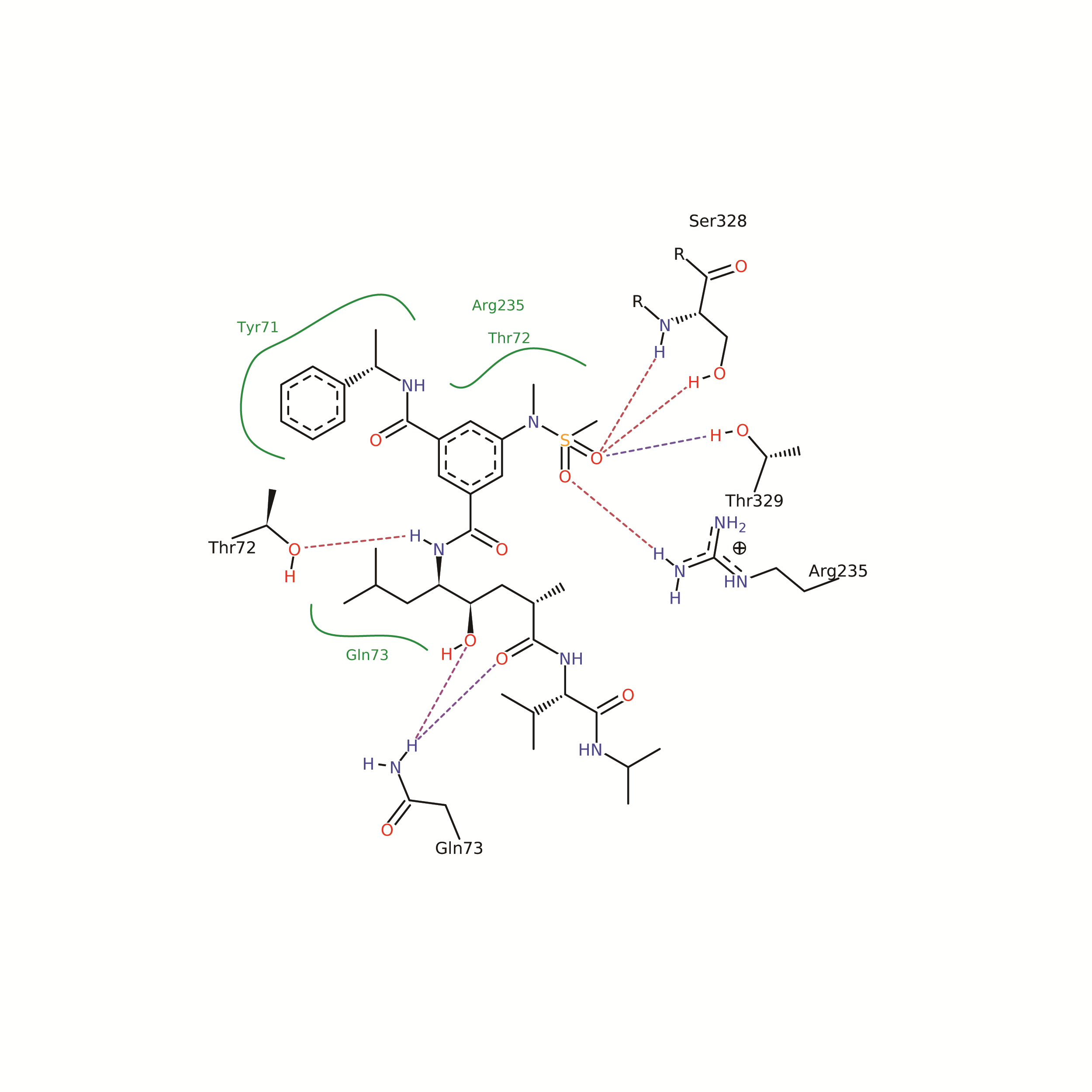 |
| 4FS4-SC6 | -37.81 | -55.23 | 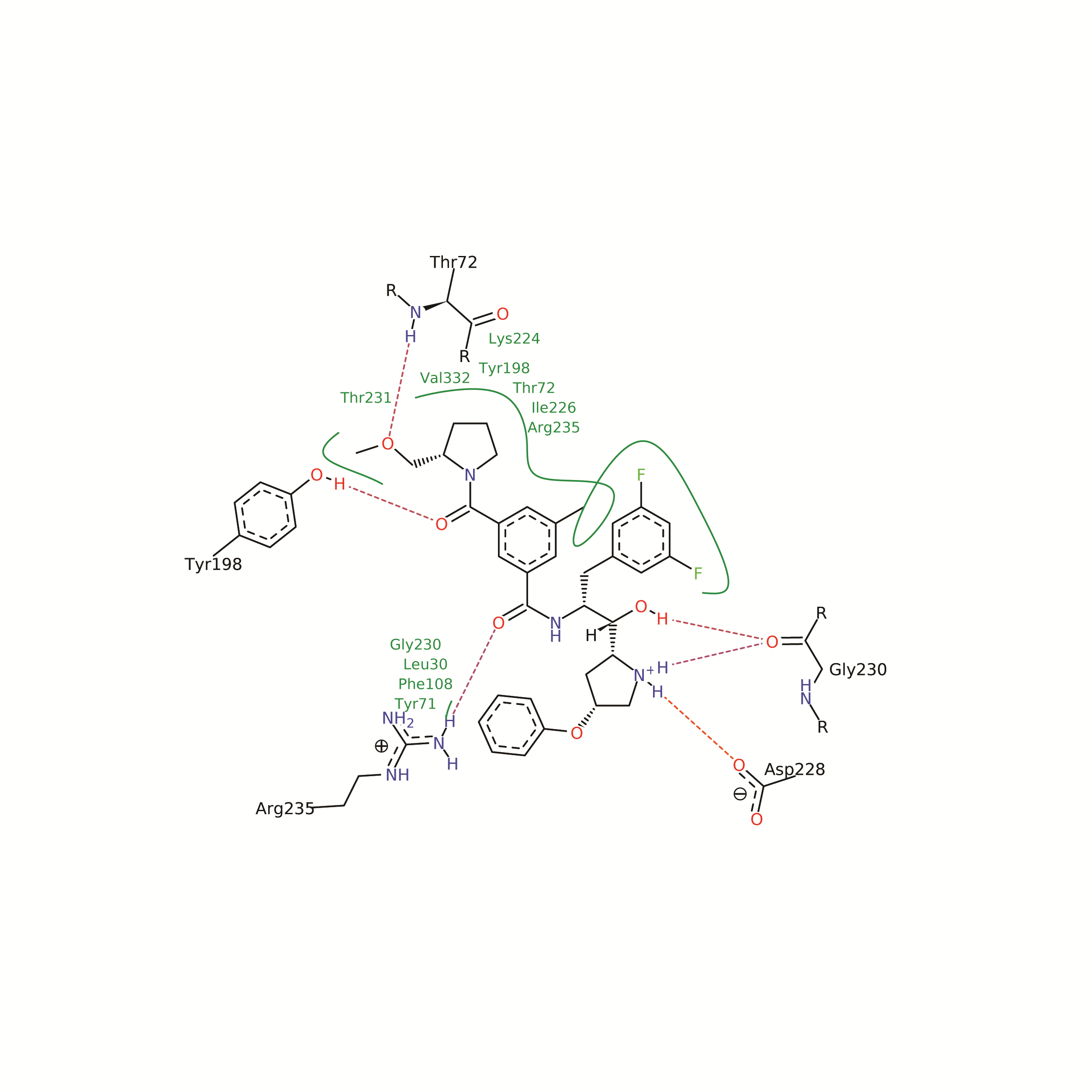 |
| 4FS4-Z76 | -39.32 | -70.44 | 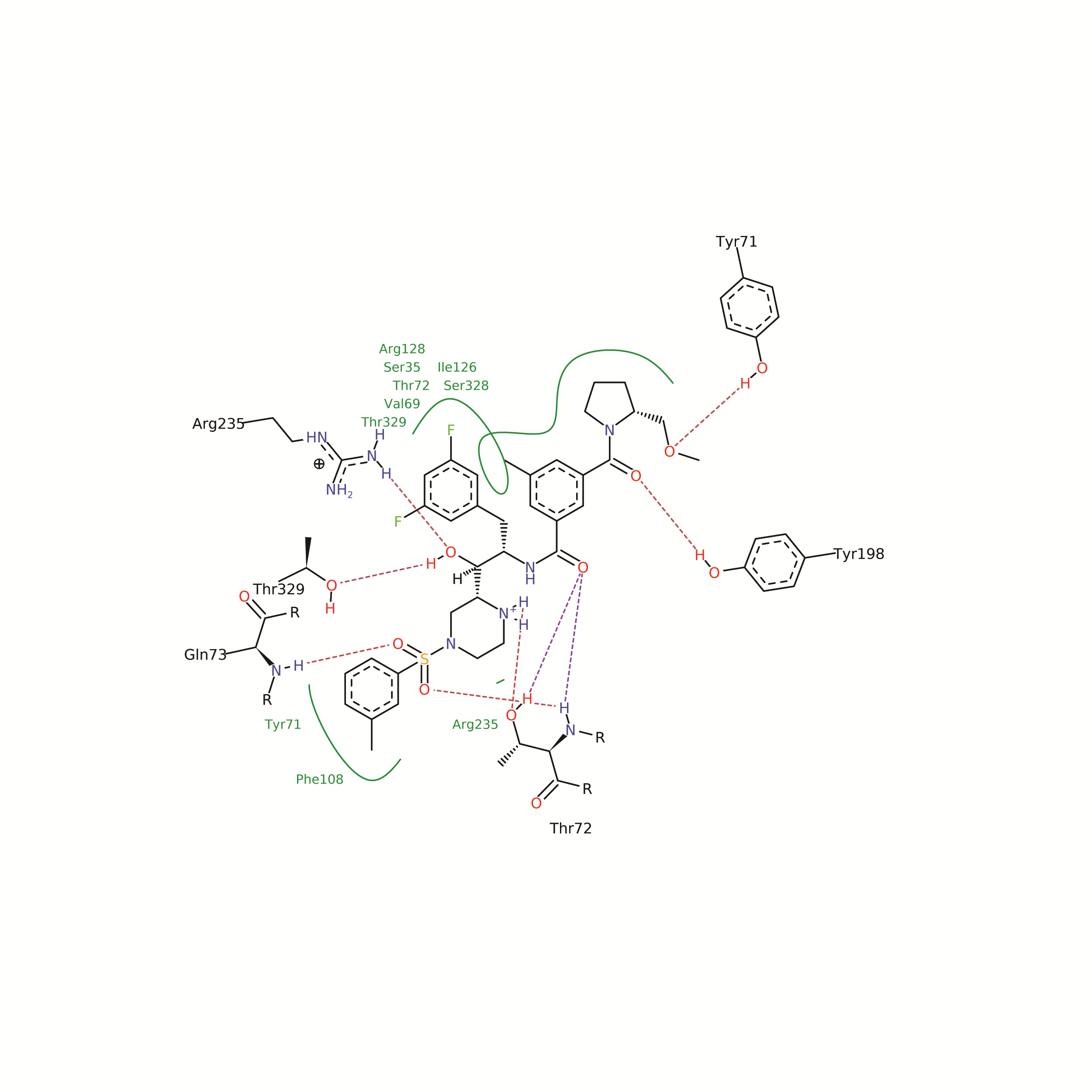 |
| 4FS4-316 | -34.92 | -87.32 | 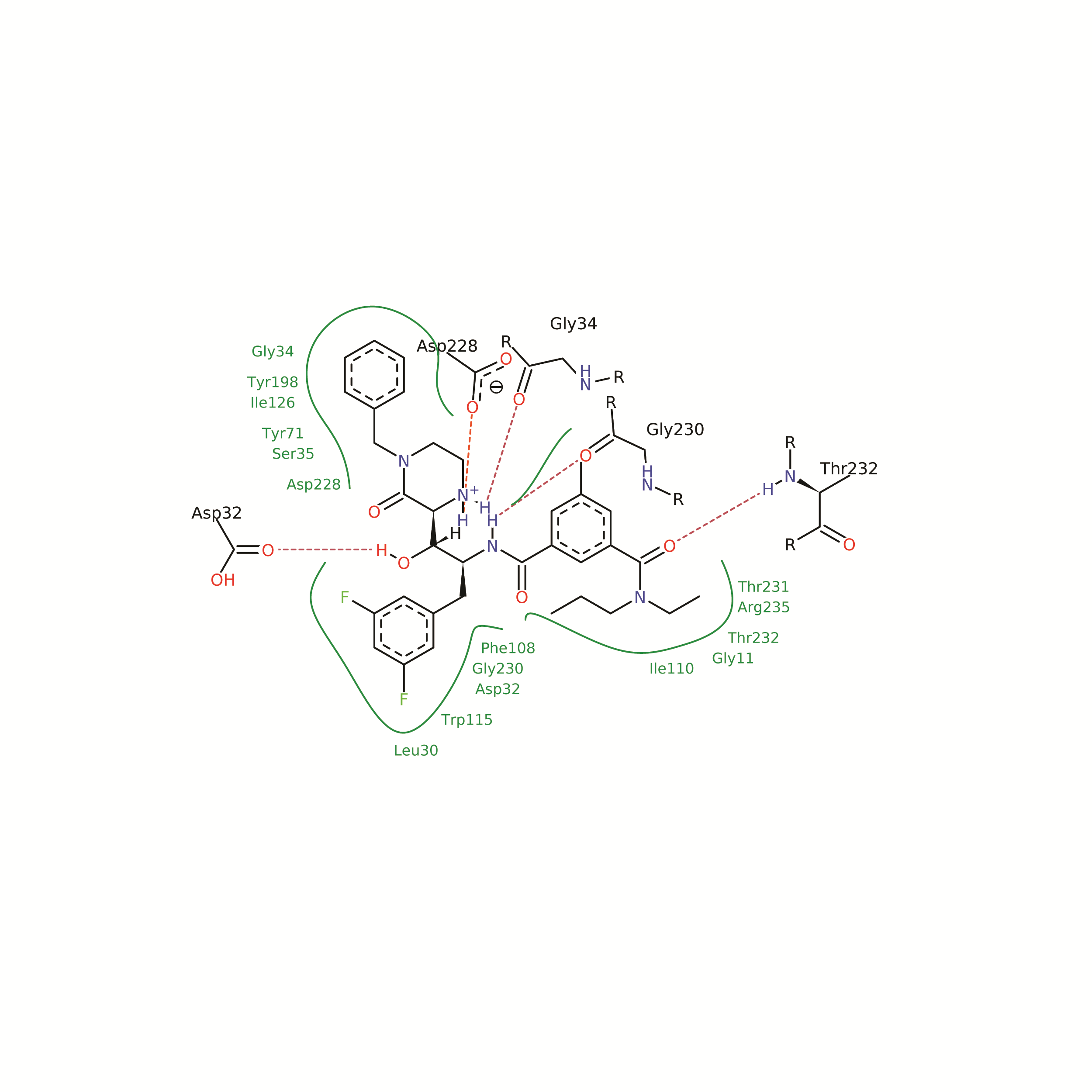 |
| 4FS4-10Q | -33.26 | -53.1 | 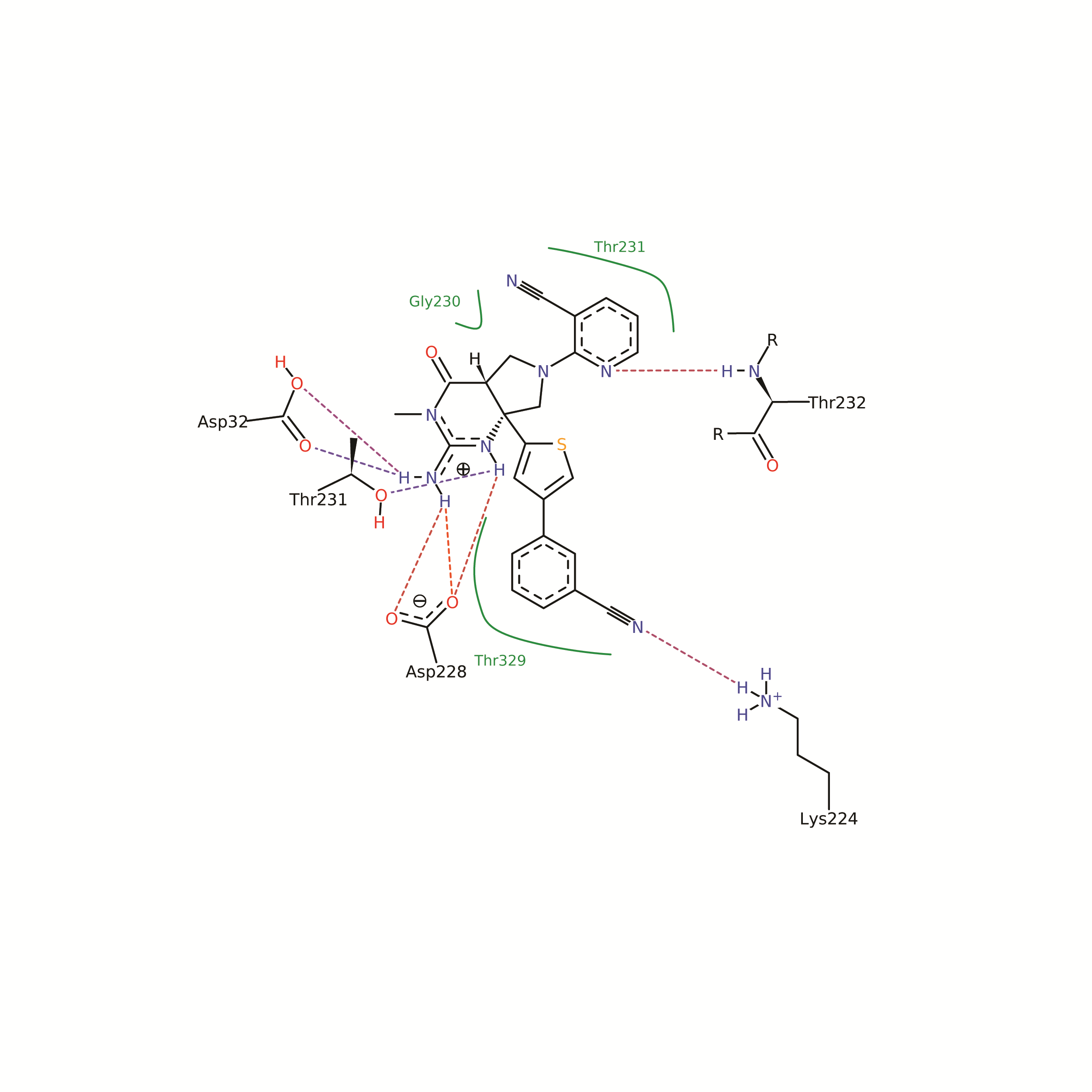 |
| 4FS4-0KQ | -23.76 | -60.97 | 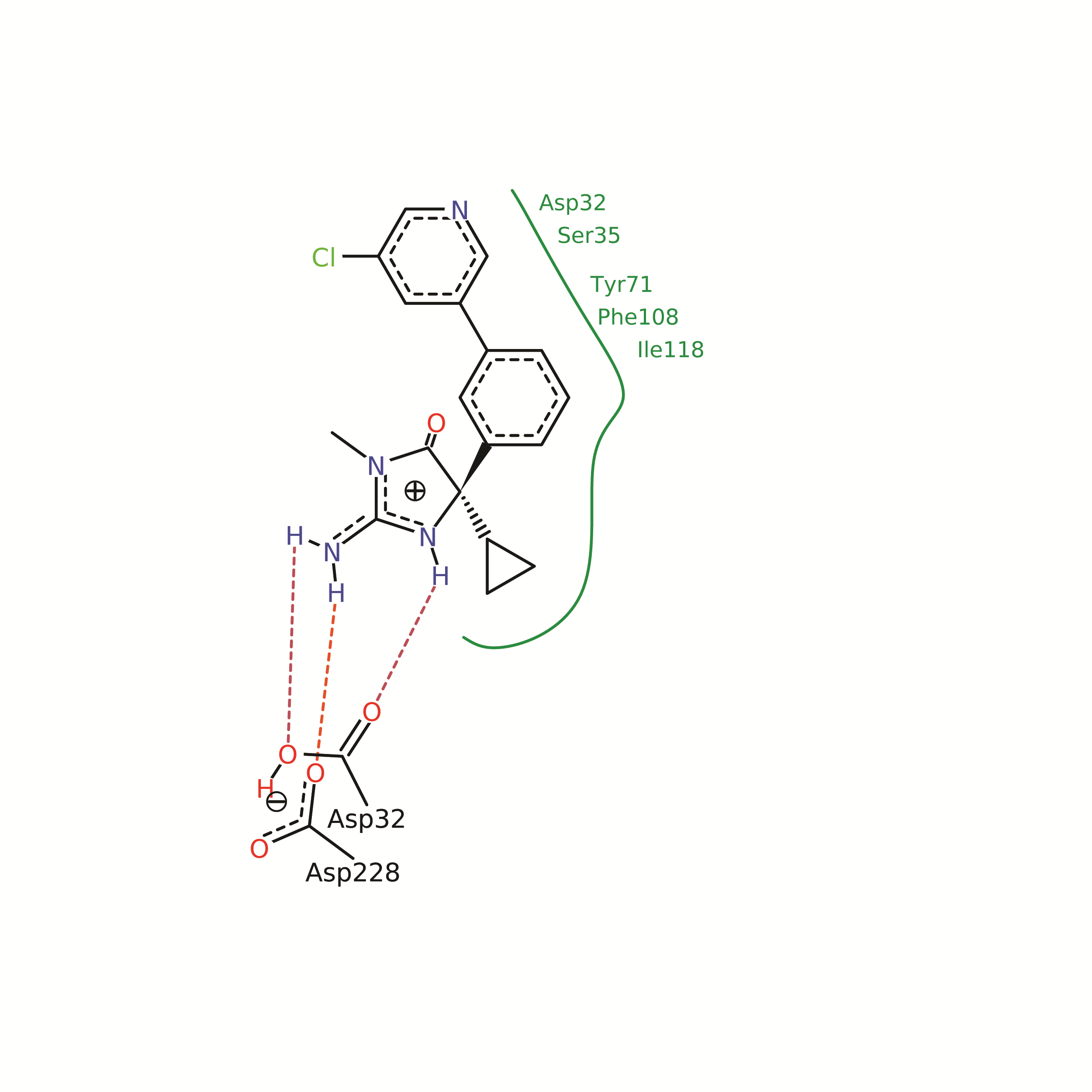 |
| 4FS4-13W | -21.14 | -67.32 | 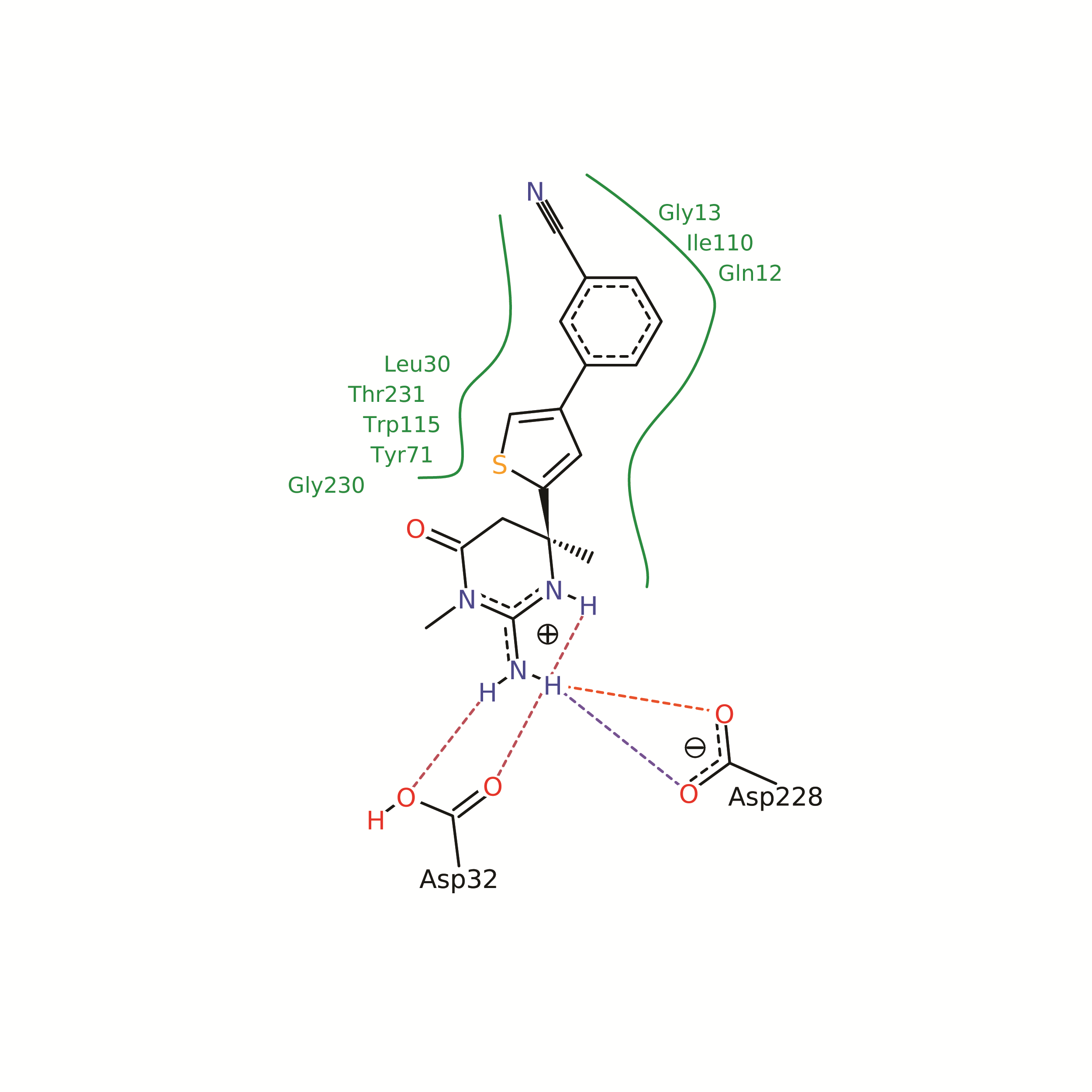 |
| **4FS4-H24** | -26.44 | -25.76 | 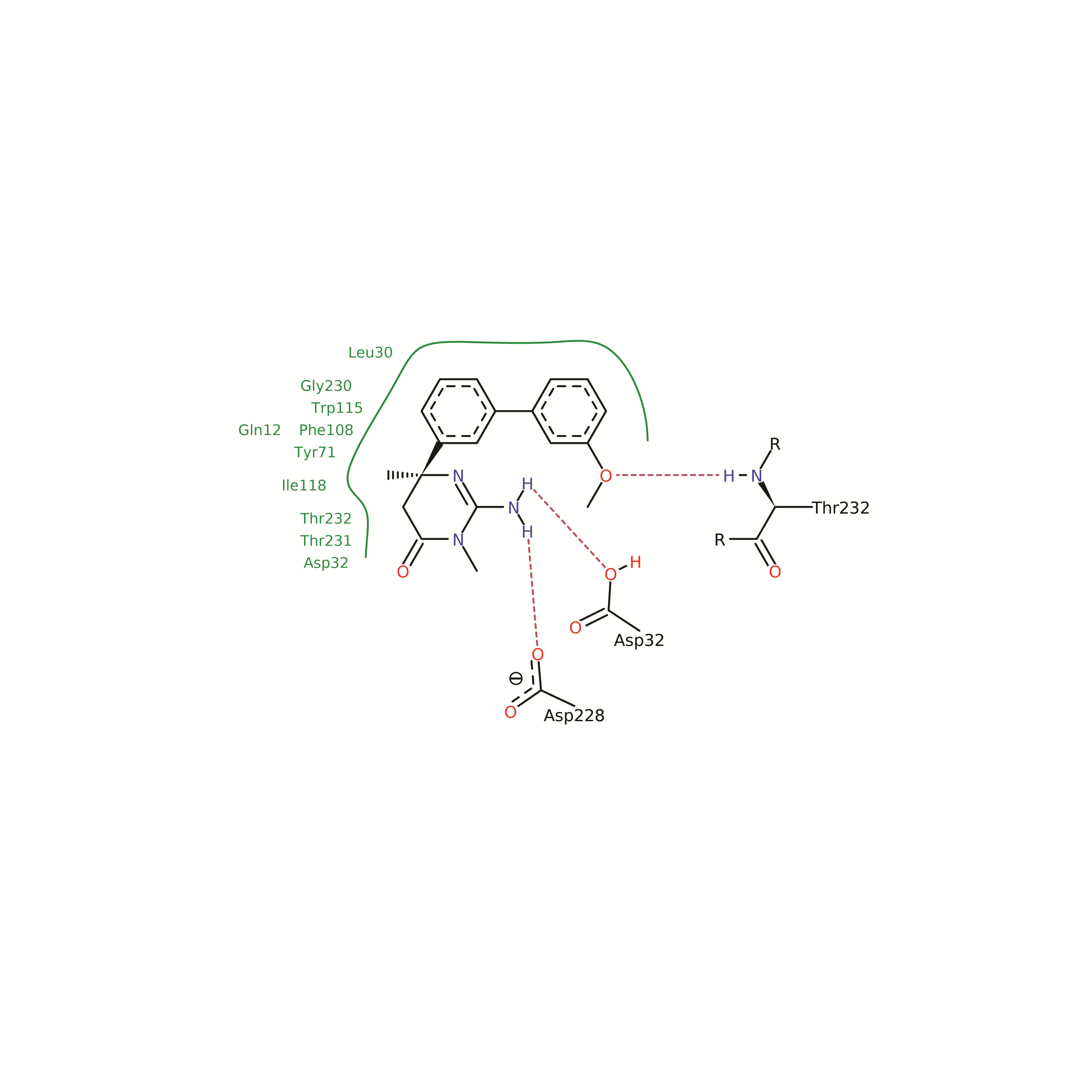 |
| **Cross-docking studies with 2QMG as receptor using parameter 3** | | | |
| 2QMG-23I | -37.61 | -65.82 | 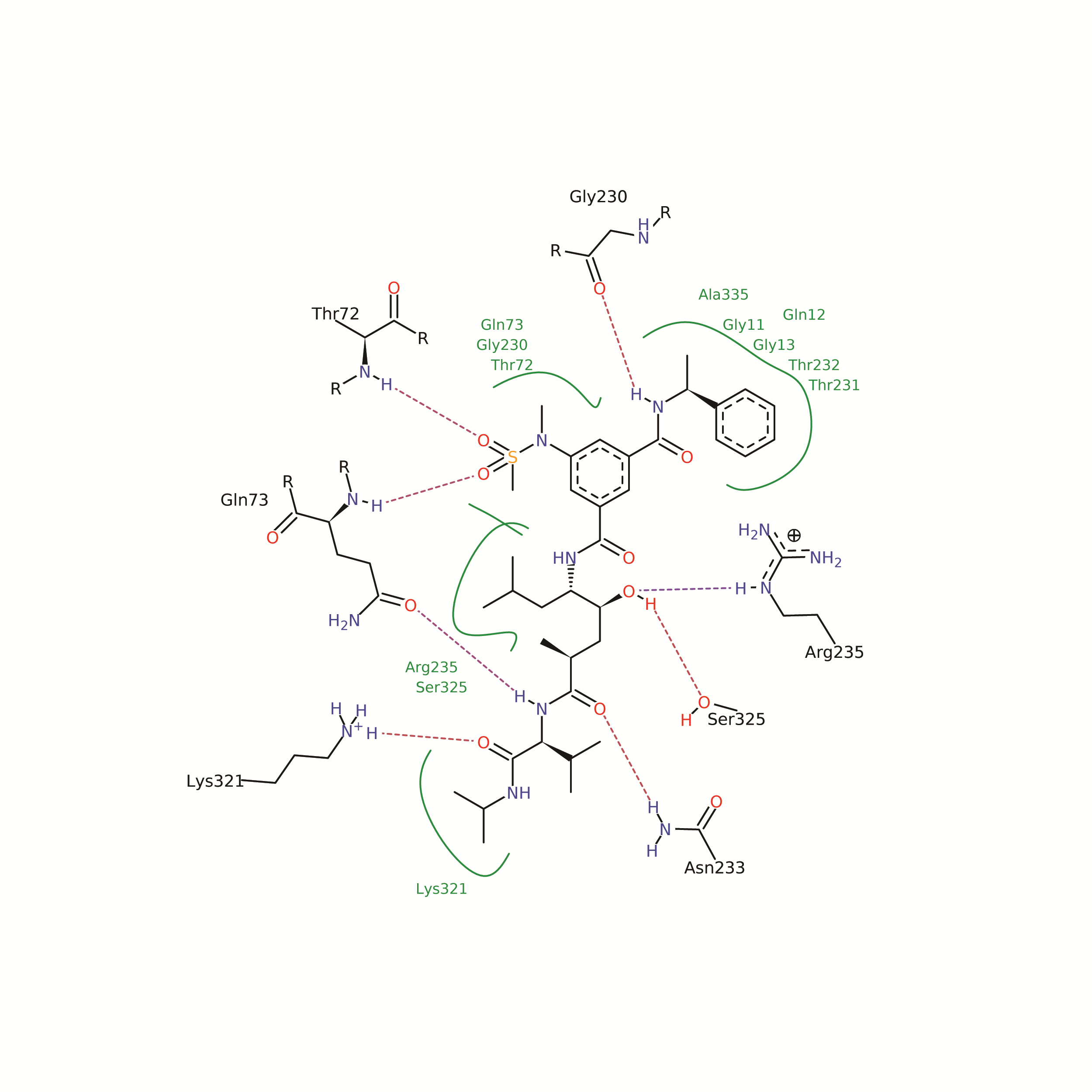 |
| 2QMG-SC6 | -47.99 | -111.67 |  |
| 2QMG-Z76 | -52.84 | -97.37 |  |
| 2QMG-316 | -44.02 | -111.38 |  |
| 2QMG-10Q | -23.96 | -59.63 |  |
| 2QMG-0KQ | -24.37 | -56.448 |  |
| 2QMG-13W | -30.03 | -80.95 |  |
| 2QMG-H24 | -22.81 | -37.12 |  |
| **Cross-docking with 2QMG as a receptor using parameter 2** | | | |
| 2QMG-23I | -34.04 | -64.24 |  |
| 2QMG-SC6 | -47.59 | -110.2 |  |
| 2QMG-Z76 | -52.84 | -104.72 |  |
| 2QMG-316 | -41.89 | -95.09 |  |
| 2QMG-10Q | -21.4 | -41.56 |  |
| 2QMG-0KQ | -21.54 | -51.85 |  |
| 2QMG-13W | -30.03 | -80.75 |  |
| 2QMG-H24 | -17.54 | -44.54 |  |
| **Cross-docking with 2QMG as a receptor using parameter 1** | | | |
| 2QMG-23I | -35.51 | -54.58 |  |
| 2QMG-SC6 | -47.99 | -115.78 |  |
| 2QMG-Z76 | -52.84 | -104.72 |  |
| 2QMG-316 | -43.8 | -80.992 |  |
| 2QMG-10Q | -27.23 | -41.56 |  |
| 2QMG-0KQ | -26.54 | -55.3 |  |
| 2QMG-13W | -30.03 | -80.79 |  |
| 2QMG-H24 | -17.54 | -44.54 |  |
| **Cross-docking studies with 3CIC as receptor using parameter 3** | | | |
| 3CIC-23I | -44.05 | -92.1 |  |
| 3CIC-SC6 | -38.25 |  |  |
| 3CIC-Z76 | -41.77 |  |  |
| 3CIC-316 | -41.51 | -86.102 |  |
| 3CIC-10Q | -26.22 | -42.48 |  |
| 3CIC-0KQ | -23.81 | -59.72 |  |
| 3CIC-13W | -32.18 | -73.94 |  |
| 3CIC-H24 | -22.09 | -23.58 |  |
| **Cross-docking with 3CIC as a receptor using parameter 2** | | | |
| 3CIC-23I | -32.39 | -54.7 |  |
| 3CIC-SC6 | -35.9 | -92.449 |  |
| 3CIC-Z76 | -37.27 | -85.5 |  |
| 3CIC-316 | -36.75 | -97.967 |  |
| 3CIC-10Q | -22.64 | -24.77 |  |
| 3CIC-0KQ | -23.81 | -59.72 |  |
| 3CIC-13W | -31.63 | -74.44 |  |
| 3CIC-H24 | -22.09 | -23.58 |  |
| **Cross-docking with 3CIC as a receptor using parameter 1** | | | |
| 3CIC-23I | -26.4 | -54.7 |  |
| 3CIC-SC6 | -42.99 | -87.55 |  |
| 3CIC-Z76 | -43.99 | -97.5 |  |
| 3CIC-316 | -49.88 | -99.4 |  |
| 3CIC-10Q | -27.8 | -24.77 |  |
| 3CIC-0KQ | -25.66 | -58.04 |  |
| 3CIC-13W | -35.42 | -74.44 |  |
| 3CIC-H24 | -24.23 | -23.58 |  |
| **Cross-docking studies with 3LPK as receptor using parameter 3** | | | |
| 3LPK-23I | -33.64 | -52.68 |  |
| 3LPK-SC6 | -42.18 | -110.082 |  |
| 3LPK-Z76 | -44.98 | -101.07 |  |
| 3LPK-316 | -44.35 | -103.83 |  |
| 3LPK-10Q | -26.69 | -72.79 |  |
| 3LPK-0KQ | -27.46 | -66.55 |  |
| 3LPK-13W | -32.71 | -71.73 |  |
| 3LPK-H24 | -26.78 | -46.95 |  |
| **Cross-docking with 3LPK using parameter 2** | | | |
| 3LPK-23I | -33.35 | -83.78 |  |
| 3LPK-SC6 | -39.78 | -113.87 |  |
| 3LPK-Z76 | -45.67 | -122.72 |  |
| 3LPK-316 | -39.76 | -89.62 |  |
| 3LPK-10Q | -26.69 | -72.79 |  |
| 3LPK-0KQ | -27.4 | -66.55 |  |
| 3LPK-13W | -32.71 | -71.73 |  |
| 3LPK-H24 | -21.47 | -37.57 |  |
| **Cross-docking with 3LPK as a receptor using parameter 1** | | | |
| 3LPK-23I | -37.66 | -73.98 |  |
| 3LPK-SC6 | -39.54 | -120.11 |  |
| 3LPK-Z76 | -53.721 | -98.328 |  |
| 3LPK-316 | -41.31 | 118.95 |  |
| 3LPK-10Q | -26.69 | -72.79 |  |
| 3LPK-0KQ | -27.4 | -66.55 |  |
| 3LPK-13W | -32.71 | -71.73 |  |
| 3LPK-H24 | -21.47 | -41.17 |  |

**Table S5**- Docking studies with the compounds used by Ghosh *et al.* for 2P4J

| BACE1-SAR compound complex | K_i_ | Match scores | MMGBSA ΔG | Interactions |
| --- | --- | --- | --- | --- |
| 2P4J-5a | 57.8 | -39.67 | -72.526 |  |
| 2P4J-5b | 1.8 | -44.03 | -70.072 |  |
| 2P4J-5c | 136 | -38.12 | -77.267 |  |
| 2P4J-5d | 1.1 | -44.2 | -90.316 |  |
| 2P4J-5e | 315 | -32.81 | -72.769 |  |
| 2P4J-5f | 438 | -37.9 | -47.437 |  |
| 2P4J-5g | 27 | -40.09 | -85.301 |  |
| 2P4J-22 | 788 | -35.68 | -79.31 |  |

**Table S6**- Docking studies with the compounds used by Mandal *et al.* for 4H3G

| BACE1-SAR Compound  compex | K_i_ | Match scores | MMGBSA ΔG | Interactions |
| --- | --- | --- | --- | --- |
| 4H3G-26 | 3 | -27.72 | -76.968 |  |
| 4H3G-27 | 2 | -28.5 | -82.358 |  |
| 4H3G-28 | 1 | -28.66 | -82.86 |  |
| 4H3G-31 | 90 | -22.5 | -82.554 |  |
| 4H3G-32 | 132 | -23.18 | -78.26 |  |
| 4H3G-33 | 26 | -21.38 | -79.845 |  |
| 4H3G-34 | 15 | -25.2 | -54.44 |  |
| 4H3G-36 | 8 | -24.699 | -61.54 |  |
| 4H3G-37 | 3 | -26.86 | -71.37 |  |
| 4H3G-38 | 6 | -26.3 | -84.064 |  |
| 4H3G-39 | 6 | -25.8 | -58.559 |  |
| 4H3G-40 | 1 | -30.02 | -50.22 |  |
| 4H3G-41 | 64 | -20.71 | -82.065 |  |

**Table S7.** Match scores and MM-GBSA energies as obtained by docking of the 23 compounds to 4H3G and 2P4J.

| Compound name and  2D- structures | Match Score (upper row) obtained by docking to 4H3G and the corresponding MMGBSA energies (lower row) | Match Score (upper row) obtained by docking to 2P4J and the corresponding MMGBSA energies (lower row) |
| --- | --- | --- |
| ZINC03175470 | -40.27 | -39.45 |
|  | -43.109 | -51.37 |
| ZINC31167296 | -32.82 | -41.46 |
|  | -100.92 | -89.8 |
| ZINC02106070 | -41.38 | -38.86 |
|  | -68.51 | -55.28 |
| ZINC53276039 | -32.6 | -38.81 |
|  | -69.78 | -67.1 |
| ZINC0518219 | -37.24 | -33.58 |
|  | -39.69 | -76.31 |
| ZINC2119155 | -31.48 | -30.3 |
|  | -69.67 | -60.19 |
| ZINC02138963 | -38.79 | -31.23 |
|  | -69.128 | -59.97 |
| ZINC02137169 | -35.18 | -39.67 |
|  | -58.11 | -62.63 |
| ZINC01758814 | -35.99 | -37.98 |
|  | -71.77 | -91 |
| ZINC16027834 | -38.82 | -37.59 |
|  | -54.58 | -77.46 |
| ZINC20410529 | -33.44 | -31.34 |
|  | -42.63 | -45.39 |
| ZINC9007942 | -49.73 | -39.7 |
|  | -68.34 | -77.2 |
| ZINC72319974 | -39.54 | -38.24 |
|  | -40.11 | -31 |
| ZINC02132317 | -36.33 | -29.63 |
|  | -63.5 | -86.97 |
| ZINC02105886 | -36.83 | -37.05 |
|  | -50.68 | -53 |
| ZINC01799835 | -36.08 | -32.65 |
|  | -43.36 | -51.05 |
| ZINC01817067 | -33.72 | -33.78 |
|  | -71.48 | -58 |
| ZINC06142057 | -38.40 | -39.73 |
|  | -45.76 | -86.4 |
| ZINC01794271 | -35.85 | -30.36 |
|  | -47.12 | -71 |
| ZINC20410507 | -30.98 | -30.76 |
|  | -47.51 | -86.7 |
| ZINC01773900 | -31.12 | -34.92 |
|  | -66.98 | -63 |
| ZINC39559782 | -31.93 | -32.08 |
|  | -44.6 | -50.68 |
| ZINC04024744 | -29.5 | -32.32 |
|  | -60.75 | -61.5 |

**Table S8.** Details of the 12 BACE1 inhibitors used for construction of Pharmacophore model 1. For the details of Pharmacophore model 2 the 9 BACE1 inhibitors used for its construction are listed in Table S2 (excluding the ligand of 2G94)

| PDB ID | 2D-structure | No of rotatable bonds | IC_50_ values in nM |
| --- | --- | --- | --- |
| VG0 |  | 17 | 33 |
| VG6 |  | 15 | 40 |
| ZYE |  | 13 | 23 |
| ZY4 |  | 11 | 13 |
| 318 |  | 16 | 5 |
| 3RS |  | 7 | 0.7 |
| RTN |  | 7 | 74 |
| BXQ |  | 12 | 49 |
| BXD |  | 14 | 2 |
| 996 |  | 11 | 44 |
| 916 |  | 11 | 8.5 |
| 1QT |  | 6 | 36 |
